# Base-Catalyzed, Solvent-Free Synthesis of Rigid V-Shaped Epoxydibenzo[*b*,*f*][1,5]diazocines

**DOI:** 10.1021/acs.joc.1c00884

**Published:** 2021-06-23

**Authors:** Michał Michalak, Bartosz Bisek, Michał Nowacki, Marcin Górecki

**Affiliations:** Institute of Organic Chemistry, Polish Academy of Sciences, Kasprzaka 44/52, 01-224 Warsaw, Poland

## Abstract

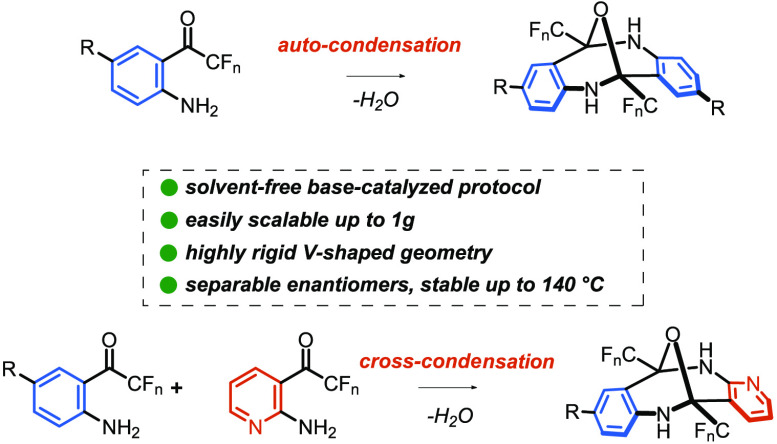

A novel method for the synthesis of epoxydibenzo[*b*,*f*][1,5]diazocines exhibiting a V-shaped molecular architecture is reported. The unique approach is based on unprecedented base-catalyzed, solvent-free autocondensation and cross-condensation of fluorinated *o*-aminophenones. The structure of the newly synthesized diazocines was confirmed independently by X-ray analysis and chiroptical methods. The rigidity of the diazocine scaffold allowed for the separation of the racemate into single enantiomers that proved to be thermally stable up to 140 °C. Furthermore, the inertness of the diazocine scaffold was demonstrated by performing a series of typical transformations, including transition metal-catalyzed reactions, proceeding without affecting the bis-hemiaminal subunit.

## Introduction

**Scheme 1:**
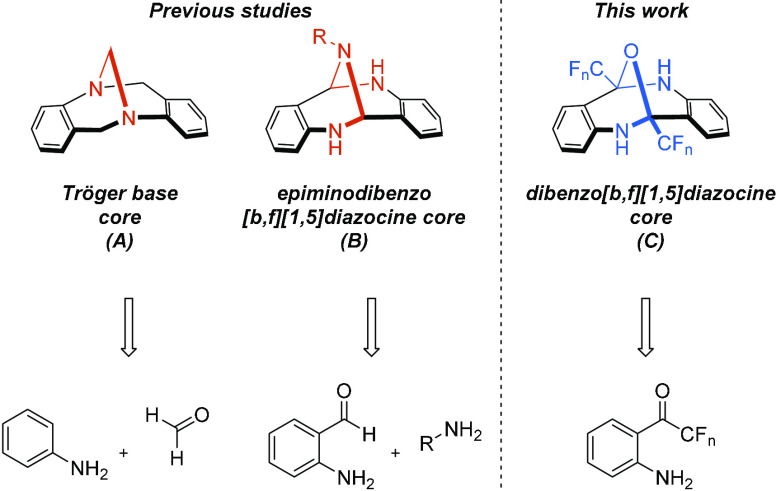
Examples of Rigid Molecules with V-Shaped Geometry

Molecules possessing rigid structures with defined curvature constitute the underpinnings of the rapidly growing supramolecular chemistry area. Those small building blocks decorated with appropriate functional groups enable the formation of higher-order structures as a result of self-assembly, which is of use in many areas. The most impressive examples include molecular tweezers,^1^ capsules,^2^ or cages.^3^ Among the molecular building blocks mentioned above, marked interest has been focused on Tröger’s base, a small molecule with a great history (structure A in Scheme 1). Its unique rigid V-shaped structure, confirmed almost 50 years after its first synthesis,^4^ has spawned an enormous number of applications in many areas, such as molecular recognition,^5^ metal catalysis, and organometallic^6^ and medicinal chemistry.^7^ The basis for widespread application arises from its trivial synthesis, carried out directly from aniline (or its derivatives) and paraformaldehyde (Scheme 1). This clearly underlines that each new, easily accessible building block for the construction of the supramolecular architecture stimulates an enormous development of the field. For these reasons, sustainable, practical methods for the synthesis of bent molecular bricks are still highly desirable.

The long-standing interest in Tröger’s base (A) has recently culminated in the efficient synthesis of its heteroatom analogues, epiminodibenzodiazocines, bearing a nitrogen bridge (structure B in Scheme 1).^8^ In contrast, to the best of our knowledge, no efficient synthetic approach to diazocines, bearing an oxygen bridge, has been developed so far (structure C in Scheme 1). We anticipated that the missing link should be easily provided by the autocondensation of *o*-aminophenones, providing a new scaffold for supramolecular chemistry. A careful inspection of the literature data revealed that epoxydibenzo[*b*,*f*][1,5]diazocines have been isolated as byproducts in the synthesis of heterocycles^9^ and natural product degradation studies in some cases, albeit in marginal yields.^10^ Herein, we wish to report an unprecedented solvent-free synthesis of epoxydibenzo[*b*,*f*][1,5]diazocines with a well-defined, rigid V-shaped structure from fluorinated *o*-aminophenones.

## Results and Discussion

Our working hypothesis was based on the assumption that fluorinated *o*-aminophenones could undergo base-catalyzed self-condensation (Table 1). To prove this, we initially screened more than 10 solvents of different polarity in the autocondensation reaction of **1a**, catalyzed by *N*,*N*,*N′*,*N′*-tetramethylguanidine (TMG) (for details, see Scheme S1). The respective diazocine **2a** was formed in all cases in good to excellent yield without the concomitant formation of byproducts such as imine or cyclic bisimine (Table 1). Astonishingly, the best results in terms of conversion and yield were achieved under solvent-free conditions, leading to diazocine **2a** in 93% yield on a 0.5 mmol scale and finally in 95% yield on a 4.5 mmol scale. It should be mentioned that nitrogen-protected aminophenones **1b–d**, including an acidic sulfonamide (**1d**), failed to react, whereas *o*-aminobenzaldehyde decomposed completely under solvent-free conditions (for details, see the Supporting Information).

**Table 1:**
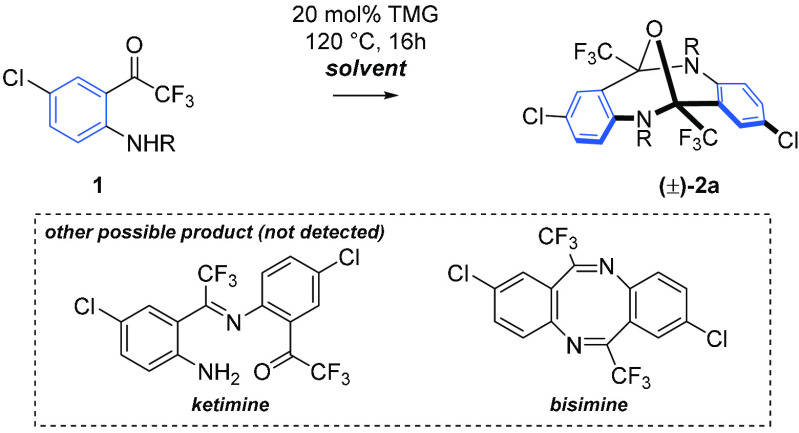
Effect of the Solvent in the Formation of Epoxydibenzo[*b*,*f*][1,5]diazocine (±)-**2a**

entry	R	solvent	conversion (%)^a^	yield of **2a** (%)^b^
1	H (**1a**)	DMF	94	87
2	H (**1a**)	DMSO	97	84
3	H (**1a**)	Py	90	86
4	H (**1a**)	MeCN	99	87
5	H (**1a**)	TMG	98	18
6	H (**1a**)	(CH_2_OH)_2_	98	73
7	H (**1a**)	*i*-PrOH	98	72
8	H (**1a**)	*n*-BuOH	84	29
9	H (**1a**)	water	97	29
10	H (**1a**)	1,4-dioxane	74	65
11	H (**1a**)	DCE	37	15
12	H (**1a**)	toluene	61	49
13	H (**1a**)	*n*-heptane	44	24
14	H (**1a**)	–	99	93 (95^c^)
15	PMB^d^ (**1b**)	–	<5	<5
16	Tr^e^ (**1c**)	–	<5	<5
17	Ts^f^ (**1d**)	–	<5	<5

a Conversion based on GC; naphtalene as the internal standard; all reactions conducted on a 0.5 mmol scale.

b Yield estimated from the calibration curve.

c On a 4.5 mmol scale.

d PMB, *p*-methoxybenzyl.

e Tr, trityl.

f Ts, *p*-toluenesulfonyl.

**Scheme 2:**
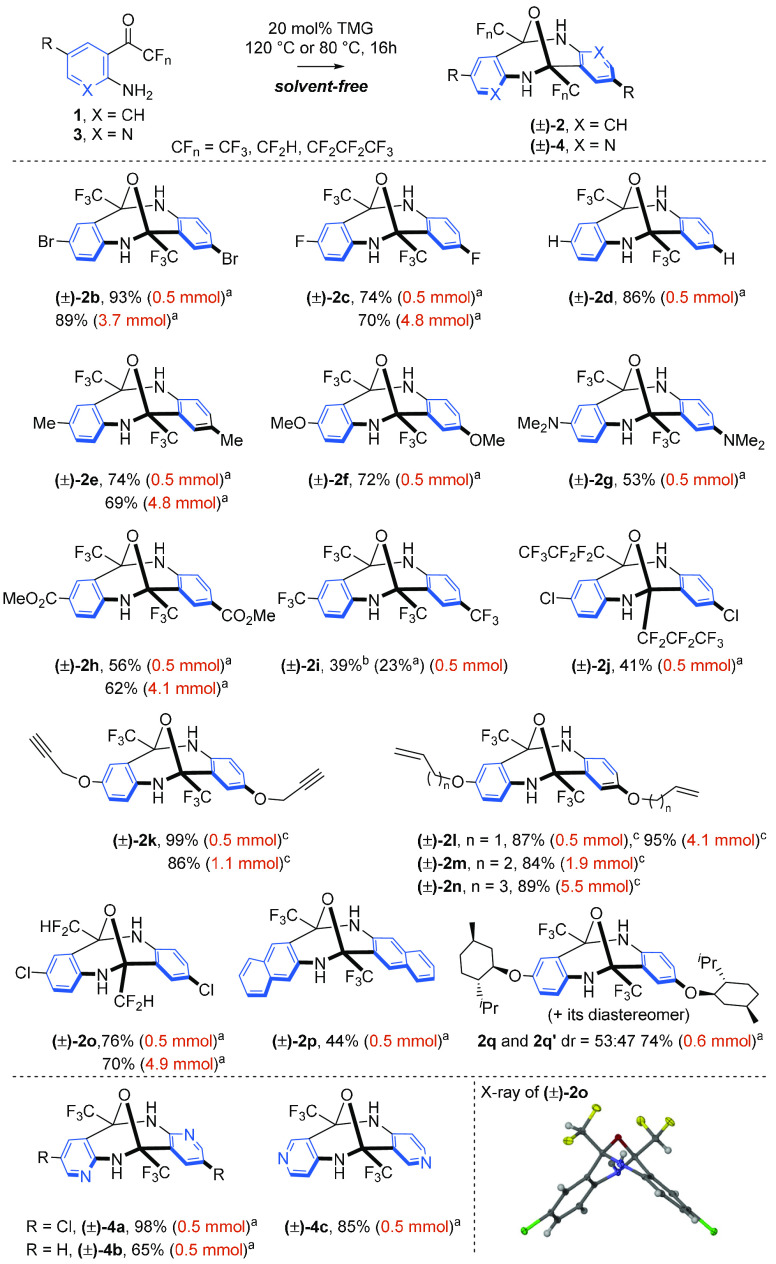
1,1,3,3-Tetramethylguanidine (TMG)-Catalyzed Solvent-Free Autocondensation of Aminophenones For 16 h at 120 °C. For 48 h at 120 °C. For 16 h at 80 °C.

With the optimal conditions secured, the scope of the method was explored. First, a group of trifluoromethyl aminophenones bearing electron-withdrawing and electron-donating groups in the *para* position to the nitrogen were investigated (Scheme 2). Generally, the formation of epoxydibenzo[*b*,*f*][1,5]diazocines proceeded in high yields, and the presence of halogen atoms (including fluorine) **2c**, alkyl ester **2h**, dimethylamine **2g**, or methoxy function **2f** was admirably tolerated. Only incorporation of the trifluoromethyl group that exerts a strong positive σ-inductive effect has delivered diazocine **2i** in a low 23% yield. A further increase in the reaction time to 48 h slightly increased the yield to 39% (for details, see the Experimental Section). Uniformly, diazocine **2j** carrying a perfluorinated side chain was also isolated in a moderate 41% yield. In contrast, the presence of a methyl group and alkoxy side chains bearing alkene or alkyne moieties afforded smoothly diazocines **2k–n**. Notably, the autocondensation of alkene- or alkyne-derived aminophenones had to be performed at a lower temperature (80 °C) to maintain the high yield. The application of enantiomerically pure aminophenones has met with partial success, leading cleanly to diazocine **2q**. However, an almost equimolar mixture of diastereomers was detected by ^19^F NMR. Further studies revealed that less nucleophilic aminopyridine derivatives could also participate in the autocondensation to give diazocines **4a–c**.

Next, we examined whether a more challenging, slightly acidic difluoromethyl ketone,^11^ prone to undergoing enolization and subsequent aldol reaction, could be involved in the TMG-catalyzed autocondensation (TMG; p*K*_a_ ≈ 15.2 in H_2_O).^12^ The incorporation of the CF_2_H group into organic molecules has received a great deal of attention in medicinal chemistry^13^ due to its ability to act as a lipophilic hydrogen bond donor modifying permeability, binding affinity, and bioavailability.^14^ The engagement in weak interactions offers an ideal platform for the construction of higher-order molecular scaffolds.^15^ To our delight, diazocine **2o** was formed in 70% yield under basic conditions without competing side reactions. The unique, rigid V-shaped structure was further evidenced by X-ray analysis (Scheme 2, structure **2o**) showing a perpendicular arrangement of the two aromatic rings, similar to Tröger’s base.^16^

The requirements for new building blocks in supramolecular chemistry include ready access to useful quantities of the compounds. Indeed, the autocondensation proved to be scalable, and there was no need for special equipment. Simply heating 1 g of aminophenones (∼5.0 mmol) in a 4 mL closed vial in the presence of a drop of TMG (20 mol %) cleanly furnished the respective products **2b**, **2c**, **2e**, **2h**, **2k**, **2l**, and **2n** without any erosion in yield in comparison to a 0.5 mmol scale. The challenging CF_2_H-substituted compound also afforded derivative **2o** in a high 70% yield, emphasizing the practical aspect of the developed method.

**Scheme 3:**
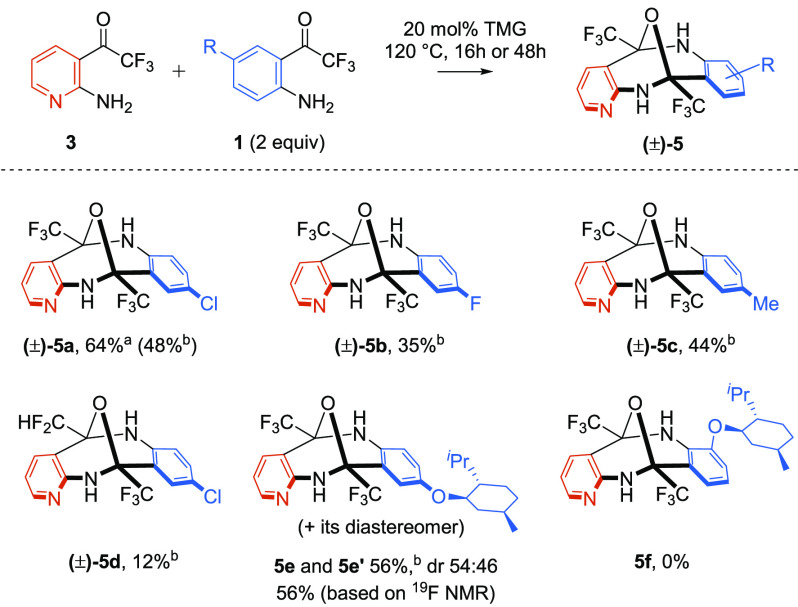
TMG-Catalyzed Solvent-Free Cross-condensation of Aminophenones For 48 h at 120 °C. For 16 h at 120 °C.

With excellent results in the autocondensation process, further investigations were directed to cross-condensation. A careful choice of aminophenones prompted by the different rates of autocondensation and a disparate polarity under chromatographic conditions enabled the isolation of a series of diazocines **5a–e**. The key for successful cross-condensation was mixing aminopyridine **3** with a 2-fold molar excess of aminophenone **1**.^17^ A chiral aminophenone bearing a *p*-menthyloxy group also afforded diazocine **5e** in 56% yield, though as a mixture of diastereomers in a ratio close to 1:1 (Scheme 3). Unfortunately, the sterically encumbered methyl substituent located in the *ortho* position adjacent to the reactive amino group suppressed cross-condensation (Scheme 3, structure **5f**).

**Scheme 4:**
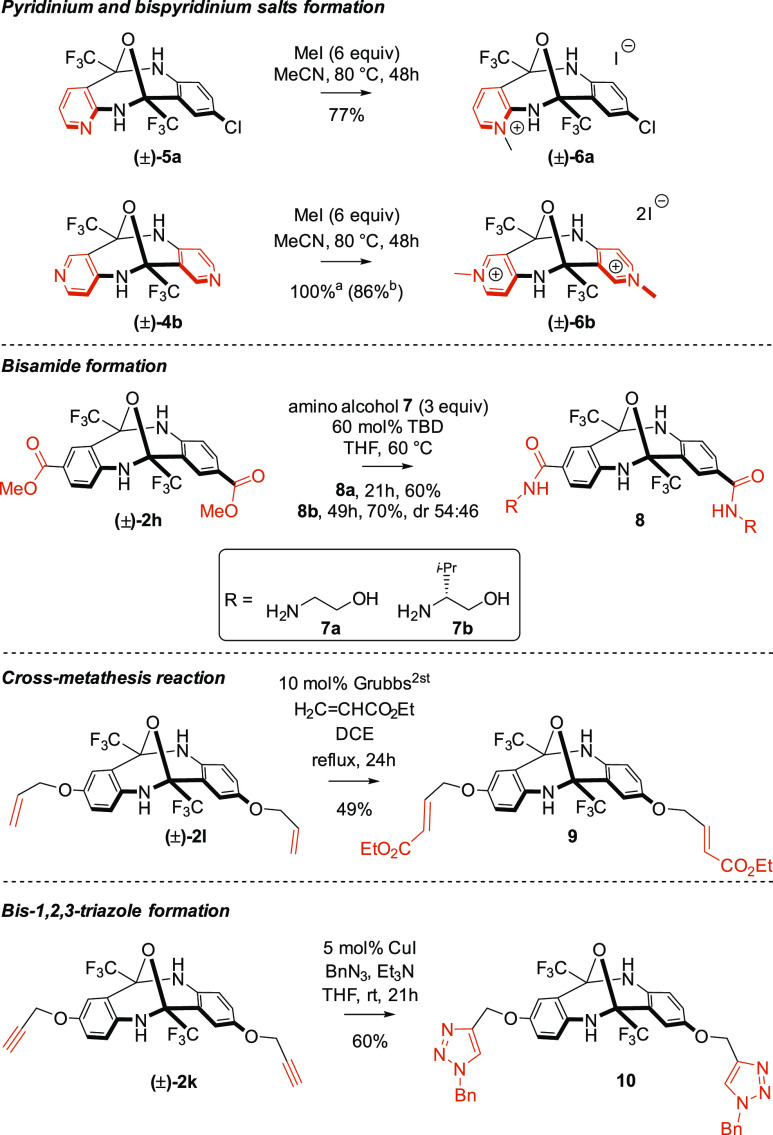
Postformation Modification of the Diazocine Core (TBD = 1,5,7-triazabicyclo[4.4.0]dec-5-ene) Isolated yield after chromatography. Isolated yield after precipitation from the reaction mixture.

The synthetic potential of the diazocine products was demonstrated by a series of well-established transformations proceeding without affecting the diazocine core (Scheme 4). First, we turned our attention to the pyridinium salt structural motif, which proved to be useful in many areas^18^ such as molecular recognition,^19^ catalysis,^20^ and medicinal chemistry.^21^ Gratifyingly, the treatment of diazocines **5a** and **4b** with MeI cleanly afforded salts **6a** and **6b**, respectively, without competitive opening of the oxygen bridge under the action of the strong alkylating agent. Moreover, the bisester function was used to surround the hydrophobic cavity of the V-shaped structure by hydrogen bond donors, useful in molecular recognition. The respective bisester **2h** was easily converted into bisamide **8** using achiral or chiral amino alcohols through a TBD-catalyzed protocol. Finally, carbon–carbon multiple bonds could also play a role in further functionalization without affecting the diazocine scaffold in metal-catalyzed reactions. Thus, bisalkene **2l** underwent a cross-metathesis reaction (CM) with acrylate, whereas bisalkyne **2k** provided bis-1,2,3-triazole **10** in high yield under standard conditions.

**Scheme 5:**
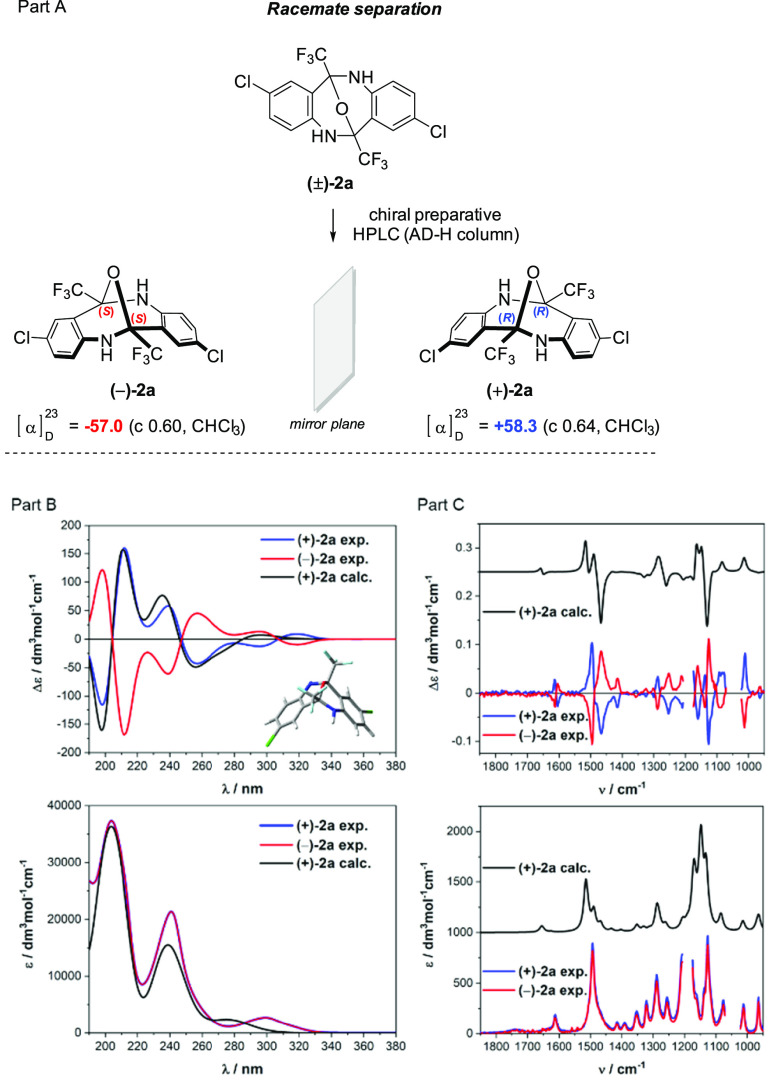
(A) Separation of Racemic (±)-**2a**, (B) Comparison of Experimental and Calculated ECD and UV Data, and (C) VCD and IR Spectra of Enantiomers of **2a** The calculations of the ECD and UV spectra were carried out at the CAM-B3LYP/def2-TZVP/PCM/CH3CN level of theory, while the VCD and IR spectra were calculated at the ωB97X-D/6-311+G(d,p)/ PCM/CH3CN level. More experimental and calculation details are provided in the Supporting Information. The inset in part B shows the geometry of the calculated structure of (+)-**2a**.

With regard to future applications, the most appealing feature of these systems is their stability in the enantiomerically pure form. Indeed, our initial experiments enabled the separation of racemic **2a** into single enantiomers on a preparative scale (Scheme 5). To assign the absolute configuration of the two enantiomers of **2a**, electronic and vibrational circular dichroism (ECD and VCD, respectively) spectra were recorded in acetonitrile and then simulated using quantum chemical methods (DFT and TDDFT). These two chiroptical spectroscopies are very sensitive to any stereochemical changes of the chiral system because they rely on electronic and vibrational transitions spanning the entire UV–vis–mid-IR spectral range. Thus, their complementary combinations provide a holistic view of the properties of any chiral molecules, enabling the conclusive assignment of their absolute configuration in solution and also deeper insight into dynamic stereochemistry.^22^

The ECD and VCD spectra of (+)-**2a** and (−)-**2a** display a perfect mirror-image relationship, confirming the enantiomeric relationship of these two newly synthesized diazocines separated by HPLC, as well as their high optical purity (Scheme 5, part A). The determination of the absolute configuration was based on the comparison of experimental and computed ECD and VCD spectra for an arbitrarily chosen *R*,*R*-enantiomer of **2a**. A conformational search using the MMFF94s force field within 10 kcal/mol followed by DFT geometry optimizations at the ωB97X-D/6-311+G(d,p)/PCM/CH_3_CN level of theory revealed only one stable conformation, indicating *ipso facto* the extremely high rigidity of the diazocine core. Moreover, the high configurational and conformational stability was also proved experimentally using variable-temperature ECD measurements by heating the decalin solutions of **2a** to 180 °C (Figure S2). The very close similarity between experimental and calculated ECD and VCD spectra observed in Scheme 5 (parts B and C) led to the conclusion that the absolute configuration of (+)-**2a** is *R*,*R*, with *S*,*S* for (−)-**2a** (Figure S2). It should be noted that the similar Tröger’s base and its derivatives underwent racemization, especially in the presence of Brønsted of Lewis acids, which makes diazocine **2a** the superior platform for further derivatization.

## Conclusions

In conclusion, we have established a new base-catalyzed, solvent-free condensation of fluorinated *o*-aminophenones for the construction of epoxydibenzo[*b*,*f*][1,5]diazocines. This unprecedented approach offers easily scalable access to a broad range of diazocines bearing a unique V-shaped structure, which was confirmed by X-ray and chiroptical analysis. The rigid molecular architecture allowed the separation of racemic diazocines into single enantiomers and proved their configurational stability by ECD measurements up to 140 °C. The ability to create a hydrophobic cavity by dibezo[*b*,*f*][1,5]diazocines, closely resembling Tröger’s base, opens up a plethora of possible applications in the area of supramolecular chemistry, which is now an ongoing subject in our group.

## Experimental Section

### General Remarks

NMR spectra were recorded in CDCl_3_, DMSO-*d*_6_, or CD_3_OD solutions (unless indicated otherwise); chemical shifts are quoted on the δ scale, with the solvent signal as the internal standard (CDCl_3_, ^1^H NMR δ 7.26, ^13^C NMR δ 77.00; DMSO-*d*_6_, ^1^H NMR δ 2.50, ^13^C NMR δ 39.40; CD_3_OD, ^1^H NMR δ 3.31, ^13^C NMR δ 49.00). High-resolution mass spectra (HRMS) were recorded using an EI technique or electrospray ionization (Supporting Information). Column chromatography was performed on Merck silica gel 60 (230–400 mesh) or alumina oxide 90 active basic (0.063–0.200 mm, Merck) using a standard glass column or a CombiFlash EzPrep system. TLC was performed on aluminum sheets, Merck 60F 254, or aluminum oxide. Optical rotations were recorded on a Jasco P-2000 polarimeter. Melting points were determined on a hot-stage apparatus and are uncorrected. Anhydrous solvents were obtained by distillation over CaH_2_ (DCM) or Na/benzophenone (THF, hexane, and MTBE). Air sensitive reactions were performed in flame-dried glassware under an argon atmosphere. Organic extracts were dried, and solvents were evaporated on a rotary evaporator. Reagents were used as they were purchased unless otherwise indicated. Aminophenones were synthesized starting from *o*-nitroaldehyde by the addition of the CF_3_ anion/reduction of NO_2_/oxidation^23^ sequence (**1b** and **1h**) or orthometalation protocol (**1a**,^24^**1c–g**, **1i–k**, **1o–q**,^25^**1h**,^26^**1s** and **1t**,^24^**1u**,^27^**1x**,^28^ and **3a–c**(25)), according to the literature procedure (for the structure of *o*-aminophenones used in this study, see Figure S1).

### Synthesis of Aminophenones by the Addition/Reduction/Oxidation Sequence

#### General Procedure for the Synthesis of Trifluoroethanol Derivatives via the Addition of Ruppert–Prakash Reagent to Aldehydes (GP1)

To a cooled solution of aldehyde (the temperature of the cooling bath was kept in the range from −20 to −10 °C; the exact temperature is given in each case) in anhydrous THF was added TMSCF_3_ (1.2 equiv). Then a catalytic amount of a solution of TBAF (1 mol %) in THF (1 M) was added dropwise (**Caution!** In some cases, strong exothermic reaction was observed); the cooling bath was removed, and the resulting mixture was stirred for 16 h at rt (TLC analysis usually indicated the presence of silyl ether). Then a solution of TBAF (usually 0.1 mL/mmol of starting aldehyde) and water (usually 0.1 mL/mmol of starting aldehyde) were added, and the mixture was stirred until silyl ether hydrolysis occurred. The reaction mixture was evaporated and redissolved in EtOAc. The solution was washed with water (twice) and brine (twice), dried over Na_2_SO_4_, and evaporated. The residue was chromatographed on silica to give pure trifluoroethanol derivatives.

#### General Procedure for the Oxidation of Trifluoroethanol Derivatives to *o*-Aminophenones (GP2)

To a three-necked round-bottom flask was added anhydrous toluene followed sequentially by CuCl (5 mol %) and 1,10-phenanthroline (5 mol %). The black complex was immediately formed, and the resulting suspension was stirred at rt for 10 min. Then diethyl hydrazinodicarboxylate (DEAD-H_2_, 495.6 mg, 1.08 mmol) was added followed by solid K_2_CO_3_ (2.0 equiv), and the mixture was stirred for an additional 5 min. Then alcohol **12** (2.78 g, 11.3 mmol) was added (as a solid in one portion), and the solution was heated at 90 °C (temperature of the oil bath) for 1 h. To secure the maximum conversion, O_2_ was bubbled through the solution for 1 h (**Caution!** Special care should be taken due to low flash point of toluene, 4.4 °C). Then the reaction mixture was allowed to cool to rt and filtered through a pad of Celite. The filtrate was concentrated *in vacuo* and chromatographed on silica to give *o*-aminophenones (in some cases, fluorinated ketones were further purified by crystallization).

##### 2,2,2-Trifluoro-1-[2-nitro-5-(prop-2-en-1-yloxy)phenyl]ethanol (**11**)

The title compound was obtained according to GP1 using 2-nitro-5-(prop-2-enyl-1-oxy)benzaldehyde^29^ (4.76 g, 23.0 mmol), anhydrous THF (50 mL), TMSCF_3_ (4.1 mL, 27.6 mmol, 1.2 equiv), and TBAF (230 μL, 0.23 mmol, 1 mol %, 1 M in THF). TBAF was added at −10 °C, and the reaction mixture was stirred for 3 h at rt. Then TBAF (2 mL) and water (2 mL) were added. After 16 h, THF was evaporated and the residue was dissolved in EtOAc (50 mL), washed with water (2 × 30 mL) and brine (2 × 30 mL), dried over Na_2_SO_4_, and evaporated. The residue was chromatographed on silica (10–15% EtOAc/hexanes) to give an orange oil (5.93 g, 92%): ^1^H NMR (200 MHz, CDCl_3_) δ 8.10 (d, *J* = 9.2 Hz, 1H), 7.46–7.39 (m, 1H), 6.99 (dd, *J* = 9.2, 2.8 Hz, 1H), 6.33 (q, *J* = 6.1 Hz, 1H), 6.15–5.90 (m, 1H), 5.54–5.25 (m, 2H), 4.71–4.60 (m, 2H), 3.34 (br s, 1H, OH); ^13^C{^1^H} NMR (50 MHz, CDCl_3_) δ 162.6, 141.1, 132.1 (q, *J*_CF_ = 1.0 Hz), 131.6, 127.9, 123.8 (q, *J*_CF_ = 281.2 Hz), 118.9, 115.5, 115.1, 69.5, 66.8 (q, *J*_CF_ = 32.2 Hz); ^19^F NMR (376 MHz, CDCl_3_) δ −77.3; HRMS calcd for C_11_H_9_F_3_NO_4_ (ESI) *m*/*z* [M – H]^−^ 276.0484, found 276.0482.

##### 1-[2-Amino-5-(prop-2-en-1-yloxy)phenyl]-2,2,2-trifluoroethanol (**12**)

To a solution of nitroalcohol **11** (5.75 g, 20.7 mmol) in THF (80 mL) was added a saturated solution of NH_4_Cl (80 mL). The resulting biphasic mixture was cooled to 0 °C, and zinc powder (8.14 g, 124.4 mmol, 6.0 equiv, Sigma-Aldrich, 10 μm) was added in a few portions. Then the reaction mixture was vigorously stirred at rt for 24 h, and the resulting suspension was filtered (washing with 1 × 50 mL of EtOAc). Then the aqueous phase was separated, saturated with solid NaCl, and extracted with EtOAc (4 × 50 mL). The combined organic extracts were dried over Na_2_SO_4_ and evaporated, and the residue was chromatographed on silica (10–25% EtOAc/hexanes) to give a pure aniline **12** as a light yellow solid (2.62 g). Fractions containing impurities were collected and crystallized from *n*-heptane to give additional (0.60 g) white solid (3.37 g, overall yield of 66%): mp 111–112 °C (DCM/*n*-heptane); ^1^H NMR (400 MHz, CDCl_3_) δ 6.81 (br s, 3H), 6.09–5.96 (m, 1H), 5.43–5.35 (m, 1H), 5.31–5.25 (m, 1H), 5.00 (q, *J* = 7.5 Hz, 1H), 4.50–4.45 (m, 2H), 4.34 (br s, 2H); ^13^C{^1^H} NMR (100 MHz, CDCl_3_) δ 153.5, 136.8, 133.4, 125.3 (q, *J*_CF_ = 281.2 Hz), 123.5, 122.2, 117.8, 116.7, 116.1, 72.4 (q, *J*_CF_ = 32.0 Hz), 69.5; ^19^F NMR (376 MHz, CDCl_3_) δ −77.6; HRMS (ESI) *m*/*z* calcd for C_11_H_13_F_3_NO_2_ [M + H]^+^ 248.0898, found 248.0895.

##### 1-[2-Amino-5-(prop-2-en-1-yloxy)phenyl]-2,2,2-trifluoroethanone (**1l**)

The title compound was obtained according to GP2 using toluene (56 mL), CuCl (55.7 mg, 0.56 mmol, 5 mol %), 1,10-phenanthroline (111.5 mg, 0.56 mmol, 5 mol %), DEAD-H_2_ (495.6 mg, 1.08 mmol), K_2_CO_3_ (2.81 g, 22.5 mmol, 2.0 equiv), and alcohol **12** (2.78 g, 11.3 mmol). Then the reaction mixture was allowed to cool to rt and filtered through a pad of Celite (washing with toluene). The filtrate was concentrated *in vacuo* and chromatographed on silica (20–50% DCM/hexanes) to give an orange solid (2.42 g, 88%): mp 76–77 °C (DCM/*n*-heptane); ^1^H NMR (400 MHz, CDCl_3_) δ 7.23–7.19 (m, 1H), 7.12 (dd, *J* = 9.1, 2.8 Hz, 1H), 6.73 (d, *J* = 9.1, 1H), 6.35 (br s, 2H, N*H*_2_), 6.09–5.97 (m, 1H, C*H*=CH_2_), 5.45–5.37 (m, 1H, CH=C*H*_2_), 5.33–5.27 (m, 1H, CH=C*H*_2_), 4.52–4.44 (m, 2H, OC*H*_2_); ^13^C{^1^H} NMR (50 MHz, CDCl_3_) δ 180.2 (q, *J*_CF_ = 33.0 Hz), 149.0, 148.9, 133.2, 128.2, 119.0, 118.2, 117.2 (q, *J*_CF_ = 289.9 Hz), 113.2 (q, *J*_CF_ = 4.2 Hz), 110.4, 69.9; ^19^F NMR (376 MHz, CDCl_3_) δ −69.9; HRMS (ESI) *m*/*z* calcd for C_11_H_11_F_3_NO_2_ [M + H]^+^ 246.0742, found 246.0736.

##### 5-(But-3-en-1-yloxy)-2-nitrobenzaldehyde (**13**)

To the solution of 5-hydroxy-2-nitrobenzaldehyde (4.25 g, 25.4 mmol) in anhydrous THF (230 mL), precooled to −10 °C, were added PPh_3_ (8.00 g, 30.5 mmol) and 3-buten-1-ol (2.55 mL, 30.5 mmol) in one portion. Then diisopropyl azodicarboxylate (6.0 mL, 30.52 mmol) was added dropwise over 5 min; the cooling bath was removed, and stirring was continued at rt for 22 h. The resulting solution was concentrated *in vacuo*, and the residue was chromatographed on silica (15–30% EtOAc/hexanes, Combi Flash) to give a yellowish oil (3.16 g, 56%): ^1^H NMR (400 MHz, CDCl_3_) δ 10.48 (s, 1H, C*H*O), 8.15 (d, *J* = 9.0 Hz, 1H), 7.32 (d, *J* = 2.8 Hz, 1H), 7.14 (dd, *J* = 9.0, 2.9 Hz, 1H), 5.93–5.81 (m, 1H, C*H*=CH_2_), 5.23–5.12 (m, 2H, CH=C*H*_2_), 4.16 (t, *J* = 6.6 Hz, 2H, OC*H*_2_), 2.63–2.55 (m, 2H, C*H*_2_CH=C*H*_2_); ^13^C{^1^H} NMR (100 MHz, CDCl_3_) δ 188.6, 163.5, 142.3, 134.5, 133.4, 127.3, 119.0, 118.0, 113.9, 68.6, 33.3; HRMS (EI) *m*/*z* calcd for C_11_H_11_NO_4_ [M]^•+^ 221.0688, found 221.0694.

##### 1-[5-(But-3-en-1-yloxy)-2-nitrophenyl]-2,2,2-trifluoroethanol (**14**)

The title compound was obtained according to GP1 using aldehyde **13** (3.13 g, 15.2 mmol), anhydrous THF (50 mL), TMSCF_3_ (2.5 mL, 17.0 mmol, 1.2 equiv), and TBAF (150 μL, 0.15 mmol, 1 mol %, 1 M in THF). The TBAF solution was added at −10 °C, and the reaction mixture was stirred for 3 h at rt. Then TBAF (1.5 mL, 1 M in THF) and water (1.5 mL) were added to deprotect silyl ether. After 16 h, THF was evaporated and the residue was dissolved in EtOAc (50 mL), washed with water (2 × 30 mL) and brine (2 × 30 mL), dried over Na_2_SO_4_, and evaporated. The residue was chromatographed on silica (10–15% EtOAc/hexanes) to give an orange oil (2.79 g, 63%): ^1^H NMR (400 MHz, CDCl_3_) δ 8.10 (d, *J* = 9.2 Hz, 1H), 7.40 (d, *J* = 2.8 Hz, 1H), 6.97 (dd, *J* = 9.2, 2.8 Hz, 1H), 6.36–6.28 (m, 1H), 5.95–5.82 (m, 1H), 5.23–5.11 (m, 2H), 4.13 (t, *J* = 6.6 Hz, 2H), 3.26 (d, *J* = 5.5 Hz, 1H), 2.62–2.54 (m, 2H); ^13^C{^1^H} NMR (100 MHz, CDCl_3_) δ 163.1, 141.1, 133.5, 132.1, 127.9, 123.0 (q, *J* = 281.4 Hz), 117.7, 115.2, 114.9, 68.1, 67.0 (q, *J* = 32.4 Hz), 33.2; ^19^F NMR (376 MHz, CDCl_3_) δ −77.3; HRMS (ESI) *m*/*z* calcd for C_12_H_11_F_3_NO_4_ [M – H]^−^ 290.0640, found 290.0631.

##### 1-[2-Amino-5-(but-3-en-1-yloxy)phenyl]-2,2,2-trifluoroethanol (**15**)

To a solution of nitroalcohol **14** (2.73 g, 9.37 mmol) in THF (20 mL) was added a saturated solution of NH_4_Cl (20 mL); the mixture was cooled to 0 °C, and zinc powder (3.67 g, 56.2 mmol, 6.0 equiv, Sigma-Aldrich, 10 μm) was added in a few portions. Then the reaction mixture was vigorously stirred at rt for 16 h and filtered through pad of Celite (washing with EtOAc). The resulting mixture was diluted with brine (30 mL) and extracted with EtOAc (3 × 20 mL). The combined organic extracts were washed with brine (2 × 20 mL), dried over Na_2_SO_4_, and evaporated. The residue was chromatographed on silica (15–50% EtOAc/hexanes, Combi Flash, 80 g column) to give a light yellow solid (1.92 g, 78%): ^1^H NMR (400 MHz, CDCl_3_) δ 6.81–6.72 (m, 3H), 5.95–5.82 (m, 1H), 5.20–5.07 (m, 2H), 4.97 (q, *J* = 7.5 Hz, 1H), 4.26 (s, 3H), 3.95 (t, *J* = 6.7 Hz, 2H), 2.56–2.46 (m, 2H); ^13^C{^1^H} NMR (100 MHz, CDCl_3_) δ 153.8, 136.2, 134.4, 125.1 (q, *J*_CF_ = 283.2 Hz), 123.6 (q, *J*_CF_ = 1.4 Hz), 122.4, 117.0, 116.4, 115.9, 72.4 (q, *J*_CF_ = 32.1 Hz), 67.8, 33.6; ^19^F NMR (376 MHz, CDCl_3_) δ −77.6; HRMS (ESI) *m*/*z* calcd for C_12_H_14_F_3_NO_2_ [M + H]^+^ 262.1055, found 262.1042.

##### 1-[2-Amino-5-(but-3-en-1-yloxy)phenyl]-2,2,2-trifluoroethanol (**1m**)

The title compound was obtained according to GP2 using anhydrous toluene (40 mL), CuCl (36.2 mg, 0.366 mmol, 5 mol %), 1,10-phenanthroline (72.6 mg, 0.366 mmol, 5 mol %), DEAD-H_2_ (322.0 mg, 1.83 mmol), solid K_2_CO_3_ (2.02 g, 14.62 mmol, 2.0 equiv), and alcohol **15** (1.91 g, 7.31 mmol). Then the reaction mixture was allowed to cool to rt, filtered through a pad of Celite, and washed with EtOAc. The filtrate was concentrated *in vacuo* and chromatographed on silica (10% EtOAc/hexanes, CombiFlash) to give an orange solid (1.62 g, 86%): mp 71–72 °C (DCM/*n*-heptane); ^1^H NMR (400 MHz, CDCl_3_) δ 7.20–7.15 (m, 1H), 7.10 (dd, *J* = 9.1, 2.8 Hz, 1H), 6.68 (d, *J* = 9.1 Hz, 1H), 6.23 (br s, 2H), 5.96–5.83 (m, 1H), 5.22–5.08 (m, 2H), 3.96 (t, *J* = 6.6 Hz, 2H), 2.56–2.48 (m, 2H); ^13^C{^1^H} NMR (100 MHz, CDCl_3_) δ 180.1 (q, *J*_CF_ = 33.1 Hz), 149.3, 148.7, 134.3, 128.0, 118.9, 117.1 (d, *J*_CF_ = 291.4 Hz), 117.1, 112.9 (q, *J*_CF_ = 4.3 Hz), 110.5, 68.2, 33.6; ^19^F NMR (376 MHz, CDCl_3_) δ −69.9; HRMS (ESI) *m*/*z* calcd for C_12_H_13_F_3_NO_2_ [M + H]^+^ 260.0898, found 260.0892.

##### 2-Nitro-5-(pent-4-en-1-yloxy)benzaldehyde (**16**)

To a solution of 5-hydroxy-2-nitrobenzaldehyde (6.0 g, 35.9 mmol) and Ph_3_P (11.30 g, 43.1 mmol) in THF (200 mL), cooled to −20 °C, was added 4-penten-1-ol (4.42 mL, 43.1 mmol) in one portion. Then diisopropyl azodicarboxylate (6.0 mL, 30.5 mmol) was added dropwise over 10 min. Stirring was continued for 1 h at −20 °C; the cooling bath was removed, and the resulting solution was stirred at rt for an additional 19 h. Then the reaction mixture was concentrated *in vacuo*, and the residue was chromatographed on silica (5–10% EtOAc/hexanes, Combi Flash) to give a yellowish oil (4.67 g, 54%): ^1^H NMR (400 MHz, CDCl_3_) δ 10.47–10.46 (m, 1H, C*H*O), 8.13 (dd, *J* = 9.1, 0.9 Hz, 1H), 7.30 (dd, *J* = 2.8, 0.8 Hz, 1H), 7.13 (dd, *J* = 9.0, 2.9 Hz, 1H), 5.89–5.77 (m, 1H, C*H*=CH_2_), 5.10–4.99 (m, 2H, CH=C*H*_2_), 4.11 (t, *J* = 6.4 Hz, 2H, OC*H*_2_), 2.28–2.21 (m, 2H, C*H*_2_CH=CH_2_), 1.97–1.89 (m, 2H, CH_2_C*H*_2_CH_2_); ^13^C{^1^H} NMR (100 MHz, CDCl_3_) δ 188.7, 163.7, 142.2, 137.2, 134.5, 127.4, 119.0, 115.9, 113.9, 68.6, 29.9, 28.1; HRMS (ESI) *m*/*z* calcd for C_12_H_12_NO_4_ [M – H]^−^ 234.0766, found 234.0763.

##### 2,2,2-Trifluoro-1-[2-nitro-5-(pent-4-en-1-yloxy)phenyl]ethanol (**17**)

The title compound was obtained according to GP1 using aldehyde **16** (4.52 g, 19.2 mmol), anhydrous THF (50 mL), TMSCF_3_ (3.4 mL, 23.1 mmol, 1.2 equiv), and TBAF (192 μL, 0.19 mmol, 1 mol %, 1 M in THF). TBAF was added at −10 °C, and the reaction mixture was stirred for 3 h at rt (TLC analysis indicated the absence of substrate). Then a TBAF solution (1.5 mL, 1 M in THF) and water (1.5 mL) were added to deprotect silyl ether. After 16 h, the reaction mixture was evaporated and the residue was dissolved in EtOAc (50 mL), washed with water (2 × 30 mL) and brine (2 × 30 mL), dried over Na_2_SO_4_, and evaporated. The residue was used in the next step without further purification.

##### 1-[2-Amino-5-(pent-4-en-1-yloxy)phenyl]-2,2,2-trifluoroethanol (**18**)

To a solution of nitroalcohol **17** (5.86 g, 19.2 mmol) in THF (40 mL) was added a saturated solution of NH_4_Cl (40 mL); the mixture was cooled to 0 °C, and zinc powder (7.53 g, 115.2 mmol, 6.0 equiv, Sigma-Aldrich, 10 μm) was added in a few portions. Then the reaction mixture was stirred at rt for 16 h and filtered through a pad of Celite (washing with EtOAc). The resulting mixture was diluted with brine (30 mL) and extracted with EtOAc (3 × 20 mL). The combined organic extracts were washed with brine (2 × 20 mL), dried over Na_2_SO_4_, and evaporated. The residue was chromatographed on silica (10–25% EtOAc/hexanes) to give a light yellow solid (4.16 g). Fractions including some impurities were collected and crystallized from *n*-heptane to obtain additional (0.41 g) aniline **18** (4.57 g, overall yield of 86%): mp >87 °C dec (precipitation from a DCM solution with *n*-heptane); ^1^H NMR (400 MHz, CDCl_3_) δ 6.82–6.75 (m, 3H), 5.91–5.79 (m, 1H, C*H*=CH_2_), 5.10–4.95 (m, 2H, HOC*H*CF_3_, CH=C*H*_2_), 4.22 (br s, 3H, NH_2_ and OH), 3.92 (t, *J* = 6.4 Hz, 2H, OC*H*_2_), 2.27–2.18 (m, 2H, C*H*_2_CH=CH_2_), 1.90–1.81 (m, 2H, CH_2_C*H*_2_CH_2_); ^13^C{^1^H} NMR (100 MHz, CDCl_3_) δ 154.1, 137.9, 136.4, 125.5 (*J*_CF_ = 281.5 Hz), 123.7, 122.5, 116.5, 115.9, 115.3, 72.5 (*J*_CF_ = 32.0 Hz), 67.9, 30.2, 28.6; ^19^F NMR (376 MHz, CDCl_3_) δ −77.6; HRMS (ESI) *m*/*z* calcd for C_13_H_17_F_3_NO_2_ [M + H]^+^ 276.1211, found 276.1203.

##### 1-[2-Amino-5-(pent-4-en-1-yloxy)phenyl]-2,2,2-trifluoroethanone (**1n**)

The title compound was obtained according to GP2 using anhydrous toluene (82 mL), CuCl (81.4 mg, 0.82 mmol, 5 mol %), 1,10-phenanthroline (162.9 mg, 0.82 mmol, 5 mol %), DEAD-H_2_ (724.0 mg, 4.11 mmol), solid K_2_CO_3_ (4.54 g, 32.9 mmol, 2.0 equiv), and alcohol **18** (4.53 g, 16.4 mmol). Then the reaction mixture was allowed to cool to rt and filtered through a pad of Celite (washing with toluene). The filtrate was concentrated *in vacuo* and chromatographed on silica (25–35% DCM/hexanes) to give an orange solid (4.06 g, 90%): mp 78–79 °C (DCM/*n*-heptane); ^1^H NMR (400 MHz, CDCl_3_) δ 7.16 (br s, 1H), 7.13–7.06 (m, 1H), 6.68 (d, *J* = 9.1 Hz, 1H), 6.23 (br s 2H, N*H*_2_), 5.92–5.78 (m, 1H, CH=C*H*_2_), 5.11–4.97 (m, 2H, C*H*=CH_2_), 3.92 (t, *J* = 6.4 Hz, 2H, OC*H*_2_), 2.28–2.19 (m, 2H, C*H*_2_CH=CH_2_), 1.92–1.82 (m, 2H, CH_2_C*H*_2_CH_2_); ^13^C{^1^H} NMR (100 MHz, CDCl_3_) δ 180.3 (*J*_CF_ = 32.8 Hz), 149.6, 148.7, 137.8, 128.1, 119.0, 117.2 (*J*_CF_ = 289.8 Hz), 115.4, 112.9 (*J*_CF_ = 4.8 Hz), 110.7, 68.2, 30.2, 28.5; ^19^F NMR (376 MHz, CDCl_3_) δ −69.8; HRMS (ESI) *m*/*z* calcd for C_13_H_15_F_3_NO_2_ [M + H]^+^ 274.1055, found 274.1046.

##### 2-Nitro-5-(prop-2-yn-1-yloxy)benzaldehyde (**19**)

To a solution of 5-hydroxy-2-nitrobenzaldehyde (8.0 g, 47.9 mmol) in DMF (75 mL) were added K_2_CO_3_ (7.28 g, 52.6 mmol), 3-bromo-1-propyne (4.3 mL, 57.4 mmol), and TBAB (154.3 mg, 0.48 mmol, 1 mol %). The resulting suspension was stirred for 22 h, and the solvent was evaporated. The residue was partitioned between a saturated aqueous solution of Na_2_CO_3_ (50 mL) and EtOAc (50 mL). The aqueous phases was separated and extracted with EtOAc (4 × 50 mL). The combined organic phases were dried over solid K_2_CO_3_ and evaporated. The residue was chromatographed on silica (25% DCM/hexanes) to give a yellow oil (7.29 g, 74%): ^1^H NMR (400 MHz, CDCl_3_) δ 10.46 (s, 1H, C*H*O), 8.16 (d, *J* = 9.0 Hz, 1H), 7.41 (d, *J* = 3.0 Hz, 1H), 7.24 (dd, *J* = 9.0, 2.9 Hz, 1H), 4.84 (d, *J* = 2.4 Hz, 2H, CH_2_), 2.59 (t, *J* = 2.4 Hz. 1H). Spectral data are in agreement with those reported previously.^30^

##### 2,2,2-Trifluoro-1-[2-nitro-5-(prop-2-yn-1-yloxy)phenyl]ethanol (**20**)

The title compound was obtained according to GP1 using aldehyde **19** (7.26 g, 35.4 mmol), anhydrous THF (70 mL), TMSCF_3_ (6.8 mL, 46.04 mmol, 1.3 equiv), and TBAF (354 μL, 0.35 mmol, 1 mol %, 1 M in THF). A TBAF solution was added at −10 °C over 15 min (**Caution!** A strong exothermic effect was observed), and the reaction mixture was stirred for 17.5 h at rt. Then a TBAF solution (2 mL, 1 M in THF) and water (2 mL) were added. After 4 h, the reaction was quenched with a saturated aqueous solution of NH_4_Cl (50 mL). The aqueous phase was separated and extracted with EtOAc (3 × 50 mL). The combined extracts were washed with brine (1 × 100 mL), dried over Na_2_SO_4_, and evaporated. The residue was chromatographed on silica (50–75% DCM/hexanes) to give **20** as a white solid (3.51 g, 36%): mp 78–79 °C (DCM/*n*-heptane); ^1^H NMR (400 MHz, CD_3_OD) δ 8.09 (d, *J* = 9.2 Hz, 1H), 7.54 (d, *J* = 2.8 Hz, 1H), 7.17 (dd, *J* = 9.3, 2.9 Hz, 1H), 4.89 (d, *J* = 2.5 Hz, 2H, C*H*_2_), 3.02 (t, *J* = 2.5 Hz, C≡C*H*); ^13^C{^1^H} NMR (100 MHz, CD_3_OD) δ 162.7, 143.3, 134.5, 128.4, 125.8 (q, *J*_CF_ = 280.7 Hz), 116.8, 116.4, 78.4, 77.9, 69.0 (q, *J*_CF_ = 31.8 Hz), 57.3; ^19^F NMR (376 MHz, CD_3_OD) δ −79.1; HRMS (ESI) *m*/*z* calcd for C_11_H_8_F_3_NO_4_ [M – H]^−^ 274.0327, found 274.0322.

##### 1-[2-Amino-5-(prop-2-yn-1-yloxy)phenyl]-2,2,2-trifluoroethanol (**21**)

To a solution of nitroalcohol **20** (3.23 g, 11.7 mmol) in THF (50 mL) was added a saturated solution of NH_4_Cl (50 mL), and then zinc powder (4.61 g, 70.4 mmol, 6.0 equiv, Sigma-Aldrich, 10 μm) was added in a few portions (a slight increase in the temperature of the reaction mixture was detected). After 18 h, the reaction mixture was filtered through a pad of Celite, and the aqueous phase was separated, saturated with solid NaCl, and extracted with EtOAc (4 × 50 mL). The combined organic extracts were dried over Na_2_SO_4_ and evaporated. The residue was filtered through a pad of silica (25% EtOAc/hexanes) and then crystallized form *n*-heptane to give **21** as a light yellow solid (1.29 g, 45%): mp 82–83 °C (*n*-heptane); ^1^H NMR (400 MHz, CDCl_3_) δ 6.89 (br s, 3H), 5.09–5.01 (m, 1H, C*H*OH), 4.81 (br s, 3H, N*H*_2_ and O*H*), 4.64 (d, *J* = 2.4 Hz, 2H, C*H*_2_), 2.51 (t, *J* = 2.4 Hz, 1H, C≡C*H*); ^13^C{^1^H} NMR (100 MHz, CDCl_3_) δ 152.2, 137.5, 125.2 (*J*_CF_ = 281.3 Hz), 123.1, 121.8, 117.1, 116.6, 78.7, 75.8, 72.2 (*J*_CF_ = 32.1 Hz), 56.7; ^19^F NMR (376 MHz, CDCl_3_) δ −77.6; HRMS (ESI) *m*/*z* calcd for C_11_H_10_F_3_NO_2_ [M + H]^+^ 246.0742, found 246.0732.

##### 1-[2-Amino-5-(prop-2-yn-1-yloxy)phenyl]-2,2,2-trifluoroethanone (**1k**)

The title compound was obtained according to modified GP2 using anhydrous toluene (22 mL), CuCl (21.3 mg, 0.216 mmol, 5 mol %), 1,10-phenanthroline (42.7 mg, 0.216 mmol, 5 mol %), DEAD-H_2_ (189.8 mg, 1.08 mmol), and solid K_2_CO_3_ (2.19 g, 8.62 mmol, 2.0 equiv). Then alcohol **21** (1.06 g, 4.31 mmol) was added (as a solid in one portion), and the solution was heated at 90 °C (temperature of the oil bath) for 40 h. To secure the maximum conversion, O_2_ was slowly bubbled through the solution for 40 h. Then the reaction mixture was allowed to cool to rt and filtered through a pad of Celite (washing with toluene). The filtrate was concentrated *in vacuo*, and the residue was chromatographed on silica (15% DCM/hexanes) to give an orange solid (348.7 mg, 33%): mp 56–58 °C (DCM/*n*-heptane); ^1^H NMR (400 MHz, CDCl_3_) δ 7.37–7.32 (m, 1H), 7.16 (dd, *J* = 9.1, 2.8 Hz, 1H), 6.75–6.67 (m, 1H), 6.29 (br s, 2H, N*H*_2_), 4.64 (d, *J* = 2.4 Hz, 2H, CH_2_), 2.53 (t, 2.4 Hz, 1H, C≡C*H*); ^13^C{^1^H} NMR (100 MHz, CDCl_3_) δ 180.3 (q, *J*_CF_ = 33.2 Hz), 149.3, 147.9, 128.3, 119.0, 117.2 (q, *J*_CF_ = 289.9 Hz), 114.6 (q, *J*_CF_ = 4.2 Hz), 110.5, 78.4, 76.1, 57.2; ^19^F NMR (376 MHz, CDCl_3_) δ −69.9; HRMS (ESI) *m*/*z* calcd for C_11_H_9_F_3_NO_2_ [M + H]^+^ 244.0585, found 244.0574.

##### 2-{[(1*R*,2*S*,5*R*)-5-Methyl-2-(propan-2-yl)cyclohexyl]oxy}aniline (**23**)

To a solution of (1*R*,2*S*,5*R*)-5-methyl-2-(propan-2-yl)cyclohexyl 2-nitrophenyl ether^31^**22** (5.65 g, 20.4 mmol) in EtOAc (100 mL) was added 10% Pd/C (1.05 g, 1.0 mmol, 5 mol %), and the resulting suspension was shaken in a Parr apparatus under an atmosphere of H_2_ (3 bar; **Caution!** Exothermic reaction!). After 3 h, the reduction was complete (as judged by TLC), and a gentle stream of argon was passed through the solution for 5 min. The resulting suspension was filtered through a pad of Celite, and the solvent was evaporated to give a colorless oil (5.05 g). Crude 2-{[(1*R*,2*S*,5*R*)-5-methyl-2-(propan-2-yl)cyclohexyl]oxy}aniline **23** was used in the next step without further purification.

##### 2,2-Dimethyl-*N*-(2-{[(1*R*,2*S*,5*R*)-5-methyl-2-(propan-2-yl)cyclohexyl]oxy}phenyl)propanamide (**24**)

To a solution of aniline **23** (5.05 g, 20.4 mmol) and Et_3_N (3.3 mL, 24.6 mmol, 1.2 equiv) in anhydrous DCM (100 mL), cooled to 0 °C, was added dropwise PivCl (2.8 mL, 22.5 mmol, 1.1. equiv); the cooling bath was removed, and the reaction mixture was stirred at rt. After 16 h, the reaction mixture was washed with 10% aqueous citric acid (2 × 30 mL) and a saturated solution of Na_2_CO_3_ (2 × 30 mL), dried over MgSO_4_, and evaporated. The residue was chromatographed on silica (5% EtOAc/hexanes) to give a colorless oil (5.47 g, 91%): [α]_D_^23^ = −91.0 (CHCl_3_, *c* = 1.0); ^1^H NMR (400 MHz, CDCl_3_) δ 8.41 (dd, *J* = 7.9, 1.7 Hz, 1H), 8.26 (br s, 1H), 7.02–6.85 (m, 3H), 4.18–4.08 (m, 1H), 2.27–2.12 (m, 2H), 1.83–1.70 (m, 2H), 1.57–1.40 (m, 2H), 1.38–1.09 (m, 2H) overlapping 1.31 (s, 9H), 1.02–0.88 (m, 1H) overlapping 0.95 (d, *J* = 7.1 Hz, 3H) and 0.92 (d, *J* = 6.6 Hz, 3H), 0.80 (d, *J* = 6.9 Hz, 3H); ^13^C{^1^H} NMR (100 MHz, CDCl_3_) δ 176.2, 146.4, 129.0, 123.2, 120.8, 119.6, 111.9, 78.4, 48.6, 40.6, 40.0, 34.4, 31.4, 27.6, 26.5, 23.8, 22.0, 20.7, 16.9; HRMS (ESI) *m*/*z* calcd for C_21_H_33_N_2_ONa [M + H]^+^ 354.2409, found 354.2403.

##### 1-(2-Amino-3-{[(1*R*,2*S*,5*R*)-5-methyl-2-(propan-2-yl)cyclohexyl]oxyphenyl)-2,2,2-trifluoroethanone (**1r**)

To a solution of 2,2-dimethyl-*N*-(2-{[(1*R*,2*S*,5*R*)-5-methyl-2-(propan-2-yl)cyclohexyl]oxy}phenyl)propanamide (**24**) (5.60 g, 17 mmol) and TMEDA (5.14 mL, 37.4 mmol, 2.2 equiv) in anhydrous THF (40 mL) was added dropwise *n*-BuLi (16.4 mL, 37.4 mmol, 2.2 equiv, 2.28 M in hexane) with a syringe pump within 40 min. Then the cooling bath was removed, and the reaction mixture was stirred for 4 h at 20 °C. The resulting yellow suspension was cooled again to −30 °C, and CF_3_CO_2_Et (2.86 mL, 23.8 mmol, 1.4 equiv) was added. Stirring was continued for 1.5 h at rt with 4 M HCl with dioxane (30 mL) and water (3 mL). The biphasic mixture was heated at 90 °C for 4 h, cooled to rt, and neutralized with a saturated solution of Na_2_CO_3_. Then the reaction mixture was extracted with EtOAc (3 × 30 mL), and the combined organic extracts were dried over MgSO_4_ and evaporated. The residue was chromatographed on silica (2.5% EtOAc/hexanes) to give ketone **1r** as a red-brown oil (1.27 g, 22%): [α]_D_^23^ = −83.5 (CHCl_3_, *c* = 0.4); IR (film) 3506, 3371, 2957, 2928, 2871, 1667, 1619, 1580, 1545, 1454 cm^–1^; ^1^H NMR (400 MHz, CDCl_3_) δ 7.37–7.31 (m, 1H), 6.92 (d, *J* = 7.6 Hz, 1H) overlapping 6.85 (br s, 2H, NH_2_), 4.16–4.07 (m, 1H), 2.24–2.10 (m, 2H), 1.82–1.70 (m, 2H), 1.64–1.54 (m, 1H), 1.54–1.40 (m, 1H), 1.38–0.99 (m, 3H), 0.99–0.85 (m, 7H), 0.79 (d, *J* = 6.96 Hz, 3H); ^13^C{^1^H} NMR (100 MHz, CDCl_3_) δ 180.7 (q, *J*_CF_*=* 33.0 Hz), 145.9, 145.2, 121.9 (q, *J*_CF_*=* 4.1 Hz), 117.1 (q, *J*_CF_*=* 289.7 Hz), 115.9, 114.7, 110.6, 78.4, 48.0, 40.0, 34.8, 31.4, 26.3, 23.7, 22.0, 20.7, 16.7; ^19^F NMR (376 MHz, CDCl_3_) δ −69.7; HRMS (ESI) *m*/*z* calcd for C_18_H_24_F_3_N_2_O [M + H]^+^ 344.1837, found 344.1834.

##### 2,2,2-Trifluoro-1-(6-nitro-1,3-benzodioxol-5-yl)ethanol (**25**)

The title compound was obtained according to GP1 using 6-nitro-1,3-benzodioxole-5-carbaldehyde (**26**) (2.0 g, 10.3 mmol), anhydrous THF (20 mL), TMSCF_3_ (2.0 mL, 13.3 mmol, 1.3 equiv), and TBAF (0.1 mL, 0.1 mmol, 1 mol %). A TBAF solution was added at −20 °C, and the reaction mixture was stirred for 4 h at rt. Then the reaction mixture was cooled to 0 °C, and water (1 mL) and a TBAF solution (1 mL, 1 M in THF) were added. After 1 h, the reaction mixture was evaporated, and the residue was dissolved in MTBE (20 mL), washed with water (2 × 20 mL) and brine (2 × 20 mL), dried over Na_2_SO_4_, and evaporated. The residue was chromatographed on silica (15% EtOAc/hexanes) to give a yellow oil (2.71 g, 99%): ^1^H NMR (400 MHz, CDCl_3_) δ 7.52 (s, 1H), 7.33 (s, 1H), 6.20 (q, *J* = 6.7 Hz, 2H), 6.17–6.14 (m, 2H), 3.20 (br s, 1H, OH); ^19^F NMR (376 MHz, CDCl_3_) δ −77.5. Spectroscopic data are in agreement with those reported previously.^32^

##### 1-(6-Amino-1,3-benzodioxol-5-yl)-2,2,2-trifluoroethanol (**27**)

To a solution of nitroalcohol **25** (2.71 g, 10.2 mmol) in reagent-grade EtOAc (100 mL) was added 10% Pd/C (543.9 mg, 0.51 mmol, 5 mol %), and the resulting mixture was shaken in a Parr apparatus under an atmosphere of H_2_ for 2 h (3 bar; **Caution!** In some cases, reduction of the nitro group has appeared to be strongly exothermic, and caution should be taken; the temperature of the reaction mixture increased from 17 to 26 °C within 10 min). Then a gentle stream of argon was bubbled through the solution for 5 min. The resulting suspension was filtered through a pad of Celite (washing with 50% EtOAc/hexanes), and solvents were evaporated to give a light-yellow solid (2.21 g, 92%). Crude aminoalcohol **28** was used in the next step without further purification.

##### 1-(6-Amino-1,3-benzodioxol-5-yl)-2,2,2-trifluoroethanone (**28**)

The title compound was obtained according to GP2 using anhydrous toluene (50 mL), CuCl (464.8 mg, 4.70 mmol, 0.5 equiv), 1,10-phenanthroline (186.1 mg, 1.03 mmol, 0.11 equiv), DEAD-H_2_ (413.6 mg, 2.35 mmol, 0.25 equiv), solid K_2_CO_3_ (2.59 g, 18.78 mmol, 2.0 equiv), and alcohol **27** (2.21 g, 9.39 mmol). Then the reaction mixture was allowed to cool to rt and filtered through a pad of Celite (washing with toluene). The filtrate was concentrated *in vacuo* and chromatographed on silica (120 g, 10–15% EtOAc/hex) to give an orange solid (1.59 g, 75%): mp 154–155 °C (*n*-heptane); IR (KBr) 3416, 3317, 3087, 3013, 2919, 1663, 1641, 1576 cm^–1^; ^1^H NMR (400 MHz, CDCl_3_) δ 7.07 (q, *J* = 2.1 Hz, 1H), 6.70 (br s, 2H), 6.17 (s, 1H), 5.95 (s, 2H); ^13^C{^1^H} NMR (100 MHz, CDCl_3_) δ 177.9 (q, *J*_CF_ = 32.8 Hz), 155.4, 153.8, 139.6, 117.5 (q, *J*_CF_ = 289.5 Hz), 106.8, 106.8, 103.9, 101.8, 96.2; ^19^F NMR (376 MHz, CDCl_3_) δ −69.7; HRMS (EI) *m*/*z* calcd for C_9_H_6_F_3_NO_3_ [M]^•+^ 233.0300, found 233.0293.

#### General Procedure for the Synthesis of Symmetric Epoxydibenzo[*b*,*f*][1,5]diazocines (GP3)

Fluoromethylketone **1** (*x* mmol) and *N*,*N*,*N′*,*N′*-tetramethylguanidine (TMG, 20 mol %) were placed in a screw-cap 4 mL vial, and the resulting mixture was heated at 120 °C (IKA heating block, temperature of the reference vial filled with silicon oil). Then the reaction mixture was diluted with EtOAc or DCM (10 mL), adsorbed on silica (or aluminum oxide), and chromatographed to give the corresponding dibenzo[*b*,*f*][1,5]diazocines **2**. The analytical sample was crystallized from a given solvent to measure the melting point.

##### (6S*,12S*)-2,8-Dichloro-6,12-bis(trifluoromethyl)-5,6,11,12-tetrahydro-6,12-epoxydibenzo[*b*,*f*][1,5]diazocine (**2a**)

The title compound was obtained according to GP3 using ketone **1a** (1.0 g, 4.47 mmol) and TMG (111 μL, 0.89 mmol, 20 mol %). The crude product was chromatographed on silica (5% EtOAc/hexanes) to give a colorless solid (911.2 mg, 95%). Autocondensation of **1a** was also performed on a 0.5 mmol scale to afford product **2a** in 93% yield (99.8 mg) after chromatography during the optimization studies. It should be mentioned that all attempts to use the Dean–Stark apparatus to continuously remove water formed during the condensation have failed (aminophenone **1a** sublimed in the condenser). Similarly, when an open round-bottom flask was used as the reaction vessel, aminophenone **1a** also easily sublimed: IR (KBr) 3380, 3352, 3070, 3041, 2902, 1776, 1775, 1612, 1493 cm^–1^; ^1^H NMR (400 MHz, CDCl_3_) δ 7.43 (br s, 2H), 7.21 (dd, *J* = 8.6, 2.3 Hz, 2H), 6.80 (d, *J* = 8.6 Hz, 2H), 4.89 (br s, 2H, 2 × N*H*). Spectral data are in agreement with those reported in the literature.^9e^ Representative chromatograms of the formation of diazocine **2a**, catalyzed by TMG, are presented below.

##### (6S*,12S*)-2,8-Dibromo-6,12-bis(trifluoromethyl)-5,6,11,12-tetrahydro-6,12-epoxydibenzo[*b*,*f*][1,5]diazocine (**2b**)

The title compound was obtained according to GP3 (16 h, 120 °C) using ketone **1b** (134.0 mg, 0.5 mmol) and TMG (12.5 μL, 0.1 mmol, 20 mol %). The crude product was chromatographed on silica (10% EtOAc/hexanes) to give a light yellow oil that solidified upon standing in the refrigerator (120.5 mg, 93%). The reaction was also performed on a 3.7 mmol scale (1.0 g of *o*-TMFK **1b**) to give the product in 89% yield (865.3 mg): IR (KBr) 3374, 3336, 1606, 1490 cm^–1^; ^1^H NMR (600 MHz, CD_3_OD) δ 7.51–7.49 (m, 2H), 7.30 (dd, *J* = 8.7, 2.2 Hz, 2H), 6.77 (d, *J* = 8.7 Hz, 2H); ^13^C{^1^H} NMR (150 MHz, CD_3_OD) δ 142.2, 134.1, 128.8 (q, *J*_CF_ = 3.1 Hz), 124.7 (q, *J*_CF_ = 281.5 Hz), 122.8, 120.4, 113.0, 83.9 (q, *J*_CF_ = 32.4 Hz); ^19^F NMR (376 MHz, CD_3_OD) δ −80.7; HRMS (ESI) *m*/*z* calcd for C_16_H_8_N_2_OBr_2_F_6_ [M]^•+^ 515.8908, found 515.8907.

##### (6S*,12S*)-2,8-Difluoro-6,12-bis(trifluoromethyl)-5,6,11,12-tetrahydro-6,12-epoxydibenzo[*b*,*f*][1,5]diazocine (**2c**)

The title compound was obtained according to GP3 using ketone **1c** (103.6 mg, 0.5 mmol) and TMG (12.5 μL, 0.1 mmol, 20 mol %). The crude product was chromatographed on silica (5% EtOAc/hexane) to give a white solid (73.3 mg, 74%). The reaction was also performed on a 4.82 mmol scale (1.0 g of *o*-TMFK **1c**) to give the product in 70% yield (670.3 mg): mp 169–170 °C (*n*-heptane); ^1^H NMR (400 MHz, CDCl_3_) δ 7.23–7.17 (m, 2H), 7.02–6.96 (m, 2H), 6.86 (dd, *J* = 8.9, 4.9 Hz, 2H), 4.77 (br s, 2H, 2 × N*H*); ^19^F NMR (376 MHz, CDCl_3_) δ −79.3, −118.8. The spectroscopic data are in agreement with those reported previously.^25^

##### (6S*,12S*)-6,12-Bis(trifluoromethyl)-5,6,11,12-tetrahydro-6,12-epoxydibenzo[*b*,*f*][1,5]diazocine (**2d**)

The title compound was obtained according to GP3 (16 h, 120 °C) using ketone **1d** (95.0 mg, 0.5 mmol) and TMG (12.5 μL, 0.1 mmol, 20 mol %). The crude product was chromatographed on silica (30–50% toluene/EtOAc) to give a yellow solid (77.6 mg, 86%): mp 140–141 °C (*n*-heptane at −78 °C); IR (KBr) 3413, 3336, 3081, 3054, 1955, 1922, 1802, 1612, 1586, 1495 cm^–1^; ^1^H NMR (400 MHz, CDCl_3_) δ 7.50–7.42 (m, 2H), 7.24–7.18 (m, 2H), 6.98–6.91 (m, 2H), 6.85–6.79 (m, 2H), 4.90 (s, 2H, 2 × N*H*); ^13^C{^1^H} NMR (100 MHz, CDCl_3_) δ 140.0, 130.4, 125.4 (q, *J*_CF_ = 3.0 Hz), 122.8 (q, *J*_CF_ = 282.4 Hz), 122.0, 120.4, 119.1, 83.2 (q, *J*_CF_ = 32.0 Hz); ^19^F NMR (376 MHz, CDCl_3_) δ −79.2; HRMS (ESI-TOF) *m*/*z* calcd for C_16_H_9_F_6_N_2_O [M – H]^−^ 359.0619, found 359.0628.

##### (6S*,12S*)-2,8-Dimethyl-6,12-bis(trifluoromethyl)-5,6,11,12-tetrahydro-6,12-epoxydibenzo[*b*,*f*][1,5]diazocine (**2e**)

The title compound was obtained according to GP3 (16 h, 120 °C) using ketone **1e** (203.2 mg, 0.5 mmol) and TMG (12.5 μL, 20 mol %, 0.1 mmol). The crude product was chromatographed on silica (50% hexane/toluene) to give an off-white solid (72.8 mg, 74%). The reaction was also performed on a 4.8 mmol scale (1.0 g of *o*-TMFK **1e**) to give product **2e** in 69% yield (659.3 mg): mp 164–165 °C (*n*-heptane, −20 °C); IR (KBr) 3361, 3308, 3029, 2930, 2868, 2742, 1621, 1585, 1507 cm^–1^; ^1^H NMR (400 MHz, CDCl_3_) δ 7.25 (br s, 2H), 7.03 (dd, *J* = 8.2, 1.5 Hz, 2H), 4.74 (d, *J* = 8.2 Hz, 2H), 4.74 (br s, 2H, N*H*), 2.25 (s, 6H, 2 × C*H*_3_); ^13^C{^1^H} NMR (100 MHz, CDCl_3_) δ 137.5, 131.8, 131.3, 125.7 (q, *J*_CF_*=* 2.9 Hz), 122.9 (q, *J*_CF_*=* 282.4 Hz), 120.5, 119.5, 83.5 (q, *J*_CF_*=* 31.7 Hz), 20.8 (*C*H_3_); ^19^F NMR (376 MHz, CDCl_3_) δ −79.2; HRMS (ESI) *m*/*z* calcd for C_18_H_15_F_6_N_2_O [M + H]^+^ 389.1089, found 389.1086.

##### (6S*,12S*)-2,8-Dimethoxy-6,12-bis(trifluoromethyl)-5,6,11,12-tetrahydro-6,12-epoxydibenzo[*b*,*f*][1,5]diazocine (**2f**)

The title compound was obtained according to GP3 (16 h, 120 °C) using ketone **1f** (109.6 mg, 0.5 mmol) and TMG (12.5 μL, 0.1 mmol, 20 mol %). The crude product was chromatographed on silica (10–15% EtOAc/hexanes) to give a white solid (75.7 mg, 72%): mp 225–226 °C (DCM; by slow evaporation); IR (KBr) 3357, 3305, 3024, 2960, 2845, 1620, 1592, 1508 cm^–1^; ^1^H NMR (400 MHz, CDCl_3_) δ 6.99 (br s, 2H), 6.87–6.79 (m, 4H), 4.58 (br s, 2H, 2 × N*H*), 3.73 (s, 6H, 2 × OC*H*_3_); ^13^C{^1^H} NMR (100 MHz, CDCl_3_) δ 155.3, 133.2, 122.8 (q, *J*_CF_ = 282.3 Hz), 121.8, 121.7, 117.2, 110.4 (q, *J*_CF_ = 3.1 Hz), 83.9 (q, *J*_CF_ = 31.5 Hz), 55.5 (O*C*H_3_); ^19^F NMR (376 MHz, CDCl_3_) δ −79.2; HRMS (ESI) *m*/*z* calcd for C_18_H_15_F_6_N_2_O_3_ [M + H]^+^ 421.0987, found 421.0984.

##### (6S*,12S*)-*N*,*N*,*N*′,*N*′-Tetramethyl-6,12-bis(trifluoromethyl)-5,6,11,12-tetrahydro-6,12-epoxydibenzo[*b*,*f*][1,5]diazocine-2,8-diamine (**2g**)

The title compound was obtained according to GP3 (16 h, 120 °C) using ketone **1g** (116.1 mg, 0.5 mmol) and TMG (12.5 μL, 0.1 mmol, 20 mol %). The crude product was chromatographed on silica (25% EtOAc/hexanes) to give a yellow oil (58.7 mg, 53%): IR (KBr) 3324, 2888, 2802, 1729, 1621, 1515 cm^–1^; ^1^H NMR (400 MHz, CDCl_3_) δ 6.84 (br s, 2H), 6.81–6.75 (m, 2H), 6.70 (dd, *J* = 8.7, 2.0 Hz, 2H), 4.51 (br s, 2H, 2 × N*H*), 2.85 [s, 12H, 2 × N(C*H*_3_)_2_]; ^13^C{^1^H} NMR (100 MHz, CDCl_3_) δ 146.7, 130.5, 123.1 (q, *J*_CF_ = 282.4 Hz), 122.1, 121.5, 116.1, 109.6, 84.3 (q, *J*_CF_ = 30.9 Hz), 41.1 [N(*C*H_3_)_2_]; ^19^F NMR (376 MHz, CDCl_3_) δ −79.2; HRMS (ESI) *m*/*z* calcd for C_20_H_20_F_6_N_4_O [M + H]^+^ 447.1614, found 447.1610.

##### Dimethyl (6SR*,12SR*)-6,12-Bis(trifluoromethyl)-5,6,11,12-tetrahydro-6,12-epoxydibenzo[*b*,*f*][1,5]diazocine-2,8-dicarboxylate (**2h**)

The title compound was obtained according to GP3 (16 h, 120 °C) using ketone **1h** (123.6 mg, 0.5 mmol) and TMG (12.5 μL, 0.1 mmol, 20 mol %). The residue was chromatographed on silica (10–15% EtOAc/hexanes) to give a white solid (145.0 mg, 56%). The reaction was also performed on a 4.1 mmol scale (1.0 g of *o*-TMFK **1h**) to give the product in 62% yield (597.2 mg): mp 256–257 °C (*n*-heptane); IR (KBr) 3350, 3308, 3010, 2958, 2850, 2489, 1716, 1620, 1508, 1491 cm^–1^; ^1^H NMR (400 MHz, CD_3_OD) δ 7.54–7.47 (m, 4H), 7.44 (dd, *J* = 8.3, 1.8 Hz, 2H), 4.80 (s, 2H), 3.82 [s, 6H, 2 × (CO_2_C*H*_3_)]; ^13^C{^1^H} NMR (100 MHz, CD_3_OD) δ 167.3 (*C*O_2_CH_3_), 143.3, 133.0, 126.5 (q, *J*_CF_ = 2.9 Hz), 133.0, 124.2 (q, *J*_CF_ = 281.5 Hz), 121.7, 119.5, 84.3 (q, *J*_CF_ = 32.4 Hz), 52.7 (CO_2_*C*H_3_); ^19^F NMR (376 MHz, CD_3_OD) δ −80.4; HRMS (ESI) *m*/*z* calcd for C_20_H_14_N_2_O_5_F_6_Na [M + Na]^+^ 499.0705, found 499.0691.

##### (6S*,12S*)-2,6,8,12-Tetrakis(trifluoromethyl)-5,6,11,12-tetrahydro-6,12-epoxydibenzo[*b*,*f*][1,5]diazocine (**2i**)

The title compound was obtained according to GP3 (16 h, 120 °C) using ketone **1i** (257.1 mg, 0.5 mmol) and TMG (12.5 μL, 0.1 mmol, 20 mol %). The crude product was chromatographed on silica (10% EtOAc/hexanes) to give an off-white solid (28.3 mg, 23%). The reaction was conducted on a 0.5 mmol scale for 48 h at 120 °C to afford the product in 39% yield after chromatography (47.8 mg): mp 142–143 °C (*n*-heptane); IR (KBr) 3413, 1631, 1595, 1524; ^1^H NMR (400 MHz, CDCl_3_) δ 7.69 (br s, 2H), 7.50 (dd, *J* = 8.5, 1.6 Hz, 2H), 6.93 (d, *J* = 8.6 Hz, 2H), 5.30 (br s, 2H, 2 × N*H*); ^13^C{^1^H} NMR (100 MHz, CDCl_3_) δ 142.6, 127.8 (q, *J*_CF_ = 3.5 Hz), 124.1 (q, *J*_CF_ = 33.1 Hz), 123.7 (q, *J*_CF_ = 270.0 Hz), 122.3 (q, *J*_CF_ = 282.5 Hz), 123.0–122.6 [m, F_3_CCH*C*CCF_3_(NH)], 119.4, 118.6, 82.5 (q, *J*_CF_ = 32.7 Hz); ^19^F NMR (470 MHz, CDCl_3_) δ −62.1, −79.1; HRMS (EI) *m*/*z* calcd for C_18_H_8_F_12_N_2_O [M]^+•^ 496.0445, found 496.0455.

##### (6S*,12S*)-2,8-Dichloro-6,12-bis(heptafluoropropyl)-5,6,11,12-tetrahydro-6,12-epoxydibenzo[*b*,*f*][1,5]diazocine (**2j**)

The title compound was obtained according to GP3 (16 h, 120 °C) using ketone **1j** (161.8 mg, 0.5 mmol) and TMG (12.5 μL, 20 mol %, 0.1 mmol). The crude product was chromatographed on silica (5% EtOAc/hexane) to give an off-white solid (65.2 mg, 41%): mp 125–127 °C (*n*-heptane); IR (KBr) 3382, 2932, 1709, 1610, 1490 cm^–1^; ^1^H NMR (400 MHz, CDCl_3_) δ 7.41 (br s, 2H), 7.21 (dd, *J* = 8.6, 2.3 Hz, 2H), 6.80 (d, *J* = 8.7 Hz, 2H), 4.97 (br s, 2H, NH); ^13^C{^1^H} NMR (100 MHz, CDCl_3_) δ 138.3, 130.9, 127.3, 125.7–125.9 (m), 121.6, 120.6, 84.6–83.8 (m) (signals of perfluorinated groups have been omitted from the description of the ^13^C NMR spectrum for the sake of clarity due to complicated multiplicity); ^19^F NMR (376 MHz, CDCl_3_) δ −80.8 to −80.9 (m, 3F), −116.5 to −119.3 (m, 2F), −121.6 to −124.3 (m, 2F); HRMS (ESI) *m*/*z* calcd for C_20_H_7_F_14_N_2_O [M – H]^−^ 626.9712, found 626.9726.

(6S*,12S*)-2,8-Bis(prop-2-yn-1-yloxy)-6,12-bis(trifluoromethyl)-5,6,11,12-tetrahydro-6,12-epoxydibenzo[*b*,*f*][1,5]diazocine (**2k**). The title compound was obtained according to GP3 (16 h, 80 °C) using ketone **1k** (100 mg, 0.41 mmol) and TMG (10.5 μL, 0.1 mmol, 20 mol %). The crude product was chromatographed on silica (40% DCM/hexanes) to give a white solid (95.0 mg, 99%). The reaction was slightly scaled up using ketone **1k** (287.0 g, 1.18 mmol) and TMG (30 μL, 0.236 mmol, 20 mol %) to give a white solid (238.5 mg, 86%): mp 159–160 °C (DCM/*n*-heptane); ^1^H NMR (400 MHz CDCl_3_) δ 7.10 (br s, 2H), 6.98–6.75 (m, 4H), 4.65 (br s, 2H, N*H*), 4.60 (br s, 4H, OC*H*_2_), 2.50 (br s, 2H, CH_2_CC*H*); ^13^C{^1^H} NMR (100 MHz, CDCl_3_) δ 153.2, 134.2, 122.9 (q, *J*_CF_ = 282.6 Hz), 121.8, 121.6, 118.3, 112.3 (q, *J*_CF_ = 2.7 Hz), 83.9 (q, *J*_CF_ = 31.4 Hz), 78.3, 75.9, 56.6; ^19^F NMR (376 MHz, CDCl_3_) δ −79.2; HRMS (ESI) *m*/*z* calcd for C_22_H_15_F_6_N_2_O_3_ [M + H]^+^ 469.0987, found 469.0996.

(6S*,12S*)-2,8-Bis(prop-2-yn-1-yloxy)-6,12-bis(trifluoromethyl)-5,6,11,12-tetrahydro-6,12-epoxydibenzo[*b*,*f*][1,5]diazocine (**2l**). The title compound was obtained according to GP3 (16 h, 80 °C) using ketone **1l** (122.6 mg, 0.5 mmol) and TMG (12.5 μL, 0.1 mmol, 20 mol %). The crude product was chromatographed on silica (15–30% DCM/hexanes) to give a white solid (102.4 mg, 87%). The reaction was conducted on a 4.1 mmol scale using ketone **1l** (1.0 g, 4.08 mmol) and TMG (102 μL, 0.82 mmol, 20 mol %) to give **2l** as a white solid (918.0 mg, 95%): mp >160 °C dec (DCM/*n*-heptane); ^1^H NMR (400 MHz CDCl_3_) δ 7.04–7.00 (m, 2H), 6.87–6.76 (m, 4H), 6.05–5.94 (m, 2H, CH_2_C*H*=CH_2_), 5.37 (ddd, *J* = 17.2, 3.2, 1.6 Hz, 2H, CH_2_C*H*=C*H*H’), 5.27 (ddd, *J* = 10.5, 2.8, 1.4 Hz, 2H, CH_2_C*H*=CH*H*′), 4.57 (m, 2H), 4.47–4.42 (m, 4H, OCH_2_); ^13^C{^1^H} NMR (50 MHz, CDCl_3_) δ 154.3, 133.5, 133.1, 123.0 (q, *J*_CF_ = 282.5 Hz), 121.9, 121.7, 118.1, 118.0, 111.6 (q, *J*_CF_ = 3.2 Hz), 84.0 (q, *J*_CF_ = 31.5 Hz), 84.9, 84.3, 83.6, 83.0, 69.4; ^19^F NMR (376 MHz, CDCl_3_) δ −79.2; HRMS (ESI) *m*/*z* calcd for C_22_H_19_F_6_N_2_O_3_ [M + H]^+^ 473.1300, found 473.1315.

(6S*,12S*)-2,8-Bis(but-3-en-1-yloxy)-6,12-bis(trifluoromethyl)-5,6,11,12-tetrahydro-6,12-epoxydibenzo[*b*,*f*][1,5]diazocine (**2m**). The title compound was obtained according to GP3 (16 h, 80 °C) using ketone **1m** (501.1 mg, 1.92 mmol) and TMG (48.4 μL, 0.39 mmol, 20 mol %). The progress of the reaction was monitored by TLC on alumina. The crude product was chromatographed on aluminum oxide (Brockman activity scale I, 5–10% EtOAc/hexanes) to give a white solid (407.5 mg, 84%): mp 138–139 °C (DCM/*n*-heptane); ^1^H NMR (400 MHz, CDCl_3_) δ 7.00 (br s, 2H), 6.85–6.74 (m, 4H), 5.93–5.79 (m, 2H), 5.19–5.04 (m, 4H), 4.58 (s, 2H), 3.97–3.87 (m, 4H), 2.54–2.43 (m, 4H); ^13^C{^1^H} NMR (100 MHz, CDCl_3_) δ 154.6, 134.3, 133.3, 122.8 (q, *J* = 282.5 Hz), 121.8, 121.6, 117.8, 117.1, 83.9 (q, *J* = 31.5 Hz), 67.7, 33.6; ^19^F NMR (376 MHz, CDCl_3_) δ −79.2; HRMS (ESI) *m*/*z* calcd for C_24_H_23_F_6_N_2_O_3_ [M + H]^+^ 501.1613, found 501.1609.

##### (6S*,12S*)-2,8-Bis(pent-4-en-1-yloxy)-6,12-bis(trifluoromethyl)-5,6,11,12-tetrahydro-6,12-epoxydibenzo[*b*,*f*][1,5]diazocine (**2n**)

The title compound was obtained according to GP3 (16 h, 80 °C) using ketone **1n** (1.50 g, 5.49 mmol) and TMG (138 μL, 0.1 mmol, 20 mol %). The crude product was chromatographed on silica (20–30% DCM/hexanes) to give an off-white solid (1.30 g, 89%): mp 125–127 °C (DCM/*n*-heptane); ^1^H NMR (400 MHz CDCl_3_) δ 6.99 (s, 2H), 6.85–6.76 (m 4H), 5.90–5.77 (m, 2H, CH_2_=C*H*CH_2_), 5.10–4.96 (m, 4H, C*H*_2_=CHCH_2_), 4.59 (br s, 2H), 3.88 (t, *J* = 6.3 Hz, 4H, OC*H*_2_), 2.25–2.16 (m, 4H, CH_2_=CHC*H*_2_), 1.88–1.79 (m, 4H, CH_2_C*H*_2_CH_2_); ^13^C{^1^H} NMR (100 MHz, CDCl_3_) δ 154.9, 137.9, 133.3, 123.0 (q, *J*_CF_ = 282.4 Hz), 122.0, 121.7, 117.8, 115.3, 111.3 (q, *J*_CF_ = 3.0 Hz), 84.0 (q, *J*_CF_ = 31.4 Hz), 67.7, 30.2, 28.5; ^19^F NMR (376 MHz, CDCl_3_) δ −79.2; HRMS (ESI) *m*/*z* calcd for C_26_H_26_F_6_N_2_O_2_Na [M + Na]^+^ 551.1745, found 551.1742.

##### (6S*,12S*)-2,8-Dichloro-6,12-bis(difluoromethyl)-5,6,11,12-tetrahydro-6,12-epoxydibenzo[*b*,*f*][1,5]diazocine (**2o**)

The title compound was obtained according to GP3 (16 h, 120 °C) using ketone **1o** (102.8 mg, 0.5 mmol) and TMG (12.5 μL, 0.1 mmol, 20 mol %). The crude product was chromatographed on silica (10% EtOAc/hexanes) to give an off-white solid (74.4 mg, 76%). The reaction was conducted on a 4.9 mmol scale (1.0 g) to afford 668.1 mg of diazocine **2o** (70%): mp 175–177 °C (*n-*heptane); IR (KBr) 3372, 3320, 3072, 3000, 1897, 1775, 1739, 1608, 1577, 1499 cm^–1^; ^1^H NMR (400 MHz, CDCl_3_) δ 7.40 (br s, 2H), 7.16 (dd, *J* = 8.6, 2.3 Hz, 2H), 6.74 (d, *J* = 8.6 Hz, 2H), 6.02 (dd, *J*_HF_ = 54.9, 54.9 Hz, 2H, 2 × C*H*F_2_), 4.88 (br s, 2H, 2 × N*H*); ^13^C{^1^H} NMR (100 MHz, CDCl_3_) δ 138.9, 130.2, 126.4, 125.6 (dd, *J*_CF_ = 4.2, 4.2 Hz), 122.3, 119.6, 114.1 (dd, *J*_CF_ = 254.2, 248.9 Hz), 81.7 (dd, *J*_CF_ = 25.3, 23.2 Hz); ^19^F NMR (376 MHz, CDCl_3_) δ −129.7 (d, *J* = 290.4 Hz), −131.6 (d, *J* = 290.5 Hz); HRMS (ESI) *m*/*z* calcd for C_16_H_9_F_4_N_2_OCl_2_ [M – H]^−^ 391.0028, found 391.0034.

##### (6S*,12S*)-7,15-Bis(trifluoromethyl)-7,8,15,16-tetrahydro-7,15-epoxydinafto[1,2-*b*:1′,2′-*f*][1,5]diazocine (**2p**)

The title compound was obtained according to GP3 (16 h, 120 °C) using ketone **1p** (119.6 mg, 0.5 mmol) and TMG (12.5 μL, 20 mol %, 0.1 mmol). The crude product was chromatographed on silica (5% EtOAc/hexane; the *R_f_* of **2p** is slightly higher than that of substrate **1p**) to give a bright yellow solid (50.8 mg, 44%): mp 208–210 °C (*n*-heptane); IR (KBr) 3321, 3075, 1582, 1514 cm^–1^; ^1^H NMR (400 MHz, CDCl_3_) δ 8.05 (d, *J* = 8.0 Hz, 2H), 7.74 (d, *J* = 7.6 Hz, 2H), 7.60 (d, *J* = 8.7 Hz, 2H), 7.57–7.41 (m, 6H), 5.25 (s, 2H, 2 × N*H*); ^13^C{^1^H} NMR (100 MHz, CDCl_3_) δ 136.3, 134.0, 128.2, 127.4, 126.5, 125.8, 123.2 (q, *J*_CF_ = 282.9 Hz), 122.9, 122.2 (q, *J*_CF_ = 3.0 Hz), 121.1, 116.0, 84.1 (q, *J*_CF_ = 31.6 Hz); ^19^F NMR (376 MHz, CDCl_3_) δ −78.2; HRMS (ESI) *m*/*z* calcd for C_24_H_14_F_6_N_2_ONa [M + Na]^+^ 483.0908, found 483.0899.

(6*S*,12*S*)-2,8-Bis{[(1*R*,2*S*,5*R*)-5-methyl-2-(propan-2-yl)cyclohexyl]oxy}-6,12-bis(trifluoromethyl)-5,6,11,12-tetrahydro-6,12-epoxydibenzo[*b*,*f*][1,5]diazocine (**2q**) and (6*R*,12*R*)-2,8-Bis{[(1*R*,2*S*,5*R*)-5-methyl-2-(propan-2-yl)cyclohexyl]oxy}-6,12-bis(trifluoromethyl)-5,6,11,12-tetrahydro-6,12-epoxydibenzo[*b*,*f*][1,5]diazocine (**2q′**). The title compounds were obtained according to GP3 (16 h, 120 °C) using ketone **1q** (200 mg, 0.58 mmol) and TMG (14.6 μL, 0.12 mmol, 20 mol %). The crude product was chromatographed on silica (2% EtOAc/hexane) to give a yellow foam (143.7 mg, 74%). The diastereomeric ratio was estimated on the basis of ^19^F NMR to be 53:47. The diastereomeric ratio was independently determined by HPLC analysis using a Daicel Chiralpak OD-H column (2% *i*-PrOH/hexane, flow rate of 1.0 mL/min, λ = 235 nm) to give a similar result (52:48): *t*_R_ = 5.4 min (major), *t*_R_ = 9.2 min (minor); IR (KBr) 3333, 2956, 2927, 2871, 1723, 1617, 1581, 1502 cm^–1^; ^1^H NMR (400 MHz, CDCl_3_) δ 6.99 (br s, 2H), 6.86–6.75 (m, 4H), 4.55 and 4.55 (2 × br s, 2H, N*H*), 3.92–3.81 (m, 2H), 2.25–2.11 (m, 2H), 2.10–1.98 (m, 2H), 1.75–1.65 (m, 4H), 1.50–1.34 (m, 4H), 1.14–0.94 (m, 4H), 0.94–0.82 (m, 14H), 0.75 (d, *J* = 6.7 Hz, 3H), 0.73 (d, *J* = 6.7 Hz, 3H); ^13^C{^1^H} NMR (100 MHz, CDCl_3_) δ 153.9 (×2), 133.2, 133.1, 122.9 (q, *J*_CF_ = 282.6 Hz, ×2), 121.8 (×2), 121.4, 121.3, 119.3, 119.0, 113.5 (q, *J*_CF_ = 2.7 Hz), 113.2 (q, *J*_CF_ = 2.8 Hz), 83.8 (q, *J*_CF_ = 31.5 Hz, ×2), 78.7, 78.6, 48.2 (×2), 40.3, 40.2, 34.5, 34.4, 31.4, 25.9 (×2), 23.6, 23.5, 22.1, 20.8, 16.4, 16.3; ^19^F NMR (376 MHz, CDCl_3_) δ −79.2, −79.3; HRMS (EI) *m*/*z* calcd for C_36_H_46_F_6_N_2_O_3_ [M]^+•^ 668.3413, found 668.3405.

##### (5S*,11S*)-3,9-Dichloro-5,11-bis(trifluoromethyl)-5,6,11,12-tetrahydro-5,11-epoxydipyrido[2,3-*b*:2′,3′-*f*][1,5]diazocine (**4a**)

The title compound was obtained according to GP3 (16 h, 120 °C) using ketone **2a** (112.3 mg, 0.5 mmol) and TMG (12.5 μL, 20 mol %, 0.1 mmol). The crude product was chromatographed on silica (5% EtOAc/toluene) to give a light gray solid (105.2 mg, 98%): mp 252–253 °C (*n*-heptane); IR (KBr) 3190, 3057, 2925, 1869, 1842, 1607, 1577, 1505 cm^–1^; ^1^H NMR (400 MHz, CD_3_OD) δ 8.17 (d, *J* = 2.3 Hz, 2H), 7.78–7.74 (m, 2H), 4.54 (br s, 2H, 2 × N*H*); ^13^C{^1^H} NMR (100 MHz, CD_3_OD) δ 153.4, 149.9, 134.6 (q, *J*_CF_ = 2.8 Hz), 123.9, 123.0 (q, *J*_CF_ = 281.7 Hz), 117.0, 84.3 (q, *J*_CF_ = 33.5 Hz); ^19^F NMR (376 MHz, CD_3_OD) δ −80.7; HRMS (EI) *m*/*z* calcd for C_14_H_6_Cl_2_F_6_N_4_O [M]^•+^ 429.9823, found 429.9817.

##### (5S*,11S*)-5,11-Bis(trifluoromethyl)-5,6,11,12-tetrahydro-5,11-epoxydipyrido[2,3-*b*:2′,3′-*f*][1,5]diazocine (**4b**)

The title compound was obtained according to GP3 (16 h, 120 °C) using ketone **2c** (95.0 mg, 0.5 mmol) and TMG (12.5 μL, 0.1 mmol, 20 mol %). The crude product was chromatographed on silica (5% MTBE/DCM to 5–10% MeOH/DCM) to give a white solid (59.2 mg, 65%): mp 309–310 °C (EtOH/*n*-heptane, product not soluble in CHCl_3_, DCM, *n*-heptane, MeOH); IR (KBr) 3161, 3105, 3004, 2929, 2880, 1914, 1912, 1609, 1587, 1525 cm^–1^; ^1^H NMR (400 MHz, DMSO-*d*_6_) δ 8.96 (br s, 2H, 2 × N*H*), 8.17 (dd, *J* = 4.8, 1.5 Hz, 2H), 7.73 (d, *J* = 7.8 Hz, 2H), 6.90 (dd, *J* = 7.8, 4.8 Hz, 2H); ^13^C{^1^H} NMR (100 MHz, DMSO-*d*_6_) δ 153.7, 150.3, 134.1, 122.5 (q, *J*_CF_ = 282.5 Hz), 118.3, 116.1, 114.6, 83.0 (q, *J*_CF_ = 32.6 Hz); ^19^F NMR (376 MHz, DMSO-*d*_6_) δ −78.3; HRMS (ESI) *m*/*z* calcd for C_14_H_9_F_6_N_4_O [M + H]^+^ 363.0681, found 363.0670.

##### (6S*,12S*)-6,12-Bis(trifluoromethyl)-5,6,11,12-tetrahydro-6,12-epoxydipyrido[4,3-*b*:4′,3′-*f*][1,5]diazocine (**4c**)

The title compound was obtained according to GP3 (16 h, 120 °C) using ketone **2b** (95.1 mg, 0.5 mmol) and TMG (12.5 μL, 0.1 mmol, 20 mol %). The crude product was chromatographed on silica (6–10% MeOH/DCM) to give an off-white solid (73.8 mg, 85%): mp >325 °C dec; IR (KBr) 3176, 3126, 3094, 3038, 2993, 2928, 2873, 2789, 2839, 2518, 2322, 1938, 1901, 1866, 1617, 1585, 1531 cm^–1^; ^1^H NMR (400 MHz, CD_3_OD) δ 8.37 (s, 2H), 8.16 (d, *J* = 5.8 Hz, 2H), 6.85 (d, *J* = 5.8 Hz, 2H); ^13^C{^1^H} NMR (100 MHz, CD_3_OD) δ 150.6, 150.1, 146.4–146.1 (m, 1H), 123.8 (q, *J*_CF_ = 281.7 Hz), 117.0, 112.4, 82.7 (q, *J*_CF_ = 33.8 Hz); ^19^F NMR (376 MHz, CD_3_OD) δ −80.7; HRMS (ESI) *m*/*z* calcd for C_14_H_9_F_6_N_4_O [M + H]^+^ 363.0681, found 363.0671.

#### General Procedure for the Synthesis of Unsymmetrical Epoxydibenzo[*b*,*f*][1,5]diazocines (GP4)

Fluoromethylketone pyridine-derived **3a** (FMK, *x* mmol), fluoroketone **1a** (2 equiv), and *N*,*N*,*N′*,*N′*-tetramethylguanidine (TMG, 20 mol %/equiv of aminophenone) were placed in a screw-cap vial (4 mL), and the resulting mixture was heated at 120 °C (IKA heating block, temperature of the reference vial filled with silicon oil). Then the reaction mixture was diluted with EtOAc or DCM (10 mL), adsorbed on silica (or aluminum oxide), and chromatographed to give corresponding epoxydibenzo[*b*,*f*][1,5]diazocines **5**. The analytical sample was crystallized from the appropriate solvent to measure the melting point. In all cases, in addition to cross-condensation product **5**, diazocine **2a** was formed and isolated via chromatography. In addition, diazocine **4b** (resulting from autocondensation of **3c**) and unreacted aminophenone **3c** were detected by TLC (**4b** and **3c** were not isolated due to their marginal amounts).

##### (5S*,11S*)-9-Fluoro-5,11-bis(trifluoromethyl)-5,6,11,12-tetrahydro-5,11-epoxypyrido[3,2-*c*][1,5]benzodiazocine (**5a**)

The title compound was obtained according to GP4 (16 h, 120 °C) using ketone **1a** (223.6 mg, 1.0 mmol), ketone **3c** (95.1 mg, 0.5 mmol), and TMG (37.5 μL, 0.3 mmol, 20 mol %/equiv of aminophenone). The residue was chromatographed on silica (10–25% EtOAc/hexanes) to give diazocine **2a** (127.1 mg) and a mixture of diazocine **5a** and pyridine derivative **3c**. The resulting mixture was chromatographed on alumina (15% EtOAc/hexanes, activity on Brockman scale III) to give **5a** as a white solid (126.4 mg, 64%).

##### Experiment on a 2.5 mmol Scale

Ketone **1a** (1.118 g, 5.0 mmol, 2 equiv), ketone **3c** (475.5 mg, 2.5 mmol), and TMG (188 μL, 1.5 mmol) were placed in a 4 mL screw-cap vial and heated at 120 °C for 16 h. Then the reaction mixture was cooled to rt, dissolved in a mixture of MeOH and DCM (10% MeOH/DCM), adsorbed on silica (10 g), and chromatographed (10–25% EtOAc/hexanes) to give a light-yellow solid of **5a** (469.3 mg, 48%). In addition, **2a** (712.1 mg) was also isolated: mp 227–229 °C (*n*-heptane); IR (KBr) 3381, 3341, 3196, 3181, 3168, 3097, 3077, 3002, 2948, 1600, 1589, 1512 cm^–1^; ^1^H NMR (400 MHz, CD_3_OD) δ 8.13 (dd, *J* = 4.9, 1.7 Hz, 1H), 7.79–7.73 (m, 1H), 7.45–7.40 (m, 1H), 7.19 (dd, *J* = 8.7, 2.4 Hz, 1H), 6.86 (dd, *J* = 7.8, 4.9 Hz, 1H) overlapping 6.84 (d, *J* = 8.8 Hz, 1H), 4.55 (br s, 2H, 2 × N*H*); ^13^C{^1^H} NMR (100 MHz, CD_3_OD) δ 155.3, 150.8, 141.4, 135.5 (q, *J*_CF_ = 2.2 Hz), 131.4, 126.4, 126.0 (q, *J*_CF_ = 3.3 Hz), 124.1 (q, *J*_CF_ = 281.6 Hz), 123.9 (q, *J*_CF_ = 281.6 Hz), 122.5, 120.5, 117.0, 116.5, 84.3 (q, *J*_CF_ = 32.6 Hz), 84.2 (q, *J*_CF_ = 33.0 Hz); ^19^F NMR (376 MHz, CD_3_OD) δ −80.6, −80.7; HRMS (ESI) *m*/*z* calcd for C_15_H_9_ClF_6_N_3_O [M + H]^+^ 396.0338, found 396.0329.

##### (5S*,11S*)-9-Methyl-5,11-bis(trifluoromethyl)-5,6,11,12-tetrahydro-5,11-epoxypyrido[3,2-*c*][1,5]benzodiazocine (**5b**)

The title compound was obtained according to GP4 (16 h, 120 °C) using ketone **1c** (207.1 mg, 1.0 mmol), ketone **3c** (95.1 mg, 0.5 mmol), and TMG (37.5 μL, 0.3 mmol, 20 mol %/equiv of aminophenone). The residue was chromatographed on silica (10% EtOAc/hexanes) to give **2c** (154.9 mg) and a mixture of diazocine **5b** and pyridine derivative **3c**. The resulting mixture of **5b** and **3c** was further chromatographed on alumina (15% EtOAc/hexanes, activity on Brockman scale III) to give **5b** as a white solid (66.4 mg, 35%): mp 196–197 °C (*n*-heptane); IR (KBr) 3356, 3161, 3098, 2994, 2926, 1604, 1588, 1445 cm^–1^; ^1^H NMR (400 MHz, CD_3_OD) δ 8.12 (dd, *J* = 4.8, 1.4 Hz, 1H), 7.81–7.72 (m, 1H), 7.22–7.15 (m, 1H), 7.03–6.96 (m, 1H), 6.91–6.82 (m, 2H), 4.55 (br s, 1H, N*H*); ^13^C{^1^H} NMR (100 MHz, CD_3_OD) δ 158.6 (d, *J*_CF_*=* 237.5 Hz), 155.4, 150.8, 138.6 (d, *J*_CF_ = 1.8 Hz), 135.7 (q, *J*_CF_*=* 2.7 Hz), 124.1 (q, *J*_CF_ = 281.6 Hz), 123.8 (q, *J*_CF_ = 281.6 Hz), 122.3 (d, *J*_CF_*=* 6.8 Hz), 121.2 (d, *J*_CF_*=* 7.6 Hz), 118.6 (d, *J*_CF_*=* 23.1 Hz), 117.0, 116.5, 112.5 (dq, *J*_CF_*=* 24.8, 3.0 Hz), 84.7–83.6 (m) overlapping 84.7 (q, *J*_CF_*=* 32.2 Hz); ^19^F NMR (376 MHz, CD_3_OD) δ −80.7 (×2), −123.7; HRMS (ESI) *m*/*z* calcd for C_15_H_9_F_7_N_3_O [M + H]^+^ 380.0634, found 380.0633.

(5S*,11S*)-9-Chloro-1-methyl-5,11-bis(trifluoromethyl)-5,6,11,12-tetrahydro-5,11-epoxypyrido[3,2-*c*][1,5]benzodiazocin-1-ium iodide (**5c**). The title compound was obtained according to GP4 (16 h, 120 °C) using ketone **1e** (203.2 mg, 1.0 mmol), ketone **3c** (95.1 mg, 0.5 mmol), and TMG (37.5 μL, 0.3 mmol, 20 mol %/equiv of aminophenone). The residue was chromatographed on silica (15–25% EtOAc/hexanes) to give **2e** (135.9 mg) and a mixture of diazocine **5c** and pyridine derivative **3c**. The resulting mixture of **5c** and **3c** was chromatographed on alumina (30% MTBE/hexanes) to give a colorless solid (82.9 mg, 44%): mp 209–210 °C (*n-*heptane); IR (KBr) 3374, 3333, 3182, 3098, 3070, 3000, 2936, 1948, 1928, 1601, 1588, 1516 cm^–1^; ^1^H NMR (400 MHz, CD_3_OD) δ 8.09 (dd, *J* = 4.9, 1.7 Hz, 1H), 7.78–7.73 (m, 1H), 7.24 (br s, 1H), 7.01 (dd, *J* = 8.3, 1.4 Hz, 1H), 6.81 (dd, *J* = 7.8, 4.9 Hz, 1H), 6.76 (d, *J* = 8.2 Hz, 1H), 2.21 (s, 3H, C*H*_3_); ^13^C{^1^H} NMR (100 MHz, CD_3_OD) δ 155.6, 150.5, 139.8, 135.5 (q, *J*_CF_ = 2.8 Hz), 132.0, 131.7, 126.1 (q, *J*_CF_ = 2.8 Hz), 124.3 (q, *J*_CF_ = 281.6 Hz), 124.1 (q, *J*_CF_ = 281.4 Hz), 121.4, 119.5, 117.0, 116.8, 84.7 (q, *J* = 32.1 Hz), 84.5 (q, *J* = 32.7 Hz), 20.7 (C*H*_3_); ^19^F NMR (376 MHz, CD_3_OD) δ −80.4, −80.7; HRMS (EI) *m*/*z* calcd for C_16_H_11_F_6_N_3_O [M]^•+^ 376.0875, found 376.0876.

(5S*,11S*)-9-Chloro-5-(difluoromethyl)-11-(trifluoromethyl)-5,6,11,12-tetrahydro-5,11-epoxypyrido[3,2-*c*][1,5]benzodiazocine (**5d**). The title compound was obtained according to GP4 (16 h, 120 °C) using ketone **1a** (223.6 mg, 1.0 mmol), ketone **3d** (86.1 mg, 0.5 mmol), and TMG (37.5 μL, 0.3 mmol, 20 mol %/equiv of aminophenone). The crude product was chromatographed on silica (10% EtOAc/hexanes) to give **2a** (136.6 mg) and a mixture of diazocine **5d** and pyridine derivative **3d** (a complicated mixture of products was detected by TLC). The resulting mixture was chromatographed on reversed phase silica (20% H_2_O/MeOH, RP-18) to give a colorless solid (22.7 mg, 12%) mp 229–230 °C (*n*-heptane); IR (KBr) 3412, 3383, 3336, 3202, 3090, 2992, 2943, 2850, 1899, 1601, 1565, 1508 cm^–1^; ^1^H NMR (400 MHz, CD_3_OD) δ 8.10 (br s, 1H), 7.77 (d, *J* = 7.8 Hz, 1H), 7.44–7.40 (m, 1H), 7.15 (dd, *J* = 8.7, 2.4 Hz, 1H), 6.86–6.79 (m, 2H), 6.23 (dd, *J*_HF_*=* 54.7, 54.3 Hz, 1H, C*H*F_2_); ^13^C{^1^H} NMR (100 MHz, CD_3_OD) δ 155.5, 150.1, 141.7, 135.8 (dd, *J*_CF_*=* 4.3, 2.5 Hz), 131.2, 126.0 (q, *J*_CF_ = 3.0 Hz), 126.0, 124.1 (q, *J*_CF_*=* 281.6 Hz), 122.8, 120.4, 117.9–117.6 (m), 117.1–116.7 (m), 115.7 (dd, *J*_CF_ = 248.4, 247.0 Hz), 84.4–83.2 (m); ^19^F NMR (376 MHz, CD_3_OD) δ −80.5, −132.0 (dd, *J* = 291.2, 0.0 Hz), −133.6 (dd, *J* = 291.1, 0.0 Hz); HRMS (ESI) *m*/*z* calcd for C_15_H_10_ClF_5_N_3_O [M + H]^+^ 378.0433, found 378.0427.

(5*S*,11*S*)-9-{[(1*R*,2*S*,5*R*)-5-Methyl-2-(propan-2-yl)cyclohexyl]oxy}-5,11-bis(trifluoromethyl)-5,6,11,12-tetrahydro-5,11-epoxypyrido[3,2-*c*][1,5]benzodiazocine (**5e**) and (5*R*,11*R*)-9-{[(1*R*,2*S*,5*R*)-5-Methyl-2-(propan-2-yl)cyclohexyl]oxy}-5,11-bis(trifluoromethyl)-5,6,11,12-tetrahydro-5,11-epoxypyrido[3,2-*c*][1,5]benzodiazocine (**5e′**). The title compounds were obtained according to GP4 (16 h, 120 °C) using ketone **3c** (95.1 mg, 0.5 mmol), ketone **1q** (343.4 mg, 1.0 mmol), and TMG (37.5 μL, 0.3 mmol, 20 mol %/equiv of aminophenone). The residue was chromatographed on silica (10–15% EtOAc/hexanes) to give **2q** (214.4 mg) and an inseparable mixture of **5e** and **5e′** as a white solid (145.0 mg, 56%): mp 197–198 °C (*n-*heptane); IR (KBr) 3309, 3213, 3093, 2960, 2929, 2875, 2395, 1925, 1600, 1504 cm^–1^; ^1^H NMR (600 MHz, CD_3_OD) δ 8.10 (dd, *J* = 4.9, 1.5 Hz, 1H), 7.77 (d, *J* = 7.8 Hz, 1H), 7.01 (br s, 1H), 6.86–6.78 (m, 3H), 3.97–3.88 (m, 1H), 2.22–2.11 (m, 1H), 2.10–2.02 (m, 1H), 1.75–1.66 (m, 2H), 1.50–1.38 (m, 2H), 1.17–1.07 (m, 1H), 0.97–0.84 (m, 8H), 0.75 (d, *J* = 7.0 Hz, 0.5 × 3H), 0.74 (d, *J* = 7.0 Hz, 0.5 × 3H); ^13^C{^1^H} NMR (150 MHz, CD_3_OD) δ 155.6 (×2), 154.4, 154.2, 150.6, 135.8, 135.4, 135.3, 124.3 (q, *J*_CF_ = 281.7 Hz), 124.1 (q, *J*_CF_ = 281.1 Hz), 122.6, 122.5, 121.4, 121.4, 120.3, 120.1, 116.8 (×2), 113.3, 113.2, 85.6–84.5 (m) overlapping 84.3 (q, *J*_CF_ = 32.3 Hz), 79.6, 49.7, 49.6, 41.6, 41.5, 35.7, 35.6, 32.5 (×2), 27.2, 24.7 (×2), 22.5 (×2), 21.1 (×2) 16.9, 16.8; ^19^F NMR (376 MHz, CD_3_OD) δ −80.4, −80.5, −80.7, −80.7; HRMS (ESI) *m*/*z* calcd for C_25_H_28_F_6_N_3_O_2_F_6_ [M + H]^+^ 516.2086, found 516.2081.

### Functionalization of Epoxydibenzo[*b*,*f*][1,5]diazocines

(5S*,11S*)-9-Chloro-1-methyl-5,11-bis(trifluoromethyl)-5,6,11,12-tetrahydro-5,11-epoxypyrido[3,2-*c*][1,5]benzodiazocin-1-ium iodide (**6a**). Epoxydibenzodiazocine **5a** (60 mg, 0.152 mmol, 1 equiv), MeCN (2 mL), and MeI (66 μL, 1.06 mmol, 7 equiv) were placed in a screw-cap vial (4 mL), and the resulting mixture was heated at 80 °C for 48 h (IKA heating block, temperature of the reference vial filled with silicon oil). Then the reaction mixture was evaporated. The residue was chromatographed on silica (5–15% EtOAc/hexanes) to give a pale green solid (63.1 mg, 77%): mp >180 °C dec (*n*-heptane); ^1^H NMR (600 MHz, CD_3_OD) δ 7.37 (dd, *J* = 6.8, 1.7 Hz, 1H), 7.34–7.28 (m, 2H), 7.08 (dd, *J* = 8.7, 2.4 Hz, 1H), 6.78 (d, *J* = 8.6 Hz, 1H), 5.97 (t, *J* = 7.0 Hz, 1H), 3.44 (s, 3H, C*H*_3_); ^13^C{^1^H} NMR (150 MHz, CD_3_OD) δ 156.1, 141.7, 140.7, 133.2 (q, *J*_CF_ = 3.0 Hz), 129.8, 125.9 (q, *J*_CF_ = 3.4 Hz), 125.2, 125.1 (q, *J*_CF_ = 280.9 Hz), 124.7, 124.4 (q, *J*_CF_ = 281.9 Hz), 121.6, 119.6, 103.9, 88.3 (q, *J*_CF_ = 31.3 Hz), 83.0 (q, *J*_CF_ = 32. Hz), 39.5; ^19^F NMR (376 MHz, CD_3_OD) δ −80.7, −81.1; HRMS (ESI) *m*/*z* calcd for C_16_H_11_ClF_6_N_3_O [M]^+^ 410.0495, found 410.0483.

(6S*,12S*)-2,8-Dimethyl-6,12-bis(trifluoromethyl)-5,6,11,12-tetrahydro-6,12-epoxydipyrido[4,3-*b*:4′,3′-*f*][1,5]diazocine-2,8-diium Diiodide (**6b**). To a solution (in a screw-cap 4 mL vial) of epoxydibenzodiazocine **4b** (50 mg, 0.138 mmol, 1 equiv) in a mixture of MeCN (2 mL) and MeOH (1 mL) was added MeI (52 μL, 0.828 mmol, 6 equiv), and the resulting mixture was heated at 90 °C for 18 h (IKA heating block, temperature of the reference). Then the reaction mixture was diluted with MeOH and evaporated with silica. The residue was chromatographed on silica (10–20% MeOH/DCM) to give a white solid (89.2 mg, ∼100%). The reaction was conducted on large scale using epoxydibenzodiazocine **4b** (150 mg, 0.414 mmol), MeI (0.15 mL, 2.49 mmol, 6.0 equiv), MeCN (6 mL), and MeOH (3 mL). The resulting homogeneous mixture (MeOH was used to solubilize diazocine **4b**) was heated at 90 °C for 18 h. Then the reaction mixture was evaporated, redissolved in a minimal volume of MeOH (3 mL), and precipitated with Et_2_O (6 mL). The resulting solid was filtered, washed with Et_2_O (3 × 2 mL), and dried *in vacuo* to give an off-white solid (230.4 mg, 86%): mp >330 °C (MeOH/Et_2_O); ^1^H NMR (400 MHz, CD_3_OD) δ 7.84 (s, 2H), 7.72 (d, *J* = 7.4 Hz, 2H), 6.67 (d, *J* = 7.4 Hz, 2H), 3.82 (s, 6H, 2 × C*H*_3_); ^13^C{^1^H} NMR (125 MHz, CD_3_OD) δ 157.0, 142.3, 138.6, 124.2 (q, *J*_CF_ = 281.4 Hz), 117.0, 114.1, 84.7 (q, *J*_CF_ = 33.0 Hz), 45.2; ^19^F NMR (376 MHz, CD_3_OD) δ −80.5; HRMS (ESI) *m*/*z* calcd for C_16_H_13_F_6_N_4_O [M – H]^+^ 391.0995, found 391.0995.

(6S*,12S*)-*N*,*N*′-Bis(2-hydroxyethyl)-6,12-bis(trifluoromethyl)-5,6,11,12-tetrahydro-6,12-epoxydibenzo[*b*,*f*][1,5]diazocine-2,8-dicarboxamide (**8a**). To a solution of epoxydibenzo[*b*,*f*][1,5]diazocine **2h** (50.0 mg, 0.105 mmol) in THF (1.5 mL) were added ethanolamine **7a** (19.2 mg, 0.315 mmol) and TBD (8.8 mg, 0.063 mmol, 60 mol %). The resulting reaction mixture was heated at 60 °C for 21 h, quenched with a saturated solution of NH_4_Cl (10 mL), and extracted with EtOAc (6 × 15 mL). The combined organic extracts were dried over Na_2_SO_4_ and evaporated. The residue was chromatographed on silica (10–15% MeOH/DCM) to give a white solid (33.6 mg, 60%): mp >119 °C dec (MeOH/DCM); ^1^H NMR (400 MHz, CD_3_OD) δ 7.49 (d, *J* = 8.2 Hz, 2H), 7.29–7.21 (m, 4H), 3.66 (t, *J* = 5.8 Hz, 4H, C*H*_2_), 3.44 (t, *J* = 5.8 Hz, 4H, C*H*_2_); ^13^C{^1^H} NMR (100 MHz, CD_3_OD) δ 169.8 (*C*O_2_R), 143.4, 137.7, 128.5, 126.5 (q, *J*_CF_ = 2.1 Hz), 124.3 (q, *J*_CF_ = 281.5 Hz), 123.9, 119.5, 117.6, 84.3 (q, *J*_CF_ = 32.2 Hz), 61.5, 43.5; ^19^F NMR (376 MHz, CD_3_OD) δ −80.5; HRMS (ESI) *m*/*z* calcd for C_22_H_20_F_6_N_4_O_5_Na [M + Na]^+^ 557.1236, found 557.1232.

*(6*S*,12*S*)-*N*,*N*′-Bis[(2*S*)-1-hydroxy-3-methylbutan-2-yl]-6,12-bis(trifluoromethyl)-5,6,11,12-tetrahydro-6,12-epoxydibenzo[*b*,*f*][1,5]diazocine-2,8-dicarboxamide (**8b**) and (6*R*,12*R*)-*N*,*N*′-Bis[(2*S*)-1-hydroxy-3-methylbutan-2-yl]-6,12-bis(trifluoromethyl)-5,6,11,12-tetrahydro-6,12-epoxydibenzo[*b*,*f*][1,5]diazocine-2,8-dicarboxamide (**8b′**)*. To a solution of epoxydibenzo[*b*,*f*][1,5]diazocine **2h** (100.0 mg, 0.210 mmol) in THF (2 mL) were added (*S*)-(+)-2-amino-3-methyl-1-butanol (l-Valinol, **7b**) (64.9 mg, 0.630 mmol) and TBD (17.5 mg, 0.126 mmol, 60 mol %), and the resulting reaction mixture was heated at 60 °C for 48 h. Then the reaction was quenched with solid NH_4_Cl (33.7 g, 0.630 mmol), and the mixture diluted with MeOH (15 mL) and adsorbed on silica. The residue was chromatographed on silica (5–10% MeOH/DCM) to give a white solid (90.4 mg, 70%). The diastereomeric ratio was estimated on the basis of ^19^F NMR to be 55:45: ^1^H NMR (400 MHz, CD_3_OD) δ 7.49 (d, *J* = 8.4 Hz, 2H), 7.28–7.19 (m, 4H), 3.90–3.79 (m, 2H), 3.71–3.56 (m, 4H), 2.00–1.85 (m, 2H), 1.04–0.82 [m, 12H, HOCH_2_CHCH(C*H*_3_)_2_]; ^13^C{^1^H} NMR (100 MHz, CD_3_OD) δ 170.1 (*C*O_2_R), 143.4, 138.3, 138.2, 126.5, 124.3 (q, *J*_CF_ = 282.0 Hz), 123.8, 119.7, 119.6, 117.6 (×2), 84.4 (q, *J*_CF_ = 32.0 Hz), 63.1, 58.8, 30.2 (×2), 20.1, 19.2; ^19^F NMR (376 MHz, CD_3_OD) δ −80.4, −80.5; HRMS (ESI) *m*/*z* calcd for C_28_H_32_F_6_N_4_O_5_Na [M + Na]^+^ 641.2175, found 641.2184.

Diethyl (2*E*,2′*E*)-4,4′-{[(6S*,12S*)-6,12-Bis(trifluoromethyl)-5,6,11,12-tetrahydro-6,12-epoxydibenzo[*b*,*f*][1,5]diazocine-2,8-diyl]bis(oxy)}bisbut-2-enoate (**9**). The flask was charged with epoxydibenzodiazocine **2l** (55.0 mg, 0.116 mmol, 1 equiv), a second-generation Grubbs catalyst (4.9 mg, 0.006 mmol, 5 mol %), and anhydrous DCE (2.3 mL), and a gentle stream of argon was bubbled for 30 min. Then ethyl acrylate (76 μL, 0.699 mmol, 6 equiv) was added, and the reaction mixture was refluxed for 5 h (TLC analysis indicated a partial consumption of the substrate). Then another portion of a second-generation Grubbs catalyst (4.9 mg, 0.006 mmol, 5 mol %) was added, and the mixture was heated at reflux for 19 h. The solvent was evaporated, and the residue was chromatographed on silica (10–15% EtOAc/hexanes) to give a violet oil (35.1 mg, 49%): ^1^H NMR (400 MHz, CDCl_3_) δ 7.04–6.96 [m, 4H, (2 × Ar*H*, 2 × C*H*=CHCO_2_Et)], 6.84–6.79 (m, 4H), 6.14 (dt, *J* = 15.8, 2.0 Hz, 2H, CH=C*H*CO_2_Et), 4.62–4.54 (m, 4H, ArOC*H*_2_), 4.20 (q, *J* = 7.1 Hz, 4H, OC*H*_2_CH_3_), 1.29 (t, *J* = 7.1 Hz, 6H, OCH_2_C*H*_3_); ^13^C{^1^H} NMR (100 MHz, CDCl_3_) δ 166.1, 153.8, 142.1, 134.0, 122.9 (q, *J*_CF_ = 282.6 Hz), 122.0, 121.8, 121.5, 118.0, 111.8 (q, *J*_CF_ = 3.1 Hz), 83.9 (q, *J*_CF_ = 31.3 Hz), 67.1, 60.7, 14.3; ^19^F NMR (376 MHz, CDCl_3_) δ −79.1; HRMS (ESI) *m*/*z* calcd for C_28_H_26_F_6_N_2_O_7_Na [M + Na]^+^ 639.1542, found 639.1536.

(6S*,12S*)-2,8-Bis[(1-benzyl-1*H*-1,2,3-triazol-4-yl)methoxy]-6,12-bis(trifluoromethyl)-5,6,11,12-tetrahydro-6,12-epoxydibenzo[*b*,*f*][1,5]diazocine (**10**). The contents of a 4 mL screw-cap vial charged with epoxydibenzodiazocine **2k** (50.0 mg, 0.182 mmol, 1 equiv), benzyl azide (120.9 mg, 0.908 mmol, 5 equiv), THF (2.5 mL), CuI (2.1 mg, 0.011 mmol, 6 mol %), and Et_3_N (127 μL, 0.908 mmol, 5 equiv) were stirred for 22 h at rt. The solvent was evaporated *in vacuo*. The residue was chromatographed on silica (30–40% EtOAc/toluene) to give a white solid (80.2 mg, 60%): mp >162 °C dec (MeOH/DCM); ^1^H NMR (400 MHz, CD_3_OD) δ 7.87 (s, 2H), 7.35–7.17 (m, 10H), 7.03 (br s, 2H), 6.81 (dd, *J* = 8.9, 2.7 Hz, 2H), 6.74 (d, *J* = 8.8 Hz, 2H), 5.50 (s, 4H, C*H*_2_), 4.99 (s, 4H, C*H*_2_); ^13^C{^1^H} NMR (100 MHz, CD_3_OD) δ 153.8, 145.3, 137.1, 136.5, 130.0, 129.6, 129.0, 125.2, 124.4 (*J*_CF_ = 282.1 Hz), 122.4, 120.5, 119.0, 112.7 (*J*_CF_ = 3.2 Hz), 84.8 (*J*_CF_ = 31.6 Hz), 63.0, 54.9; ^19^F NMR (376 MHz, CD_3_OD) δ −80.4; HRMS (ESI) *m*/*z* calcd for C_36_H_28_F_6_N_8_O_3_Na [M + Na]^+^ 757.2078, found 757.2086.

### Configuration Assignment and Stability Investigations

ECD spectra at room temperature were measured in acetonitrile using a Jasco J-815 spectrometer in the range of 180–400 nm (*c* = 2.9 × 10^–4^ M) in quartz cells with a path length of 0.1 or 1 cm. The following measurement parameters were used: a scanning speed of 100 nm/min, a step size of 0.2 nm, a bandwidth of 1 nm, a response time of 0.5 s, and an accumulation of three scans. The spectra were background-corrected using acetonitrile.

ECD spectra at variable temperatures were measured in decalin (*c* = 2.75 × 10^–4^ M) using a Jasco J-715 spectrometer equipped with a dedicated variable-temperature transmission cell holder from Specac. The spectra of (+)- and (−)-**2a** were recorded from 190 to 400 nm in a quartz cell with a path length of 0.1 cm. Baseline correction was achieved by subtracting the spectrum of decalin obtained under the same conditions. All spectra were normalized to Δε (cubic decimeters per mole per centimeter) using volume correction for decalin.

VCD spectra of enantiomers (+)- and (−)-**2a** were recorded simultaneously with IR spectra by a Chiral*IR*-2X instrument from BioTools (Jupiter, FL) at a resolution of 4 cm^–1^ in the range of 2000–900 cm^–1^ using CD_3_CN as a solvent. A solution with a concentration ∼0.2 M was measured in a BaF_2_ cell with a path length of 100 μm. Spectra were recorded for approximately 3 h to improve the signal-to-noise ratio. Baseline correction was achieved by subtracting the spectrum of a solvent recorded under the same conditions.

### Computational Details

A conformational search was carried out at the molecular mechanics level using the MMFF94s force field within 10 kcal/mol for (+)-**2a**. Next, the found structure was submitted for DFT optimization using Gaussian16^[1]^ at the ωB97X-D/6-311+G(d,p) level of theory applying PCM for CH_3_CN.

The same level of theory was used for VCD and IR simulations. The VCD simulated spectrum was converted to Lorentzian bands with an 8 cm^–1^ half-width and was scaled by 0.982 (the best scaling factor, giving the best agreement between the experimental and simulated spectra).

TDDFT calculations of the final ECD spectrum were carried out using the CAM-B3LYP functionals with the def2-TZVP basis set and PCM model for CH_3_CN. The calculations at the B3LYP/def2-TZVP/PCM and ωB97X-D/def2-TZVP/PCM levels yielded consistent results. Rotatory strengths were calculated using both length and velocity representations. The differences between the length and velocity of the calculated values of the rotatory strengths were <3%, and for this reason, only the velocity representations (*R*_vel_) were taken into account. The UV and ECD spectra are simulated by overlapping Gaussian functions for 40 electronic transitions using bands with a 0.3 eV exponential half-width and red-shifted by 13 nm (UV correction).

## Supporting Information Available

Copies of NMR spectra for all new compounds, chromatograms for separation of enantiomers of (+)-**2a**, Cartesian coordinates for (+)-**2a**, and detailed optimization studies of the condensation reaction leading to (+)-**2a** and (+)-**2l** (PDF)

## Accession Codes

CCDC 2055622 contains the supplementary crystallographic data for this paper. These data can be obtained free of charge via www.ccdc.cam.ac.uk/data_request/cif, or by emailing data_request@ccdc.cam.ac.uk, or by contacting The Cambridge Crystallographic Data Centre, 12 Union Road, Cambridge CB2 1EZ, UK; fax: +44 1223 336033.
