# Supplementary material for: Base-Catalyzed, Solvent-Free Synthesis of Rigid V-Shaped Epoxydibenzo[b,f][1,5]diazocines
Source: J Org Chem. 2021 Jun 23;86(13):8955–69. doi: 10.1021/acs.joc.1c00884 (PMC8279491; doi:10.1021/acs.joc.1c00884)
Supplement: Supplementary file 1 — jo1c00884_si_001.pdf [file jo1c00884_si_001.pdf]

Electronic Supplementary Information

for

# Base-catalyzed, solvent-free synthesis of rigid V-shaped epoxydibenzo[*b*,*f*][1,5]diazocines


**Michał Michalak,[a]* Bartosz Bisek,[a] Michał Nowacki[a] and Marcin Górecki[a]**

**[a] Institute of Organic Chemistry, Polish Academy of Sciences**

**Kasprzaka 44/52, 01-224 Warsaw, Poland**

**michal.michalak@icho.edu.pl**


## Table of contents


1. **Structure of aminophenones used in this studies. ........ 2**
2. **Optimization studies for the synthesis of 2a ........ 2**
3. **Chromatogram of the autocondesation of 1a leading to (+)-2a. ........ 3**
4. **Configuration assignment and stability investigations. ........ 4**
5. **Cartesian coordinates for compound (+)-2a. ........ 4**
6. **Separation of enantiomers on preparative chiral column. ........ 5**
7. **X-ray data of 2o. ........ 7**
8. **Synthetic routes to a selected aminophenones 1. ........ 9**
9. **Copies of spectra ........ 11**

## 1. Structure of aminophenones used in this studies.

Figure S1

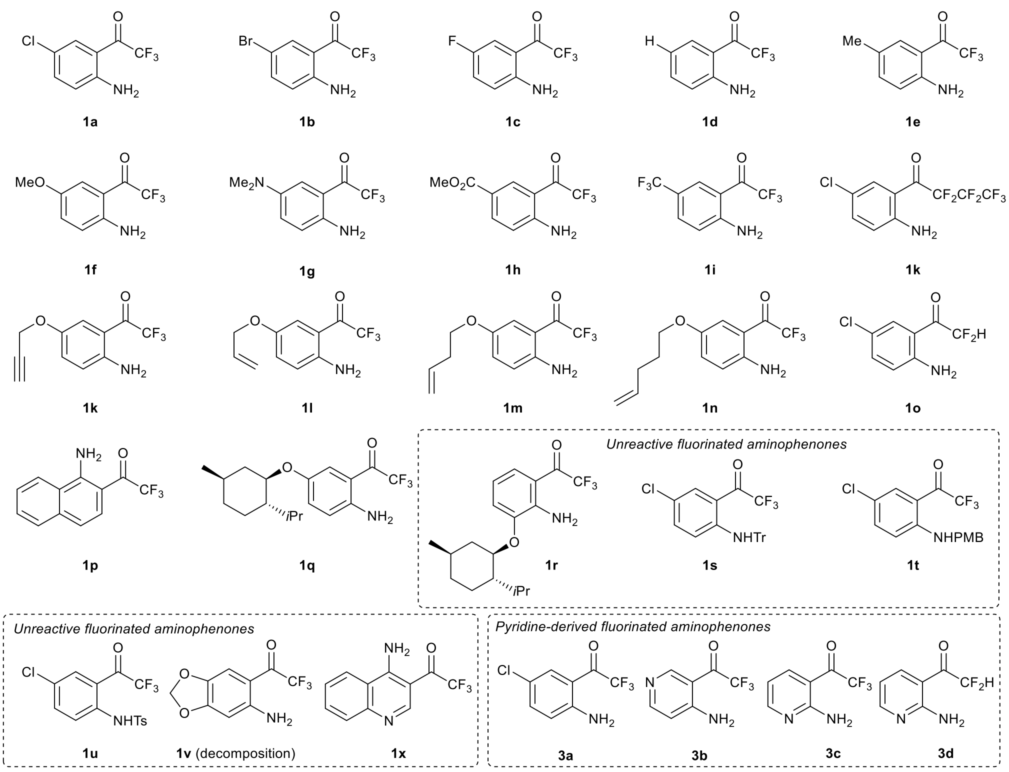


## 2. Optimization studies for the synthesis of 2a

Influence of solvent and substituent attached to nitrogen for the auto-condensation of aminophenone **1**

Scheme S1

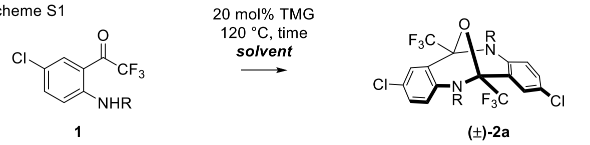


Table 1A.Influence of solvent for the formation of dibenzo[*b*,*f*][1,5]diazocine **(±)-2a** catalyzed by TMG

| | | | 3h | | 16h | |
|---|---|---|---|---|---|---|
| Entry | Solvent | R | Conversion (%)[a] | Yield (%)[b] | Conversion (%)[a] | Yield (%)[b] |
| 1 | DMF | H | 90 | 78 | 94 | 87 |
| 2 | DMSO | H | 93 | 84 | 97 | 84 |
| 3 | Py | H | 83 | 77 | 90 | 86 |
| 4 | MeCN | H | 86 | 82 | 99 | 87 |
| 5 | TMG | H | 93 | 26 | 98 | 18 |
| 6 | $(CH_2OH)_2$ | H | 95 | 70 | 98 | 73 |
| 7 | *i*-PrOH | H | 59 | 47 | 98 | 72 |
| 8 | *n*-BuOH | H | 61 | 52 | 84 | 29 |
| 9 | water | H | 80 | 64 | 97 | 29 |
| 10 | 1,4-dioxane | H | 45 | 30 | 74 | 65 |
| 11 | DCE | H | 24 | 8 | 37 | 15 |
| 12 | toluene | H | 32 | 18 | 61 | 49 |
| 13 | *n*-heptane | H | 32 | 16 | 44 | 24 |
| 14 | - | H | **99** | **99** | **99** | **98** |
| 15 | - | PMB | n.d | n.d | <5 | <5 |
| 16 | - | Tr | n.d. | n.d. | <5 | <5 |
| 17 | - | Ts | n.d. | n.d. | <5 | <5 |

[a] conversion based on GC; naphtalene as internal standard
[b] yield estimated from calibration curve

Table 1B. Effect of base

| | | | 16h |
|---|---|---|---|
| Entry | Solvent | Base (20 mol%) | Yield (%) |
| 1 | - | $K_2CO_3$ | 16[a] |
| 2 | - | $K_3PO_4$ | 12[a] |
| 3 | - | $Et_3N$ | 32[a] |
| 4 | - | $Et_2$(*i*-Pr)N | 40[a] |
| 5 | - | (*i*-Pr)$_2$NH | 26[a] |
| 6 | - | DBU | <5[a] |
| 7 | - | TBD | <5[a] |
| 8 | - | TBDMe | <5[a] |
| 9 | - | BEMP | decomposition of sub. |
| 10 | - | TMG($^t$Bu) | 17[a] |
| 11 | - | **TMG** | **98**[a] |
| 12 | toluene | TMG | 49[a] (23)[b] |

[a] yield estimated from calibration curve; [b] isolated yield

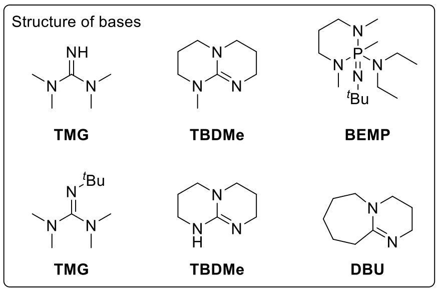

## 3. Chromatogram of the autocondesation of 1a leading to (+)-2a.

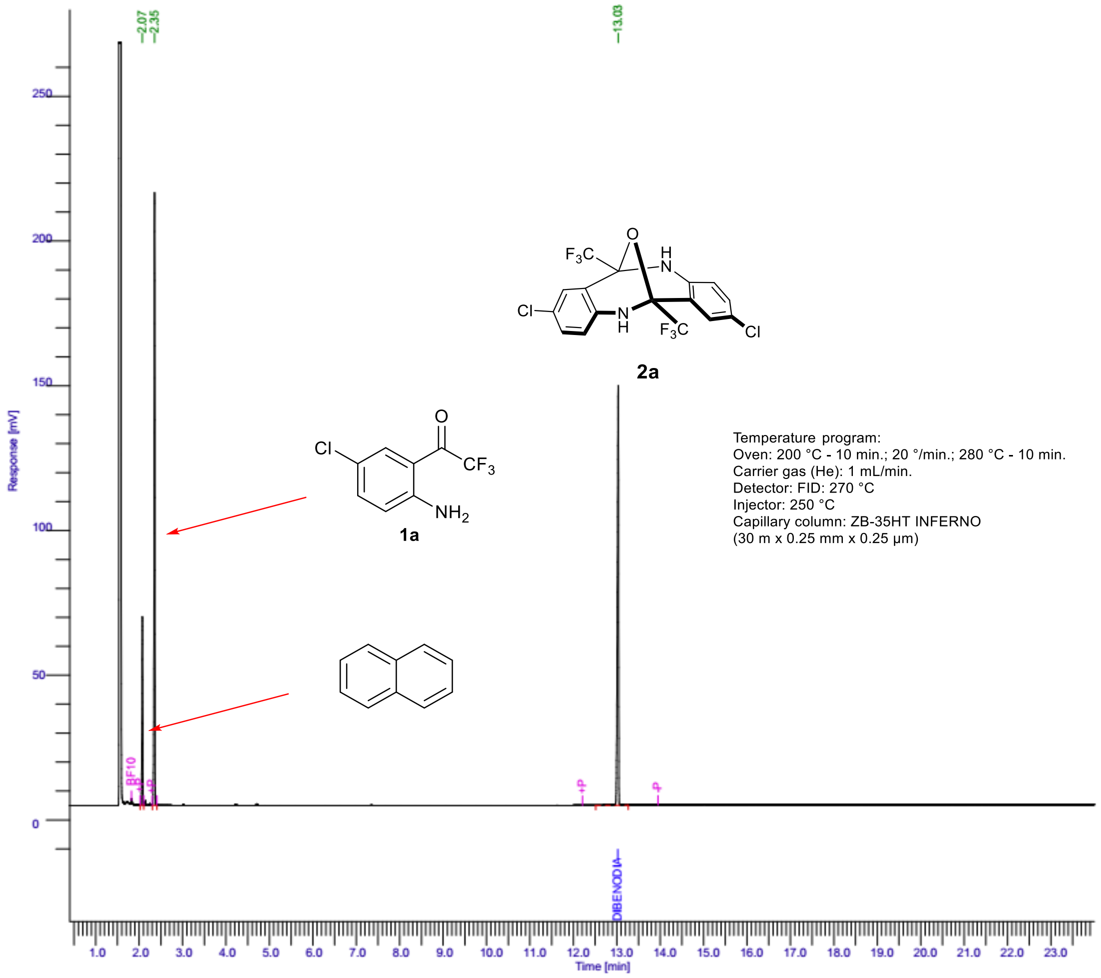


Calibration curve used to determine the yield of formation of (+)-diazocine **2a**

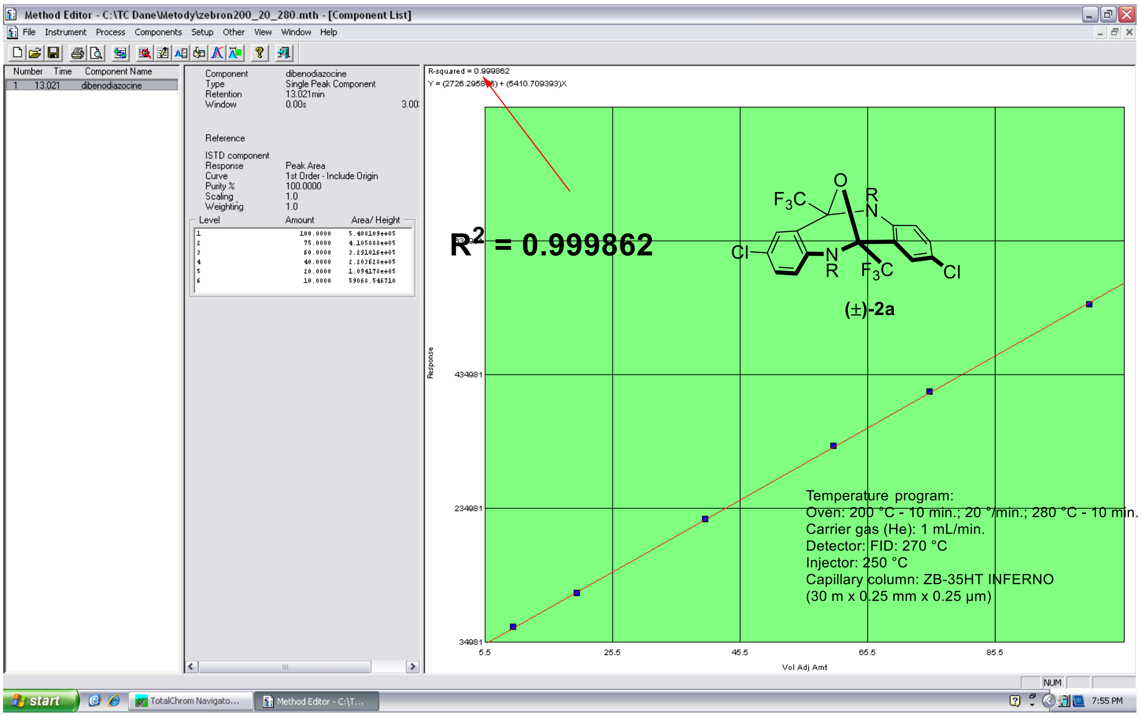

## 4. Configuration assignment and stability investigations.

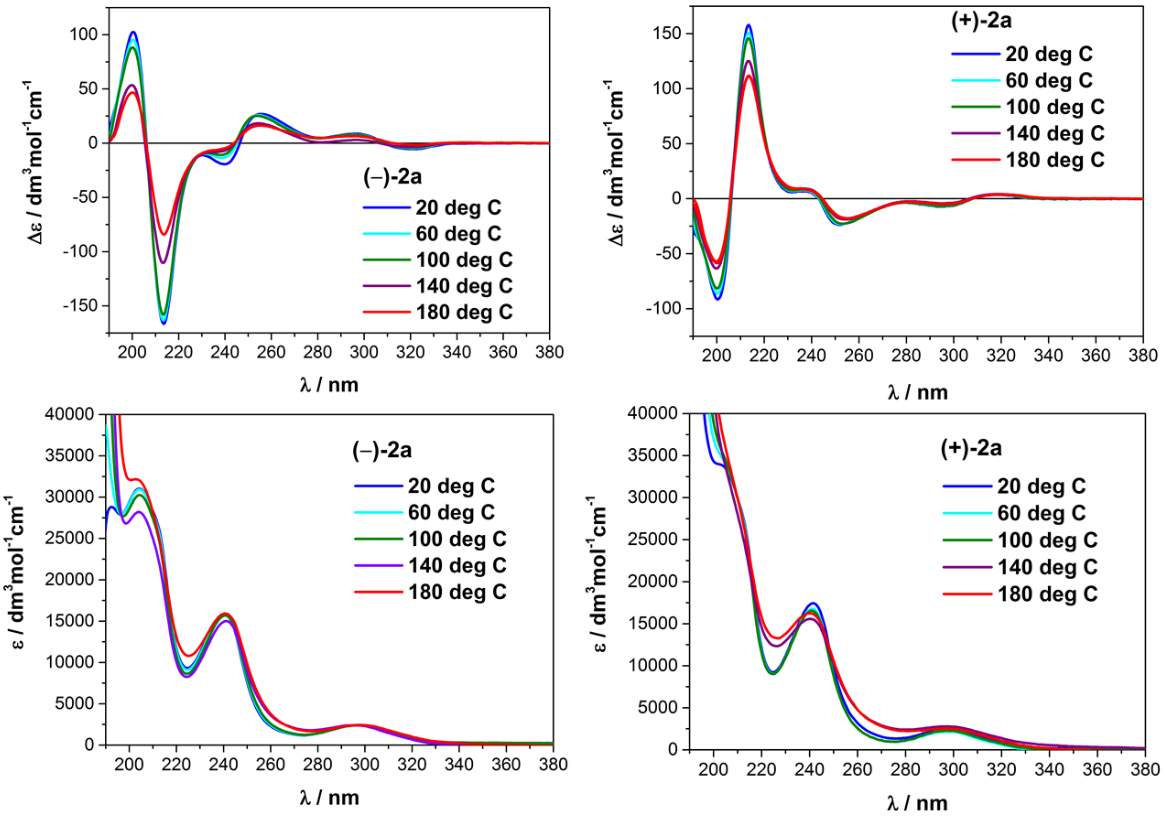


**Fig S2.** Variable temperature ECD/UV spectra of **(+)-2a** and **(-)-2a** measured in decalin.

## 5. Cartesian coordinates for compound (+)-2a.

| | | | |
|---|---|---|---|
| C | 1.570236 | 2.914217 | -1.981697 |
| C | 0.258574 | 3.307567 | -1.745642 |
| C | -0.526599 | 2.647732 | -0.819465 |
| C | -0.000000 | 1.574756 | -0.103569 |
| C | 1.312257 | 1.151567 | -0.351513 |
| C | 2.089600 | 1.838014 | -1.287256 |
| Cl | -0.412195 | 4.659198 | -2.634813 |
| N | 1.844045 | 0.057414 | 0.337708 |
| C | 0.839303 | -0.804738 | 0.917448 |
| C | -0.839303 | 0.804738 | 0.917448 |
| N | -1.844045 | -0.057414 | 0.337708 |
| C | -0.000000 | -1.574756 | -0.103569 |
| C | -1.312257 | -1.151567 | -0.351513 |
| C | -2.089600 | -1.838014 | -1.287256 |
| C | -1.570236 | -2.914217 | -1.981697 |

| | | | |
|---|---|---|---|
| C | -0.258574 | -3.307567 | -1.745642 |
| C | 0.526599 | -2.647732 | -0.819465 |
| O | 0.000000 | 0.000000 | 1.706495 |
| Cl | 0.412195 | -4.659198 | -2.634813 |
| C | -1.528256 | 1.728676 | 1.949502 |
| F | -2.021341 | 1.031902 | 2.971206 |
| F | -2.551528 | 2.388547 | 1.381700 |
| F | -0.676386 | 2.629817 | 2.439772 |
| C | 1.528256 | -1.728676 | 1.949502 |
| F | 0.676386 | -2.629817 | 2.439772 |
| F | 2.551528 | -2.388547 | 1.381700 |
| F | 2.021341 | -1.031902 | 2.971206 |
| H | 2.178511 | 3.438614 | -2.707648 |
| H | -1.545776 | 2.973267 | -0.657520 |
| H | 3.108077 | 1.517577 | -1.473714 |
| H | 2.577822 | -0.429145 | -0.160185 |
| H | -2.577822 | 0.429145 | -0.160185 |
| H | -3.108077 | -1.517577 | -1.473714 |
| H | -2.178511 | -3.438614 | -2.707648 |
| H | 1.545776 | -2.973267 | -0.657520 |

## 6. Separation of enantiomers on preparative chiral column.

The racemic diazocine (±)**-2a** we separated into enantiomers on semipreparative AD-H (1 cm × 25 cm) column using Combi Flash EzPrep (5% *i*-PrOH/hexane, 5 ml/min.; 254 nm). Each of separated samples contains 1-2 mg of racemic (±)**-2a**, and then enantiomeric purity was analyzed on analytical AD-H column (5% *i*-PrOH/hexane, 1 ml/min/; 254 nm; for representative chromatograms, see below).

05.07.2019 Chromatogram C:\ClarityChrom\DataFiles\Michalak\chiralpak-AD-H\dibenzo_racemat-1-hex-ipa-90-10-254 nm(AD-H)recznierobuklad.PRM Page 2 of 2

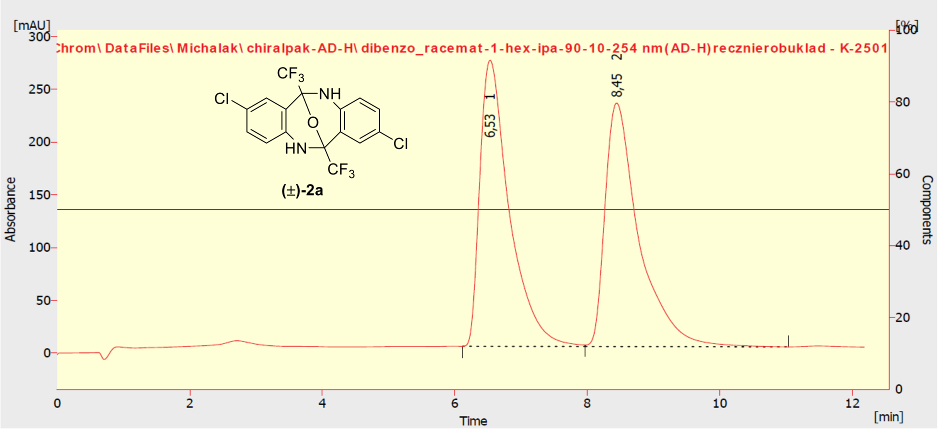


*Result Table (Uncal - C:\ClarityChrom\DataFiles\Michalak\chiralpak-AD-H\dibenzo_racemat-1-hex-ipa-90-10-254 nm(AD-H)recznierobuklad - K-2501: Channel 1)*

| | Reten. Time [min] | Area [mAU.s] | Height [mAU] | Area [%] | Height [%] | W05 [min] | Compound Name |
|---|---|---|---|---|---|---|---|
| 1 | 6,533 | 8392,881 | 271,416 | 50,5 | 54,0 | 0,47 | |
| 2 | 8,450 | 8225,659 | 230,809 | 49,5 | 46,0 | 0,52 | |
| | Total | 16618,540 | 502,225 | 100,0 | 100,0 | | |

05.07.2019 17:Chromatogram C:\ClarityChrom\DataFiles\Michalak\chiralpak-AD-H\dibenzo_1b-1-hex-ipa-90-10-254 nm(AD-H)recznierobuklad.PRM Page 2 of 2

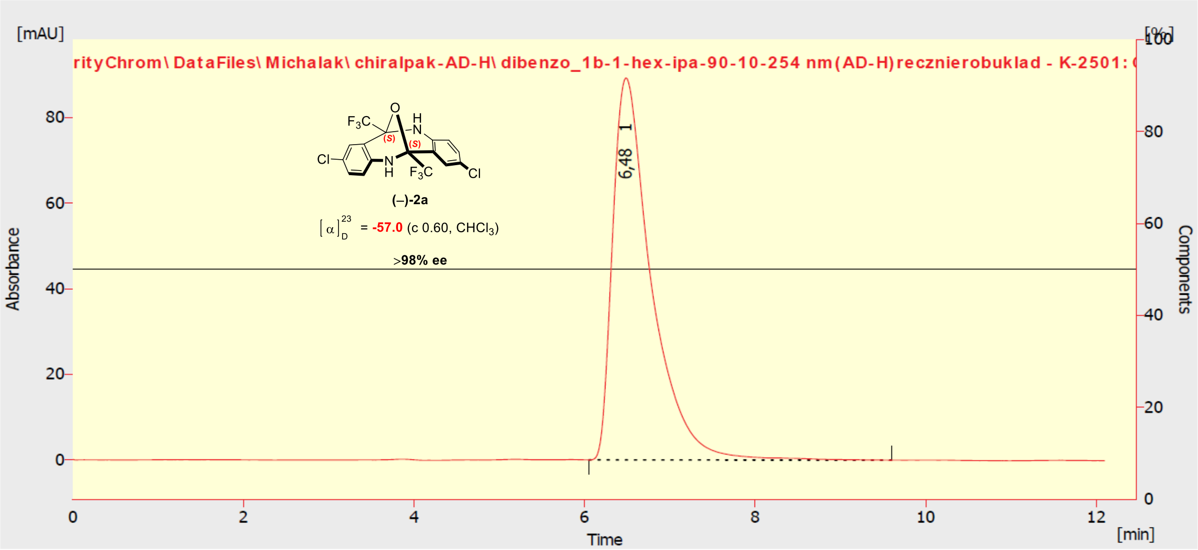


*Result Table (Uncal - C:\ClarityChrom\DataFiles\Michalak\chiralpak-AD-H\dibenzo_1b-1-hex-ipa-90-10-254 nm(AD-H)recznierobuklad - K-2501: Channel 1)*

| | Reten. Time [min] | Area [mAU.s] | Height [mAU] | Area [%] | Height [%] | W05 [min] | Compound Name |
|---|---|---|---|---|---|---|---|
| 1 | 6,483 | 2819,810 | 89,246 | 100,0 | 100,0 | 0,47 | |
| | Total | 2819,810 | 89,246 | 100,0 | 100,0 | | |

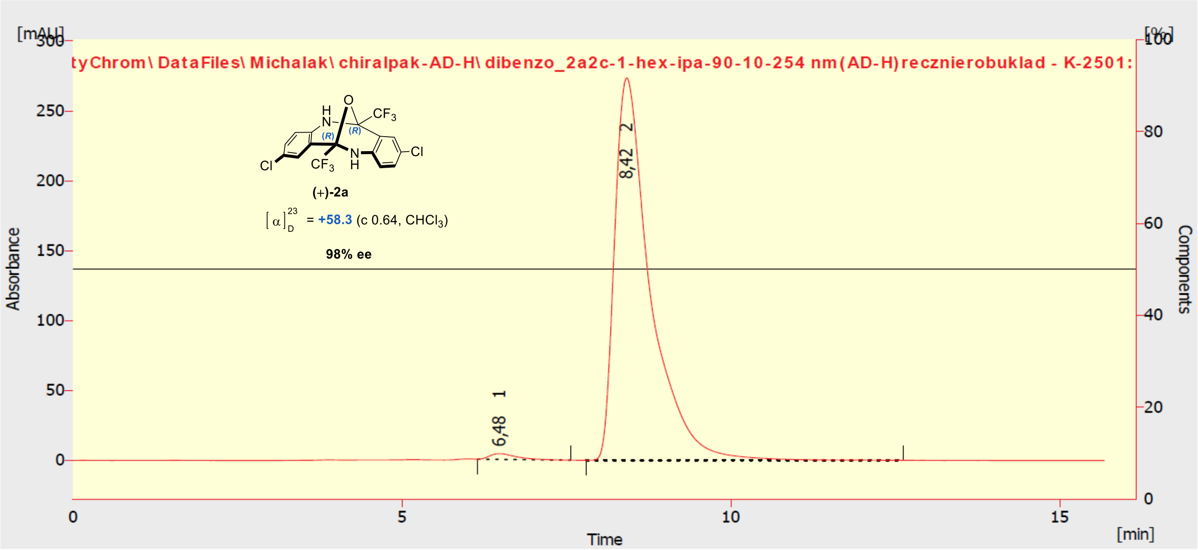


*Result Table (Uncal - C:\ClarityChrom\DataFiles\Michalak\chiralpak-AD-H\dibenzo_2a2c-1-hex-ipa-90-10-254 nm(AD-H)recznierobuklad - K-2501: Channel 1)*

| | Reten. Time [min] | Area [mAU.s] | Height [mAU] | Area [%] | Height [%] | W05 [min] | Compound Name |
|---|---|---|---|---|---|---|---|
| 1 | 6,483 | 117,107 | 4,147 | 1,1 | 1,5 | 0,43 | |
| 2 | 8,417 | 10321,295 | 273,497 | 98,9 | 98,5 | 0,53 | |
| | Total | 10438,402 | 277,644 | 100,0 | 100,0 | | |

## 7. X-ray data of 2o.

CCDC 2055622 contains the supplementary crystallographic data for this paper. This data can be obtained free of charge from The Cambridge Crystallographic Data Centre. Crystals of **2c** suitable for X-ray diffraction were grown by slow diffusion of *n*-pentane into sat. solution of **2c** in DCM. Diffraction data were collected on P14 beamline of the PETRA III synchrotron, Hmburg, Germany. Data was processed with XDS and the structure was solved and refined with SHELX. The structure was refined with anisotropic displacement parameters for all non-hydrogen atoms. All hydrogen atoms were included at geometrically predicted coordinates.

Table S1. Crystalographic data for epoxydiazocine **2c**.

| Crystal data for **2c** | |
|---|---|
| Empirical formula | $C_{16}H_{10}Cl_2F_4N_2O$ |
| Formula weight | 393.16 |
| Temperature (K) | 100 |
| Wavelength (Å) | 0.7293 |
| Crystal system | monoclinic |
| Space group | $P2_1/a$ |
| Unit cell dimensions a, b, c (Å) α, β, γ (°) | 9.0100(4), 9.0800(5), 18.480(2), 90, 93.92, 90 |
| Unit cell volume (Å$^3$) | 1508.3(2) |

| | |
|---|---|
| Z | 4 |
| Calculated density (g/cm$^3$) | 1.731 |
| Absorption coefficient (mm$^{-1}$) | 0.487 |
| F(000) | 792 |
| θ range for data collection (°) | 29.270 - 1.133 |
| Index ranges | -12 < h < 12; -11 < k < 11; -24 < l < 24 |
| Reflections collected | 23449 |
| Independent reflections | 3698 ($R_{int}$ = 0.0475) |
| Completeness to θ max | 0.971 |
| Final R indices [>2σ(I)] | 0.0301 |
| R indices [all data] | 0.0327 |
| Goodness-of-fit | 1.175 |
| Largest diff. peak and hole (e/Å$^3$) | 0.559 / -0.357 |

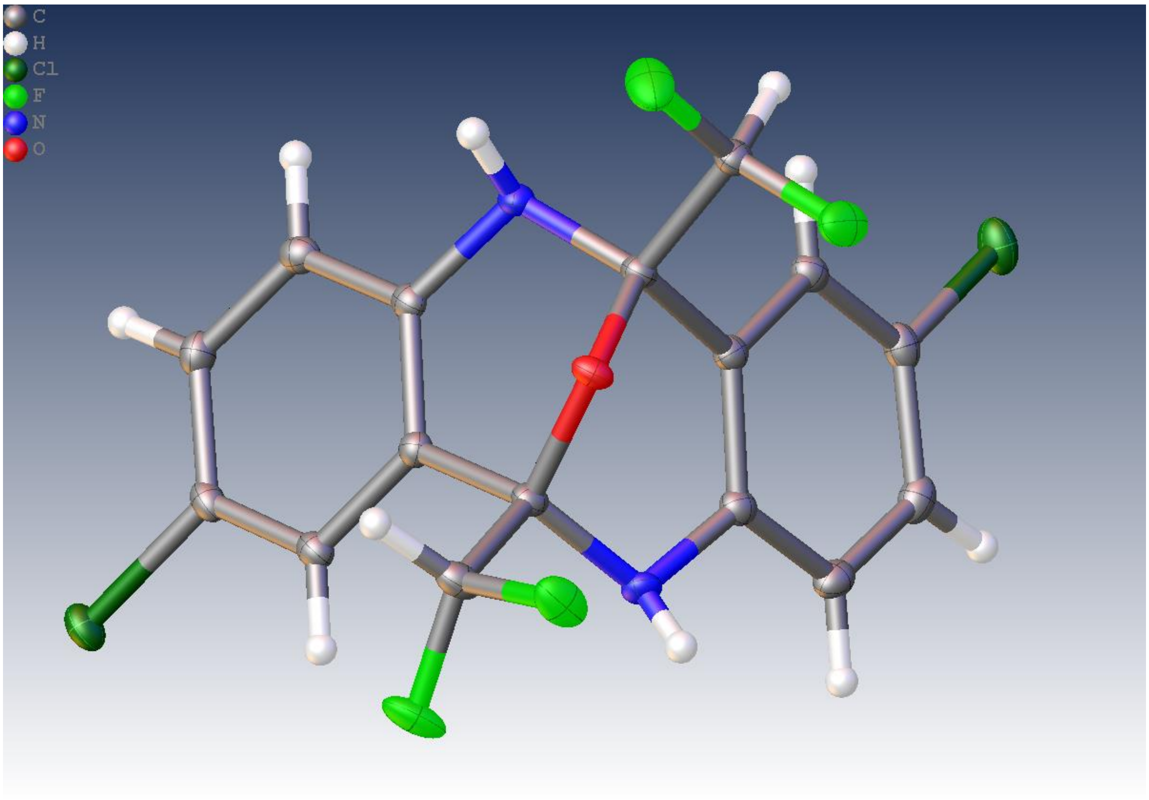


**Fig S3.** ORTEP representation of the X-ray structure of **X** with thermal ellipsoids shown at 50% probability

## 8. Synthetic routes to a selected aminophenones 1.

Scheme S2. *Synthetic route to aminophenone **1l***

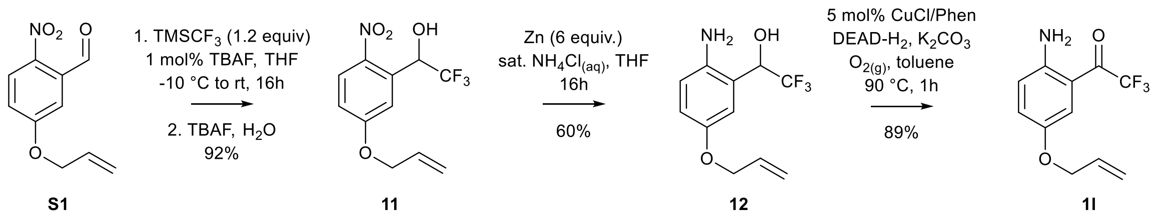


Scheme S3. *Synthetic route to aminophenone **1m***

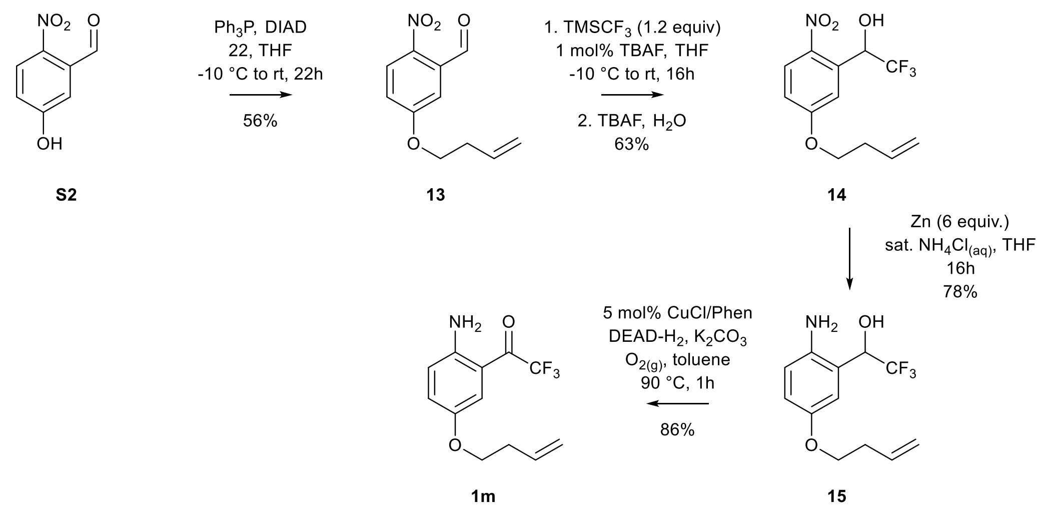


Scheme S4. *Synthetic route to aminophenone **1n***

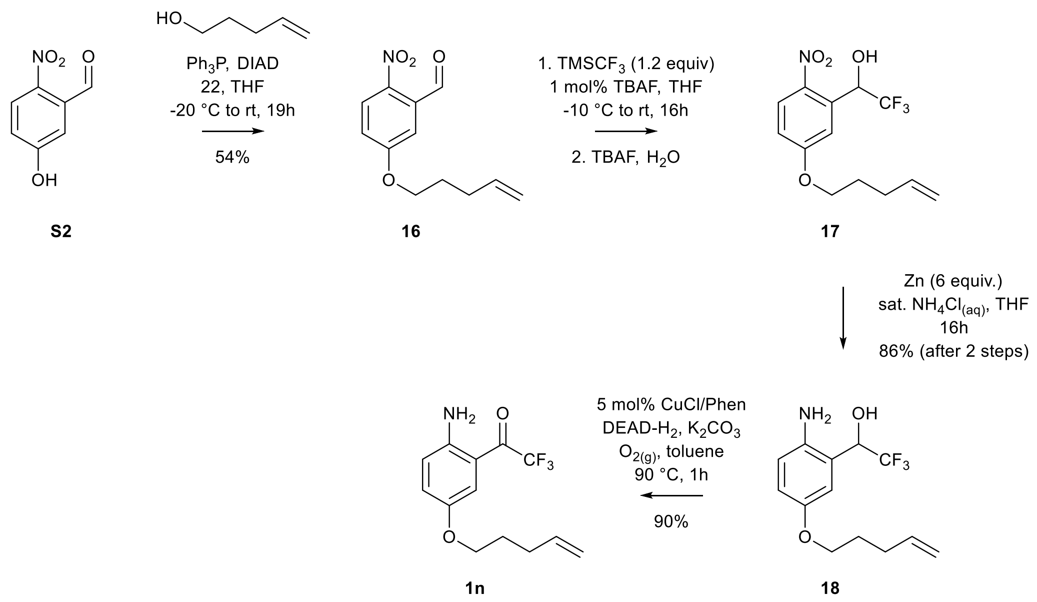

Scheme S5. *Synthetic route to aminophenone **1k***

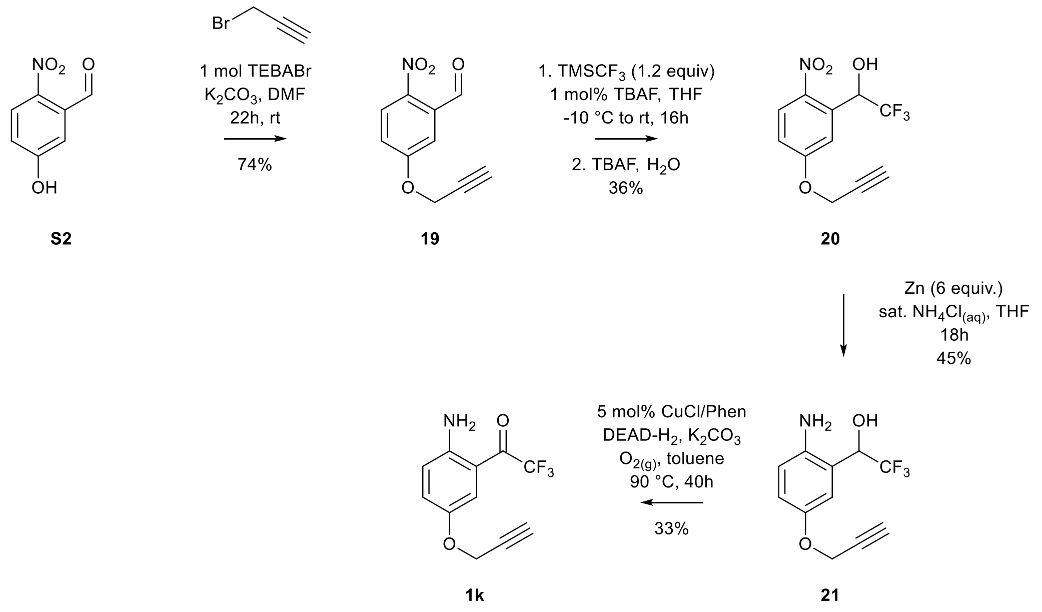


Scheme S6. *Synthetic route to aminophenone **28***

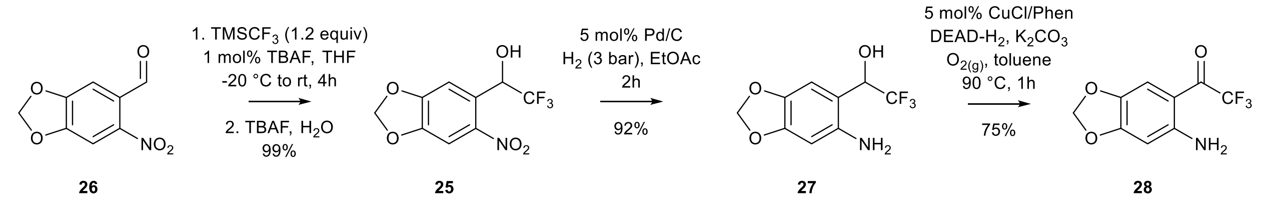


Scheme S7. *Synthetic route to aminophenone **28***

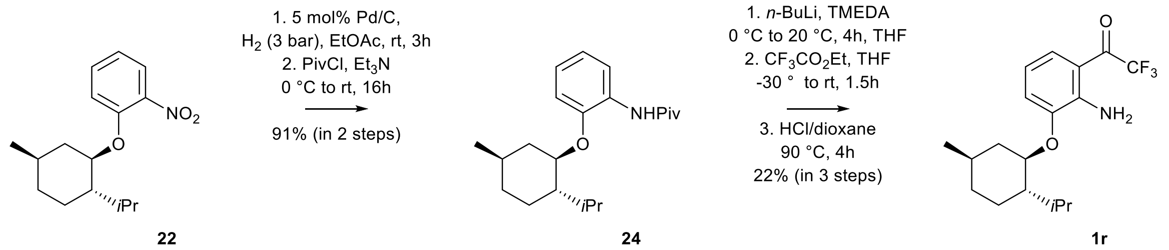

## 9. Copies of NMR spectra

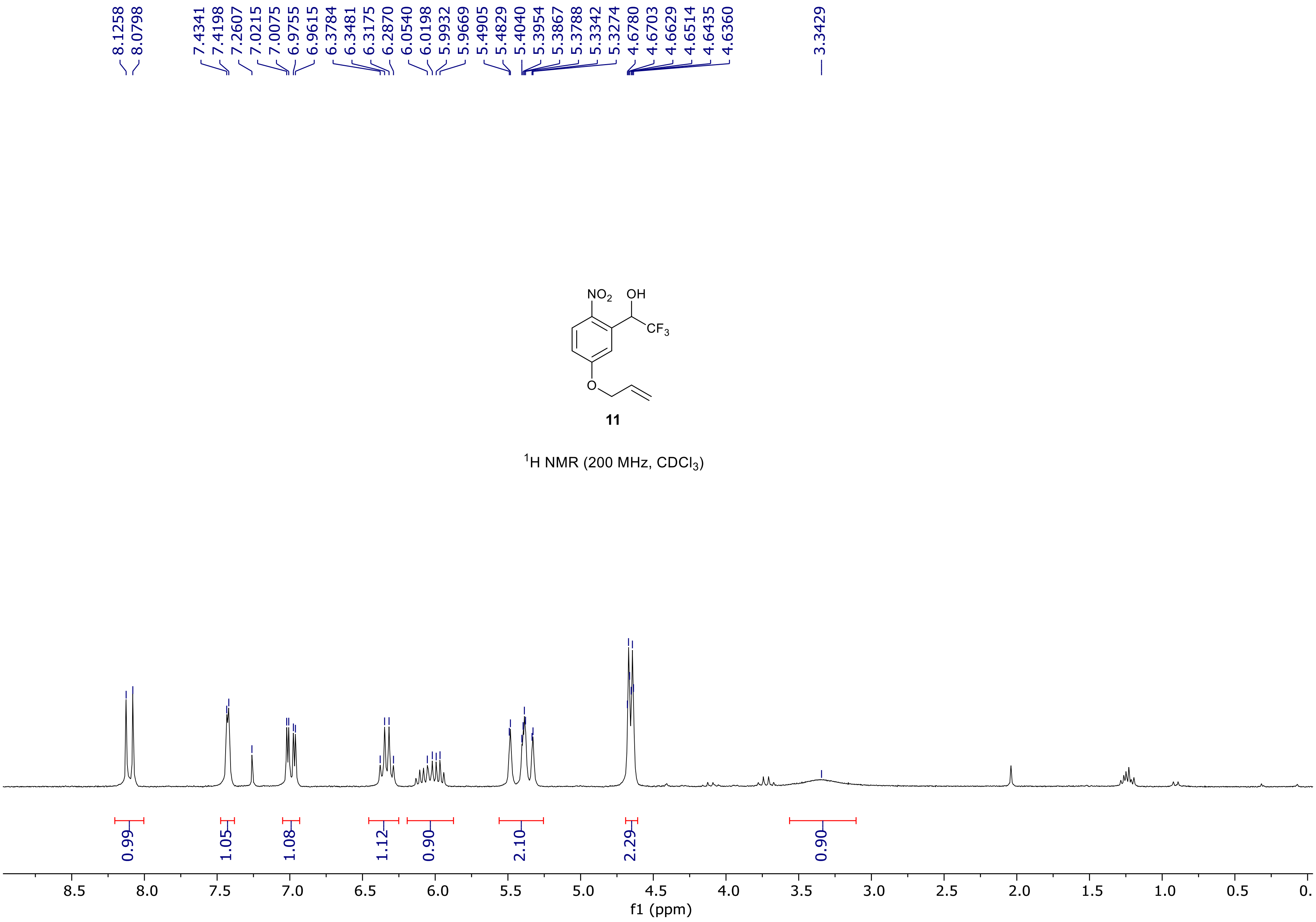
8.1258
8.0798
7.4341
7.4198
7.2607
7.0215
7.0075
6.9755
6.9615
6.3784
6.3481
6.3175
6.2870
6.0540
6.0198
5.9932
5.9669
5.4905
5.4829
5.4040
5.3954
5.3867
5.3788
5.3342
5.3274
4.6780
4.6703
4.6629
4.6514
4.6435
4.6360
3.3429
NO2
OH
CF3
O
11
1H NMR (200 MHz, CDCl3)
0.99
1.05
1.08
1.12
0.90
2.10
2.29
0.90
8.5
8.0
7.5
7.0
6.5
6.0
5.5
5.0
4.5
4.0
3.5
3.0
2.5
2.0
1.5
1.0
0.5
0.
f1 (ppm)

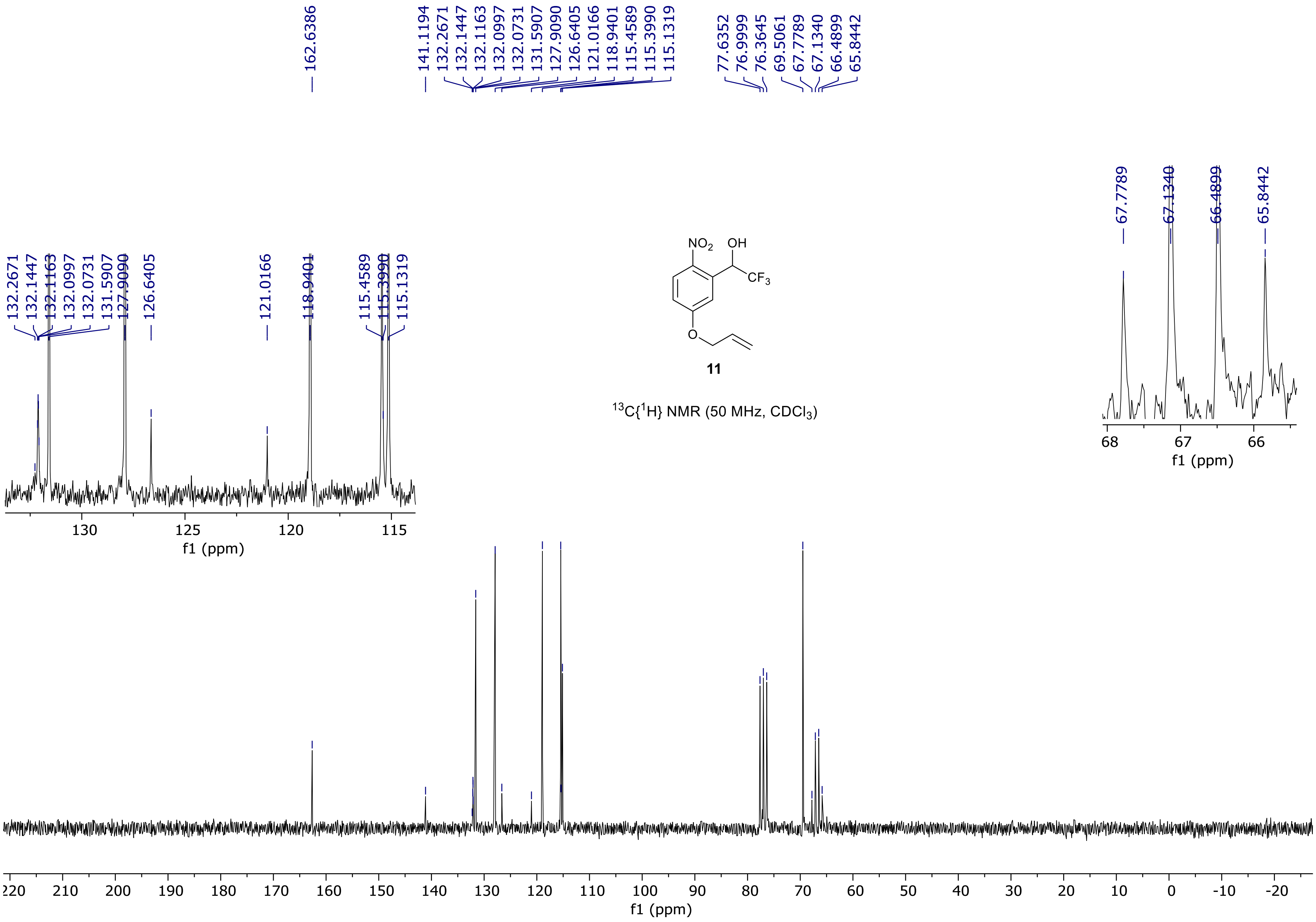
162.6386
141.1194
132.2671
132.1447
132.1163
132.0997
132.0731
131.5907
127.9090
126.6405
121.0166
118.9401
115.4589
115.3990
115.1319
77.6352
76.9999
76.3645
69.5061
67.7789
67.1340
66.4899
65.8442
NO2
OH
CF3
O
11
13C{1H} NMR (50 MHz, CDCl3)
f1 (ppm)

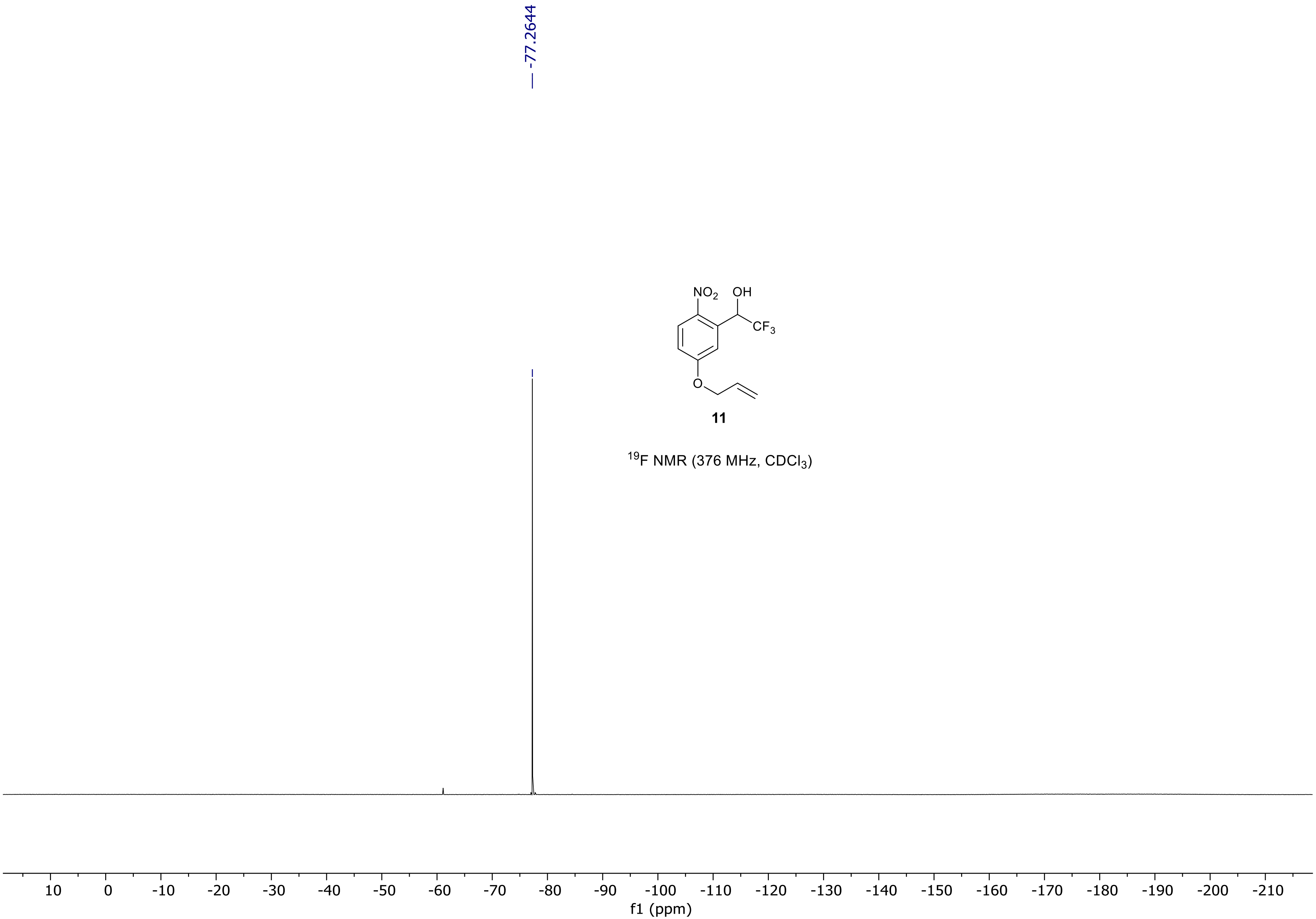
-77.2644
$NO_2$
OH
$CF_3$
O
11
$^{19}F$ NMR (376 MHz, $CDCl_3$)
10 0 -10 -20 -30 -40 -50 -60 -70 -80 -90 -100 -110 -120 -130 -140 -150 -160 -170 -180 -190 -200 -210
f1 (ppm)

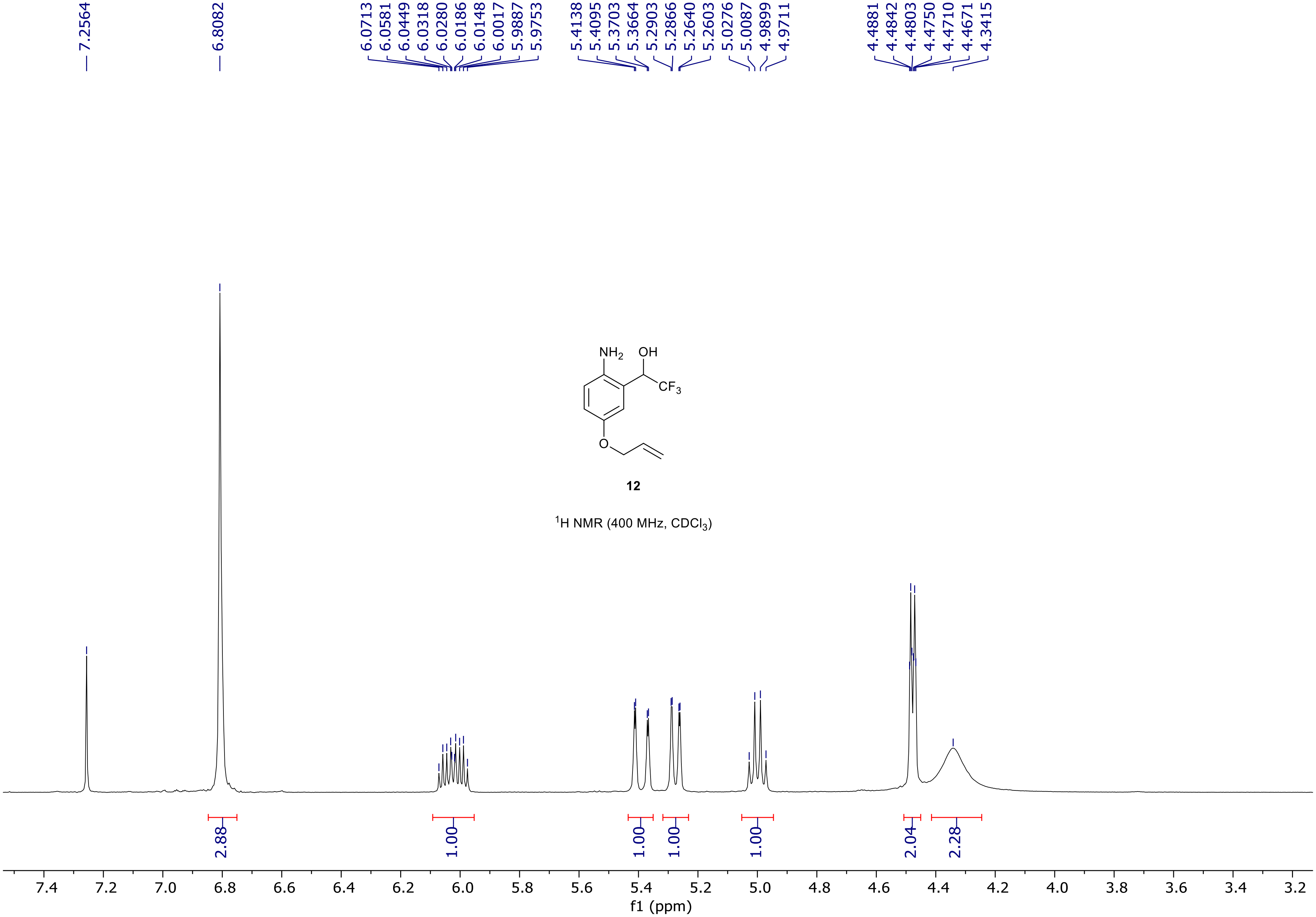
7.2564
6.8082
6.0713
6.0581
6.0449
6.0318
6.0280
6.0186
6.0148
6.0017
5.9887
5.9753
5.4138
5.4095
5.3703
5.3664
5.2903
5.2866
5.2640
5.2603
5.0276
5.0087
4.9899
4.9711
4.4881
4.4842
4.4803
4.4750
4.4710
4.4671
4.3415
NH2
OH
CF3
O
12
1H NMR (400 MHz, CDCl3)
2.88
1.00
1.00
1.00
1.00
2.04
2.28
7.4
7.2
7.0
6.8
6.6
6.4
6.2
6.0
5.8
5.6
5.4
5.2
5.0
4.8
4.6
4.4
4.2
4.0
3.8
3.6
3.4
3.2
f1 (ppm)

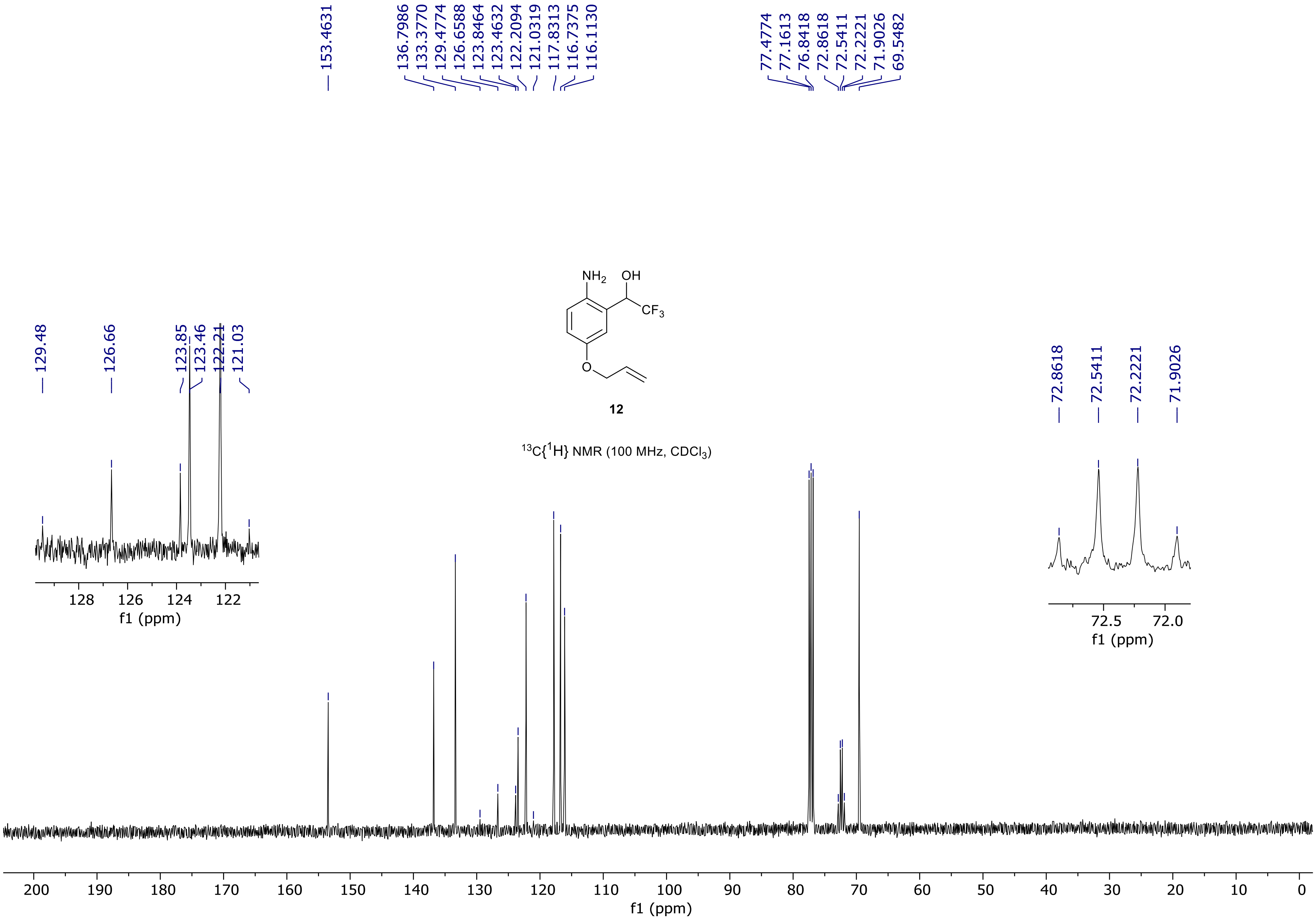
153.4631
136.7986
133.3770
129.4774
126.6588
123.8464
123.4632
122.2094
121.0319
117.8313
116.7375
116.1130
77.4774
77.1613
76.8418
72.8618
72.5411
72.2221
71.9026
69.5482
NH2
OH
CF3
O
12
13C{1H} NMR (100 MHz, CDCl3)
129.48
126.66
123.85
123.46
122.21
121.03
128
126
124
122
f1 (ppm)
72.8618
72.5411
72.2221
71.9026
72.5
72.0
f1 (ppm)
200
190
180
170
160
150
140
130
120
110
100
90
80
70
60
50
40
30
20
10
0
f1 (ppm)

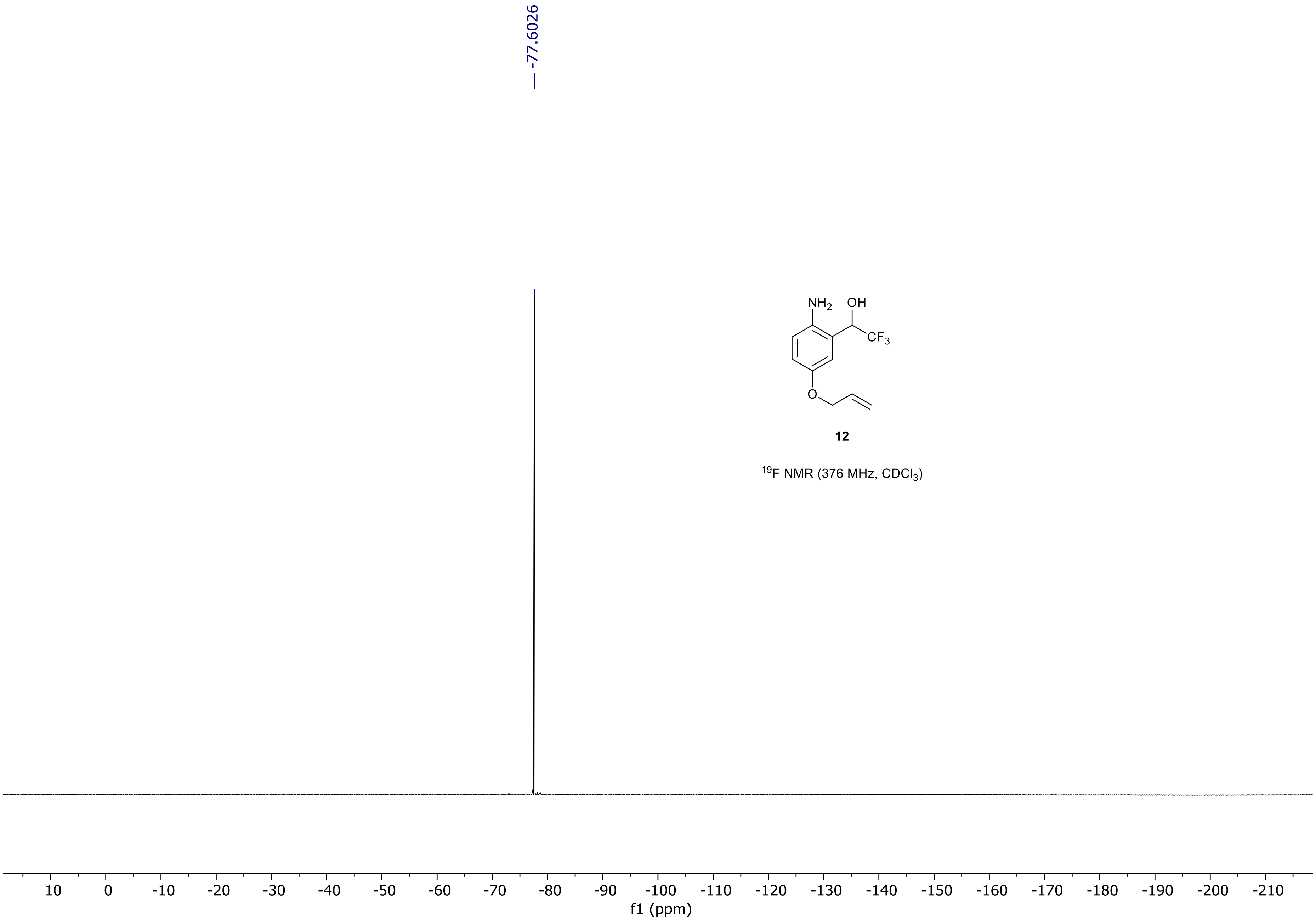
-77.6026
NH2
OH
CF3
O
12
19F NMR (376 MHz, CDCl3)
10 0 -10 -20 -30 -40 -50 -60 -70 -80 -90 -100 -110 -120 -130 -140 -150 -160 -170 -180 -190 -200 -210
f1 (ppm)

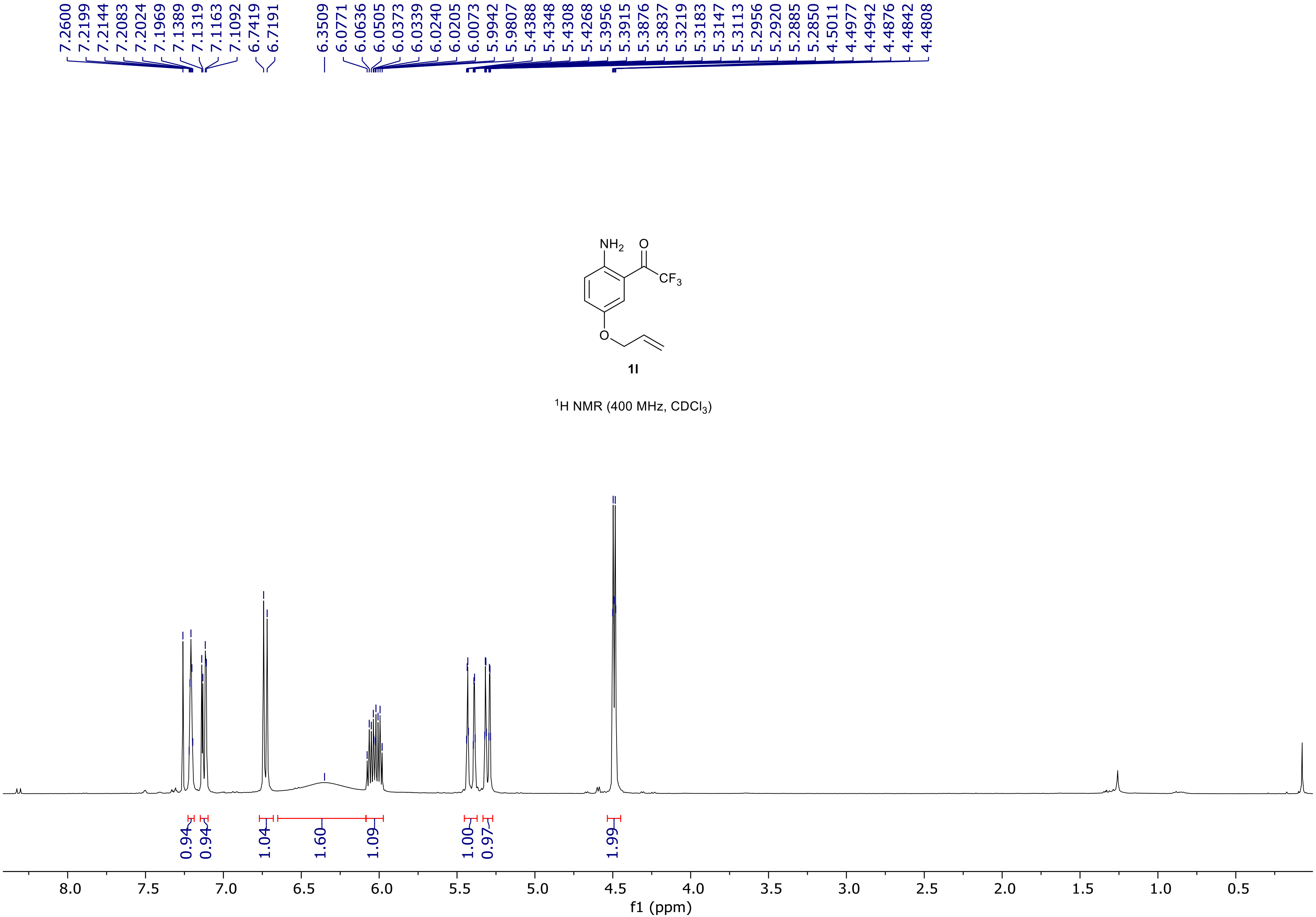
7.2600
7.2199
7.2144
7.2083
7.2024
7.1969
7.1389
7.1319
7.1163
7.1092
6.7419
6.7191
6.3509
6.0771
6.0636
6.0505
6.0373
6.0339
6.0240
6.0205
6.0073
5.9942
5.9807
5.4388
5.4348
5.4308
5.4268
5.3956
5.3915
5.3876
5.3837
5.3219
5.3183
5.3147
5.3113
5.2956
5.2920
5.2885
5.2850
4.5011
4.4977
4.4942
4.4876
4.4842
4.4808
NH2
O
CF3
O
1l
1H NMR (400 MHz, CDCl3)
0.94
0.94
1.04
1.60
1.09
1.00
0.97
1.99
8.0
7.5
7.0
6.5
6.0
5.5
5.0
4.5
4.0
3.5
3.0
2.5
2.0
1.5
1.0
0.5
f1 (ppm)

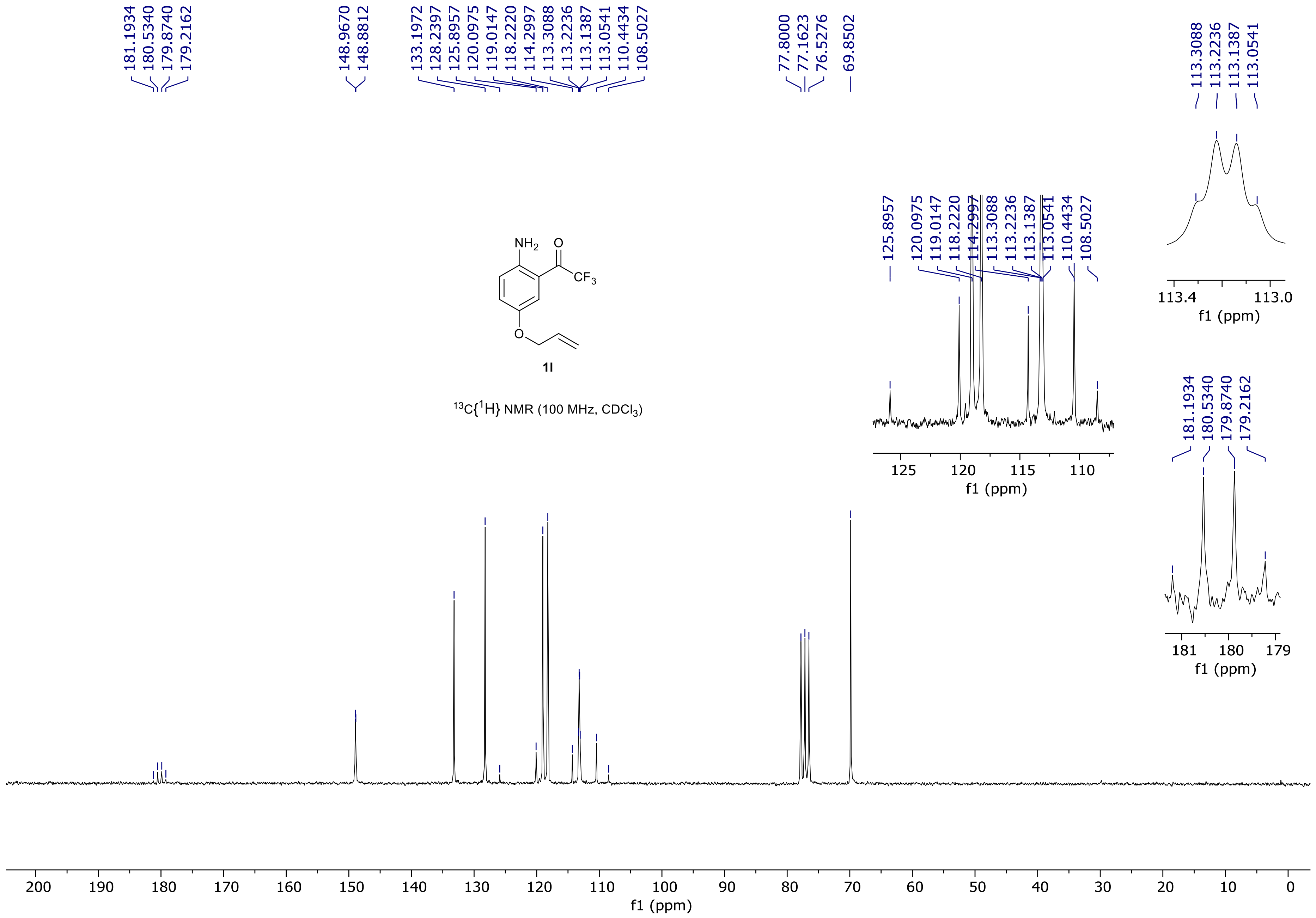

181.1934
180.5340
179.8740
179.2162
148.9670
148.8812
133.1972
128.2397
125.8957
120.0975
119.0147
118.2220
114.2997
113.3088
113.2236
113.1387
113.0541
110.4434
108.5027
77.8000
77.1623
76.5276
69.8502
NH2
O
CF3
1l
$^{13}C\{^1H\}$ NMR (100 MHz, CDCl3)
113.4
113.0
f1 (ppm)
125
120
115
110
f1 (ppm)
181
180
179
f1 (ppm)
200 190 180 170 160 150 140 130 120 110 100 90 80 70 60 50 40 30 20 10 0
f1 (ppm)

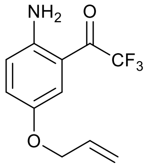

**1l**

$^{19}$F NMR (376 MHz, $CDCl_3$)

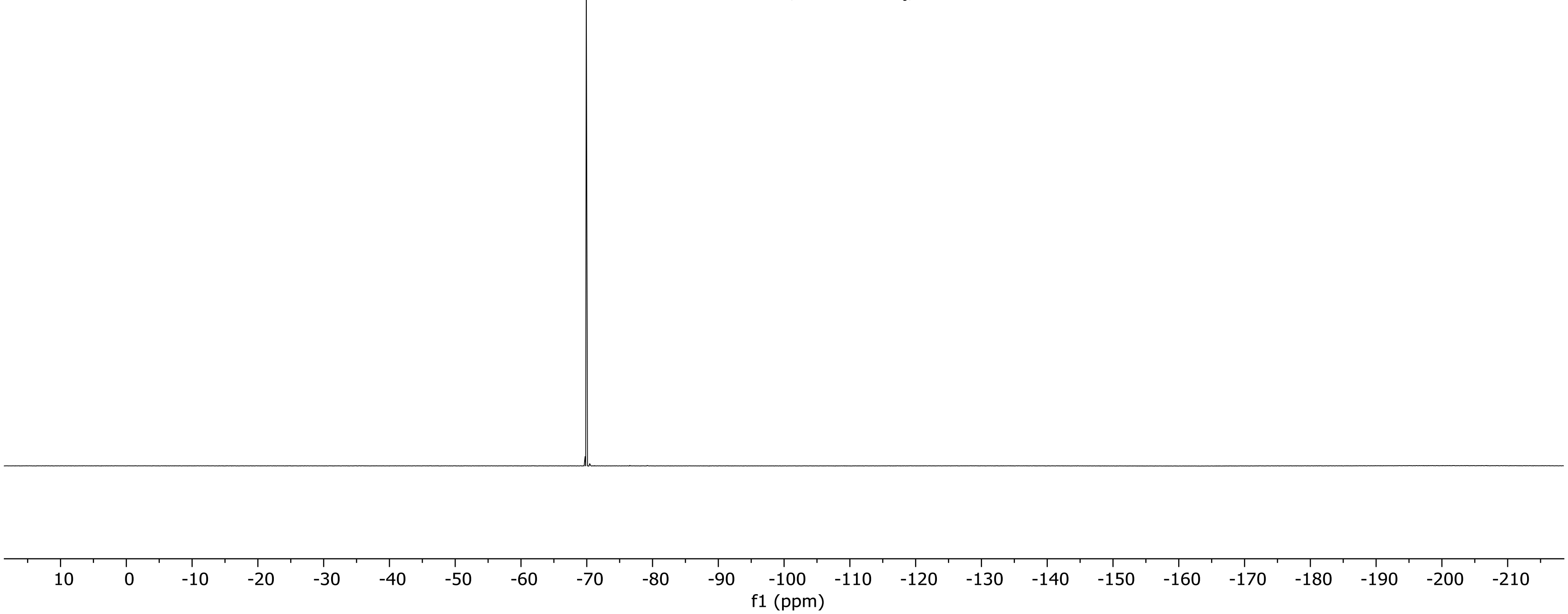

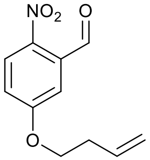

13

$^1H$ NMR (400 MHz, $CDCl_3$)

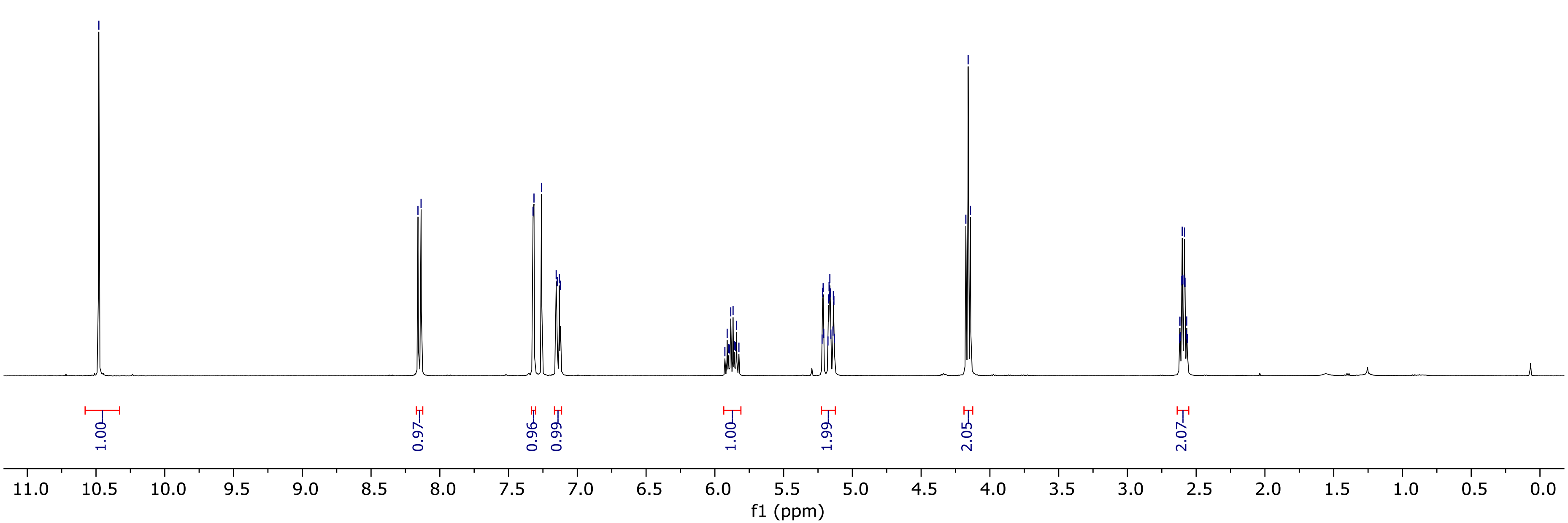

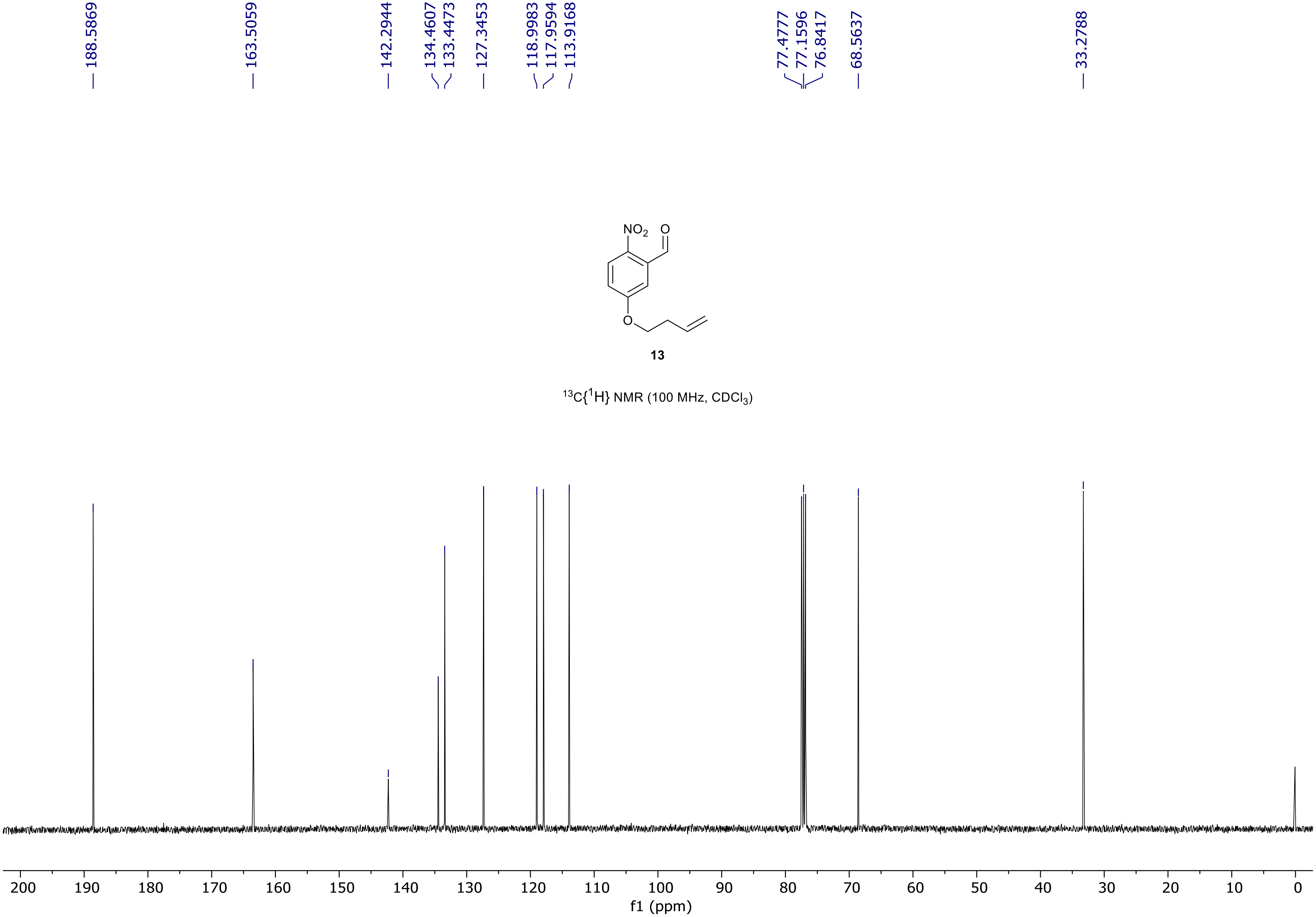

188.5869
163.5059
142.2944
134.4607
133.4473
127.3453
118.9983
117.9594
113.9168
77.4777
77.1596
76.8417
68.5637
33.2788
$NO_2$
O
O
13
$^{13}C\{^1H\}$ NMR (100 MHz, $CDCl_3$)
200 190 180 170 160 150 140 130 120 110 100 90 80 70 60 50 40 30 20 10 0
f1 (ppm)

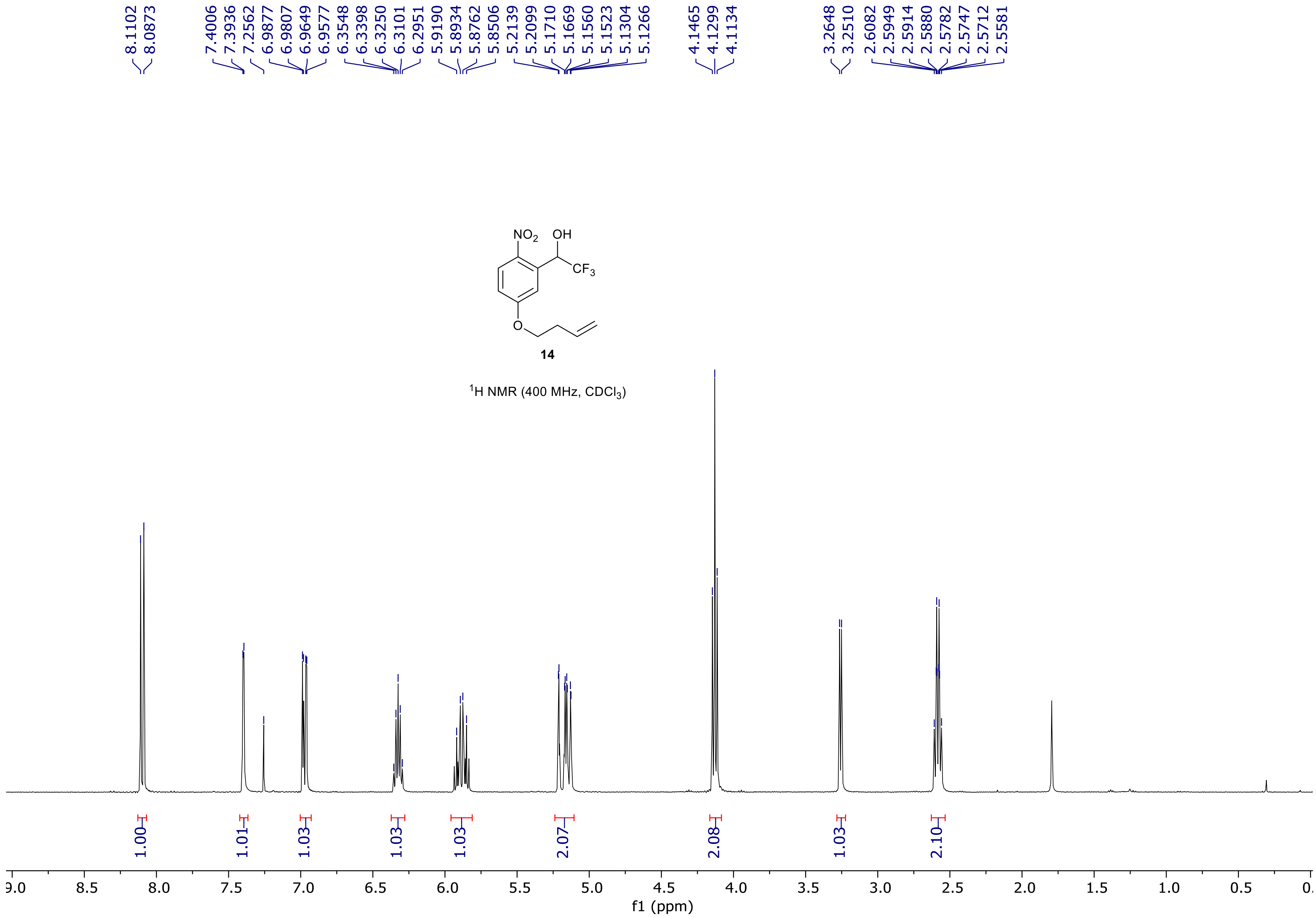
8.1102
8.0873
7.4006
7.3936
7.2562
6.9877
6.9807
6.9649
6.9577
6.3548
6.3398
6.3250
6.3101
6.2951
5.9190
5.8934
5.8762
5.8506
5.2139
5.2099
5.1710
5.1669
5.1560
5.1523
5.1304
5.1266
4.1465
4.1299
4.1134
3.2648
3.2510
2.6082
2.5949
2.5914
2.5880
2.5782
2.5747
2.5712
2.5581
NO2
OH
CF3
O
14
1H NMR (400 MHz, CDCl3)
1.00
1.01
1.03
1.03
1.03
2.07
2.08
1.03
2.10
f1 (ppm)

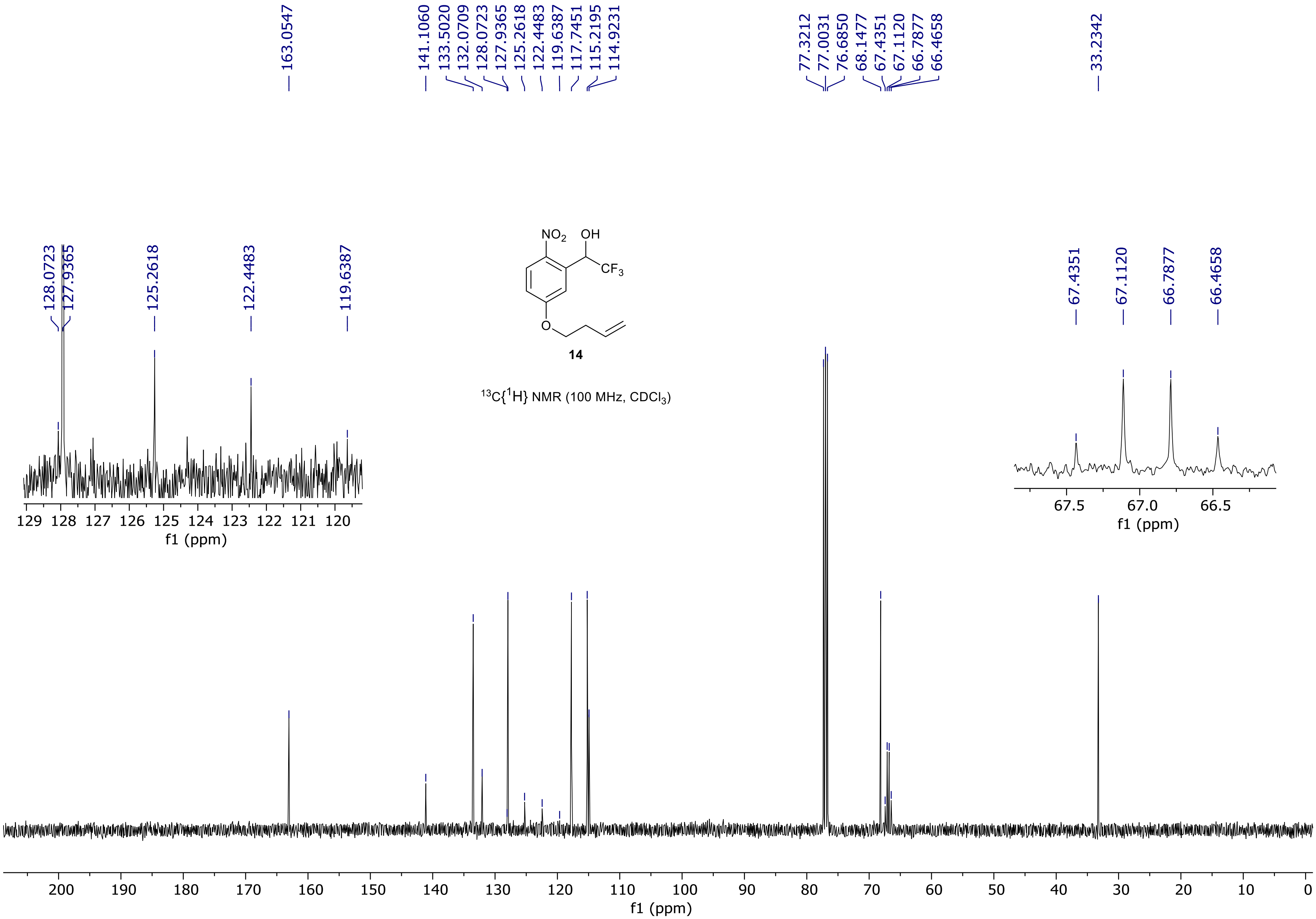
163.0547
141.1060
133.5020
132.0709
128.0723
127.9365
125.2618
122.4483
119.6387
117.7451
115.2195
114.9231
77.3212
77.0031
76.6850
68.1477
67.4351
67.1120
66.7877
66.4658
33.2342
NO2
OH
CF3
O
14
13C{1H} NMR (100 MHz, CDCl3)
f1 (ppm)

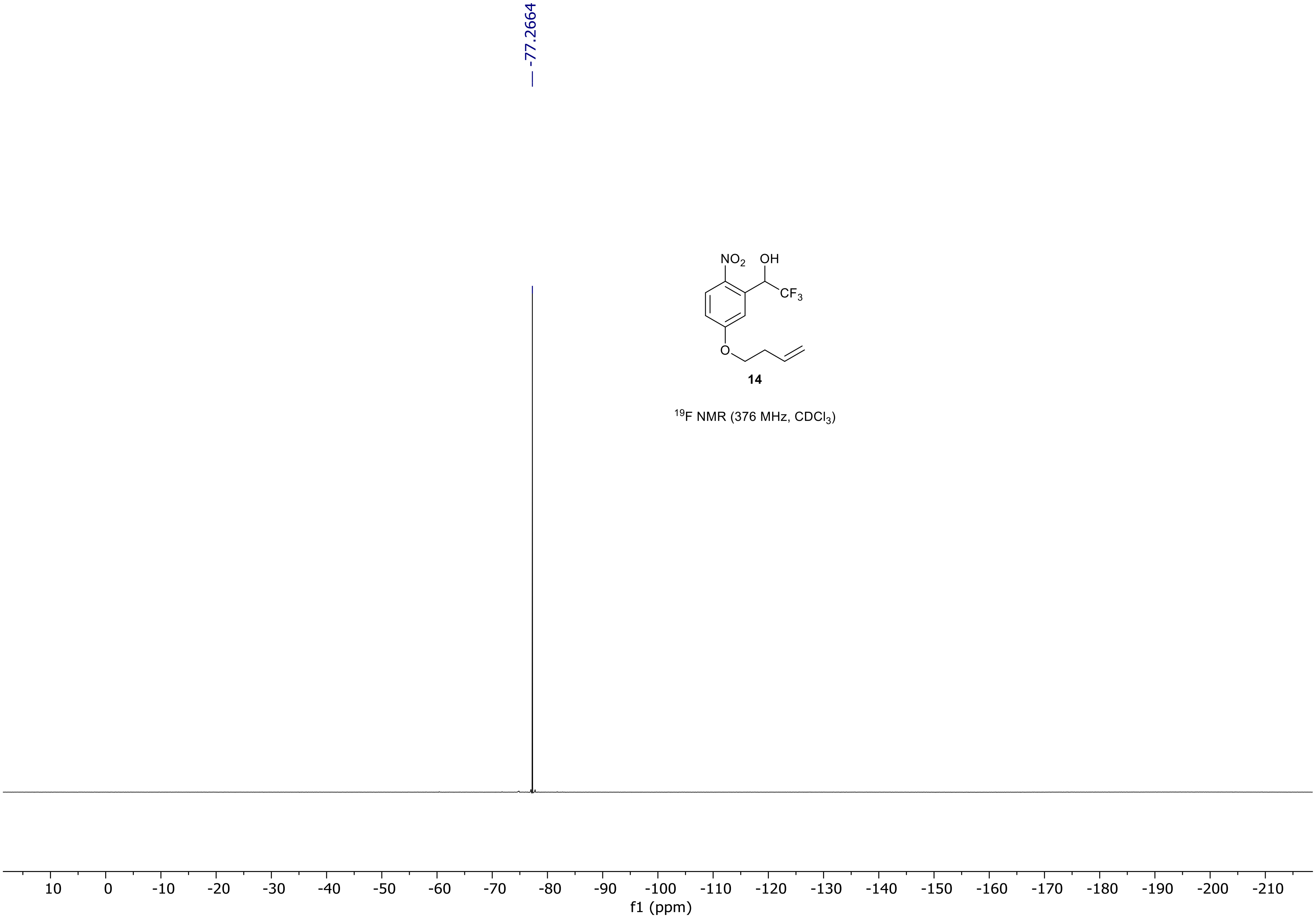


**14**

$^{19}$F NMR (376 MHz, $CDCl_3$)

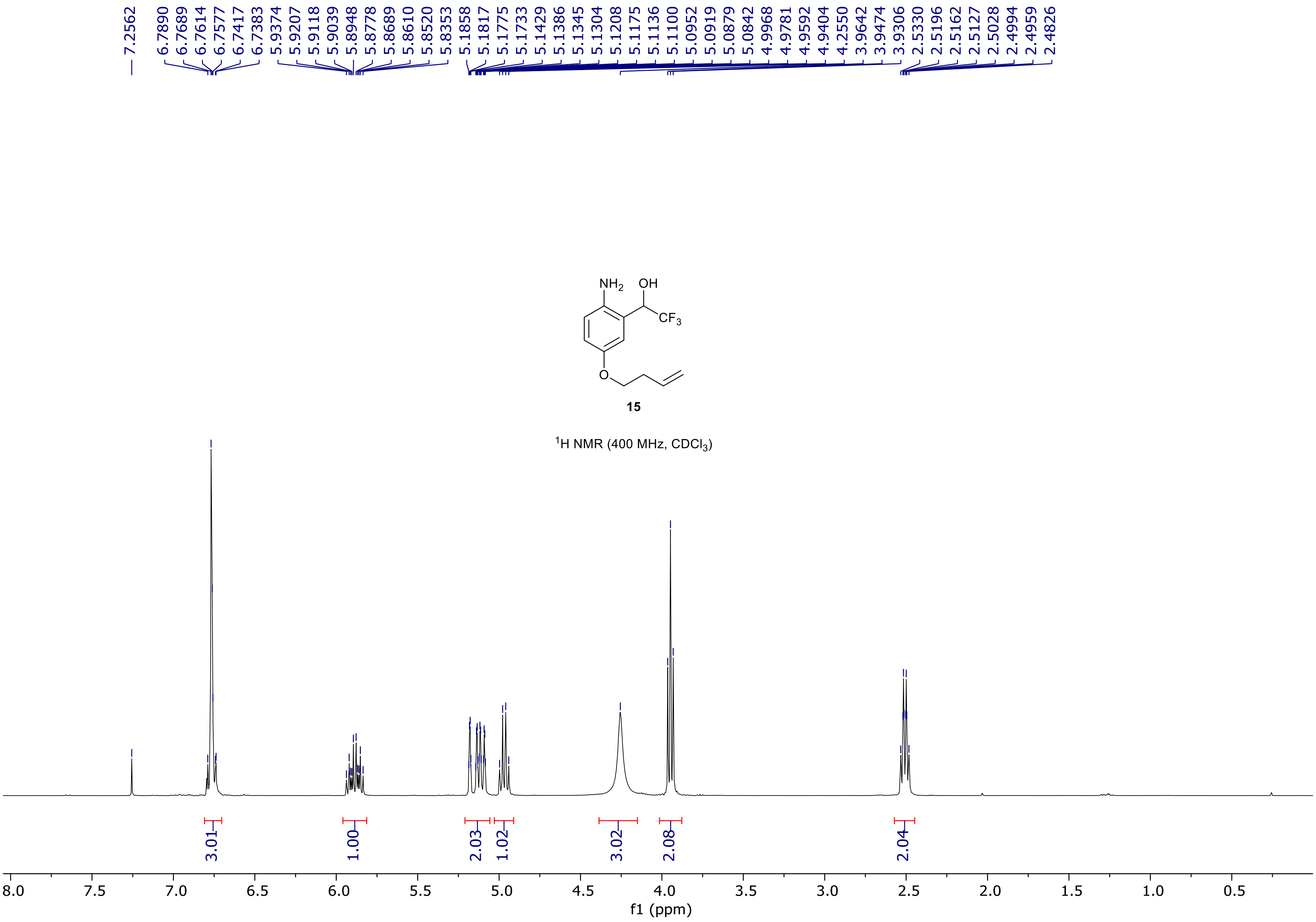

7.2562
6.7890
6.7689
6.7614
6.7577
6.7417
6.7383
5.9374
5.9207
5.9118
5.9039
5.8948
5.8778
5.8689
5.8610
5.8520
5.8353
5.1858
5.1817
5.1775
5.1733
5.1429
5.1386
5.1345
5.1304
5.1208
5.1175
5.1136
5.1100
5.0952
5.0919
5.0879
5.0842
4.9968
4.9781
4.9592
4.9404
4.2550
3.9642
3.9474
3.9306
2.5330
2.5196
2.5162
2.5127
2.5028
2.4994
2.4959
2.4826
NH2
OH
CF3
O
15
1H NMR (400 MHz, CDCl3)
3.01
1.00
2.03
1.02
3.02
2.08
2.04
8.0
7.5
7.0
6.5
6.0
5.5
5.0
4.5
4.0
3.5
3.0
2.5
2.0
1.5
1.0
0.5
f1 (ppm)

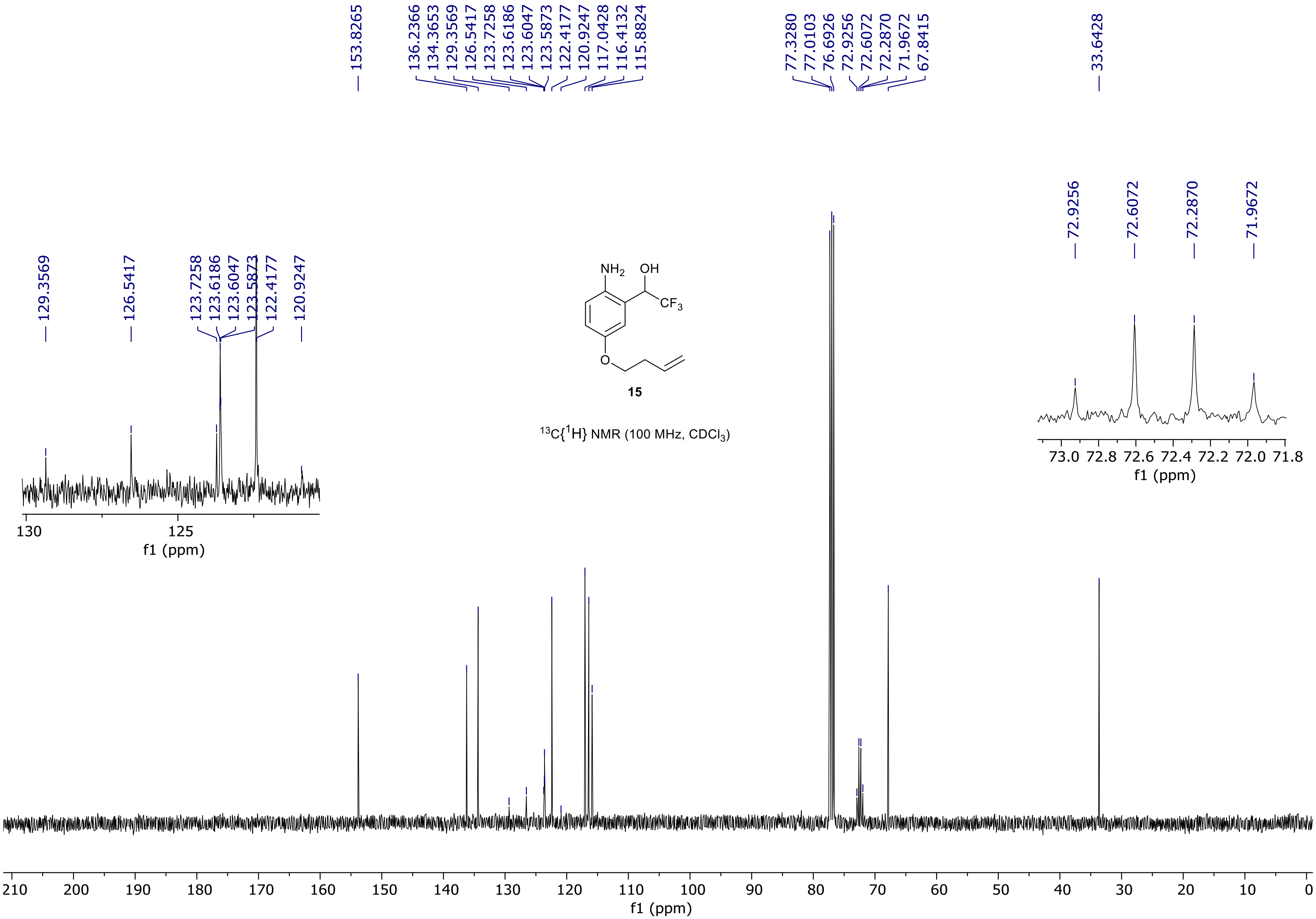
153.8265
136.2366
134.3653
129.3569
126.5417
123.7258
123.6186
123.6047
123.5873
122.4177
120.9247
117.0428
116.4132
115.8824
77.3280
77.0103
76.6926
72.9256
72.6072
72.2870
71.9672
67.8415
33.6428
NH2
OH
CF3
O
15
13C{1H} NMR (100 MHz, CDCl3)
f1 (ppm)

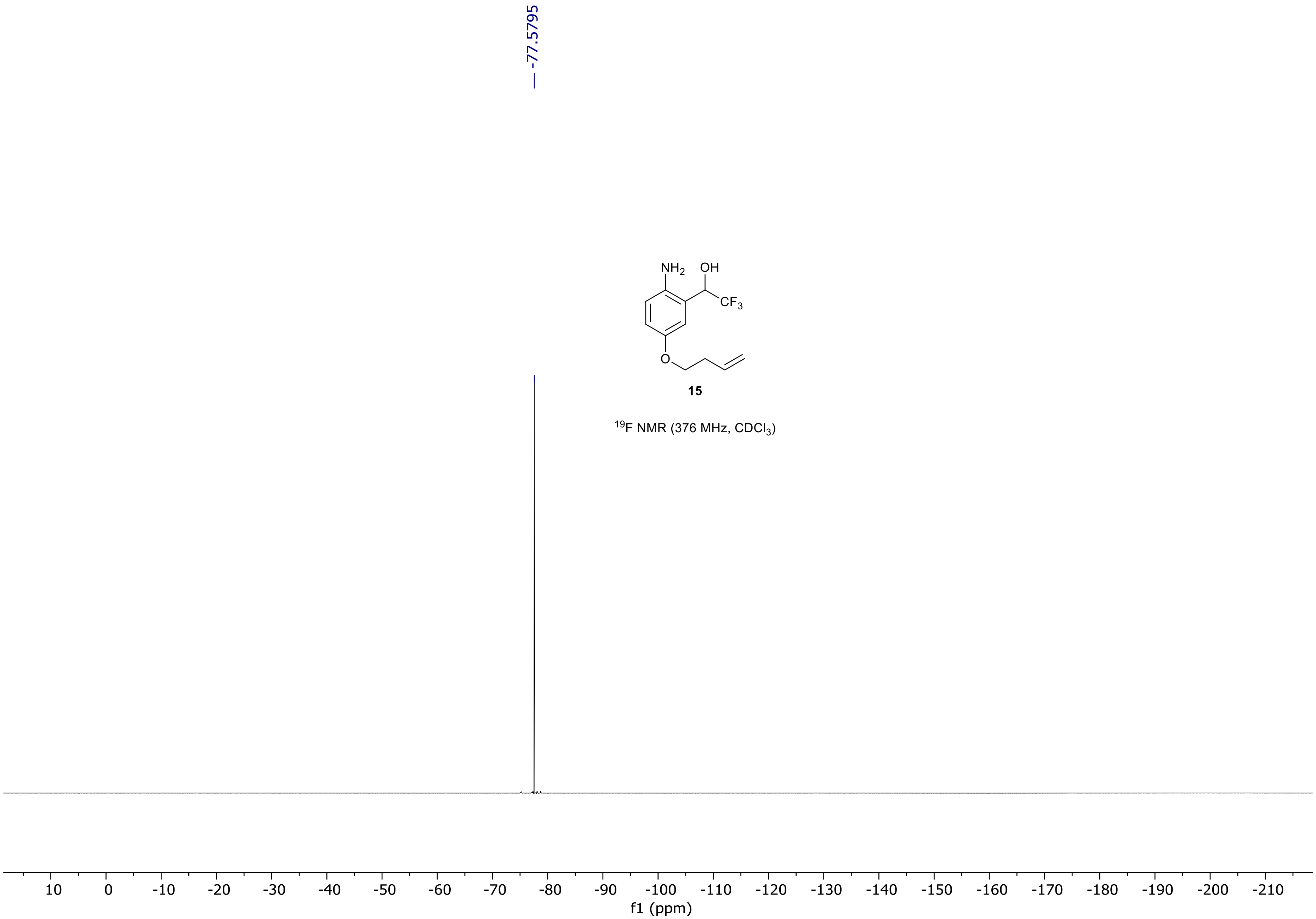

-77.5795
NH2 OH
CF3
O
15
19F NMR (376 MHz, CDCl3)
10 0 -10 -20 -30 -40 -50 -60 -70 -80 -90 -100 -110 -120 -130 -140 -150 -160 -170 -180 -190 -200 -210
f1 (ppm)

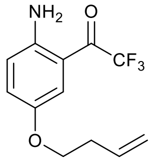


**1m**

$^{1}$H NMR (400 MHz, $CDCl_3$)

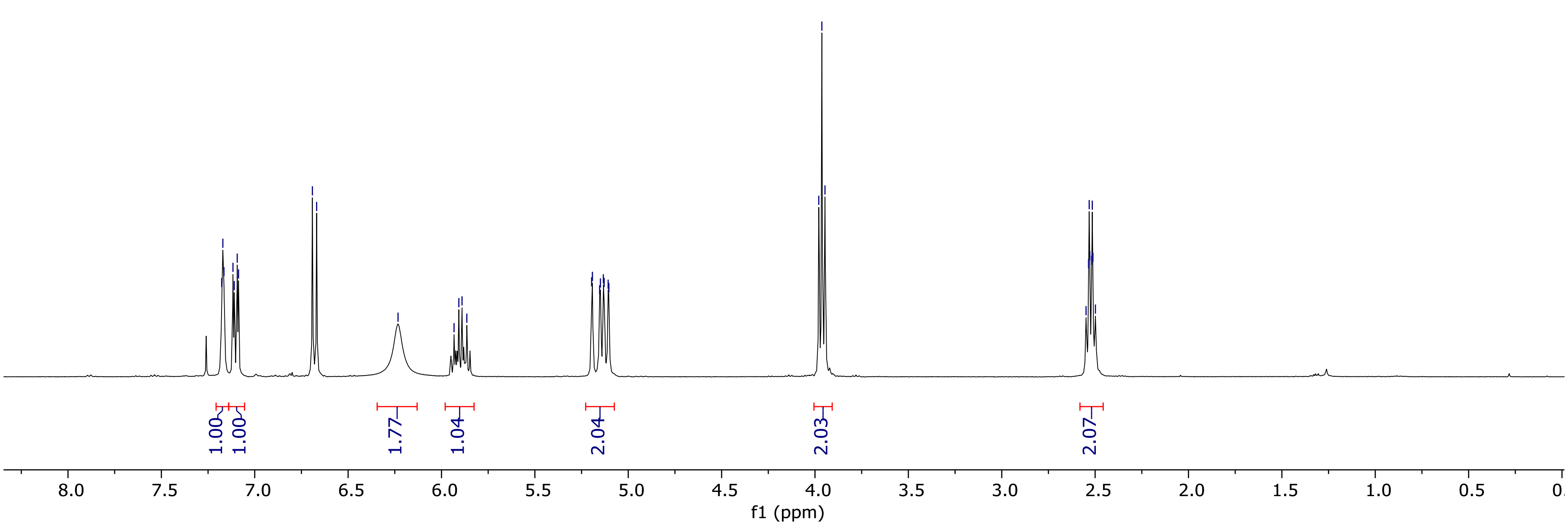

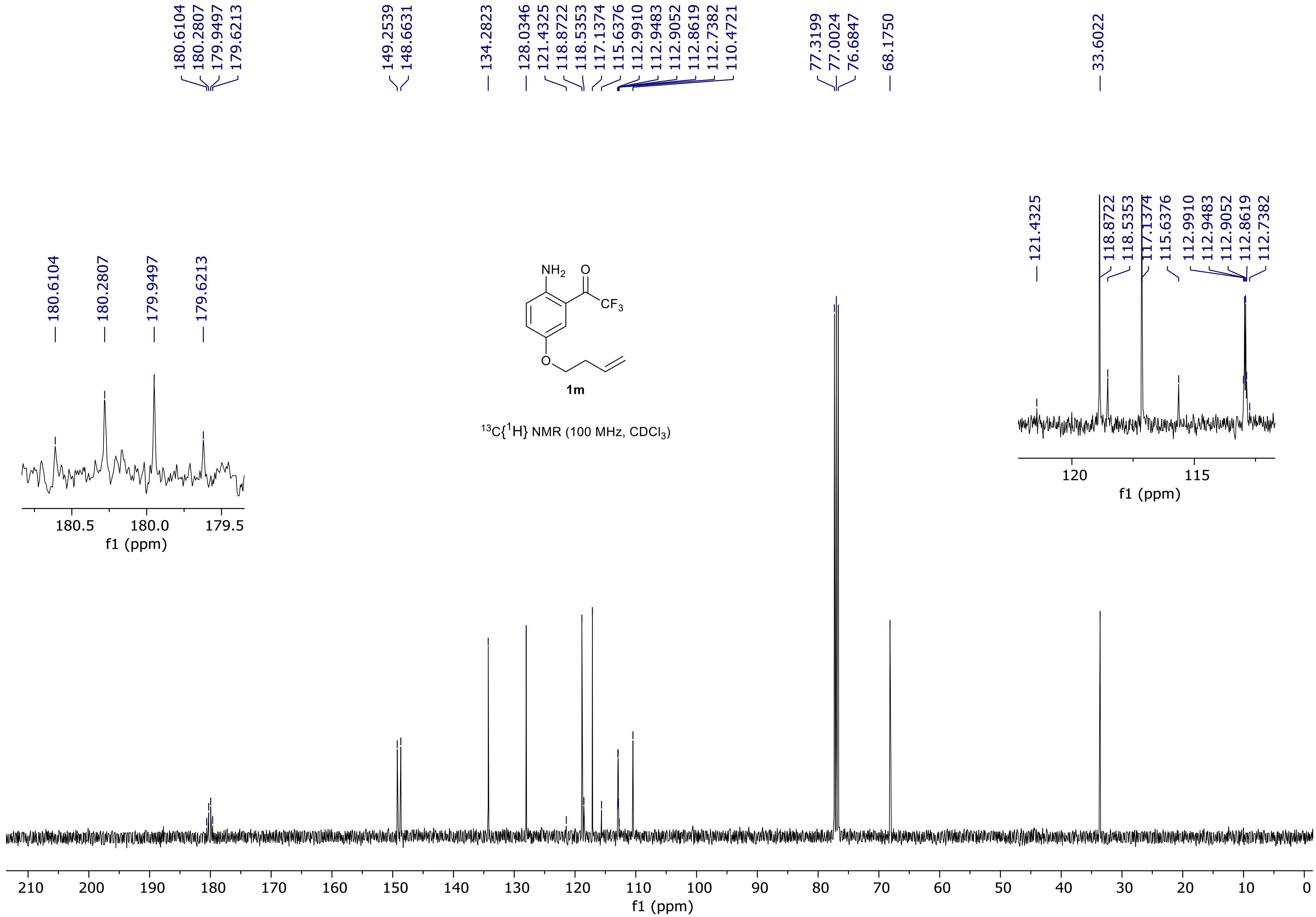

180.6104
180.2807
179.9497
179.6213
149.2539
148.6631
134.2823
128.0346
121.4325
118.8722
118.5353
117.1374
115.6376
112.9910
112.9483
112.9052
112.8619
112.7382
110.4721
77.3199
77.0024
76.6847
68.1750
33.6022
NH2
O
CF3
O
1m
13C{1H} NMR (100 MHz, CDCl3)
f1 (ppm)

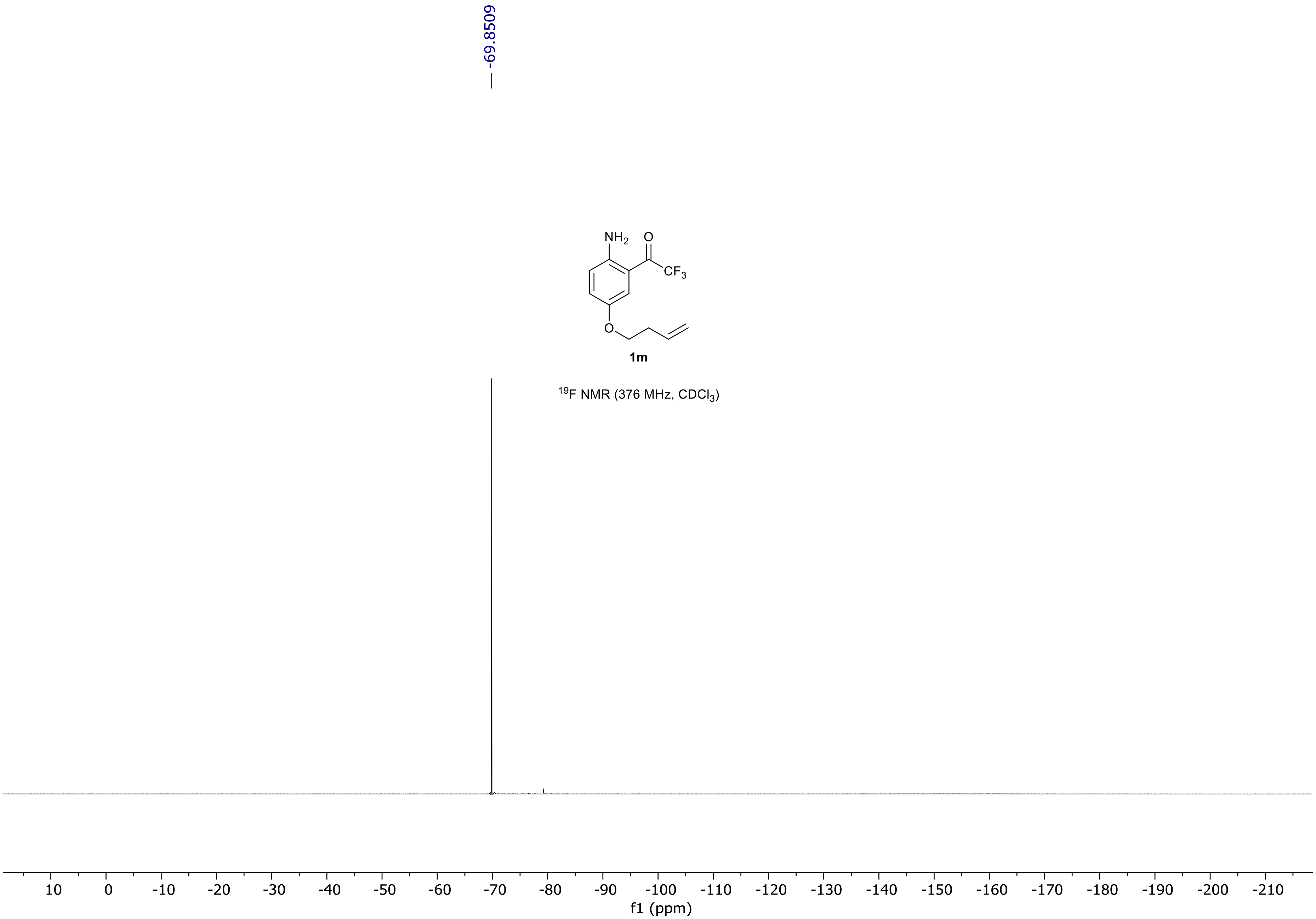

-69.8509
NH2
O
CF3
O
1m
19F NMR (376 MHz, CDCl3)
10 0 -10 -20 -30 -40 -50 -60 -70 -80 -90 -100 -110 -120 -130 -140 -150 -160 -170 -180 -190 -200 -210
f1 (ppm)

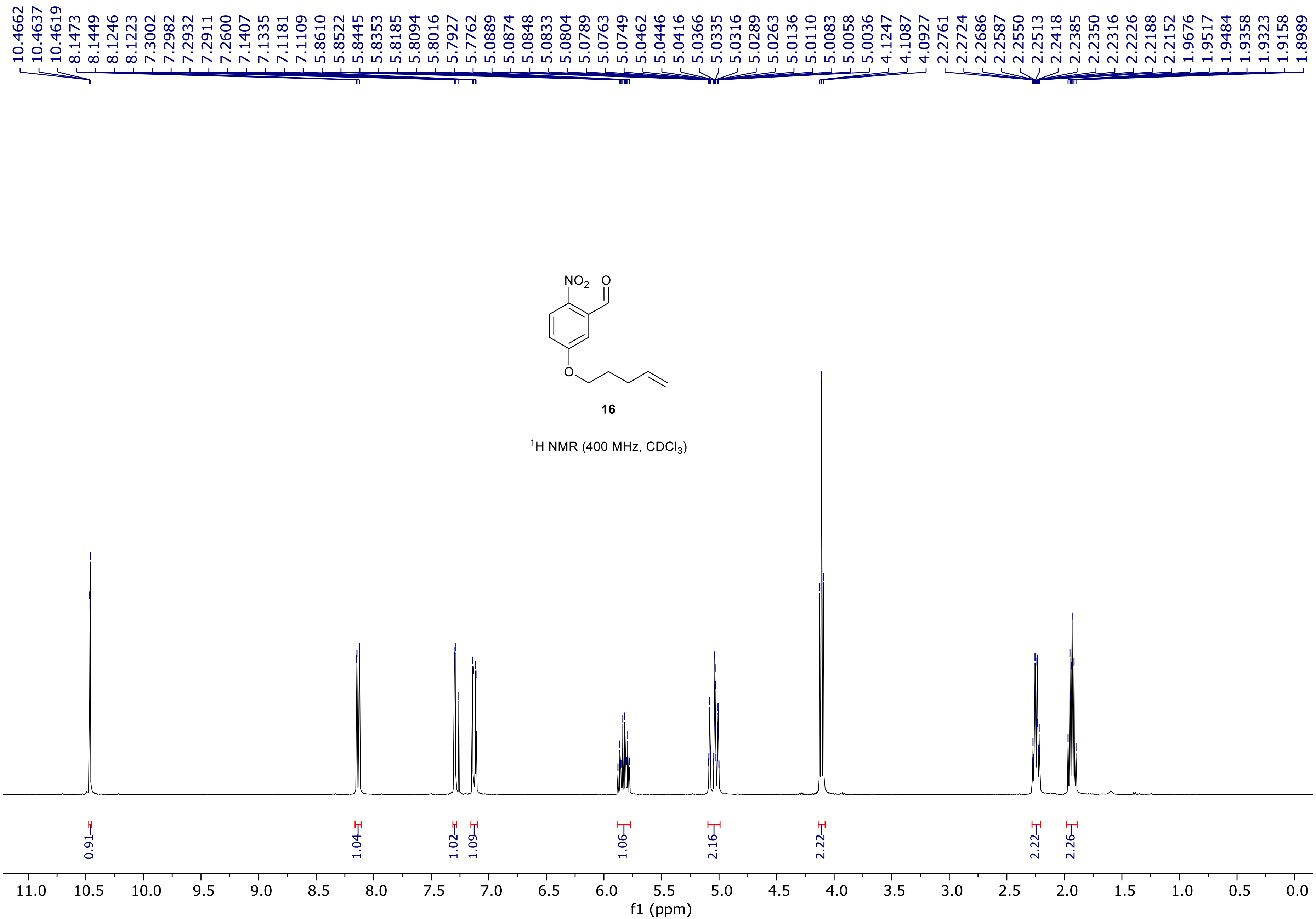


**16**

$^{1}$H NMR (400 MHz, $CDCl_3$)

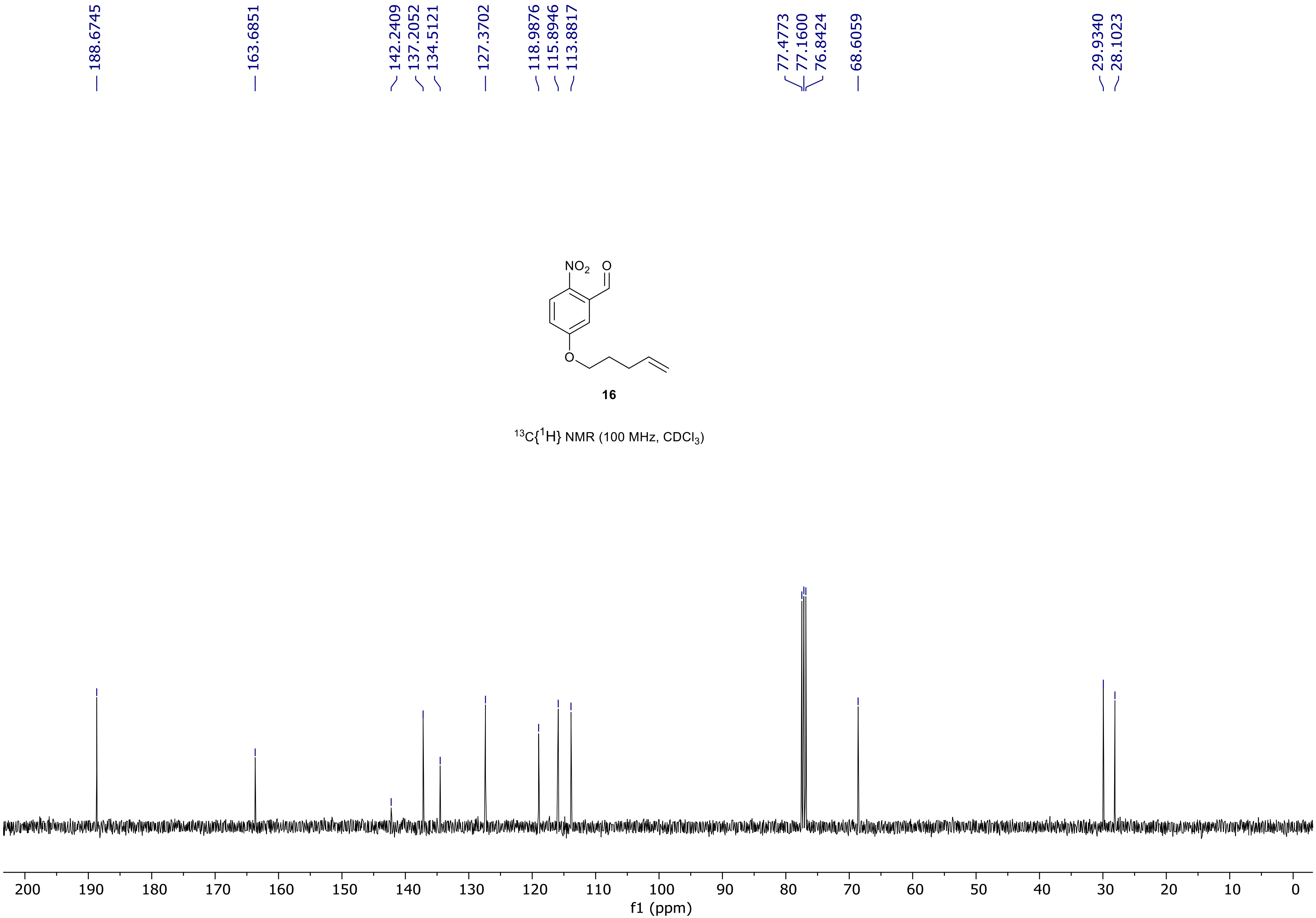
188.6745
163.6851
142.2409
137.2052
134.5121
127.3702
118.9876
115.8946
113.8817
77.4773
77.1600
76.8424
68.6059
29.9340
28.1023
NO2
O
16
13C{1H} NMR (100 MHz, CDCl3)
200
190
180
170
160
150
140
130
120
110
100
90
80
70
60
50
40
30
20
10
0
f1 (ppm)

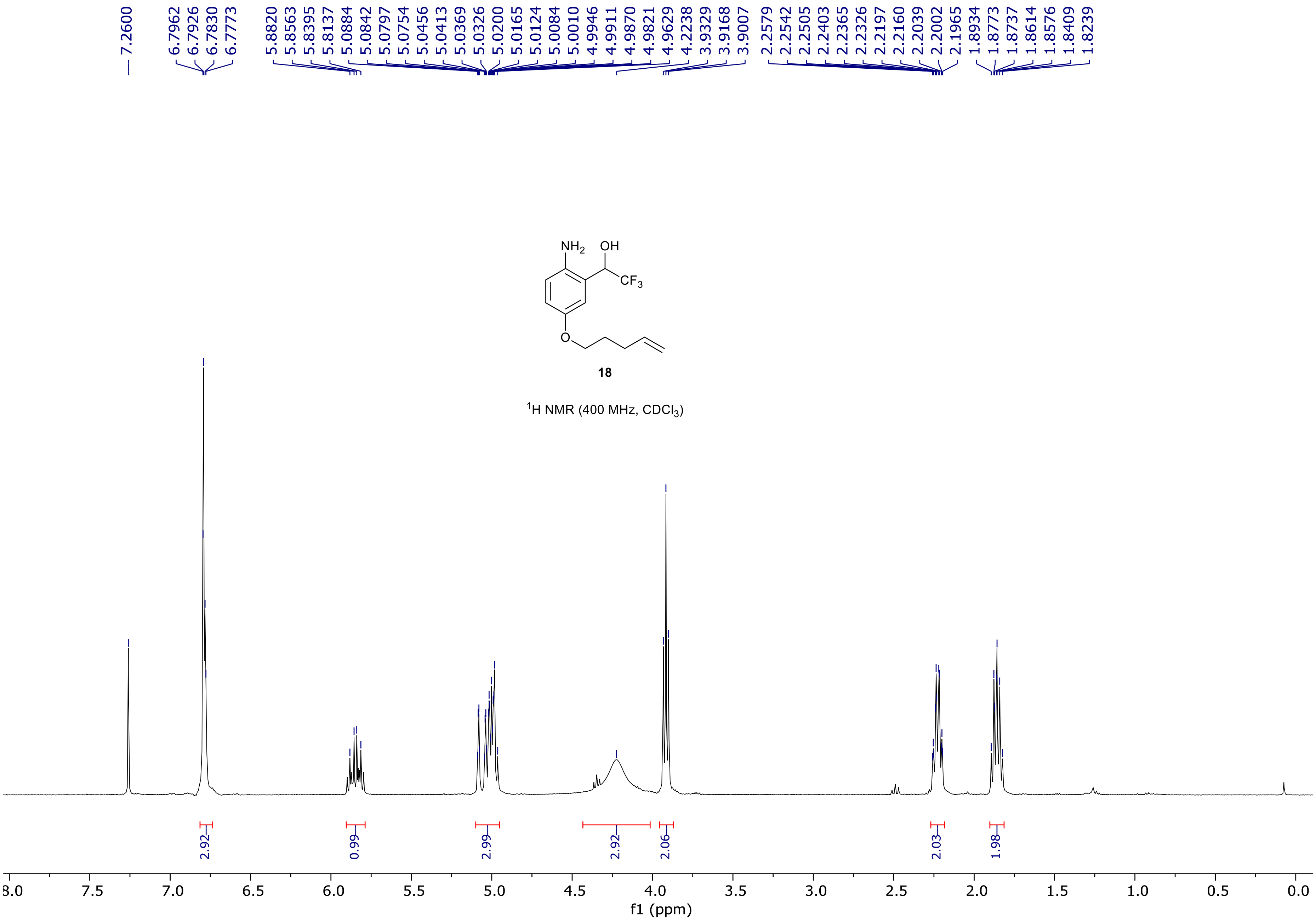

7.2600
6.7962 6.7926 6.7830 6.7773
5.8820 5.8563 5.8395 5.8137 5.0884 5.0842 5.0797 5.0754 5.0456 5.0413 5.0369 5.0326 5.0200 5.0165 5.0124 5.0084 5.0010 4.9946 4.9911 4.9870 4.9821 4.9629 4.2238 3.9329 3.9168 3.9007
2.2579 2.2542 2.2505 2.2403 2.2365 2.2326 2.2197 2.2160 2.2039 2.2002 2.1965 1.8934 1.8773 1.8737 1.8614 1.8576 1.8409 1.8239
NH2
OH
CF3
O
18
1H NMR (400 MHz, CDCl3)
2.92
0.99
2.99
2.92
2.06
2.03
1.98
8.0 7.5 7.0 6.5 6.0 5.5 5.0 4.5 4.0 3.5 3.0 2.5 2.0 1.5 1.0 0.5 0.0
f1 (ppm)

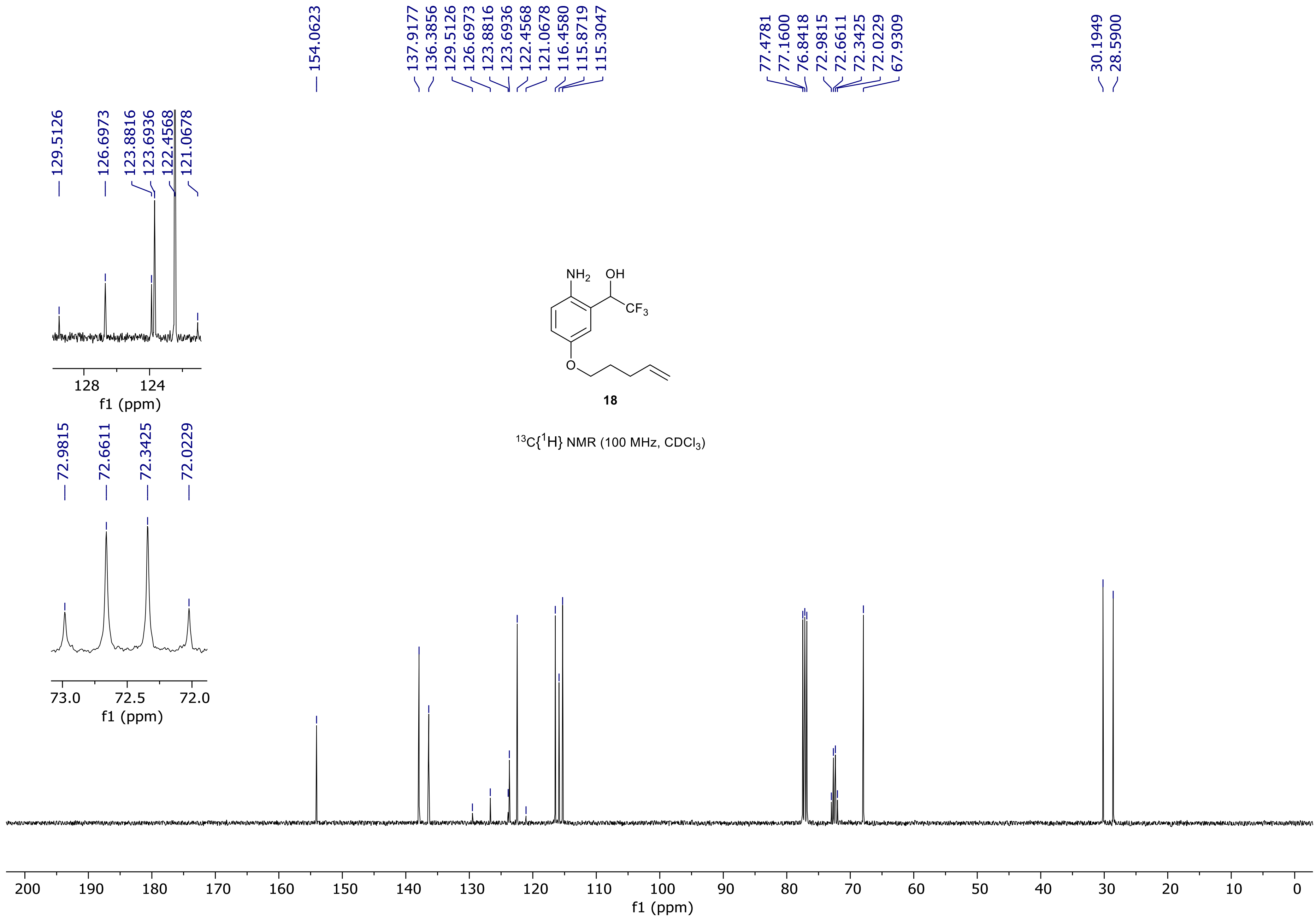
154.0623
137.9177
136.3856
129.5126
126.6973
123.8816
123.6936
122.4568
121.0678
116.4580
115.8719
115.3047
77.4781
77.1600
76.8418
72.9815
72.6611
72.3425
72.0229
67.9309
30.1949
28.5900
NH2
OH
CF3
O
18
13C{1H} NMR (100 MHz, CDCl3)
f1 (ppm)

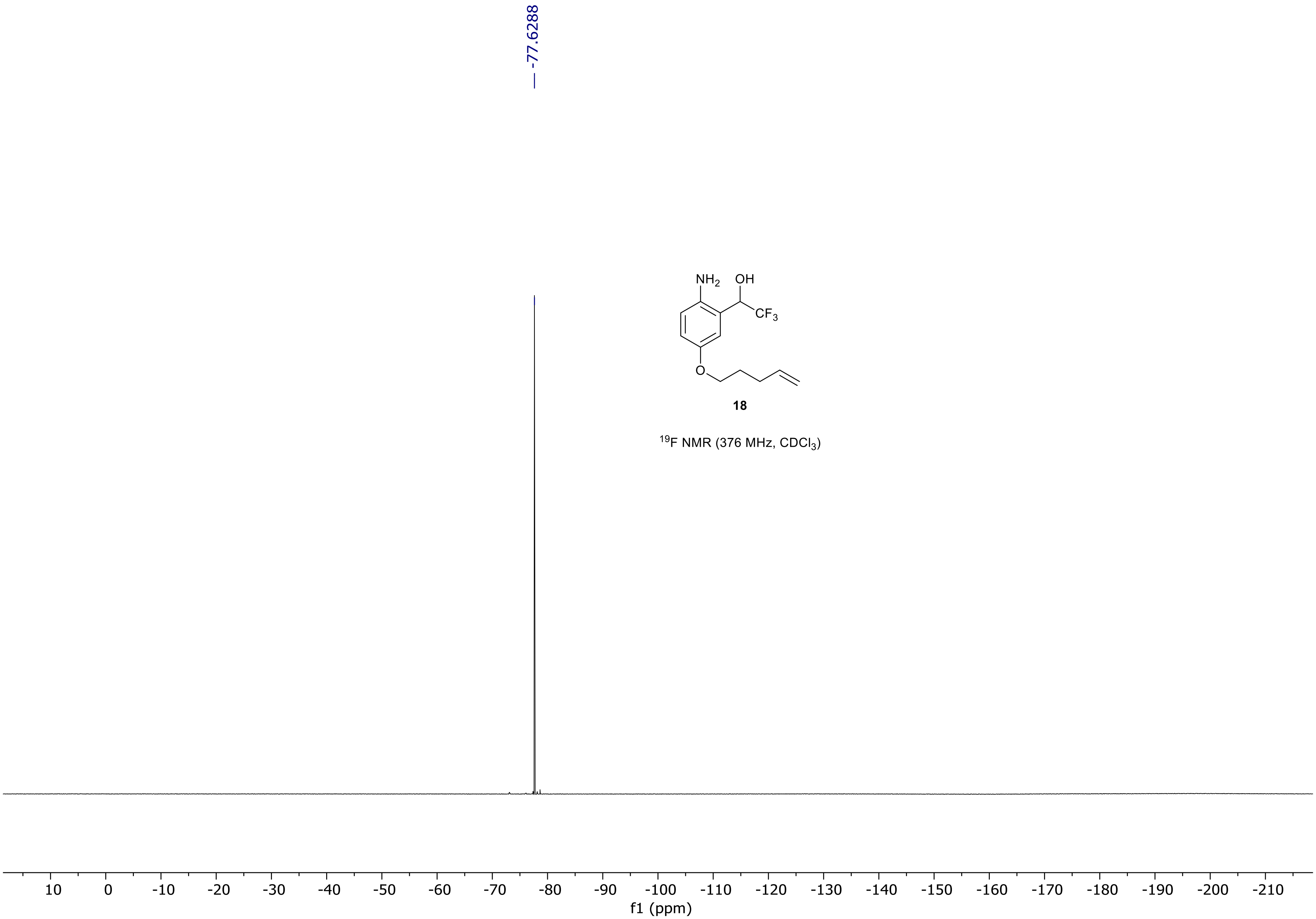
-77.6288
NH2
OH
CF3
O
18
19F NMR (376 MHz, CDCl3)
10 0 -10 -20 -30 -40 -50 -60 -70 -80 -90 -100 -110 -120 -130 -140 -150 -160 -170 -180 -190 -200 -210
f1 (ppm)

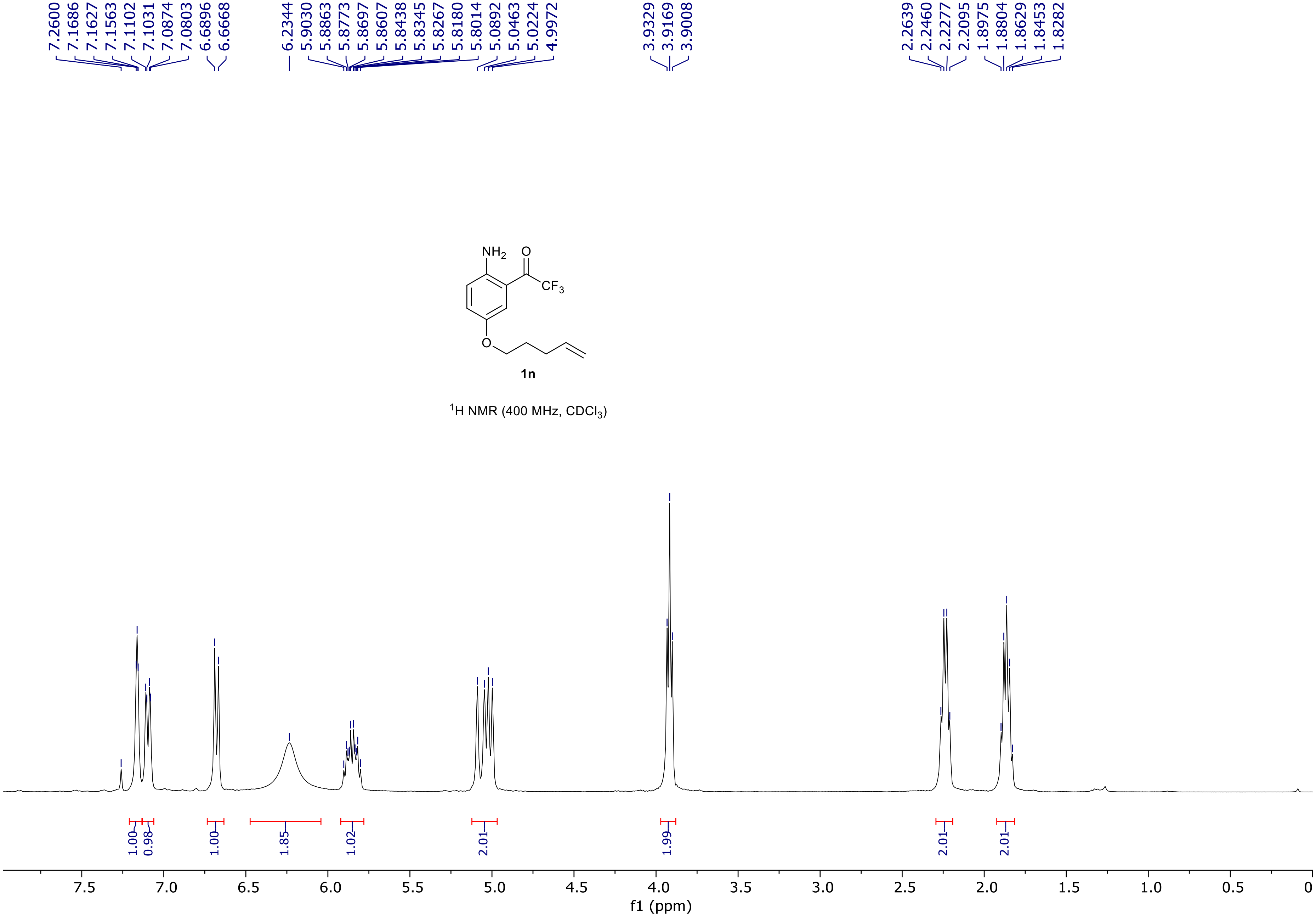
7.2600
7.1686
7.1627
7.1563
7.1102
7.1031
7.0874
7.0803
6.6896
6.6668
6.2344
5.9030
5.8863
5.8773
5.8697
5.8607
5.8438
5.8345
5.8267
5.8180
5.8014
5.0892
5.0463
5.0224
4.9972
3.9329
3.9169
3.9008
2.2639
2.2460
2.2277
2.2095
1.8975
1.8804
1.8629
1.8453
1.8282
NH2
O
CF3
O
1n
1H NMR (400 MHz, CDCl3)
1.00
0.98
1.00
1.85
1.02
2.01
1.99
2.01
2.01
f1 (ppm)

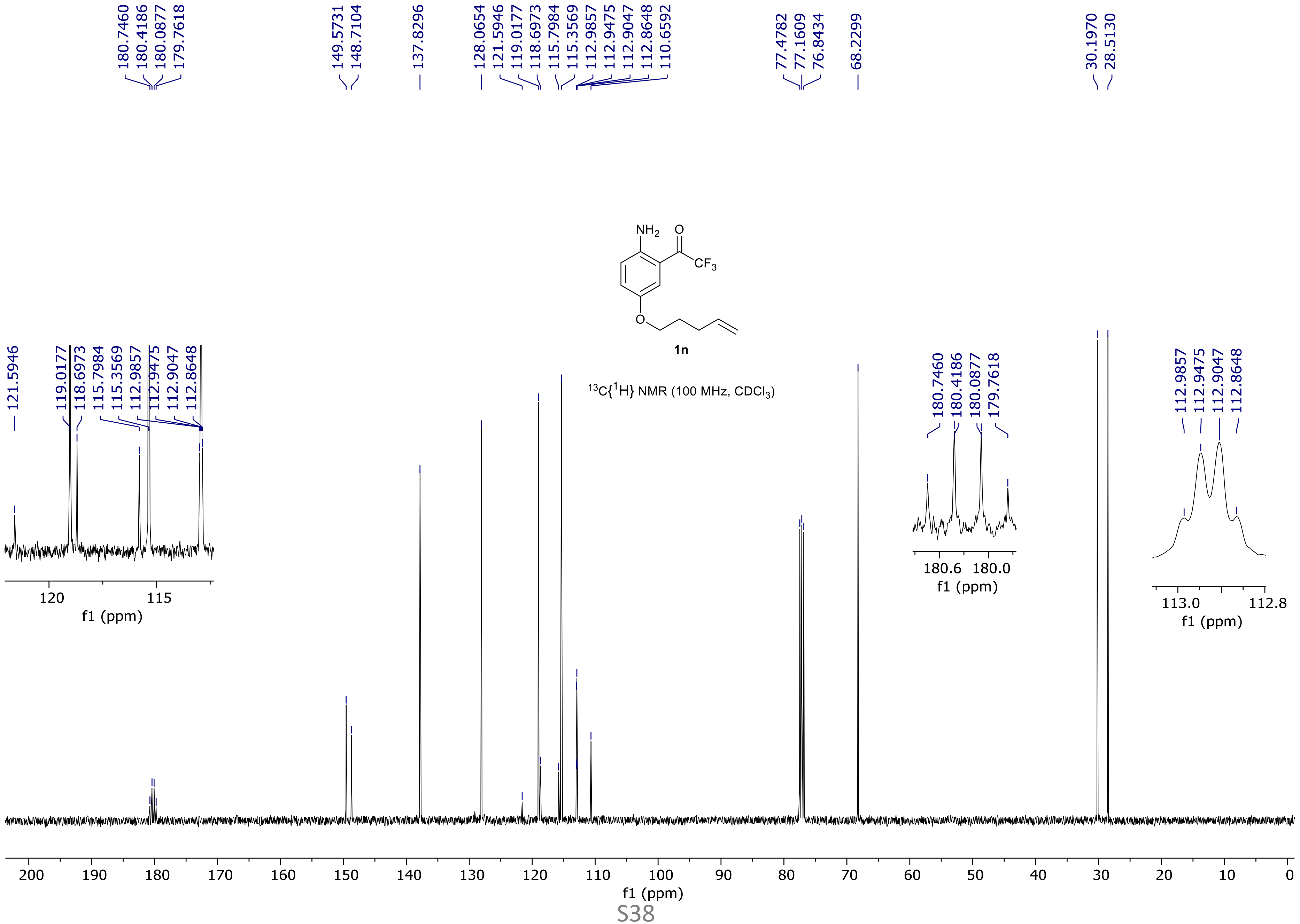
180.7460
180.4186
180.0877
179.7618
149.5731
148.7104
137.8296
128.0654
121.5946
119.0177
118.6973
115.7984
115.3569
112.9857
112.9475
112.9047
112.8648
110.6592
77.4782
77.1609
76.8434
68.2299
30.1970
28.5130
NH2
O
CF3
O
1n
13C{1H} NMR (100 MHz, CDCl3)
f1 (ppm)

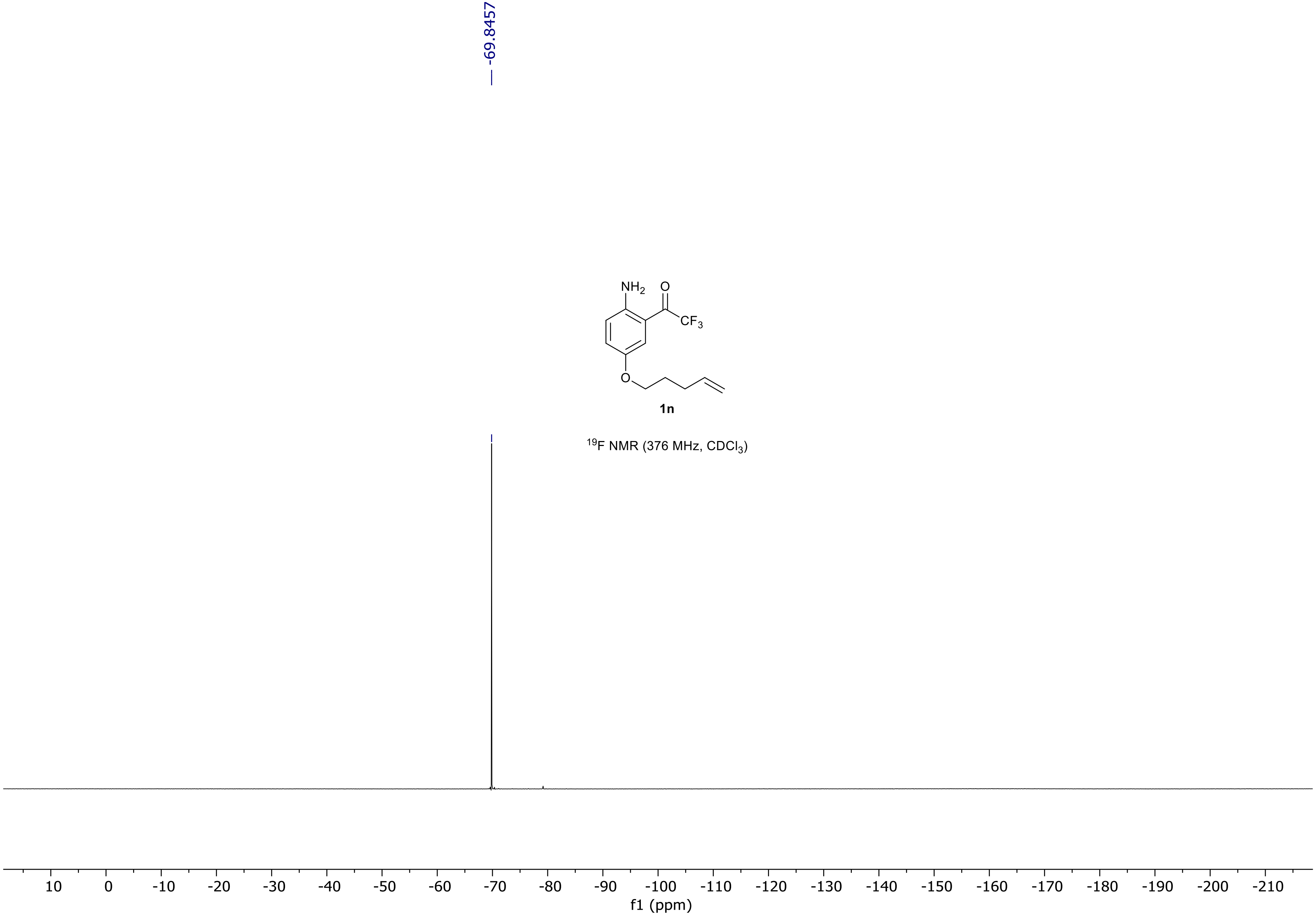

-69.8457
NH2
O
CF3
O
1n
19F NMR (376 MHz, CDCl3)
10 0 -10 -20 -30 -40 -50 -60 -70 -80 -90 -100 -110 -120 -130 -140 -150 -160 -170 -180 -190 -200 -210
f1 (ppm)

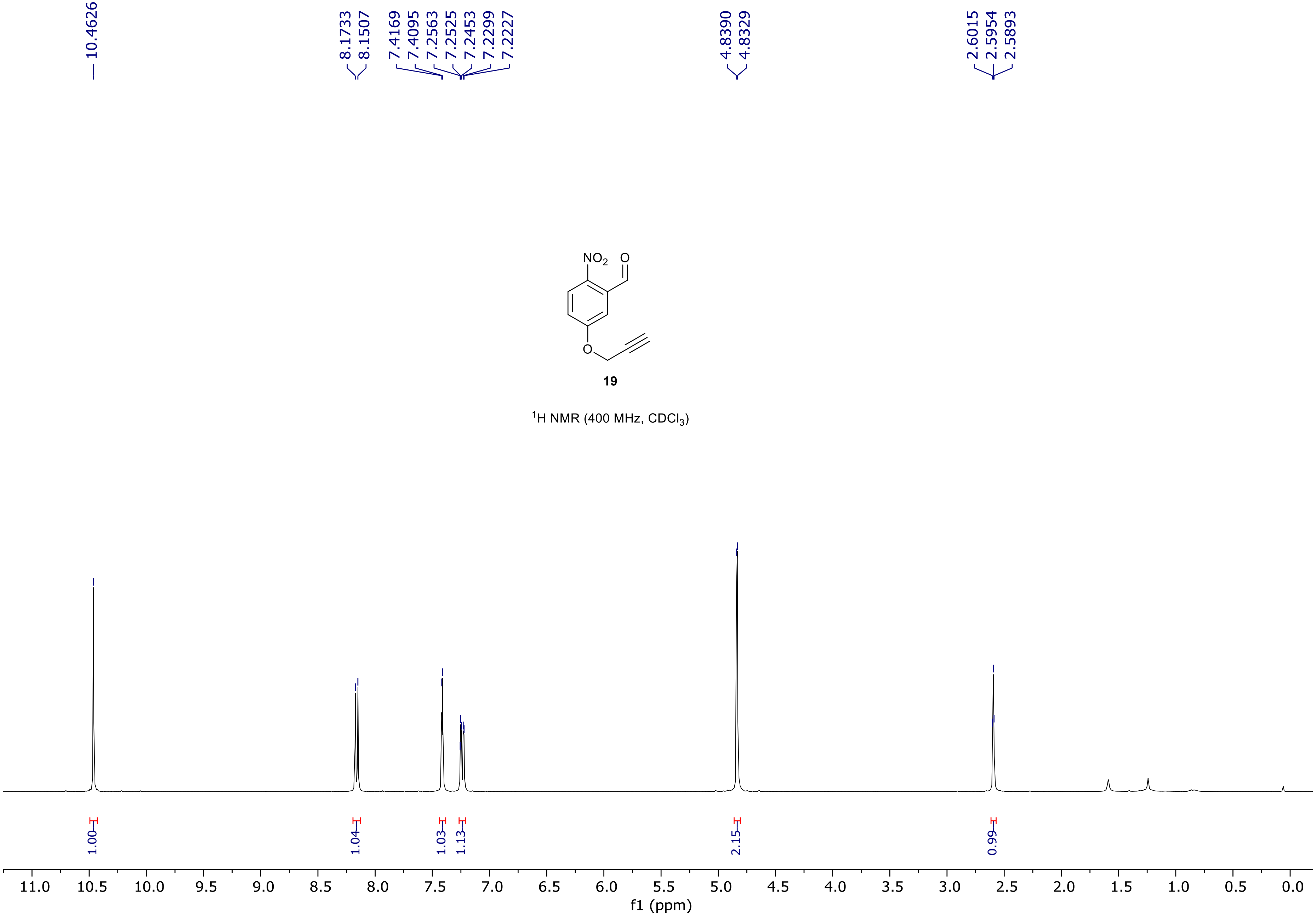
10.4626
8.1733
8.1507
7.4169
7.4095
7.2563
7.2525
7.2453
7.2299
7.2227
4.8390
4.8329
2.6015
2.5954
2.5893
$NO_2$
O
O
19
$^1$H NMR (400 MHz, $CDCl_3$)
1.00
1.04
1.03
1.13
2.15
0.99
11.0 10.5 10.0 9.5 9.0 8.5 8.0 7.5 7.0 6.5 6.0 5.5 5.0 4.5 4.0 3.5 3.0 2.5 2.0 1.5 1.0 0.5 0.0
f1 (ppm)

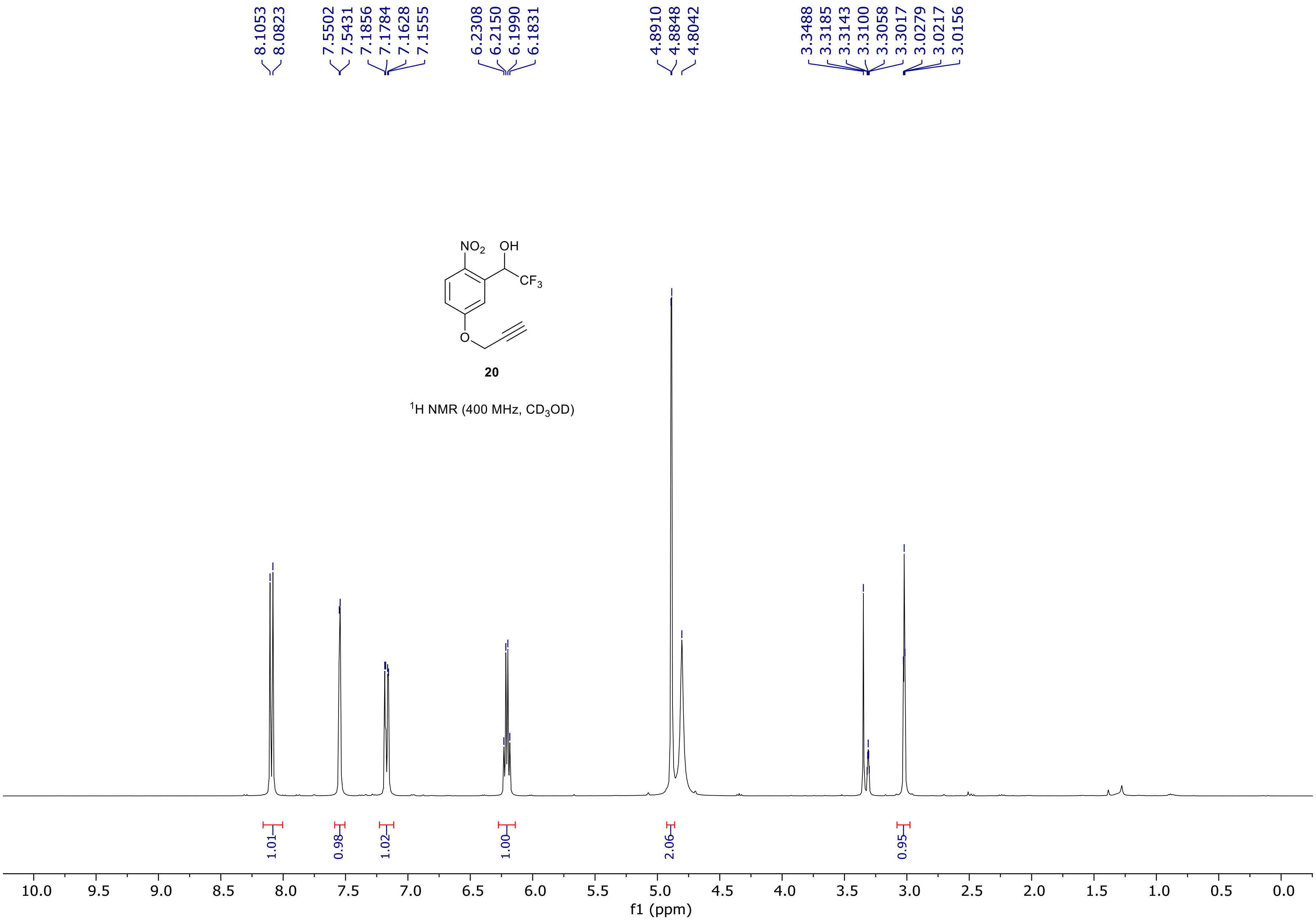
8.1053
8.0823
7.5502
7.5431
7.1856
7.1784
7.1628
7.1555
6.2308
6.2150
6.1990
6.1831
4.8910
4.8848
4.8042
3.3488
3.3185
3.3143
3.3100
3.3058
3.3017
3.0279
3.0217
3.0156
NO2
OH
CF3
O
20
1H NMR (400 MHz, CD3OD)
1.01
0.98
1.02
1.00
2.06
0.95
10.0 9.5 9.0 8.5 8.0 7.5 7.0 6.5 6.0 5.5 5.0 4.5 4.0 3.5 3.0 2.5 2.0 1.5 1.0 0.5 0.0
f1 (ppm)

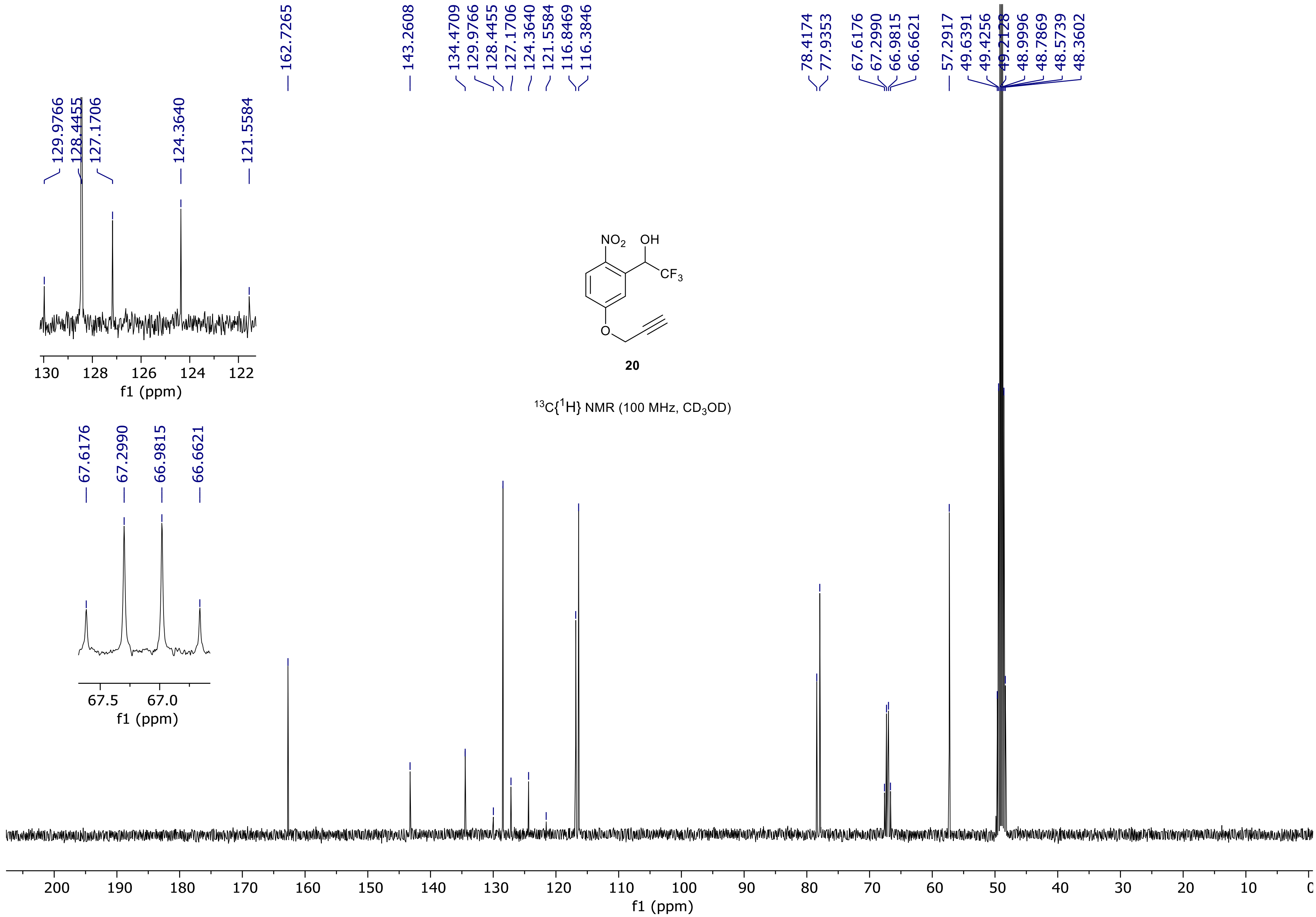
162.7265
143.2608
134.4709
129.9766
128.4455
127.1706
124.3640
121.5584
116.8469
116.3846
78.4174
77.9353
67.6176
67.2990
66.9815
66.6621
57.2917
49.6391
49.4256
49.2128
48.9996
48.7869
48.5739
48.3602
129.9766
128.4455
127.1706
124.3640
121.5584
130 128 126 124 122
f1 (ppm)
67.6176
67.2990
66.9815
66.6621
67.5 67.0
f1 (ppm)
NO2
OH
CF3
O
20
13C{1H} NMR (100 MHz, CD3OD)
200 190 180 170 160 150 140 130 120 110 100 90 80 70 60 50 40 30 20 10 0
f1 (ppm)

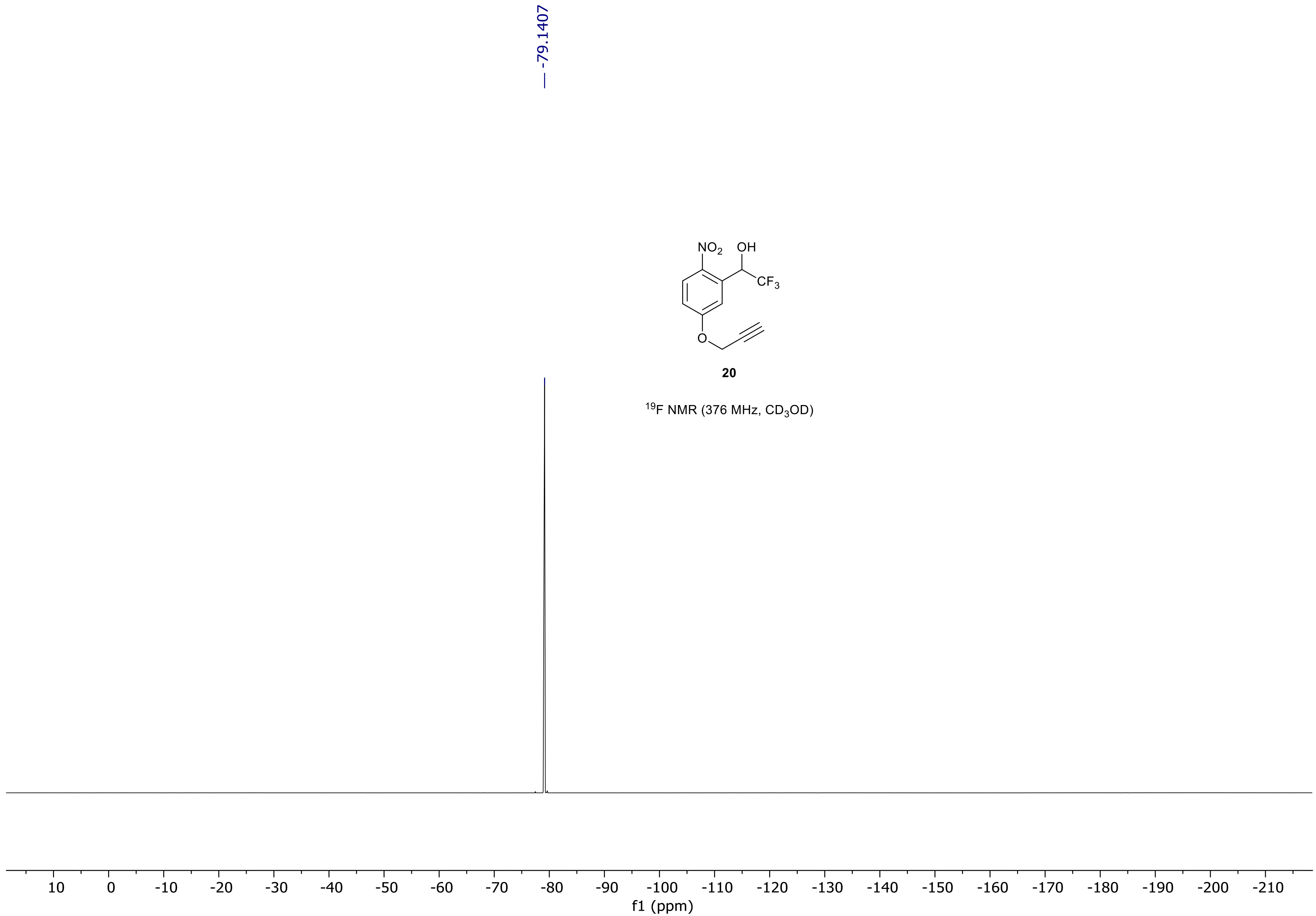
-79.1407
NO2
OH
CF3
O
20
19F NMR (376 MHz, CD3OD)
10 0 -10 -20 -30 -40 -50 -60 -70 -80 -90 -100 -110 -120 -130 -140 -150 -160 -170 -180 -190 -200 -210
f1 (ppm)

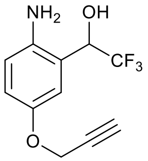


**21**

$^1$H NMR (400 MHz, $CDCl_3$)

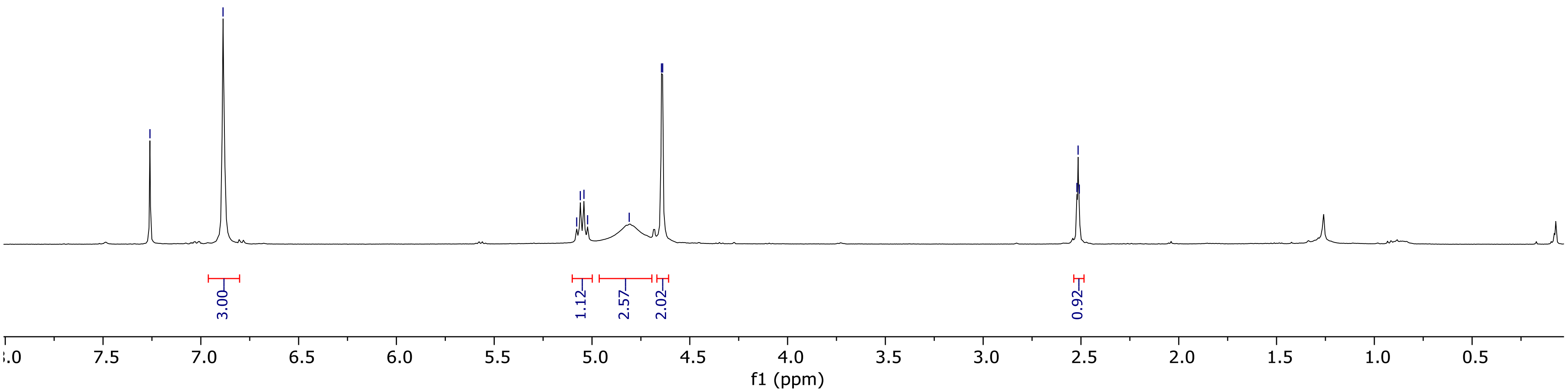

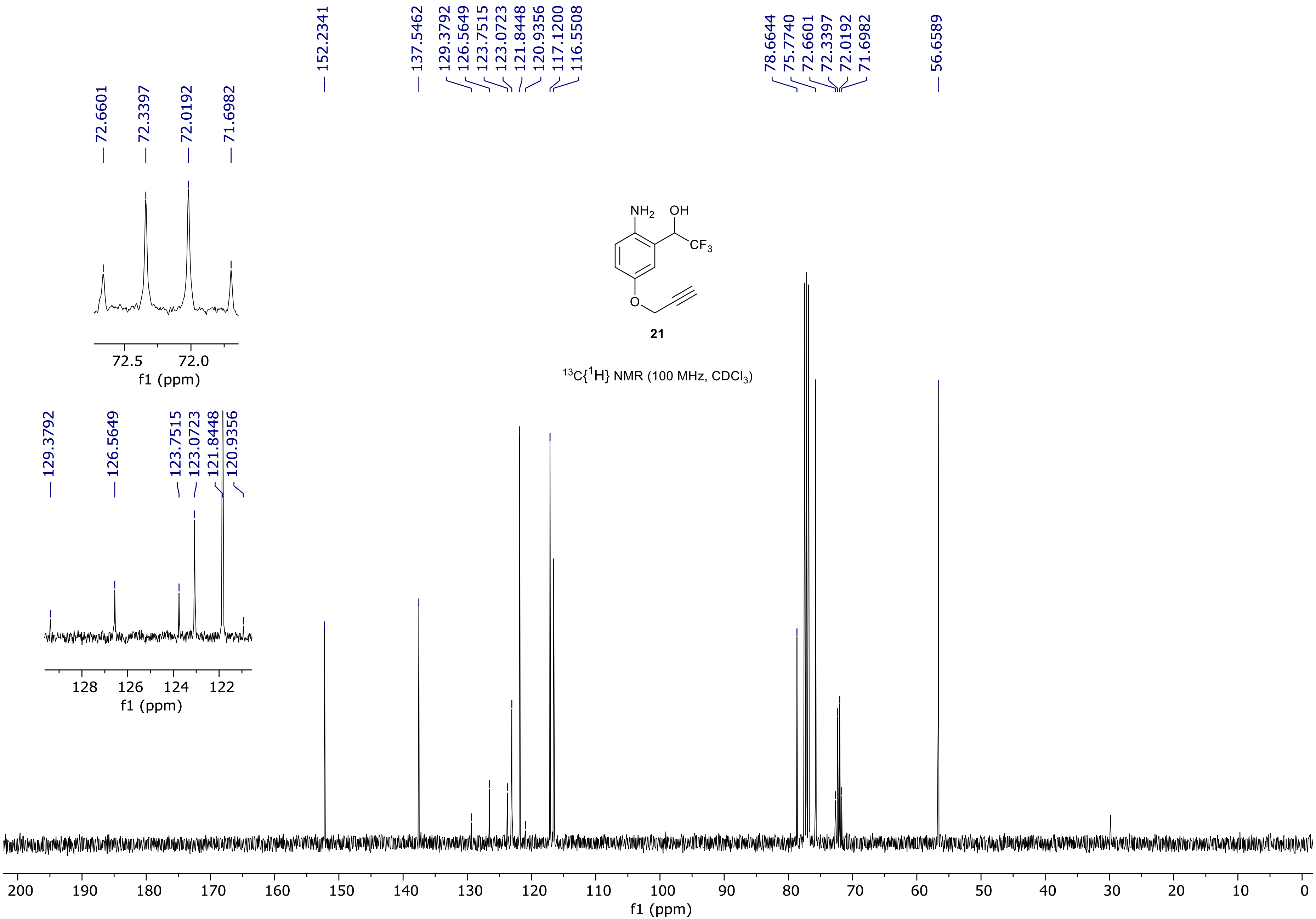

152.2341
137.5462
129.3792
126.5649
123.7515
123.0723
121.8448
120.9356
117.1200
116.5508
78.6644
75.7740
72.6601
72.3397
72.0192
71.6982
56.6589
NH2
OH
CF3
O
21
13C{1H} NMR (100 MHz, CDCl3)
f1 (ppm)

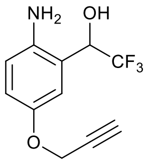

**21**

$^{19}F$ NMR (376 MHz, $CDCl_3$)

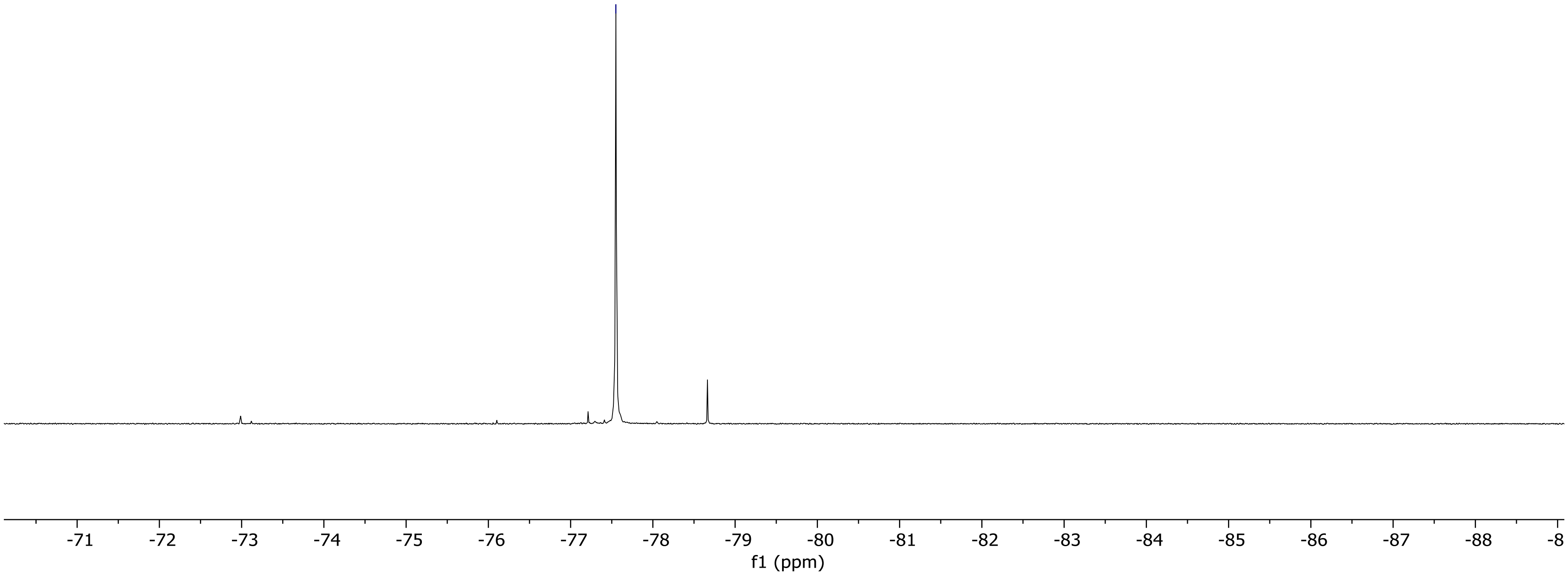

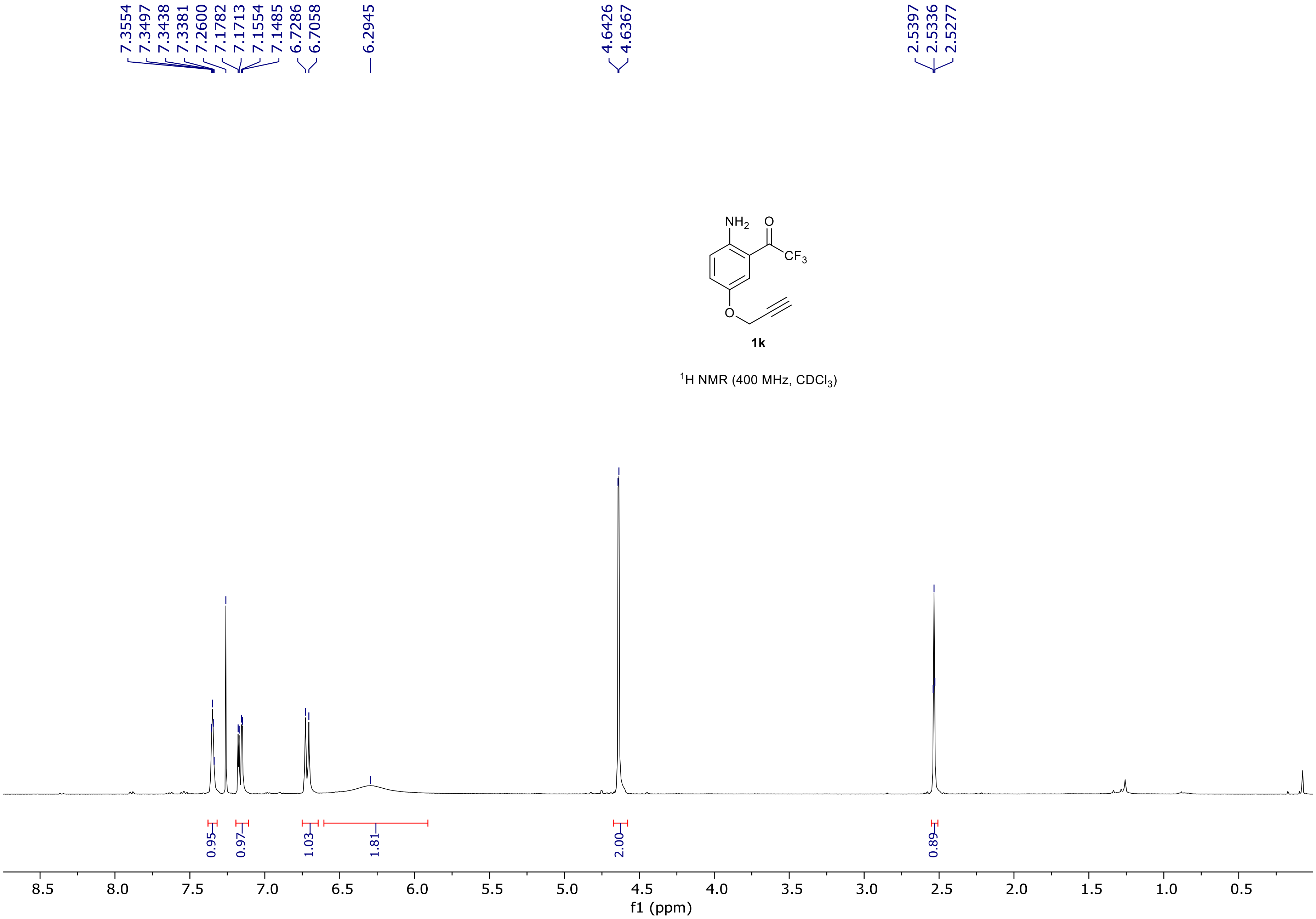

7.3554
7.3497
7.3438
7.3381
7.2600
7.1782
7.1713
7.1554
7.1485
6.7286
6.7058
6.2945
4.6426
4.6367
2.5397
2.5336
2.5277
NH2
O
CF3
O
1k
1H NMR (400 MHz, CDCl3)
0.95
0.97
1.03
1.81
2.00
0.89
8.5
8.0
7.5
7.0
6.5
6.0
5.5
5.0
4.5
4.0
3.5
3.0
2.5
2.0
1.5
1.0
0.5
f1 (ppm)

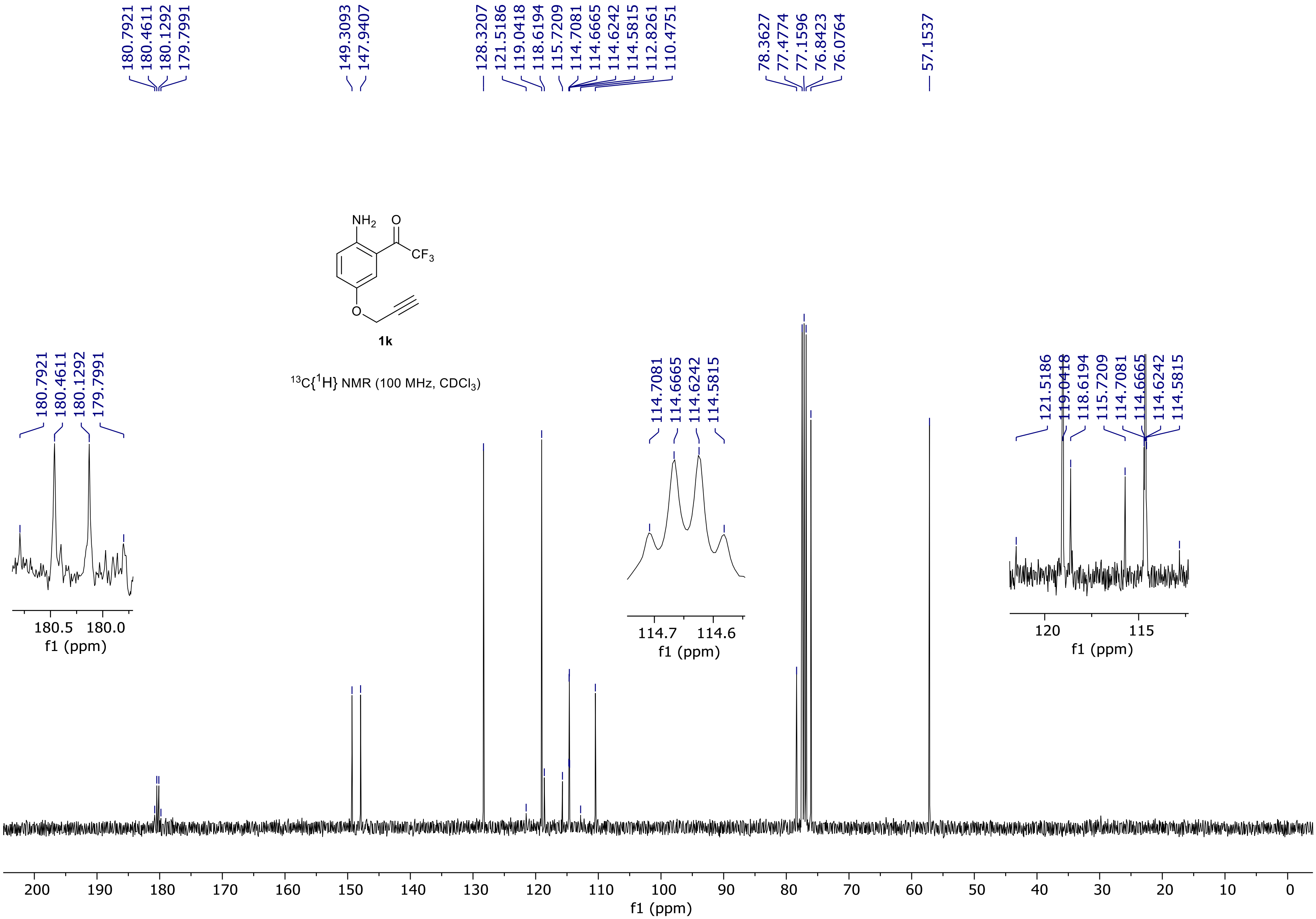
180.7921
180.4611
180.1292
179.7991
149.3093
147.9407
128.3207
121.5186
119.0418
118.6194
115.7209
114.7081
114.6665
114.6242
114.5815
112.8261
110.4751
78.3627
77.4774
77.1596
76.8423
76.0764
57.1537
NH2
O
CF3
O
1k
13C{1H} NMR (100 MHz, CDCl3)
f1 (ppm)

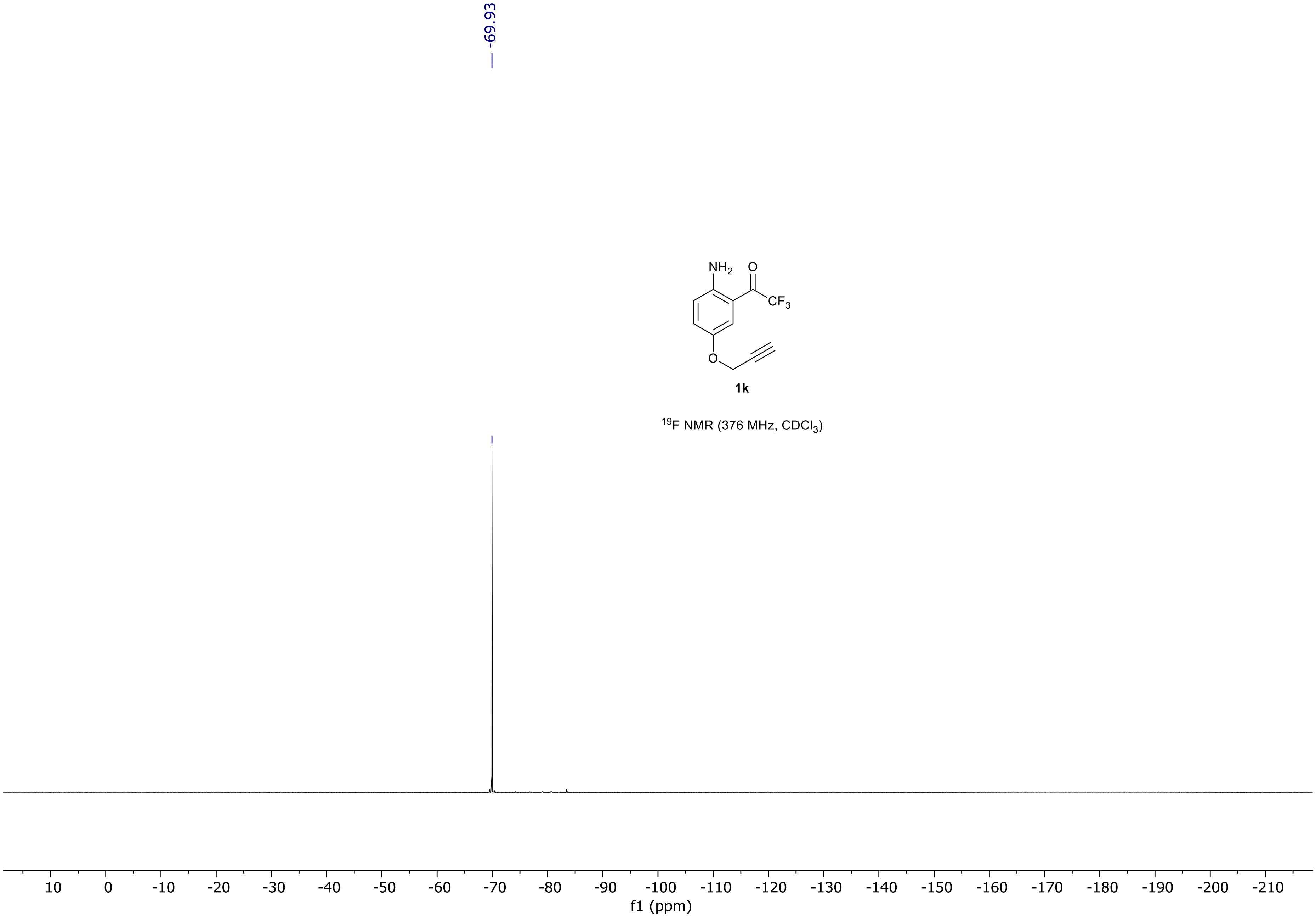
-69.93
NH2
O
CF3
O
1k
19F NMR (376 MHz, CDCl3)
10 0 -10 -20 -30 -40 -50 -60 -70 -80 -90 -100 -110 -120 -130 -140 -150 -160 -170 -180 -190 -200 -210
f1 (ppm)

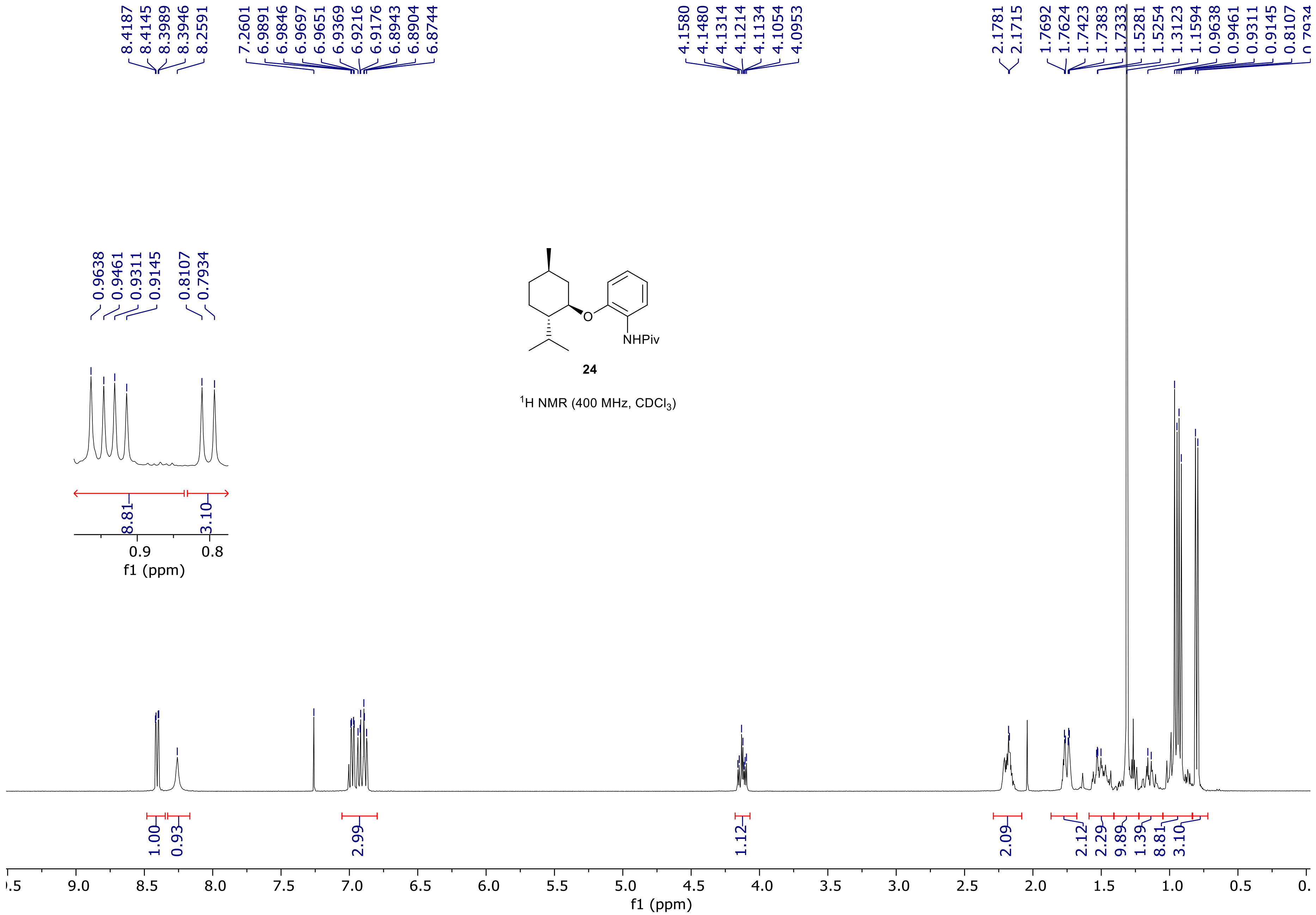

8.4187 8.4145 8.3989 8.3946 8.2591
7.2601 6.9891 6.9846 6.9697 6.9651 6.9369 6.9216 6.9176 6.8943 6.8904 6.8744
4.1580 4.1480 4.1314 4.1214 4.1134 4.1054 4.0953
2.1781 2.1715
1.7692 1.7624 1.7423 1.7383 1.7333 1.5281 1.5254 1.3123 1.1594 0.9638 0.9461 0.9311 0.9145 0.8107 0.7934
0.9638 0.9461 0.9311 0.9145 0.8107 0.7934
8.81 3.10
0.9 0.8
f1 (ppm)
O
NHPiv
24
$^1$H NMR (400 MHz, $CDCl_3$)
1.00 0.93 2.99 1.12 2.09 2.12 2.29 9.89 1.39 8.81 3.10
9.5 9.0 8.5 8.0 7.5 7.0 6.5 6.0 5.5 5.0 4.5 4.0 3.5 3.0 2.5 2.0 1.5 1.0 0.5 0.0
f1 (ppm)

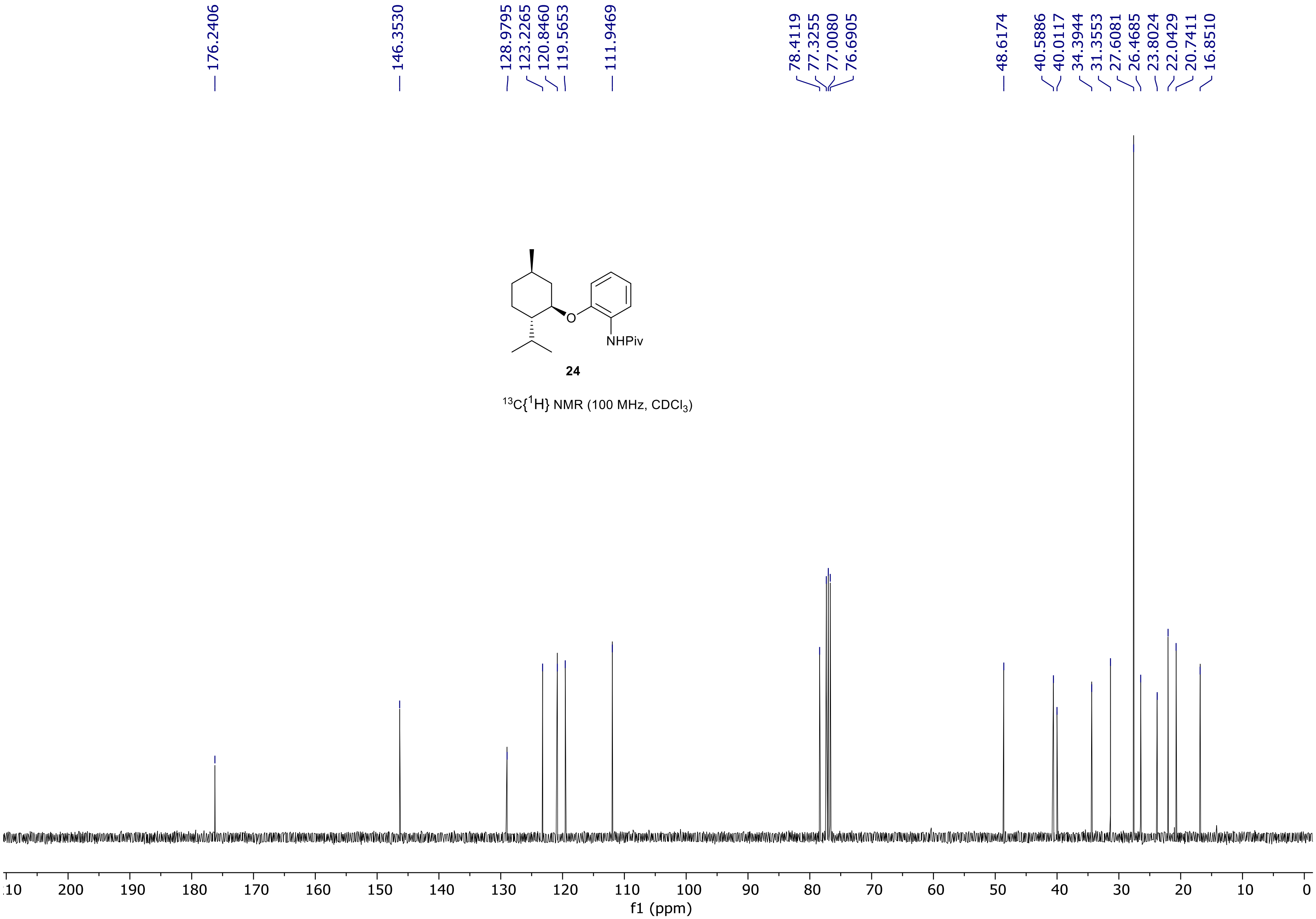


**24**

$^{13}C\{^1H\}$ NMR (100 MHz, $CDCl_3$)

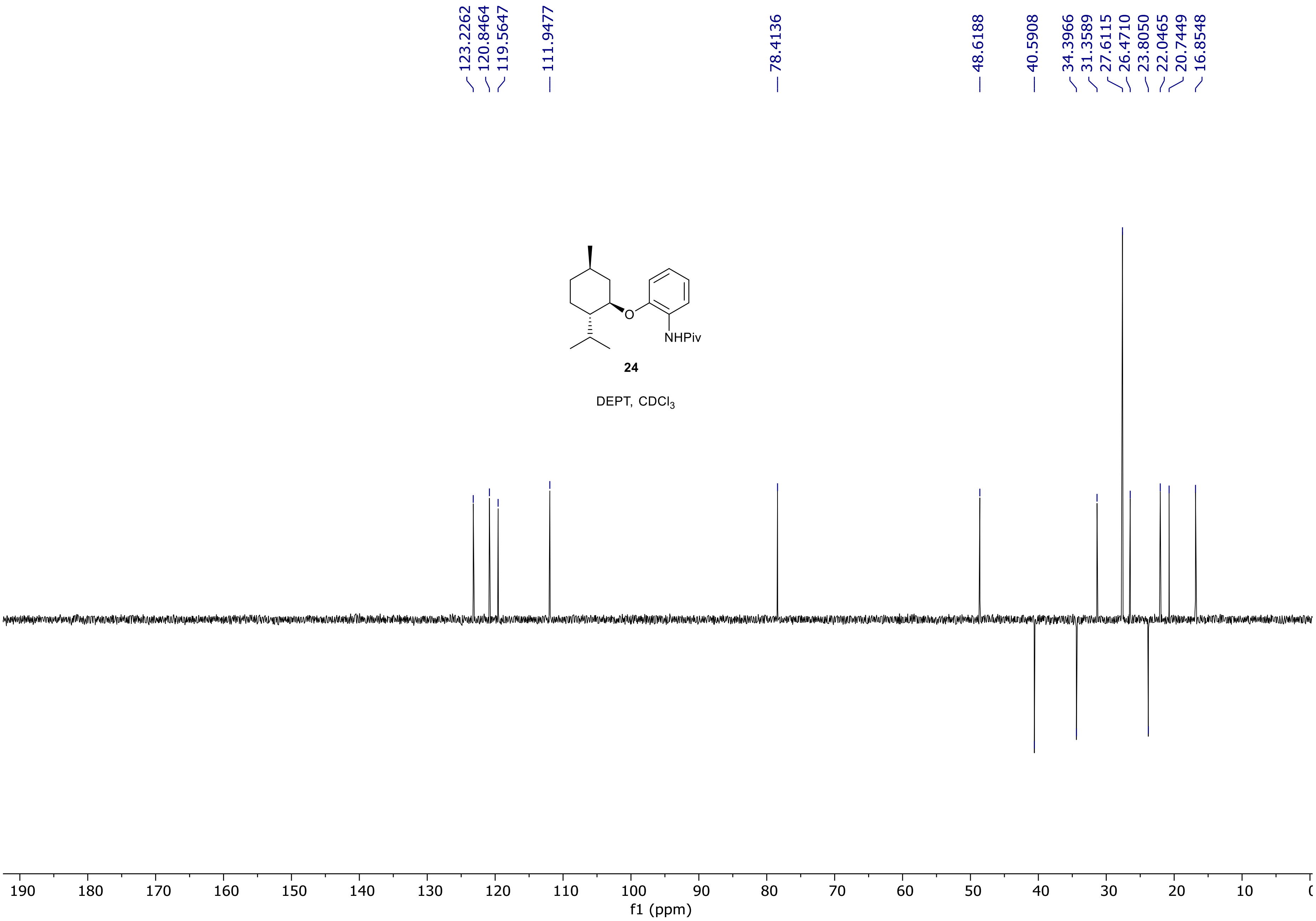
123.2262
120.8464
119.5647
111.9477
78.4136
48.6188
40.5908
34.3966
31.3589
27.6115
26.4710
23.8050
22.0465
20.7449
16.8548
NHPiv
O
24
DEPT, $CDCl_3$
190 180 170 160 150 140 130 120 110 100 90 80 70 60 50 40 30 20 10
f1 (ppm)

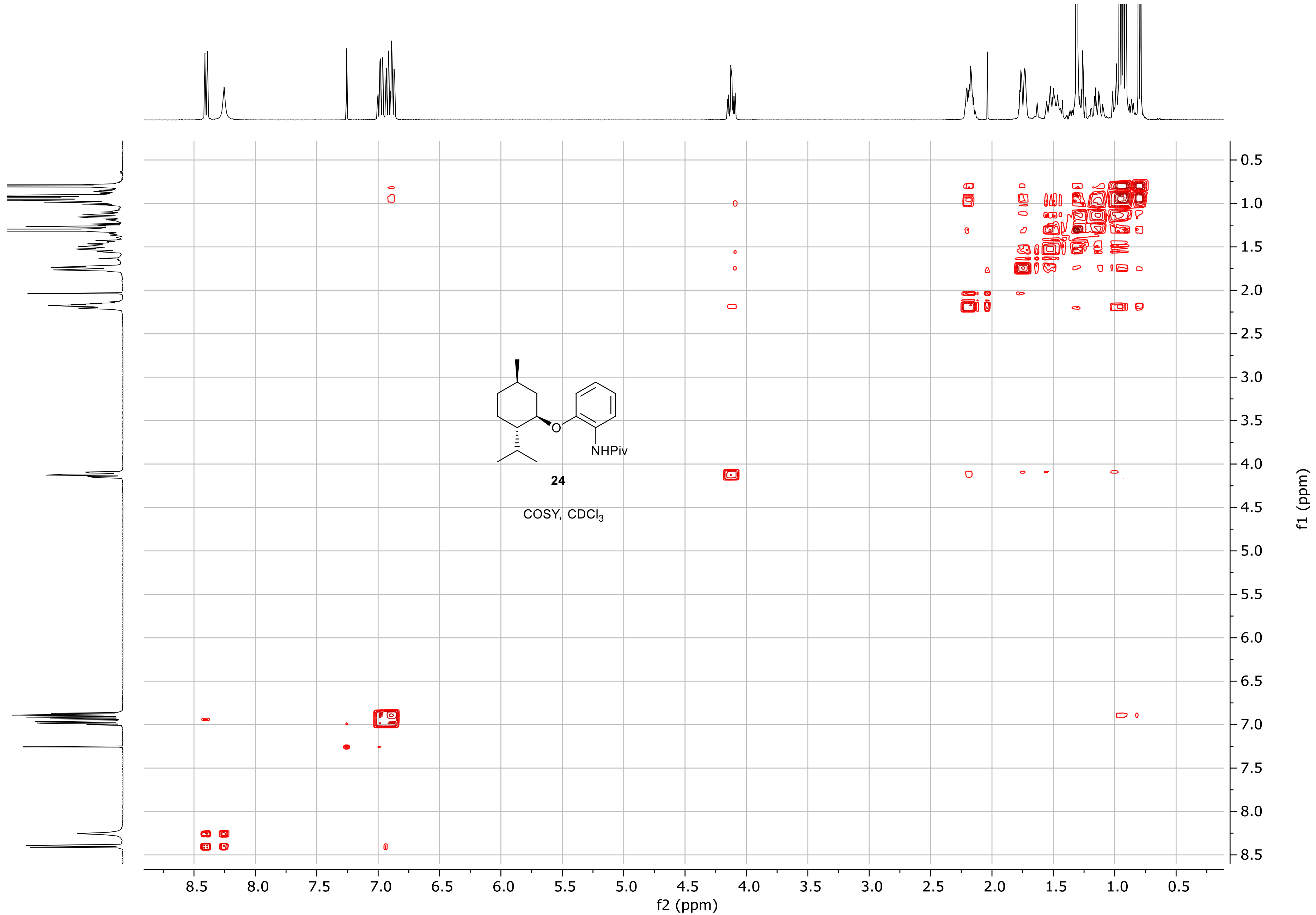
NHPiv
O
24
COSY, CDCl3
f2 (ppm)
f1 (ppm)

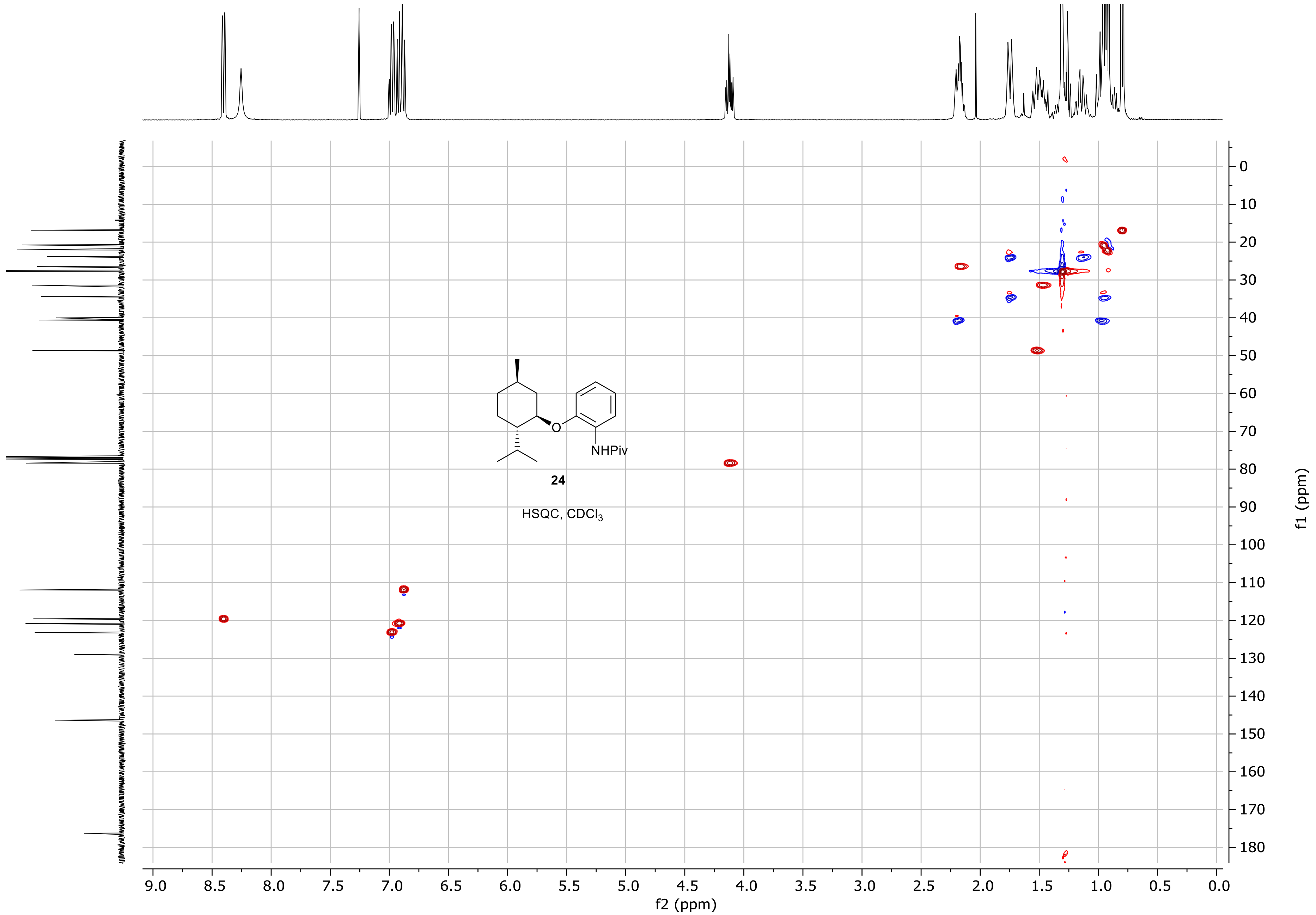

NHPiv
24
HSQC, CDCl3
f2 (ppm)
f1 (ppm)

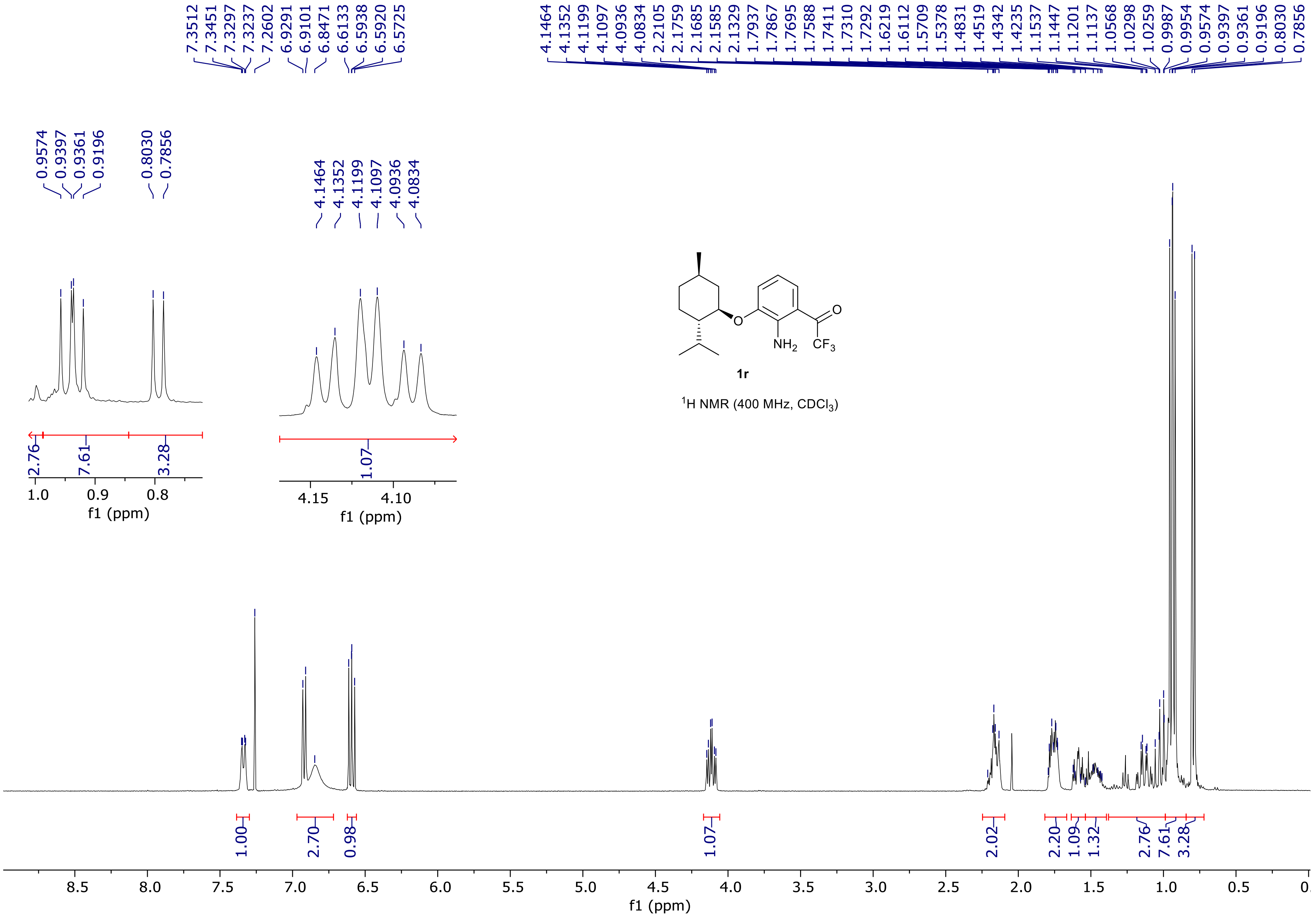
7.3512
7.3451
7.3297
7.3237
7.2602
6.9291
6.9101
6.8471
6.6133
6.5938
6.5920
6.5725
4.1464
4.1352
4.1199
4.1097
4.0936
4.0834
2.2105
2.1759
2.1685
2.1585
2.1329
1.7937
1.7867
1.7695
1.7588
1.7411
1.7310
1.7292
1.6219
1.6112
1.5709
1.5378
1.4831
1.4519
1.4342
1.4235
1.1537
1.1447
1.1201
1.1137
1.0568
1.0298
1.0259
0.9987
0.9954
0.9574
0.9397
0.9361
0.9196
0.8030
0.7856
NH2
O
O
CF3
1r
1H NMR (400 MHz, CDCl3)
1.00
2.70
0.98
1.07
2.02
2.20
1.09
1.32
2.76
7.61
3.28
f1 (ppm)

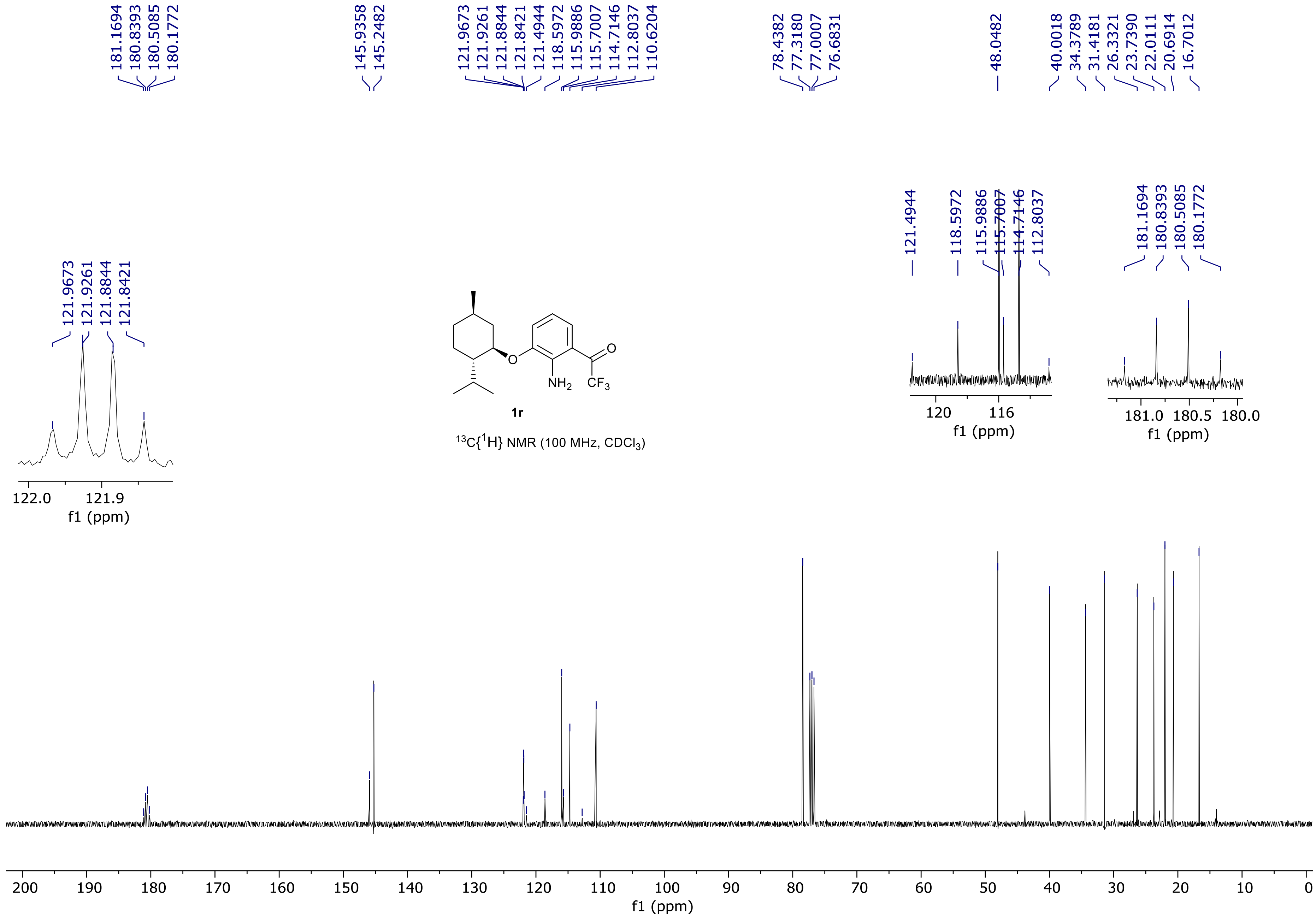
181.1694
180.8393
180.5085
180.1772
145.9358
145.2482
121.9673
121.9261
121.8844
121.8421
121.4944
118.5972
115.9886
115.7007
114.7146
112.8037
110.6204
78.4382
77.3180
77.0007
76.6831
48.0482
40.0018
34.3789
31.4181
26.3321
23.7390
22.0111
20.6914
16.7012
O
NH2
CF3
1r
13C{1H} NMR (100 MHz, CDCl3)
f1 (ppm)

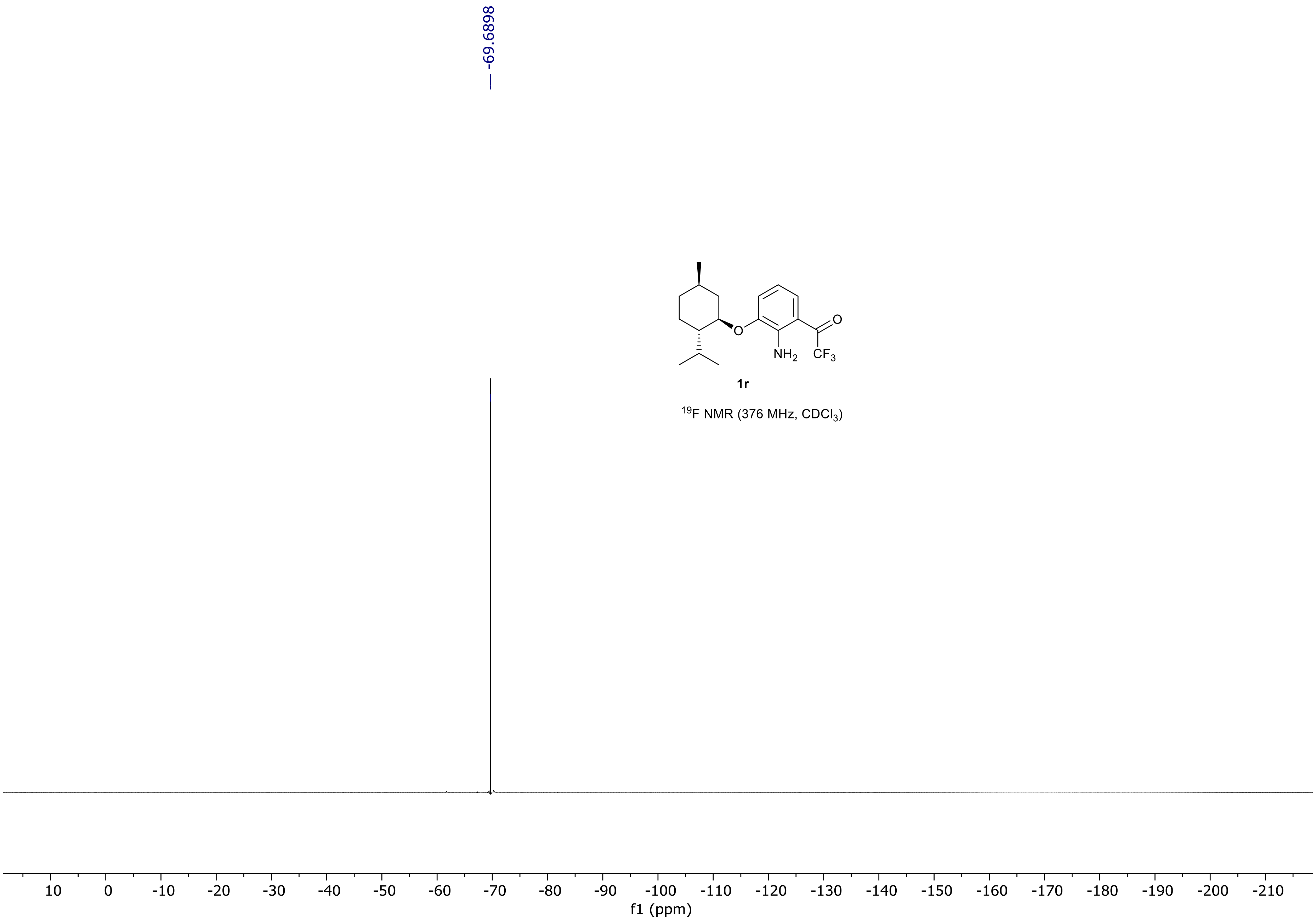
-69.6898
NH2
CF3
O
O
1r
19F NMR (376 MHz, CDCl3)
10
0
-10
-20
-30
-40
-50
-60
-70
-80
-90
-100
-110
-120
-130
-140
-150
-160
-170
-180
-190
-200
-210
f1 (ppm)

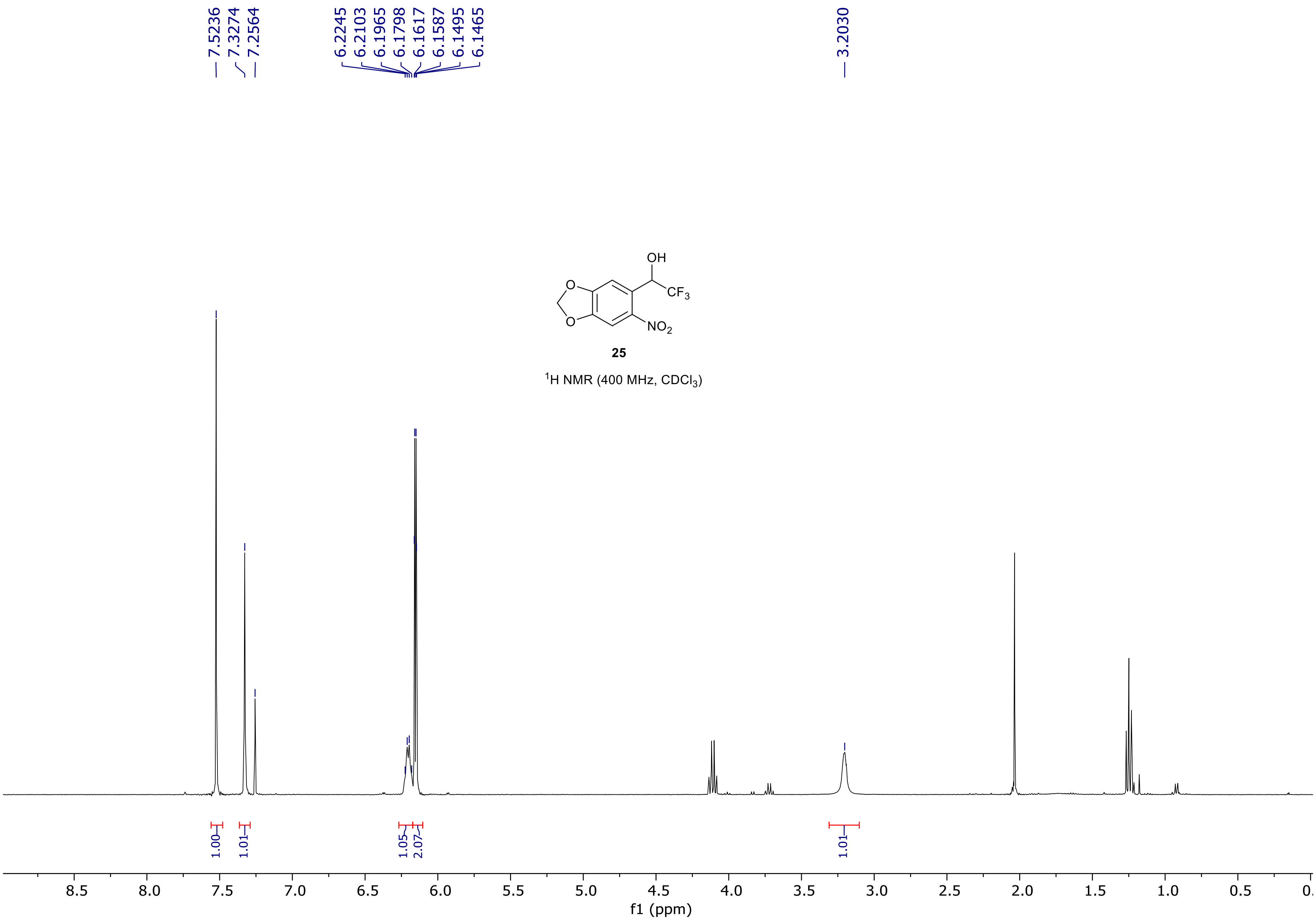


**25**

$^1H$ NMR (400 MHz, $CDCl_3$)

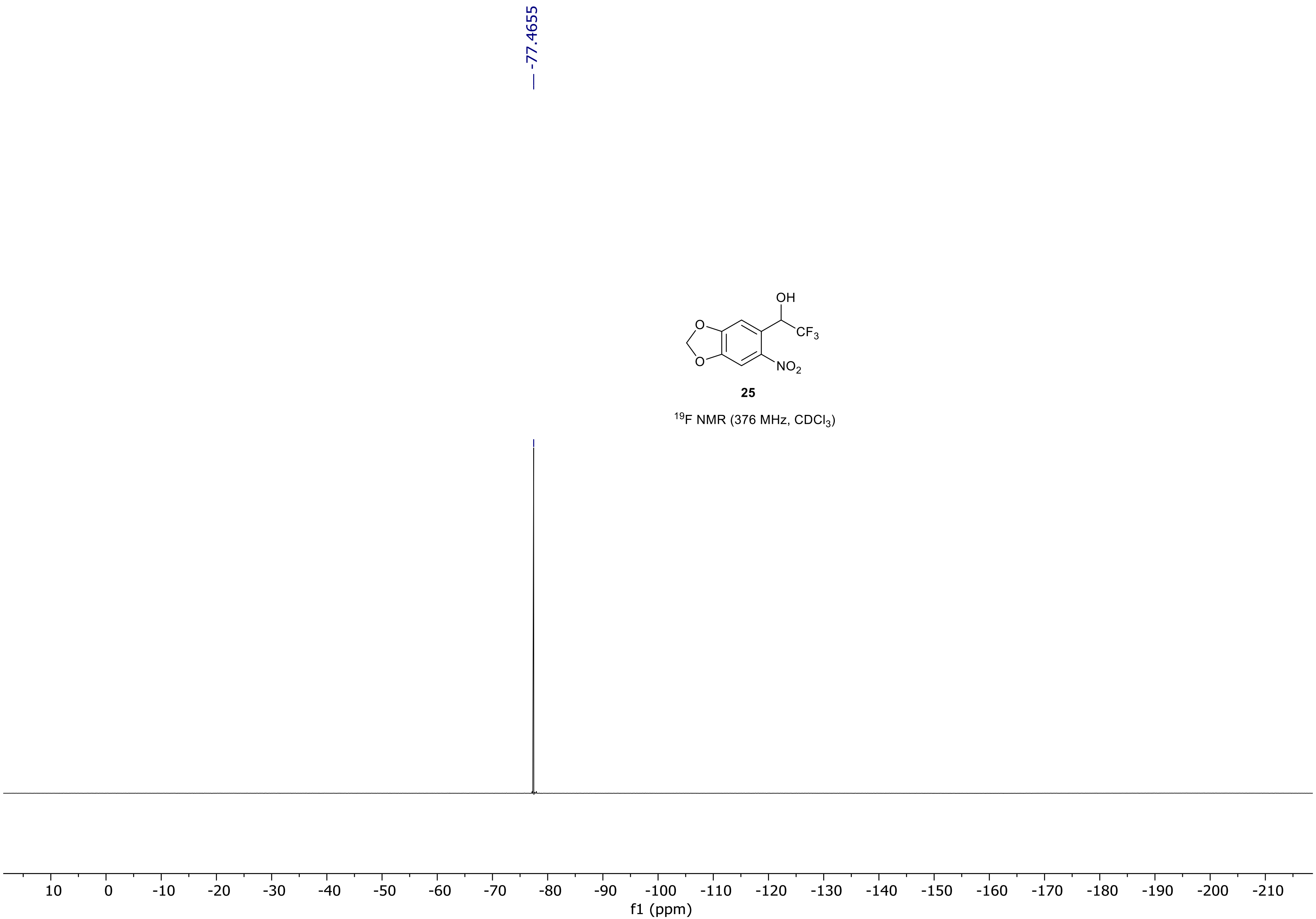
-77.4655
OH
CF3
O
O
NO2
25
19F NMR (376 MHz, CDCl3)
10 0 -10 -20 -30 -40 -50 -60 -70 -80 -90 -100 -110 -120 -130 -140 -150 -160 -170 -180 -190 -200 -210
f1 (ppm)

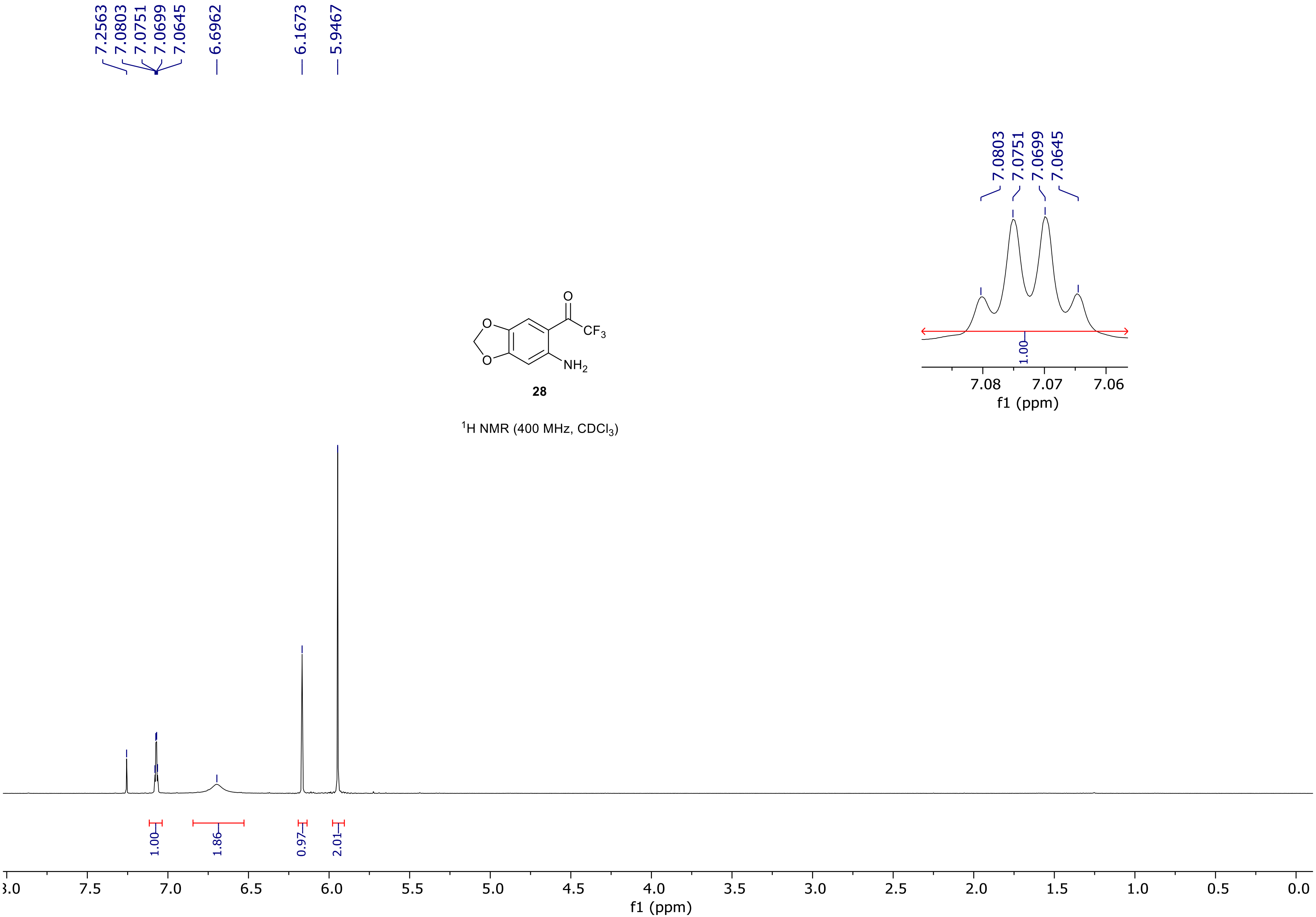
7.2563
7.0803
7.0751
7.0699
7.0645
6.6962
6.1673
5.9467
O
CF3
O
O
NH2
28
1H NMR (400 MHz, CDCl3)
1.00
7.08
7.07
7.06
f1 (ppm)
1.00
1.86
0.97
2.01
8.0
7.5
7.0
6.5
6.0
5.5
5.0
4.5
4.0
3.5
3.0
2.5
2.0
1.5
1.0
0.5
0.0
f1 (ppm)

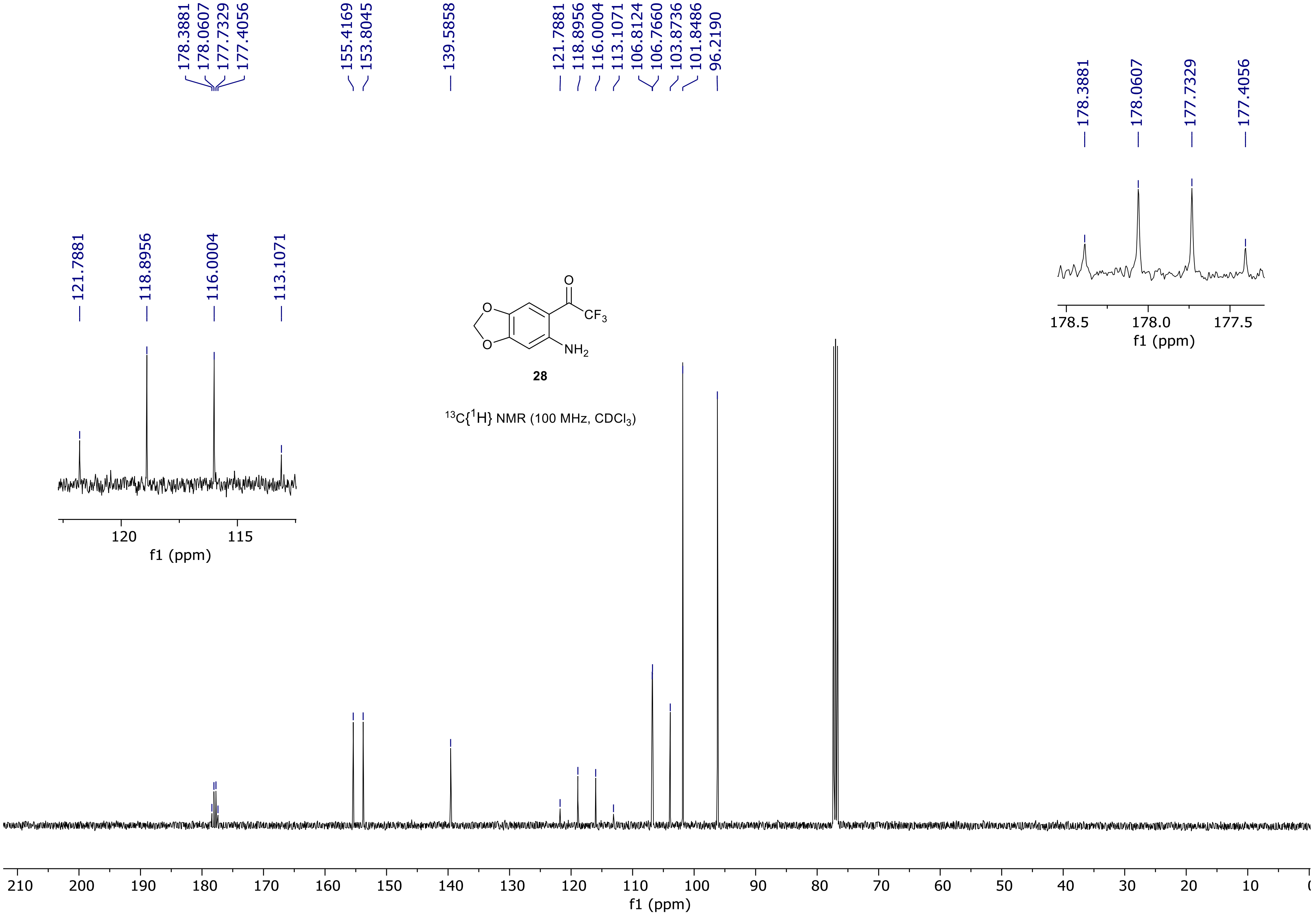


**28**

$^{13}C\{^{1}H\}$ NMR (100 MHz, $CDCl_3$)

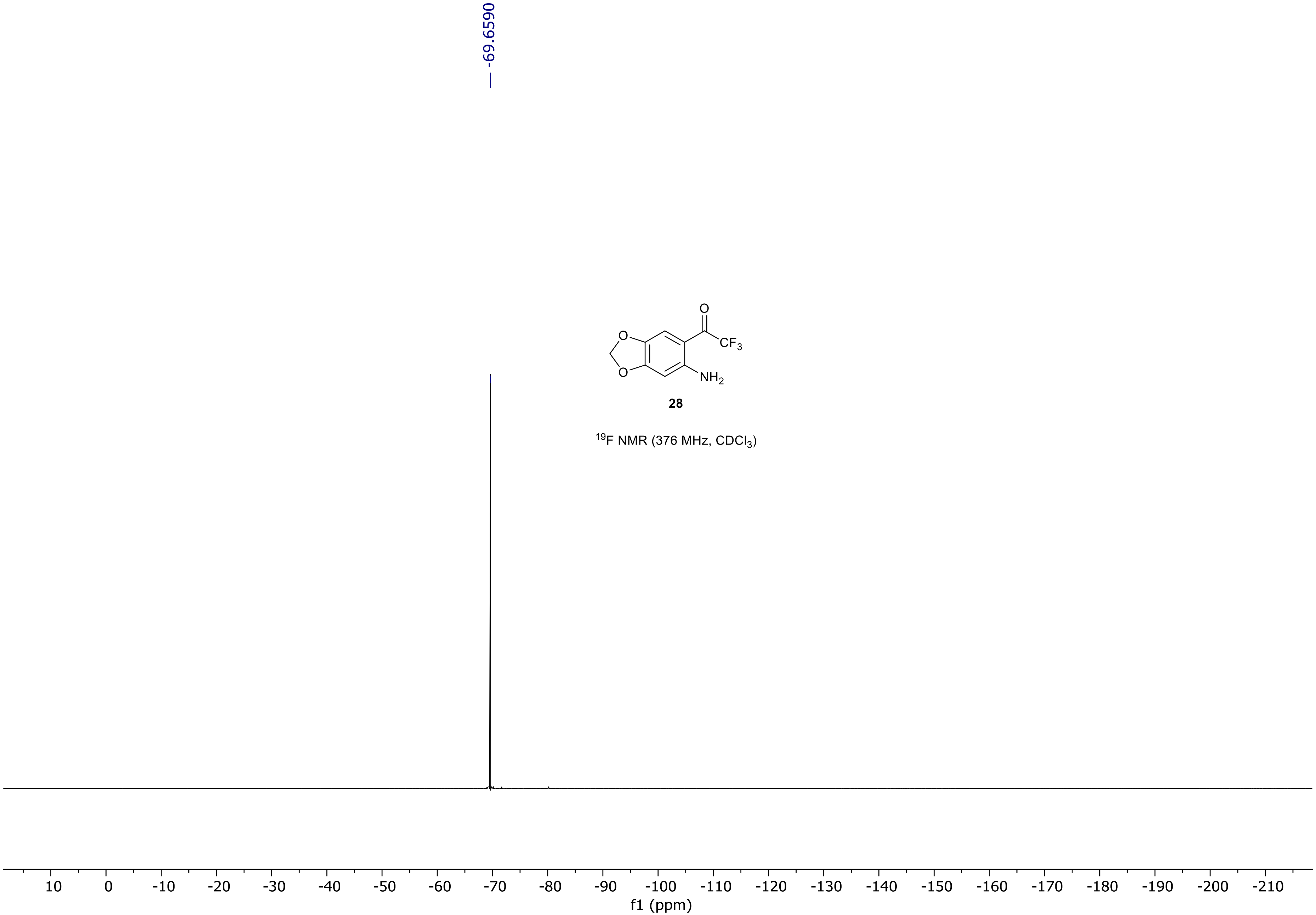
-69.6590
O
CF3
O
O
NH2
28
19F NMR (376 MHz, CDCl3)
10 0 -10 -20 -30 -40 -50 -60 -70 -80 -90 -100 -110 -120 -130 -140 -150 -160 -170 -180 -190 -200 -210
f1 (ppm)

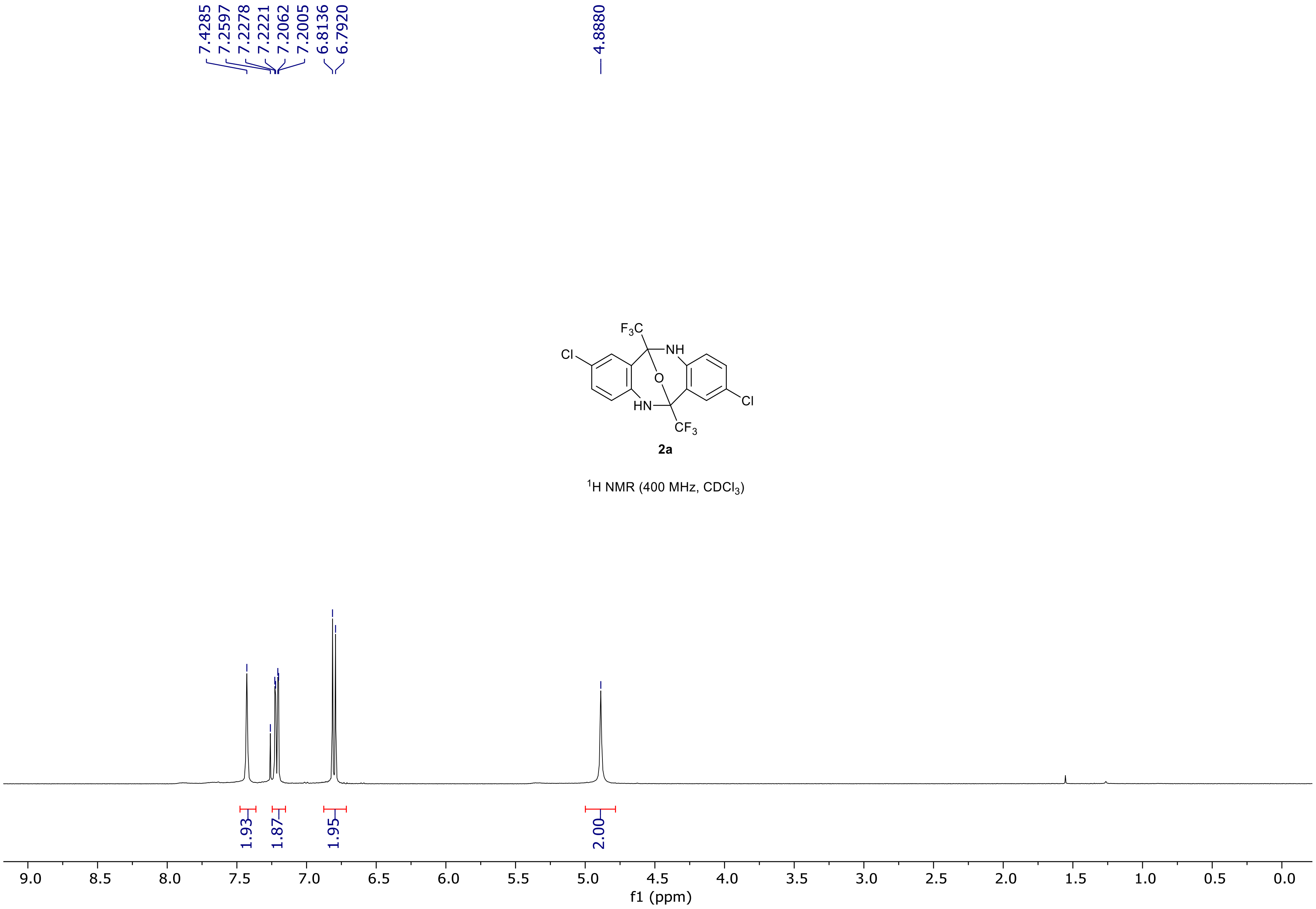


**2a**

$^1$H NMR (400 MHz, $CDCl_3$)

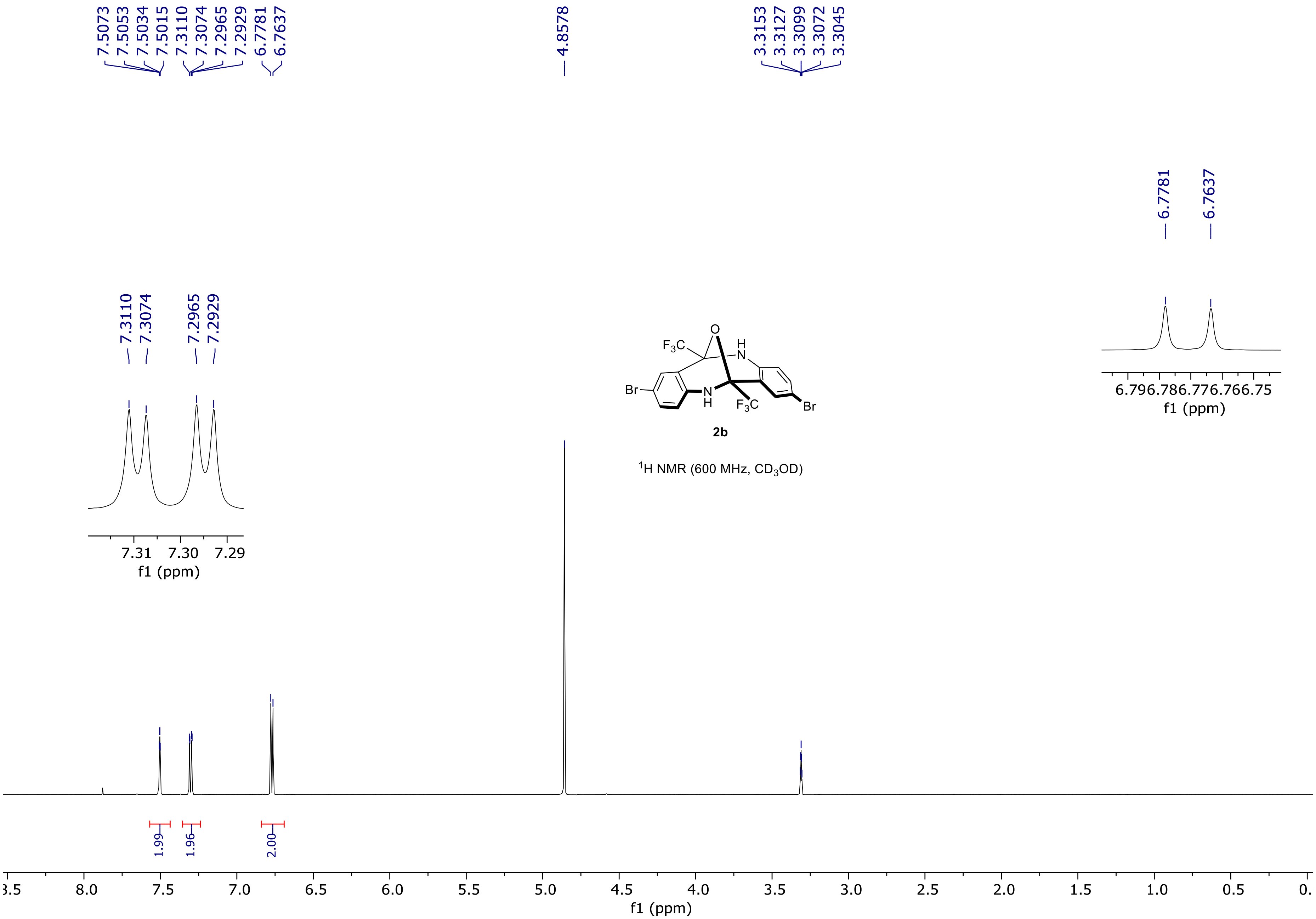
7.5073
7.5053
7.5034
7.5015
7.3110
7.3074
7.2965
7.2929
6.7781
6.7637
4.8578
3.3153
3.3127
3.3099
3.3072
3.3045
Br
F$_3$C
O
H
N
N
H
F$_3$C
Br
2b
$^1$H NMR (600 MHz, CD$_3$OD)
1.99
1.96
2.00
f1 (ppm)

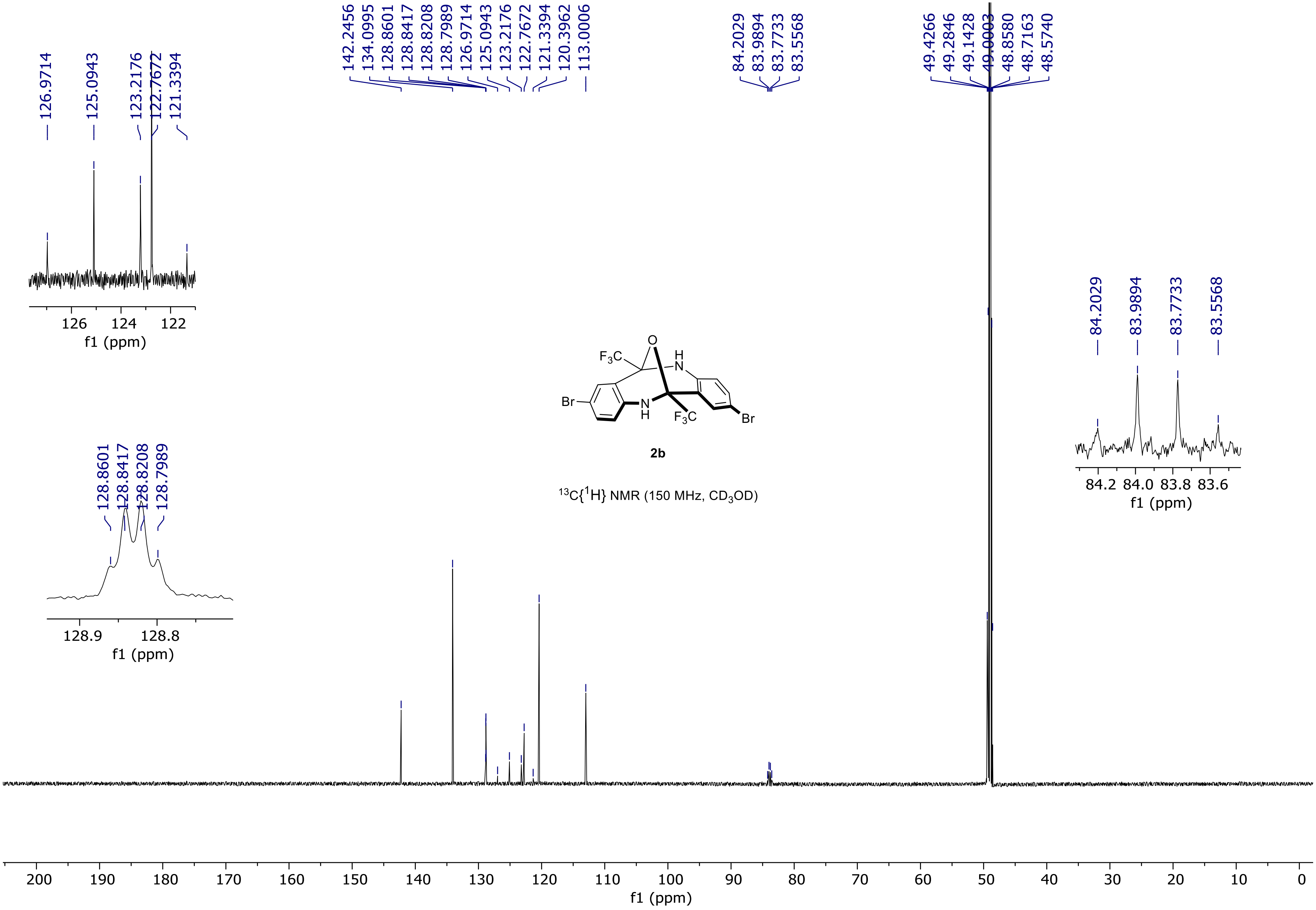
142.2456
134.0995
128.8601
128.8417
128.8208
128.7989
126.9714
125.0943
123.2176
122.7672
121.3394
120.3962
113.0006
84.2029
83.9894
83.7733
83.5568
49.4266
49.2846
49.1428
49.0003
48.8580
48.7163
48.5740
f1 (ppm)
F3C
O
H
N
Br
N
H
F3C
Br
2b
13C{1H} NMR (150 MHz, CD3OD)

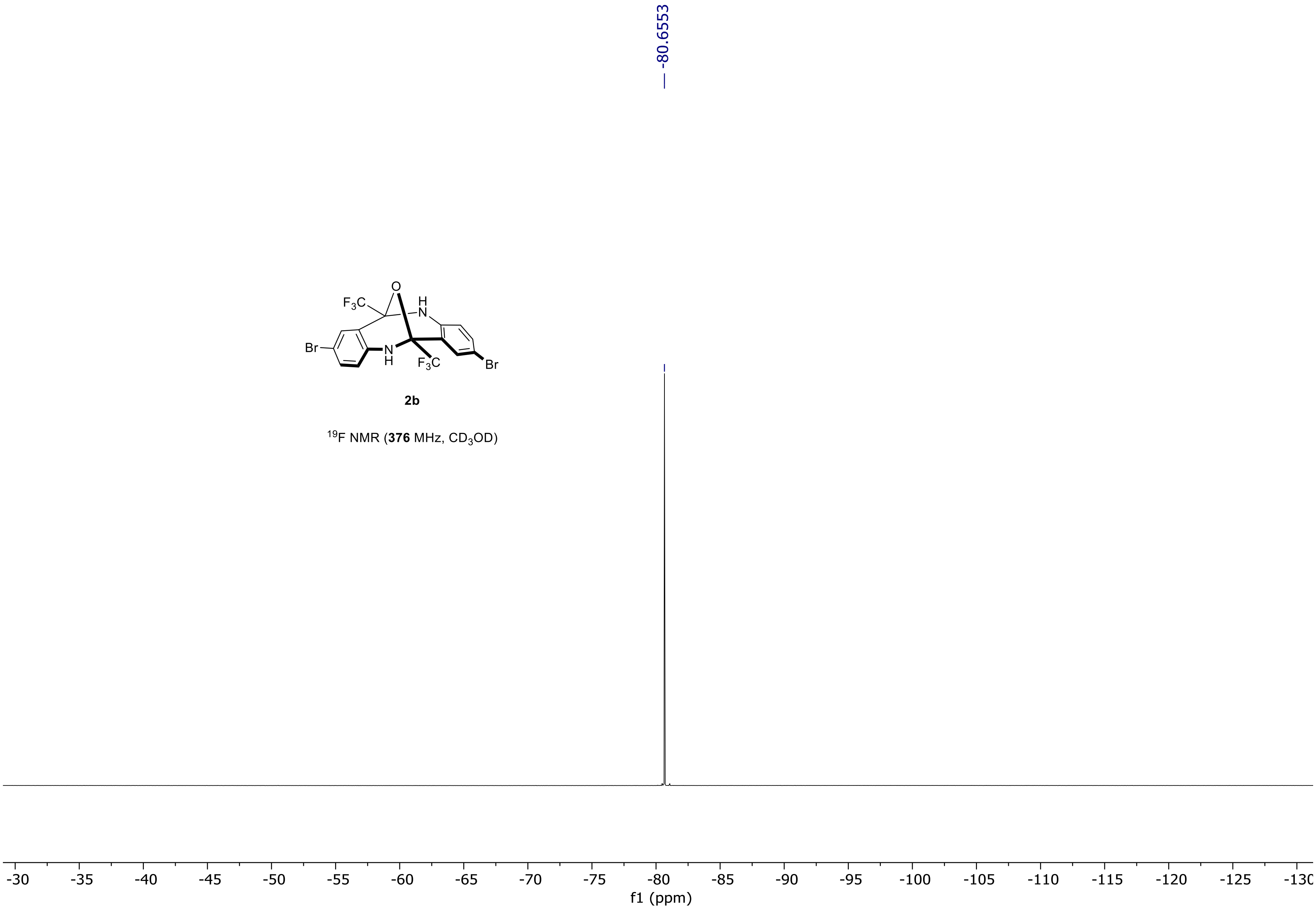
-80.6553
F3C
O
H
N
Br
N
H
F3C
Br
2b
19F NMR (376 MHz, CD3OD)
-30
-35
-40
-45
-50
-55
-60
-65
-70
-75
-80
-85
-90
-95
-100
-105
-110
-115
-120
-125
-130
f1 (ppm)

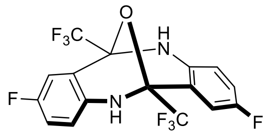


**2c**

$^1$H NMR (400 MHz, CDCl$_3$)

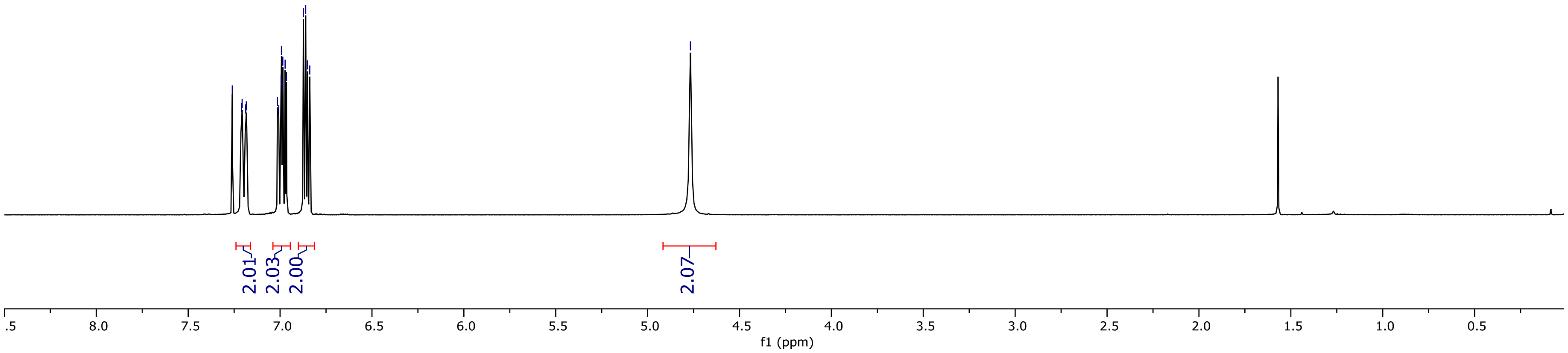

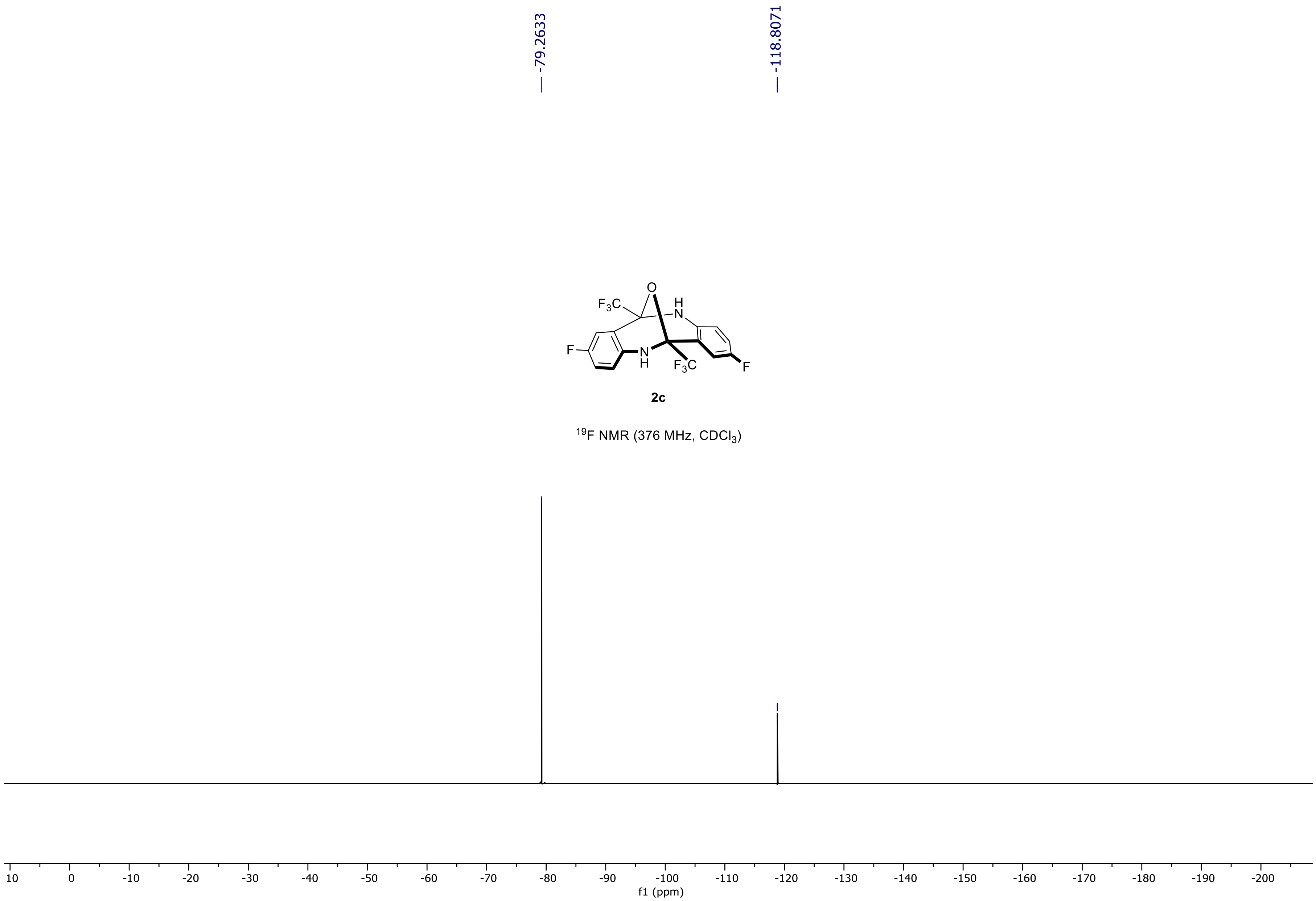


**2c**

$^{19}F$ NMR (376 MHz, $CDCl_3$)

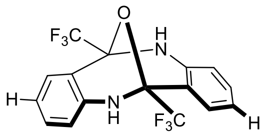


**2d**

$^{1}$H NMR (400 MHz, $CDCl_3$)

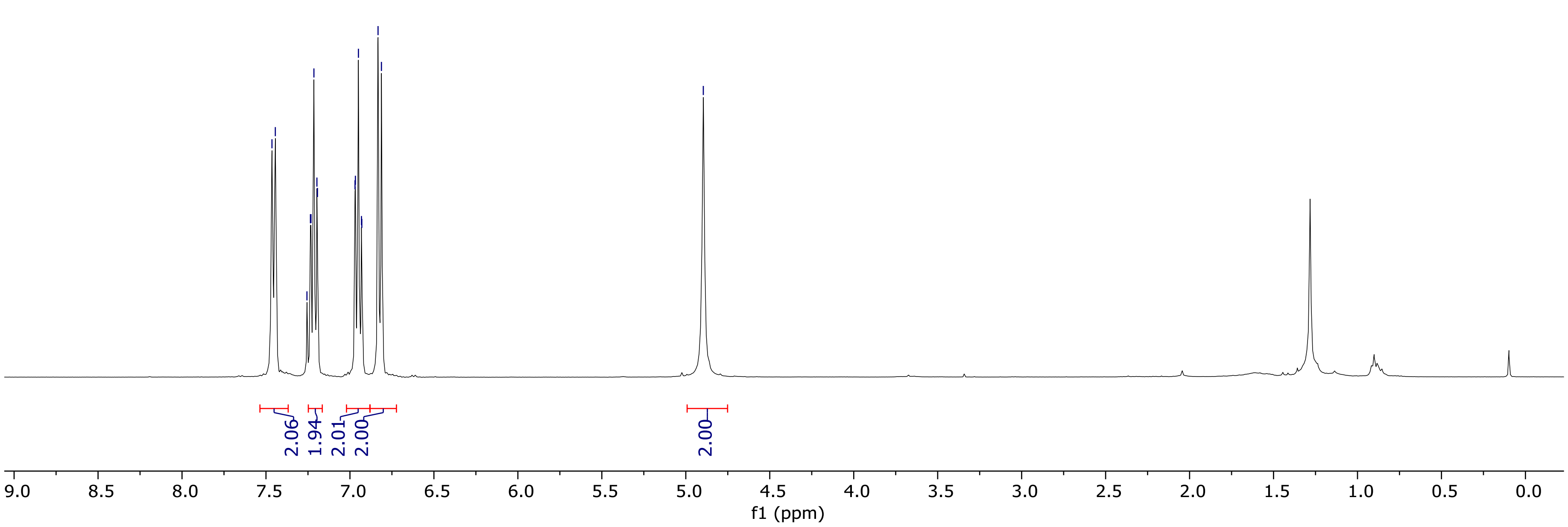

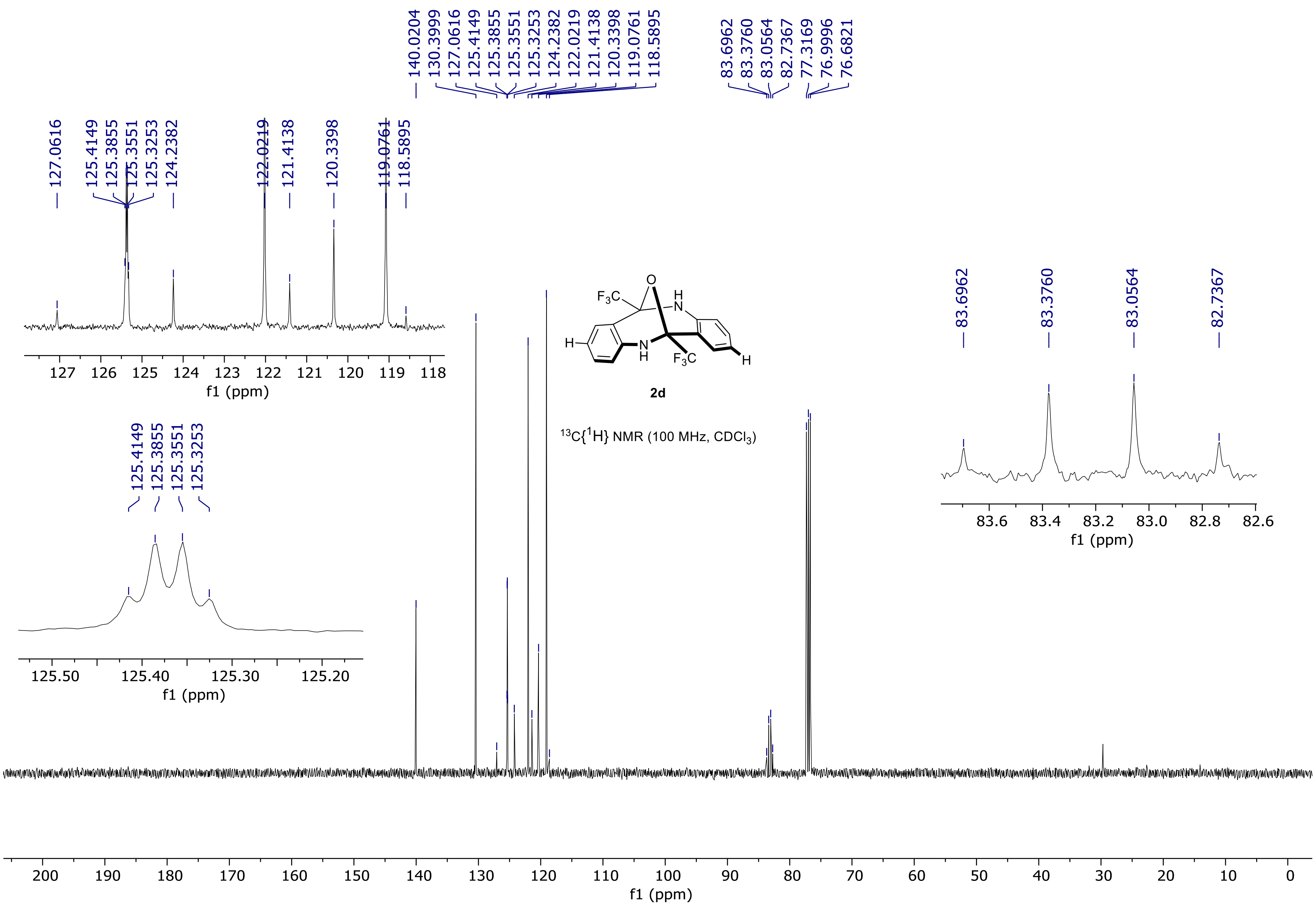
2d
$^{13}C\{^1H\}$ NMR (100 MHz, $CDCl_3$)
140.0204
130.3999
127.0616
125.4149
125.3855
125.3551
125.3253
124.2382
122.0219
121.4138
120.3398
119.0761
118.5895
83.6962
83.3760
83.0564
82.7367
77.3169
76.9996
76.6821
f1 (ppm)

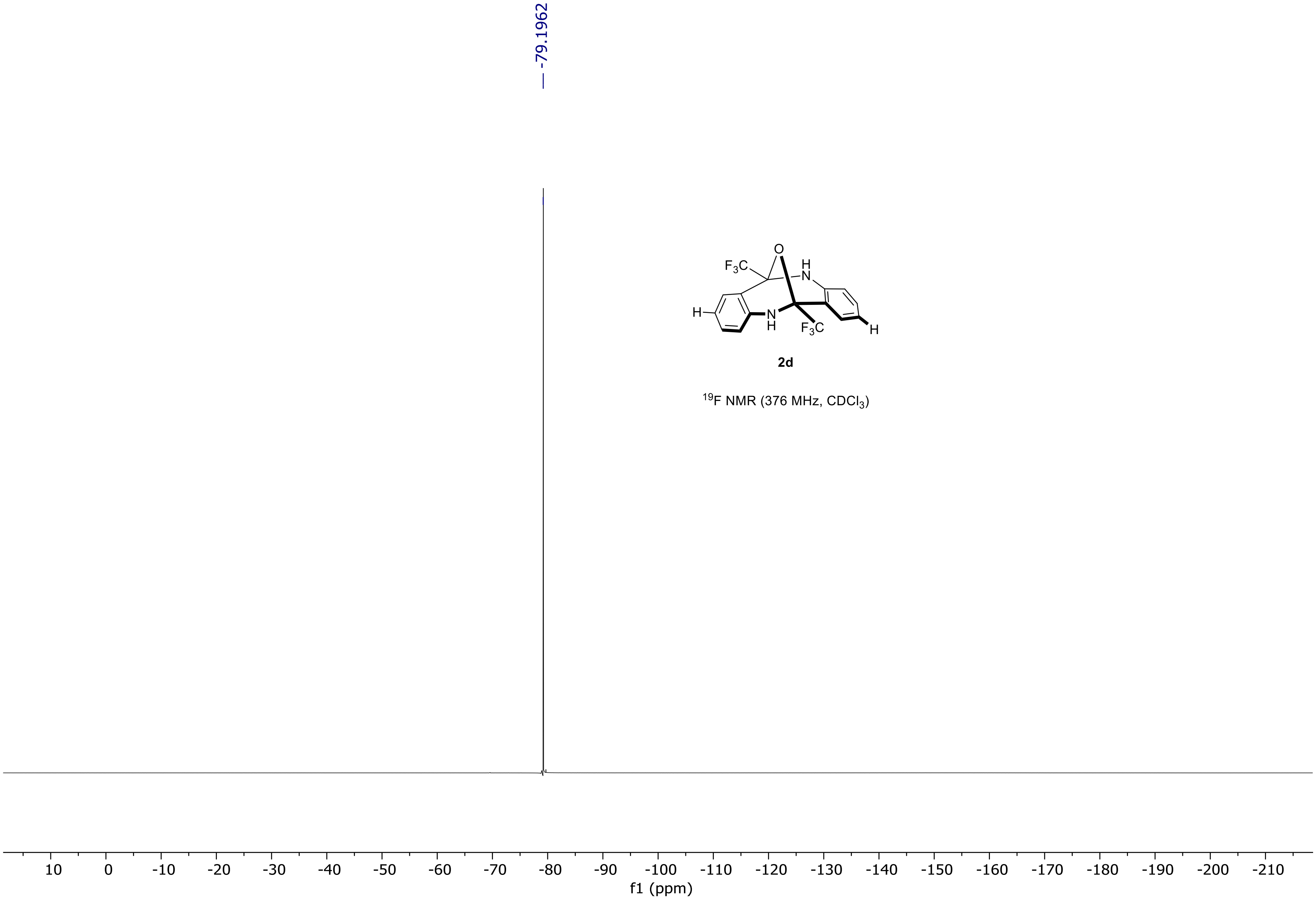
-79.1962
F3C
O
H
N
H
N
H
F3C
H
2d
19F NMR (376 MHz, CDCl3)
10 0 -10 -20 -30 -40 -50 -60 -70 -80 -90 -100 -110 -120 -130 -140 -150 -160 -170 -180 -190 -200 -210
f1 (ppm)

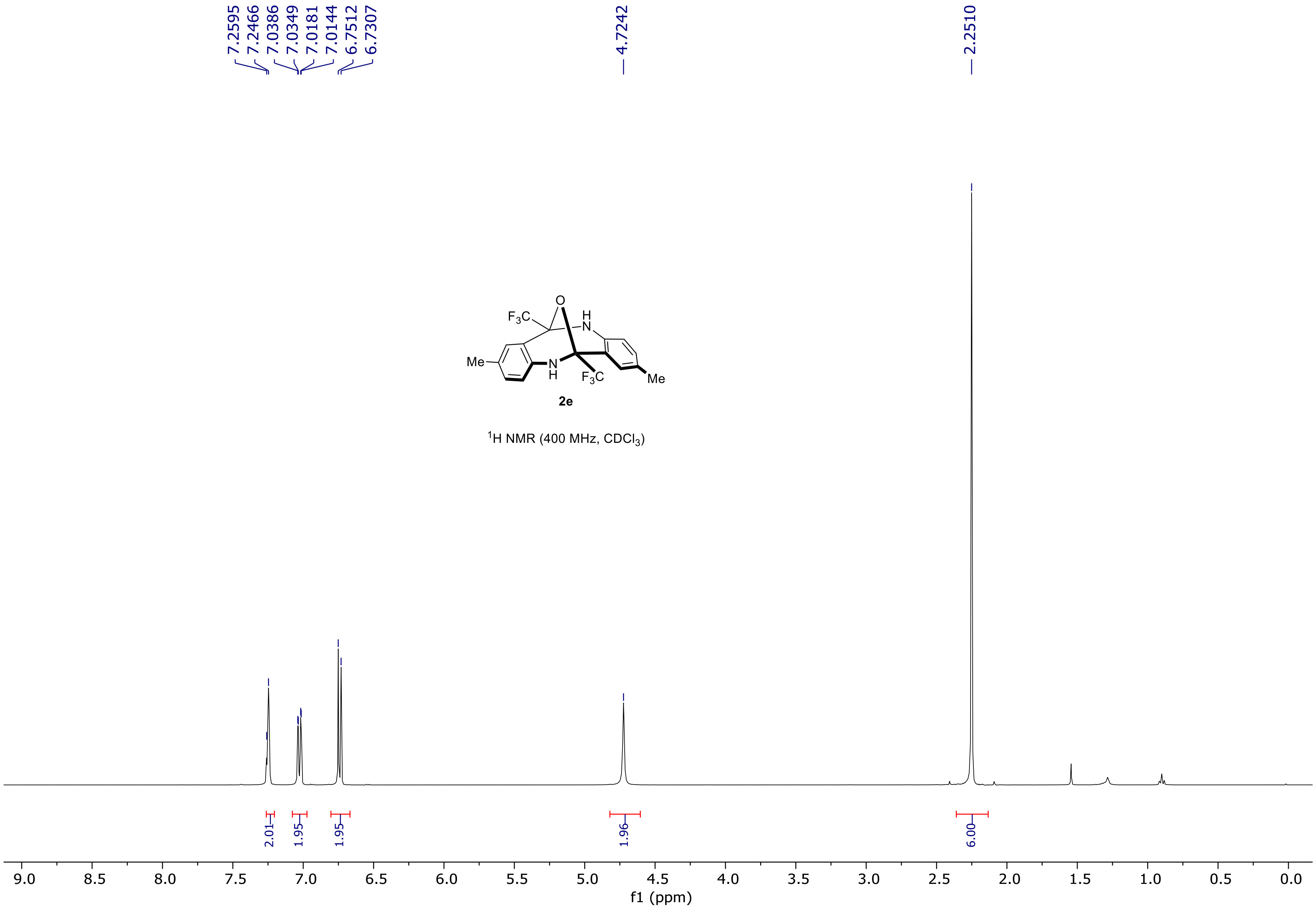
7.2595
7.2466
7.0386
7.0349
7.0181
7.0144
6.7512
6.7307
4.7242
2.2510
2e
1H NMR (400 MHz, CDCl3)
2.01
1.95
1.95
1.96
6.00
9.0 8.5 8.0 7.5 7.0 6.5 6.0 5.5 5.0 4.5 4.0 3.5 3.0 2.5 2.0 1.5 1.0 0.5 0.0
f1 (ppm)

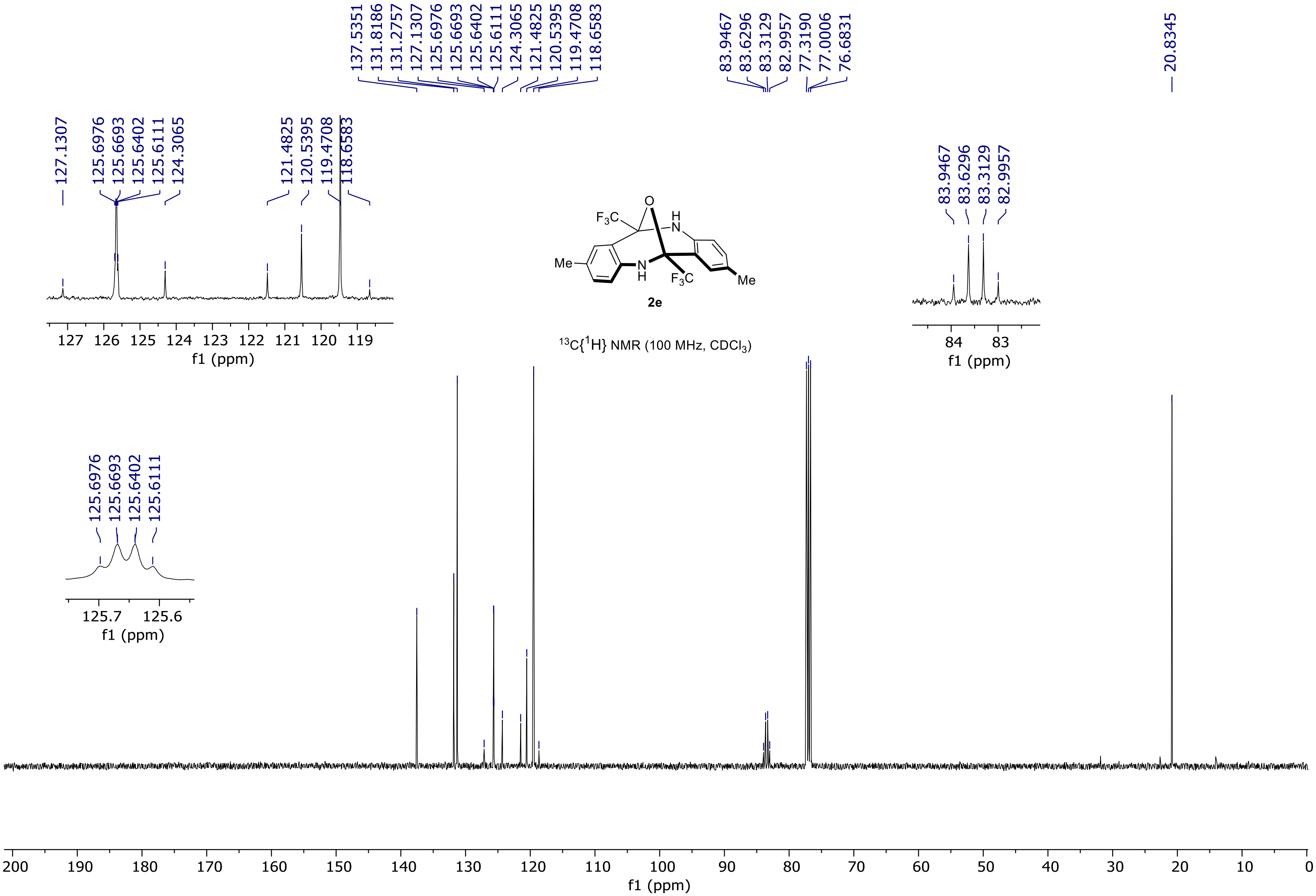
137.5351
131.8186
131.2757
127.1307
125.6976
125.6693
125.6402
125.6111
124.3065
121.4825
120.5395
119.4708
118.6583
83.9467
83.6296
83.3129
82.9957
77.3190
77.0006
76.6831
20.8345
O
$F_3C$
H
N
Me
N
H
$F_3C$
Me
2e
$^{13}C\{^1H\}$ NMR (100 MHz, $CDCl_3$)
127 126 125 124 123 122 121 120 119
f1 (ppm)
84 83
f1 (ppm)
125.7 125.6
f1 (ppm)
200 190 180 170 160 150 140 130 120 110 100 90 80 70 60 50 40 30 20 10 0
f1 (ppm)

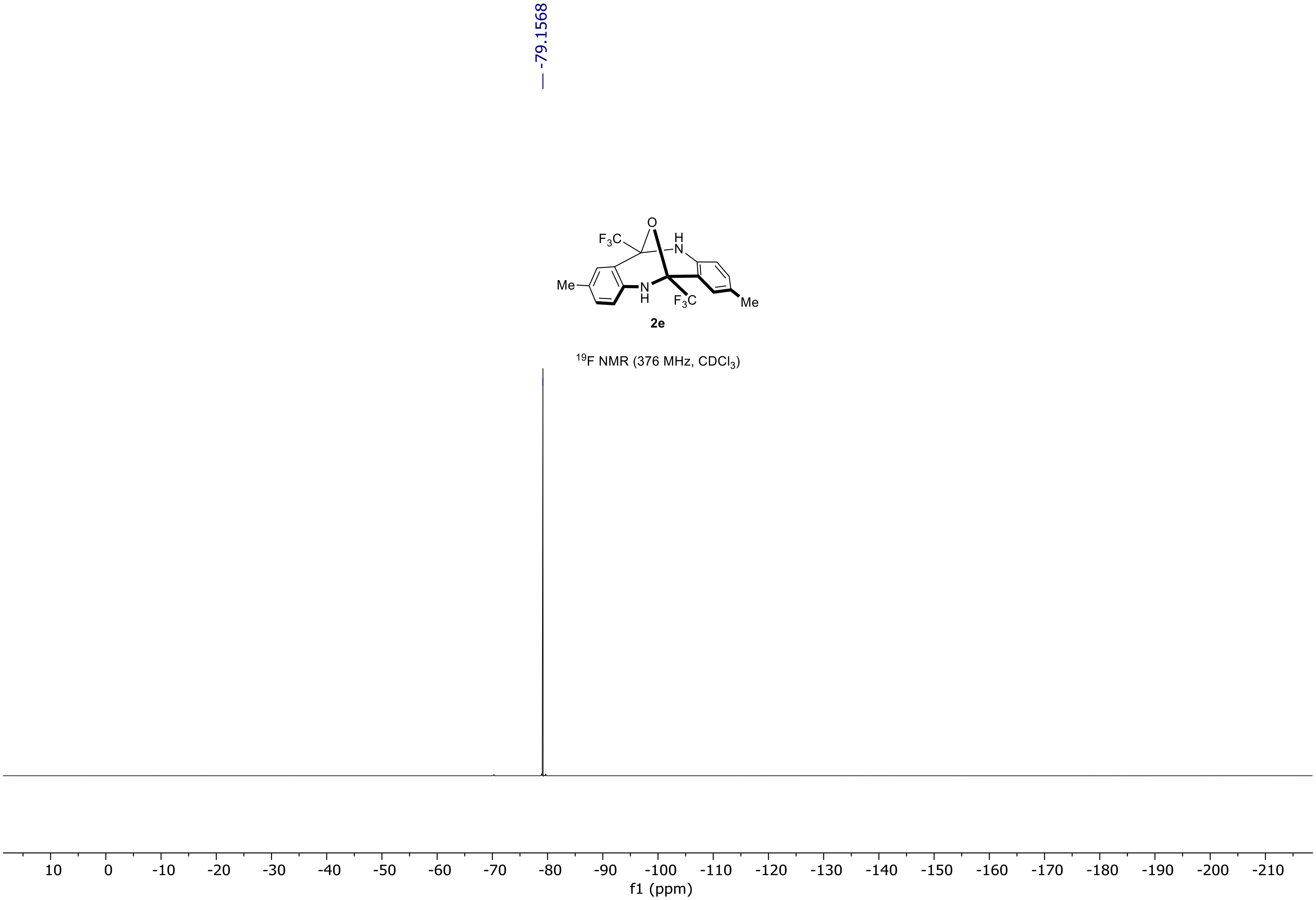
-79.1568
O
F3C
H
N
Me
N
H
F3C
Me
2e
19F NMR (376 MHz, CDCl3)
10 0 -10 -20 -30 -40 -50 -60 -70 -80 -90 -100 -110 -120 -130 -140 -150 -160 -170 -180 -190 -200 -210
f1 (ppm)

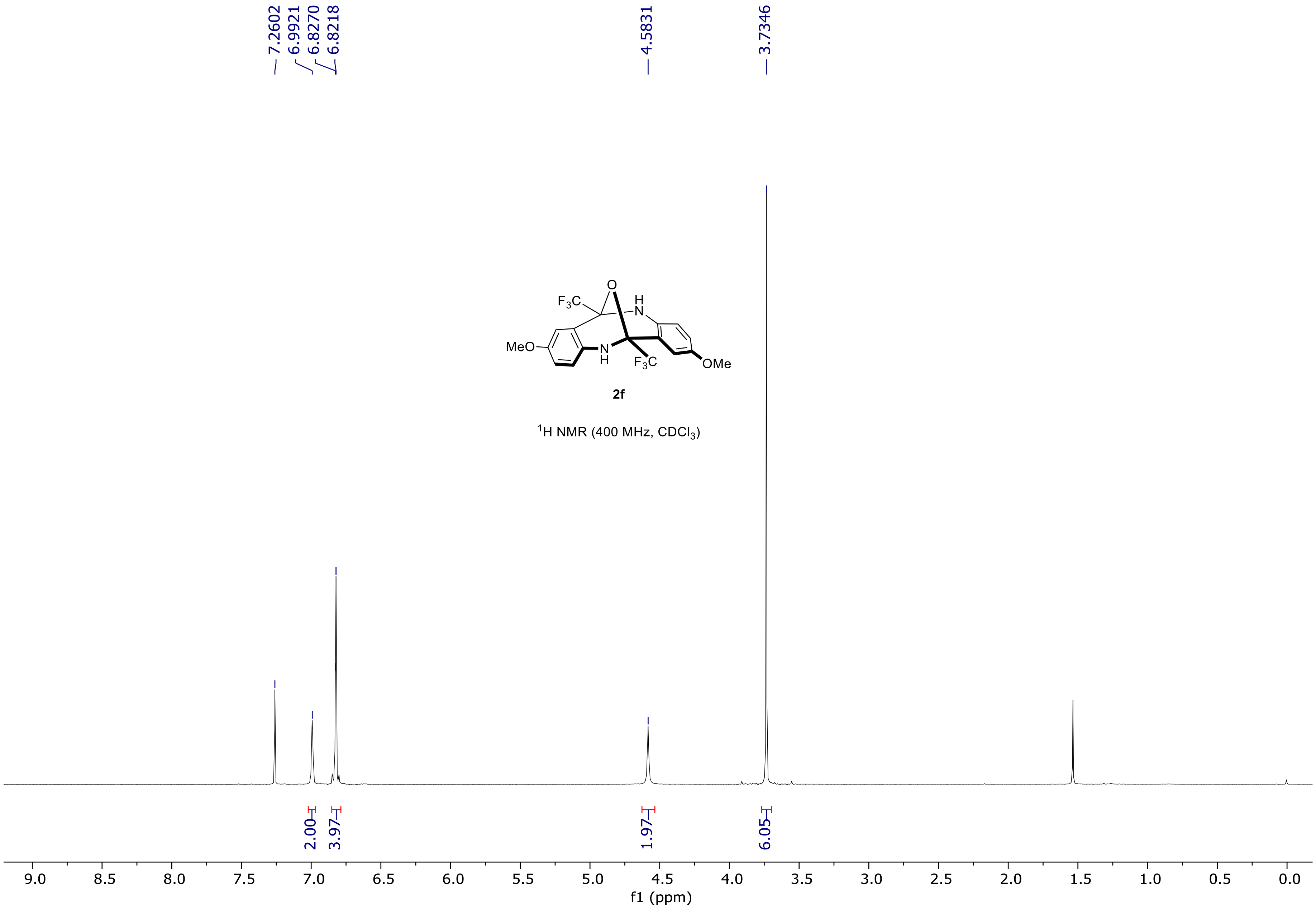


**2f**

$^1$H NMR (400 MHz, $CDCl_3$)

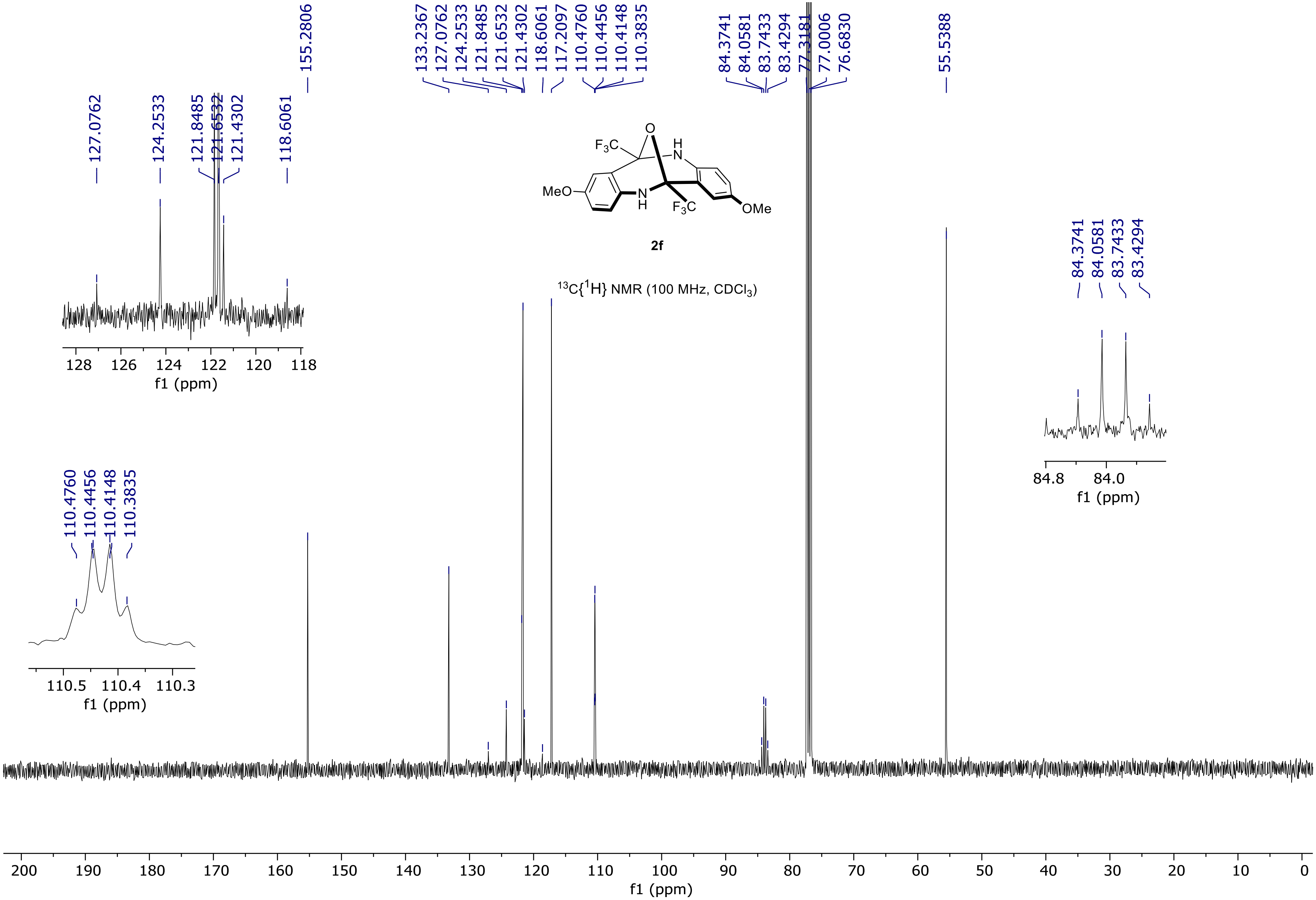

155.2806
133.2367
127.0762
124.2533
121.8485
121.6532
121.4302
118.6061
117.2097
110.4760
110.4456
110.4148
110.3835
84.3741
84.0581
83.7433
83.4294
77.3181
77.0006
76.6830
55.5388
F3C
O
H
N
MeO
N
H
F3C
OMe
2f
13C{1H} NMR (100 MHz, CDCl3)
f1 (ppm)

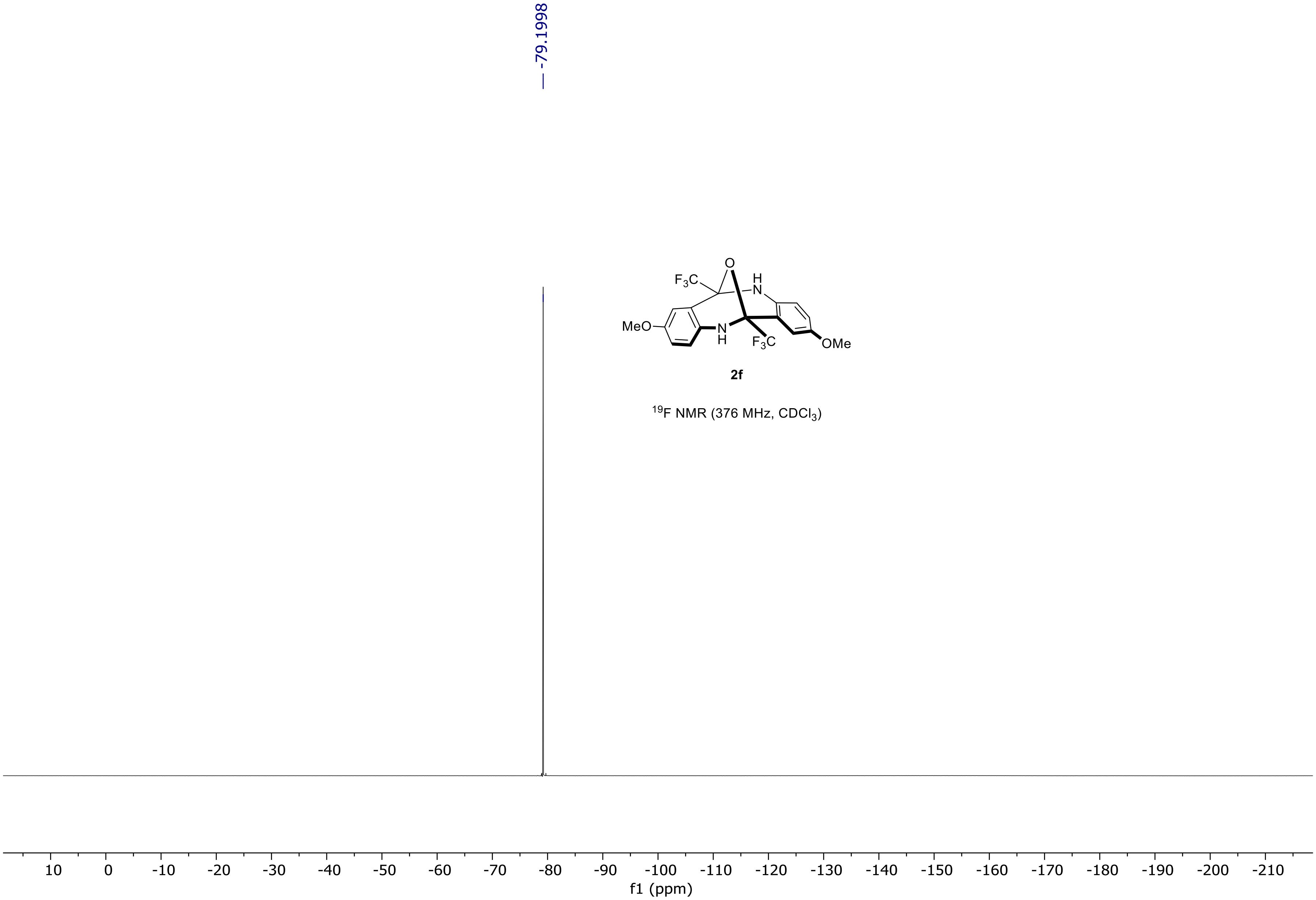

-79.1998
F3C
O
H
N
MeO
N
H
F3C
OMe
2f
19F NMR (376 MHz, CDCl3)
10 0 -10 -20 -30 -40 -50 -60 -70 -80 -90 -100 -110 -120 -130 -140 -150 -160 -170 -180 -190 -200 -210
f1 (ppm)

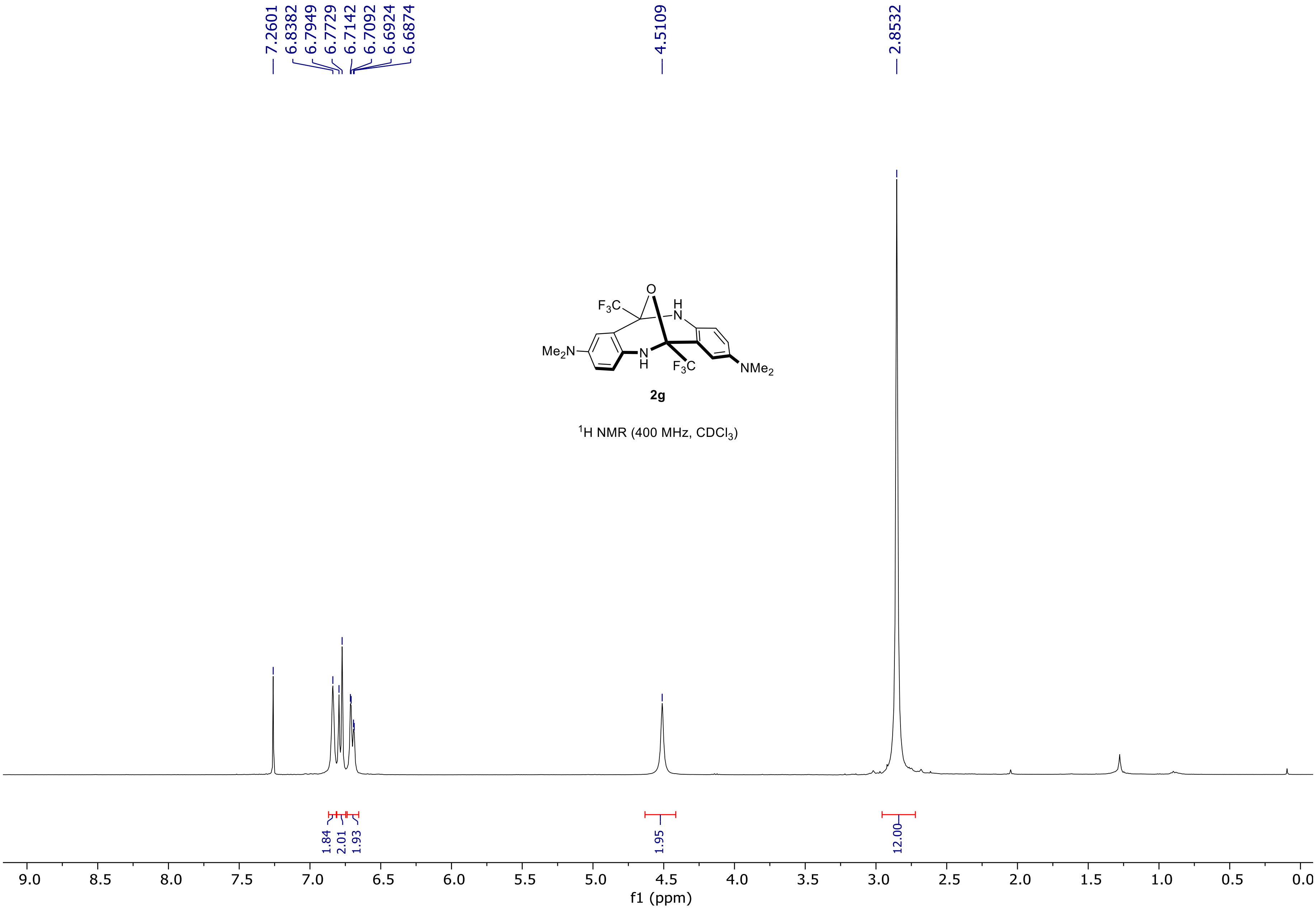


**2g**

$^{1}$H NMR (400 MHz, CDCl$_3$)

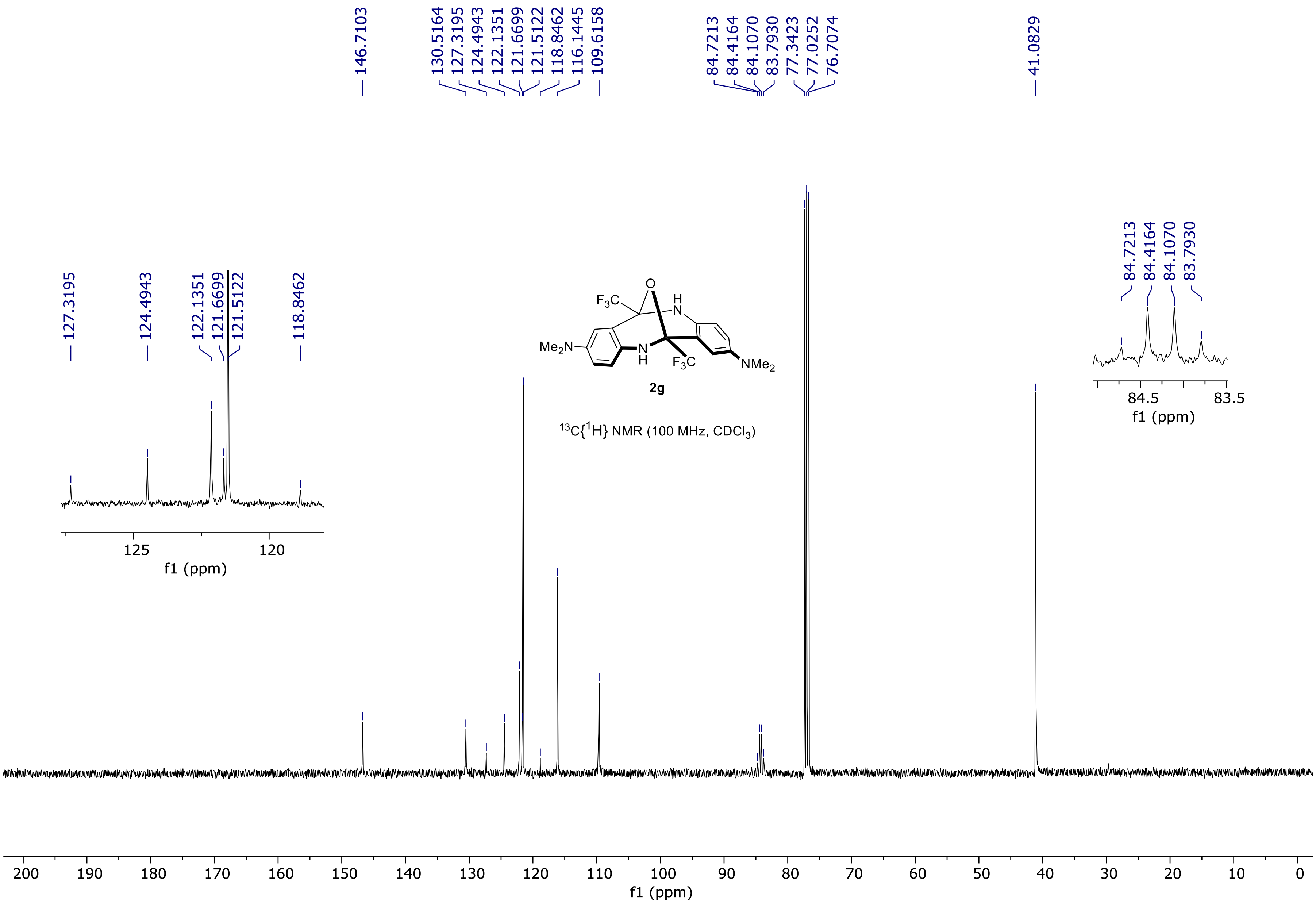

146.7103
130.5164
127.3195
124.4943
122.1351
121.6699
121.5122
118.8462
116.1445
109.6158
84.7213
84.4164
84.1070
83.7930
77.3423
77.0252
76.7074
41.0829
2g
$^{13}C\{^1H\}$ NMR (100 MHz, $CDCl_3$)
f1 (ppm)

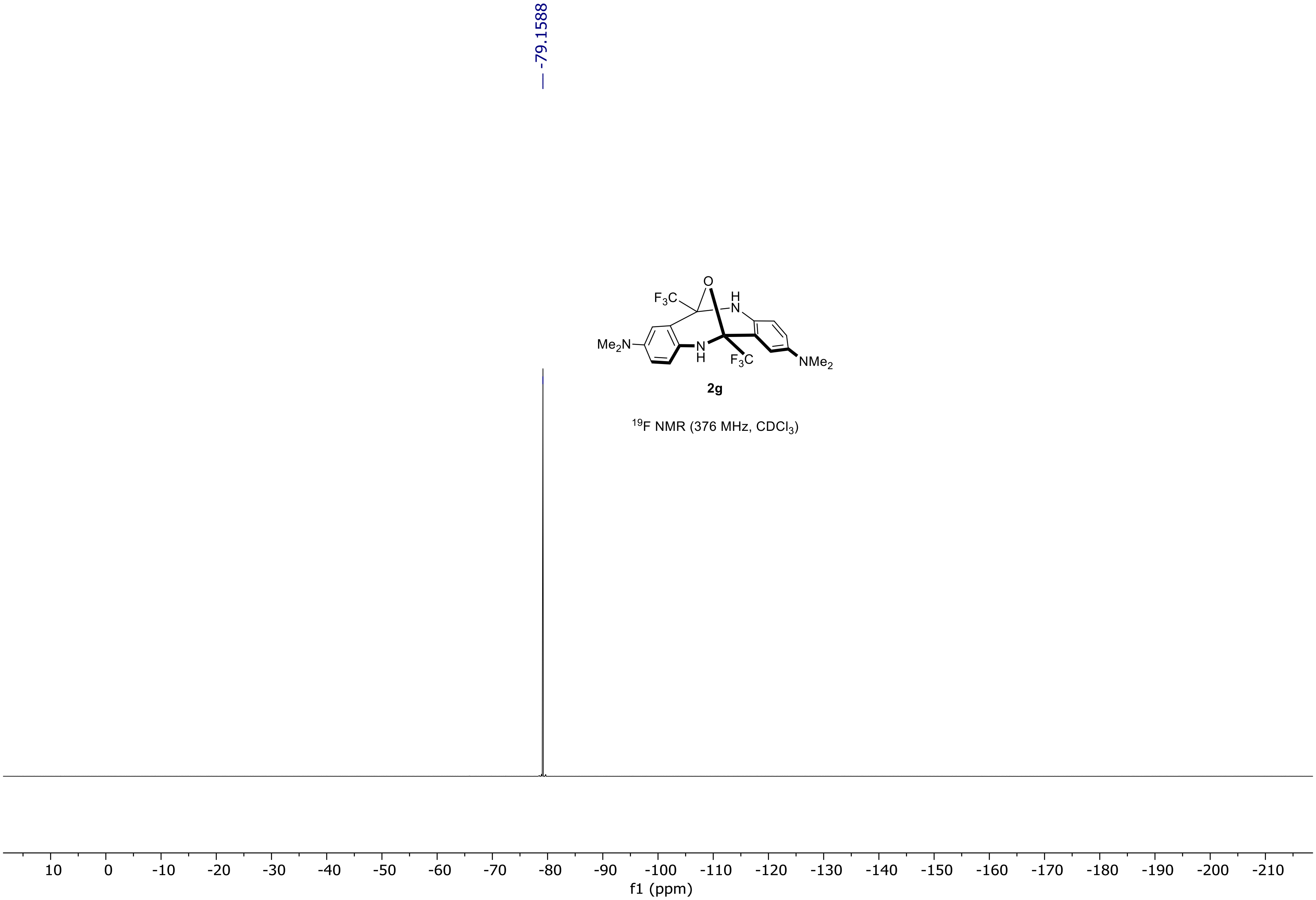
-79.1588
F3C
O
H
N
Me2N
N
H
F3C
NMe2
2g
19F NMR (376 MHz, CDCl3)
10 0 -10 -20 -30 -40 -50 -60 -70 -80 -90 -100 -110 -120 -130 -140 -150 -160 -170 -180 -190 -200 -210
f1 (ppm)

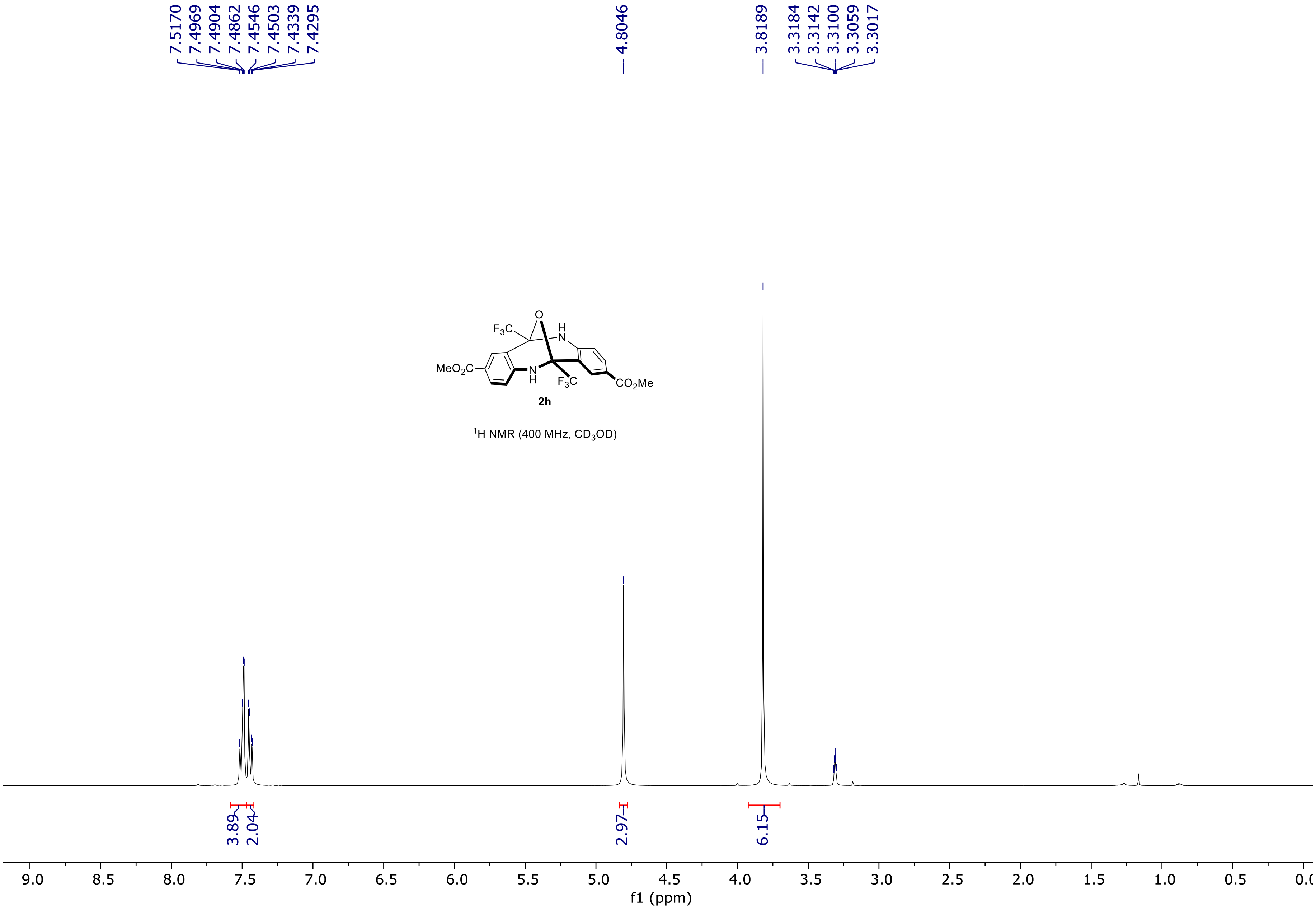
7.5170
7.4969
7.4904
7.4862
7.4546
7.4503
7.4339
7.4295
4.8046
3.8189
3.3184
3.3142
3.3100
3.3059
3.3017
$F_3C$
O
H
N
$MeO_2C$
N
H
$F_3C$
$CO_2Me$
2h
$^1H$ NMR (400 MHz, $CD_3OD$)
3.89
2.04
2.97
6.15
9.0
8.5
8.0
7.5
7.0
6.5
6.0
5.5
5.0
4.5
4.0
3.5
3.0
2.5
2.0
1.5
1.0
0.5
f1 (ppm)

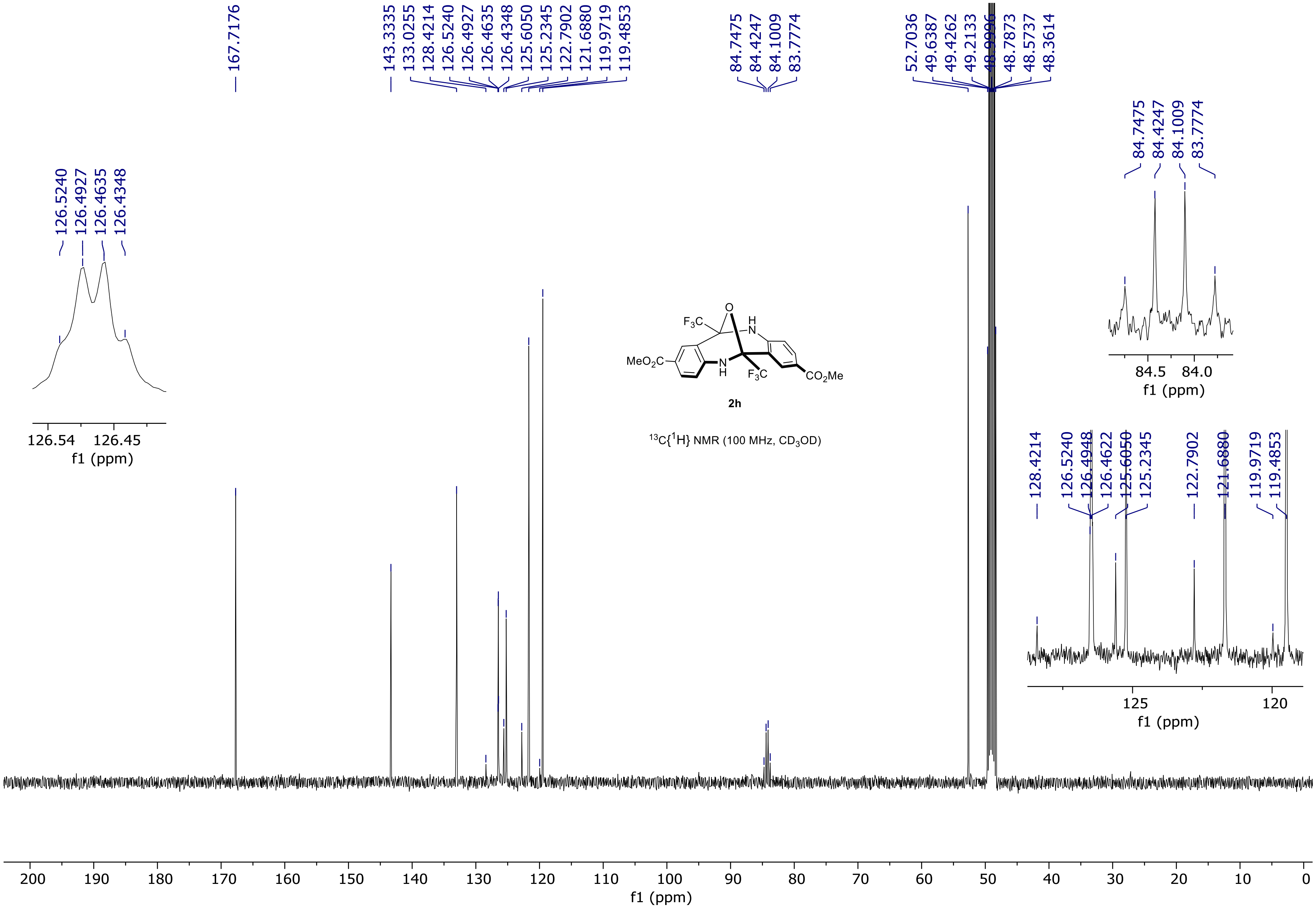
167.7176
143.3335
133.0255
128.4214
126.5240
126.4927
126.4635
126.4348
125.6050
125.2345
122.7902
121.6880
119.9719
119.4853
84.7475
84.4247
84.1009
83.7774
52.7036
49.6387
49.4262
49.2133
48.9996
48.7873
48.5737
48.3614
F3C
O
H
N
MeO2C
N
H
F3C
CO2Me
2h
13C{1H} NMR (100 MHz, CD3OD)
126.54
126.45
84.5
84.0
125
120
126.4948
126.4622
f1 (ppm)
200 190 180 170 160 150 140 130 120 110 100 90 80 70 60 50 40 30 20 10 0

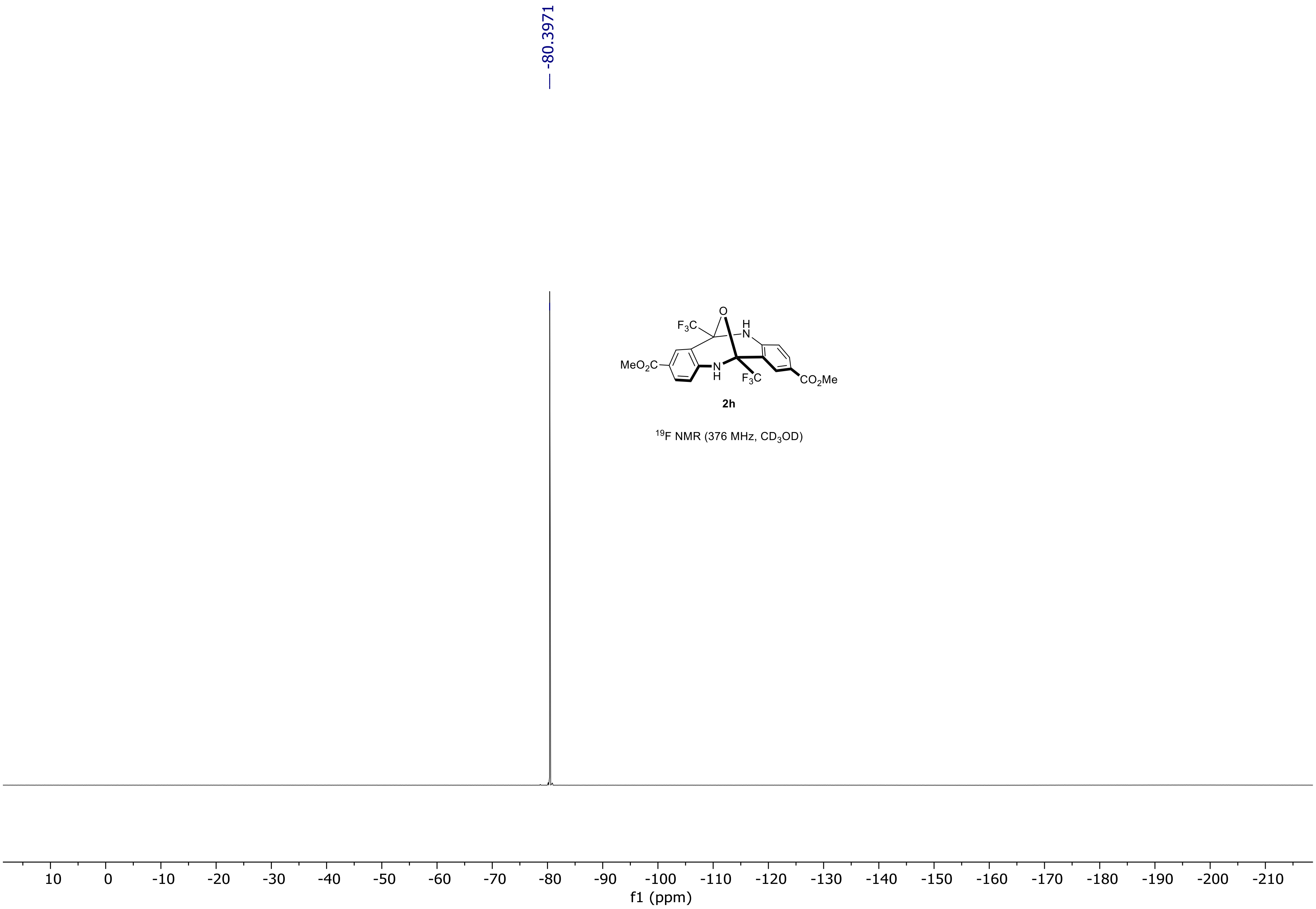
-80.3971
F3C
O
H
N
MeO2C
N
H
F3C
CO2Me
2h
19F NMR (376 MHz, CD3OD)
10 0 -10 -20 -30 -40 -50 -60 -70 -80 -90 -100 -110 -120 -130 -140 -150 -160 -170 -180 -190 -200 -210
f1 (ppm)

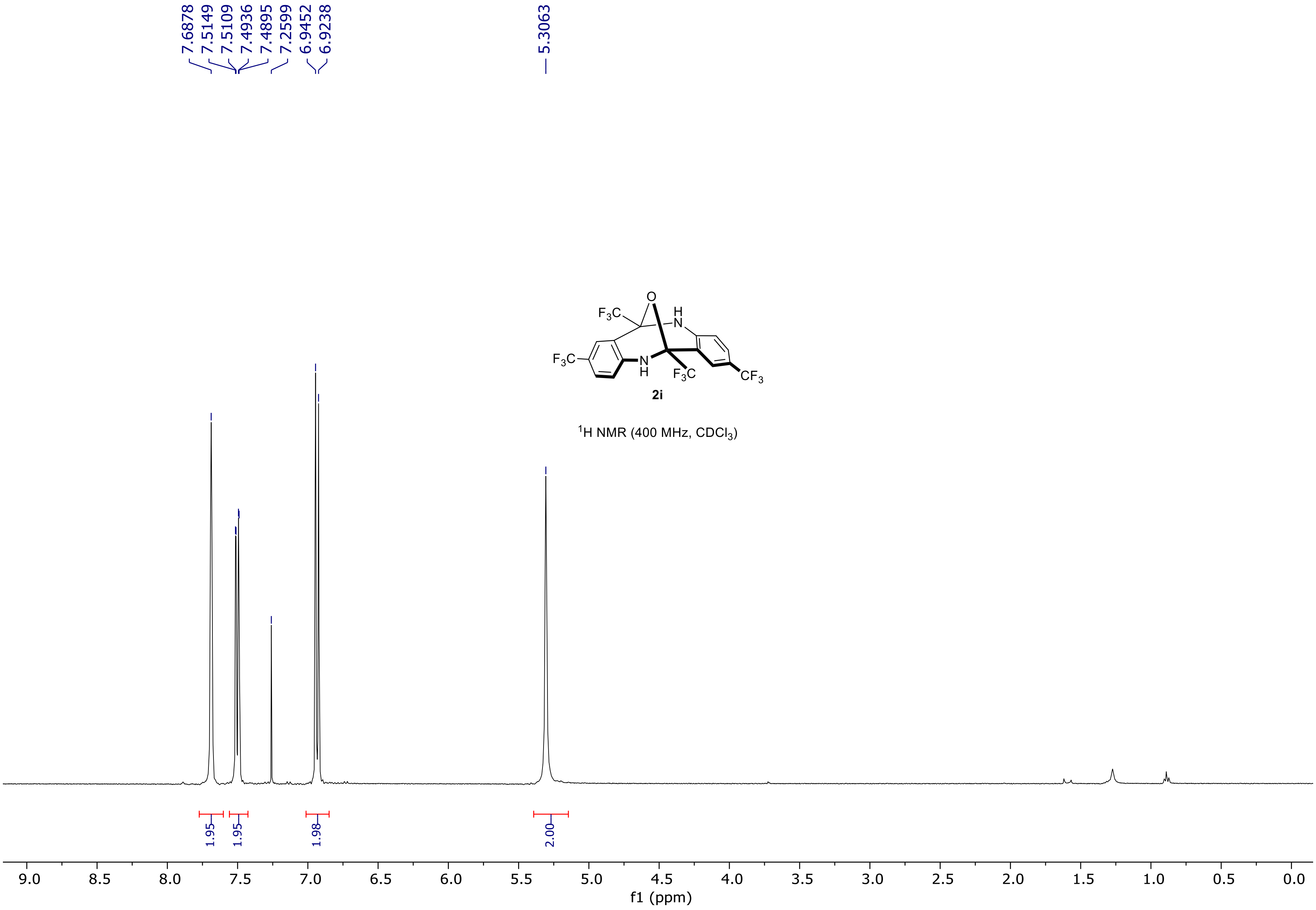


**2i**

$^{1}H$ NMR (400 MHz, $CDCl_3$)

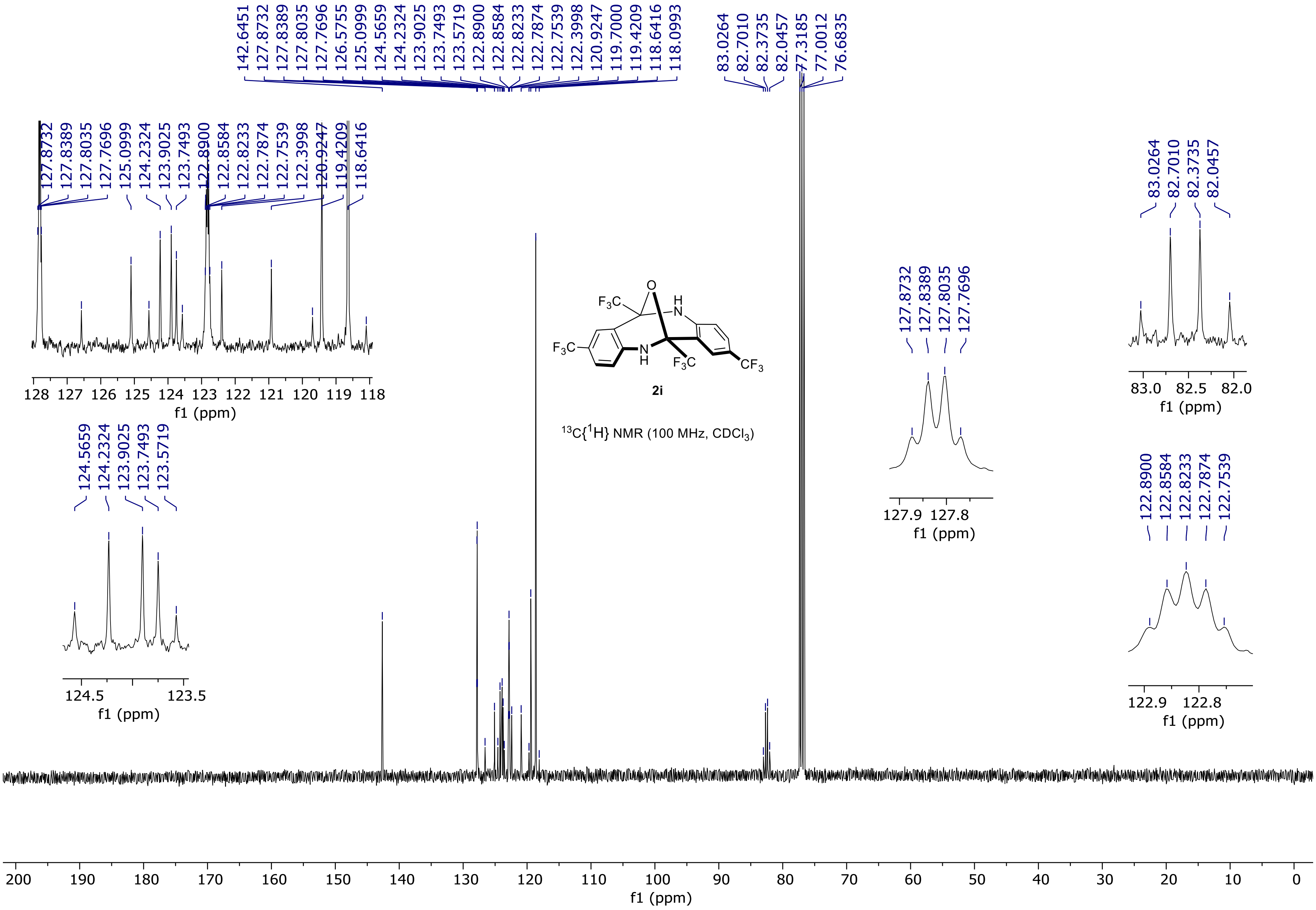
142.6451
127.8732
127.8389
127.8035
127.7696
126.5755
125.0999
124.5659
124.2324
123.9025
123.7493
123.5719
122.8900
122.8584
122.8233
122.7874
122.7539
122.3998
120.9247
119.7000
119.4209
118.6416
118.0993
83.0264
82.7010
82.3735
82.0457
77.3185
77.0012
76.6835
F3C
O
H
N
F3C
N
H
F3C
CF3
2i
13C{1H} NMR (100 MHz, CDCl3)
f1 (ppm)

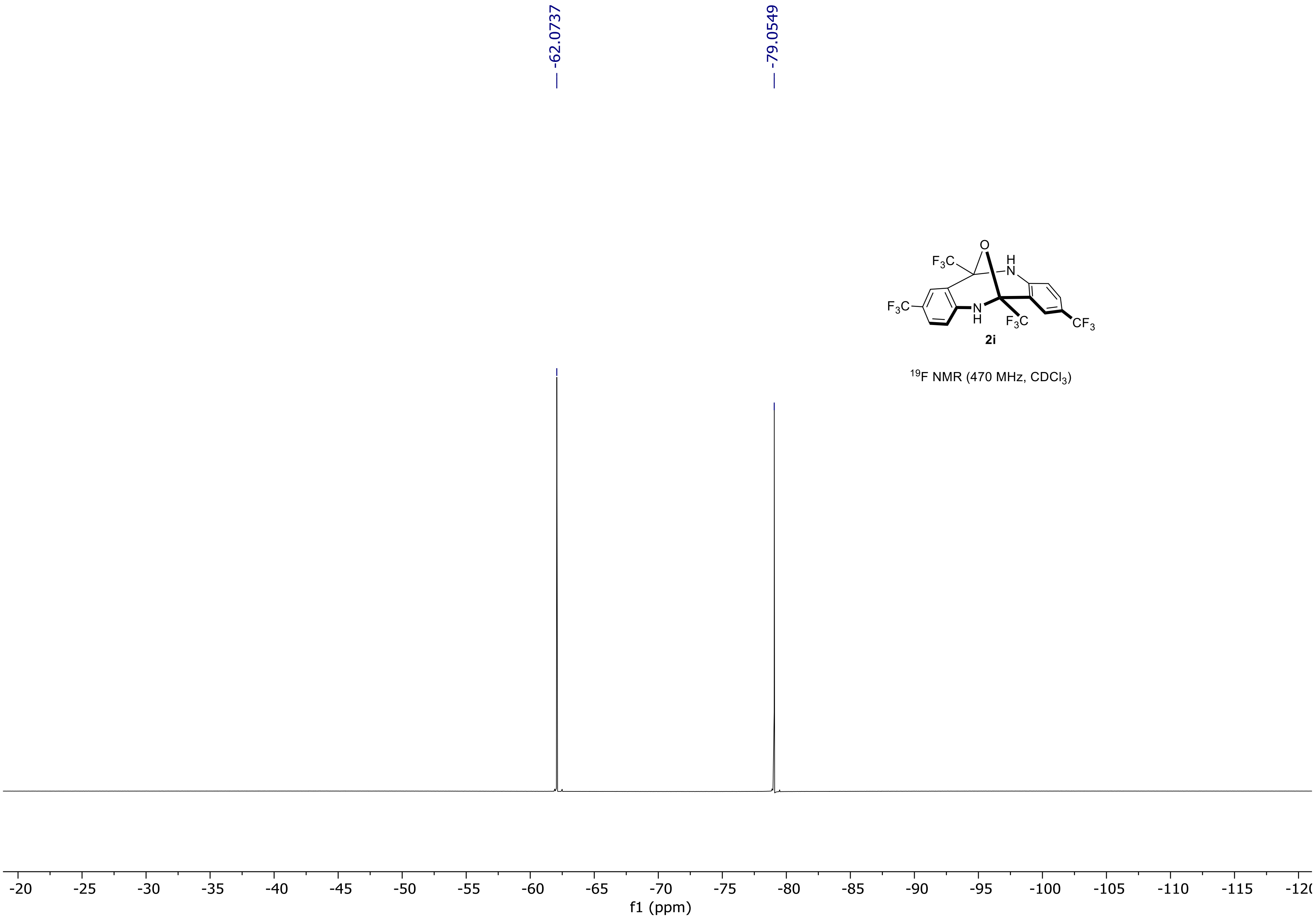
-62.0737
-79.0549
2i
$^{19}$F NMR (470 MHz, CDCl$_3$)
-20 -25 -30 -35 -40 -45 -50 -55 -60 -65 -70 -75 -80 -85 -90 -95 -100 -105 -110 -115 -120
f1 (ppm)

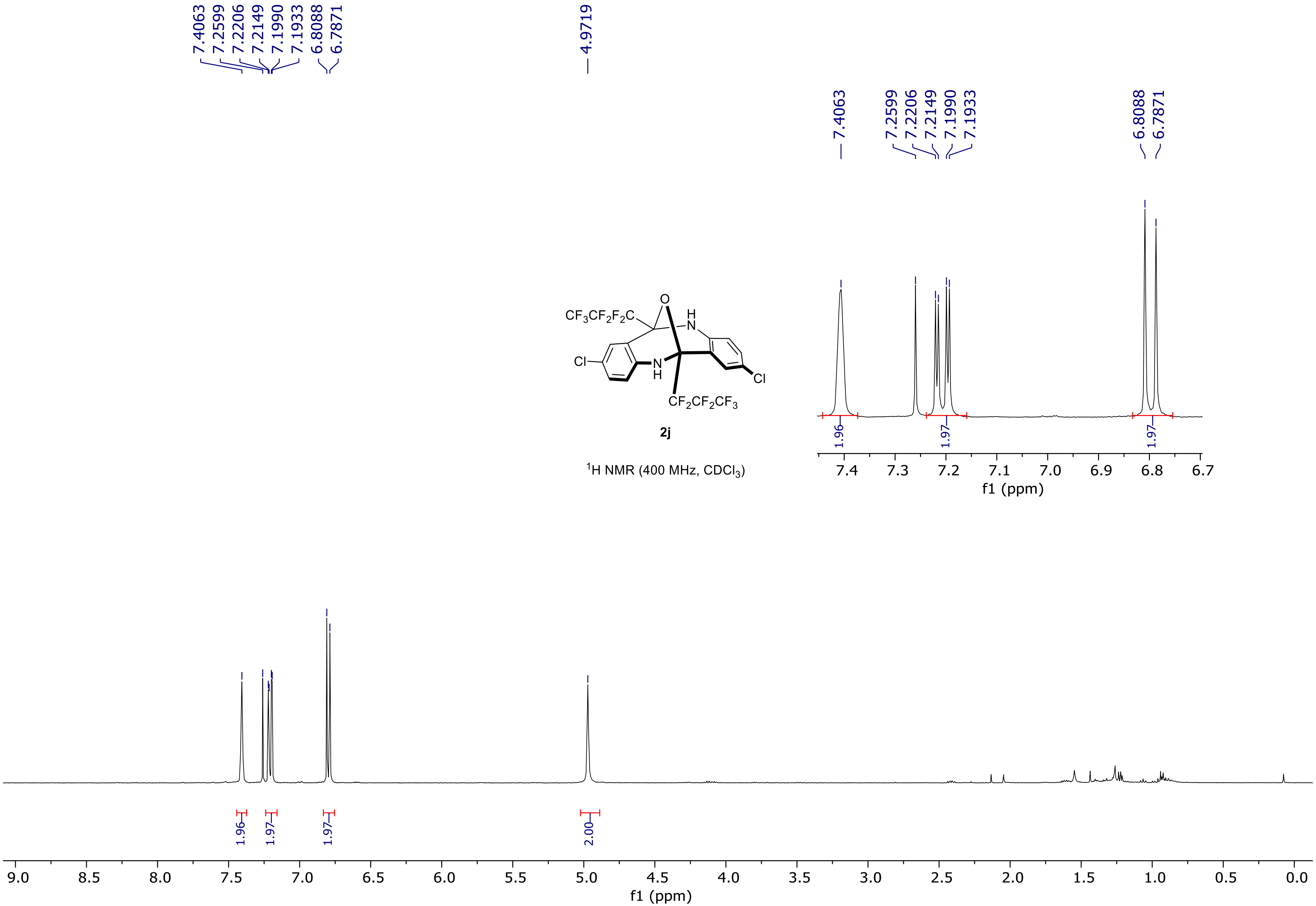


**2j**

$^{1}$H NMR (400 MHz, $CDCl_3$)

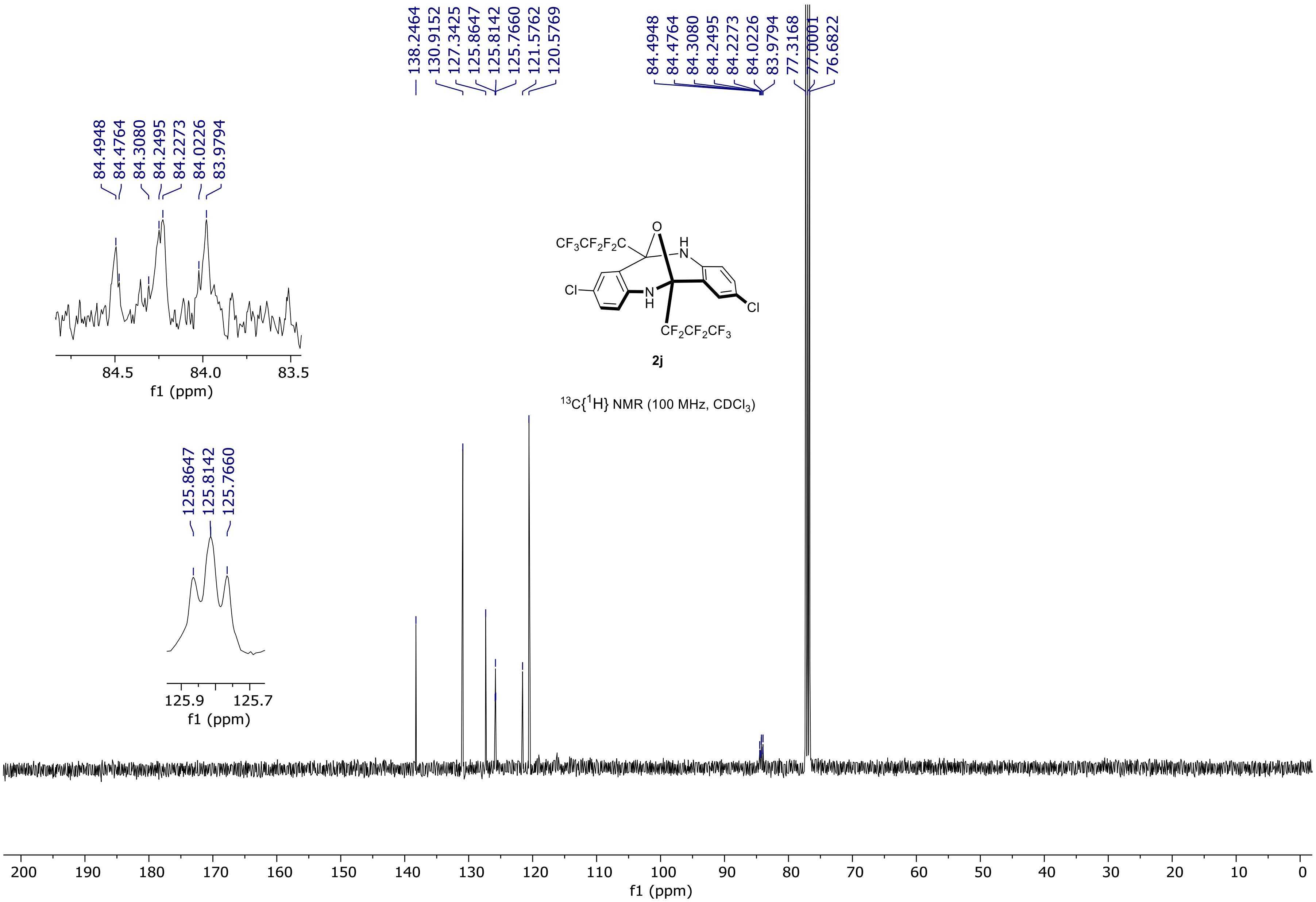
138.2464
130.9152
127.3425
125.8647
125.8142
125.7660
121.5762
120.5769
84.4948
84.4764
84.3080
84.2495
84.2273
84.0226
83.9794
77.3168
77.0001
76.6822
$CF_3CF_2F_2C$
O
H
N
Cl
N
H
Cl
$CF_2CF_2CF_3$
2j
$^{13}C\{^1H\}$ NMR (100 MHz, $CDCl_3$)
f1 (ppm)

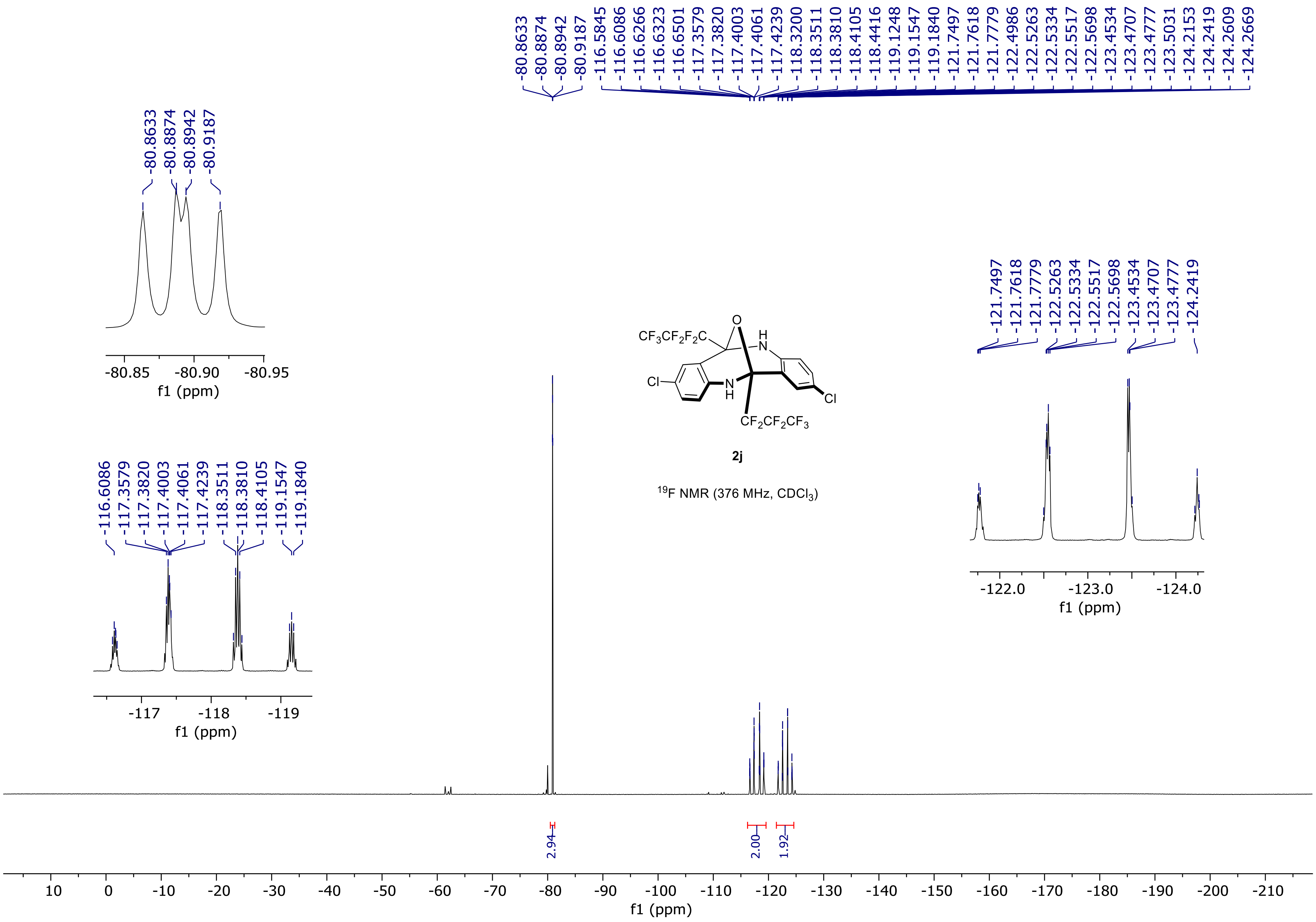
2j
$^{19}$F NMR (376 MHz, $CDCl_3$)
f1 (ppm)
2.94
2.00
1.92

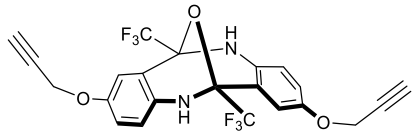


**2k**

$^{1}$H NMR (400 MHz, $CDCl_3$)

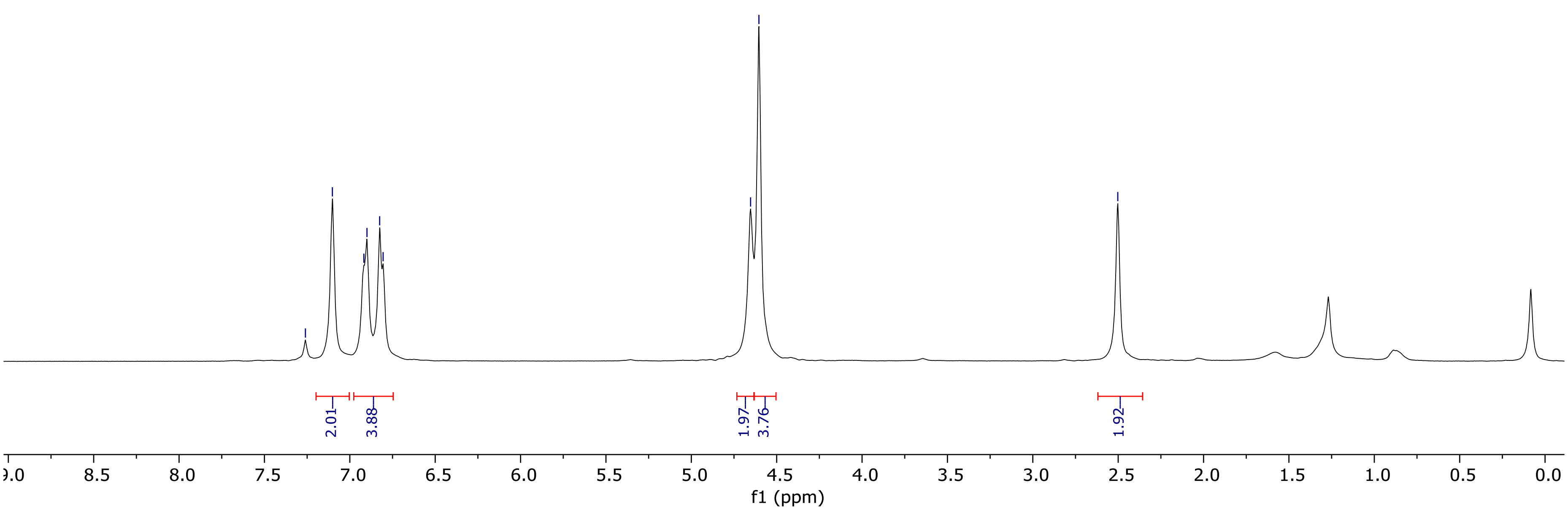

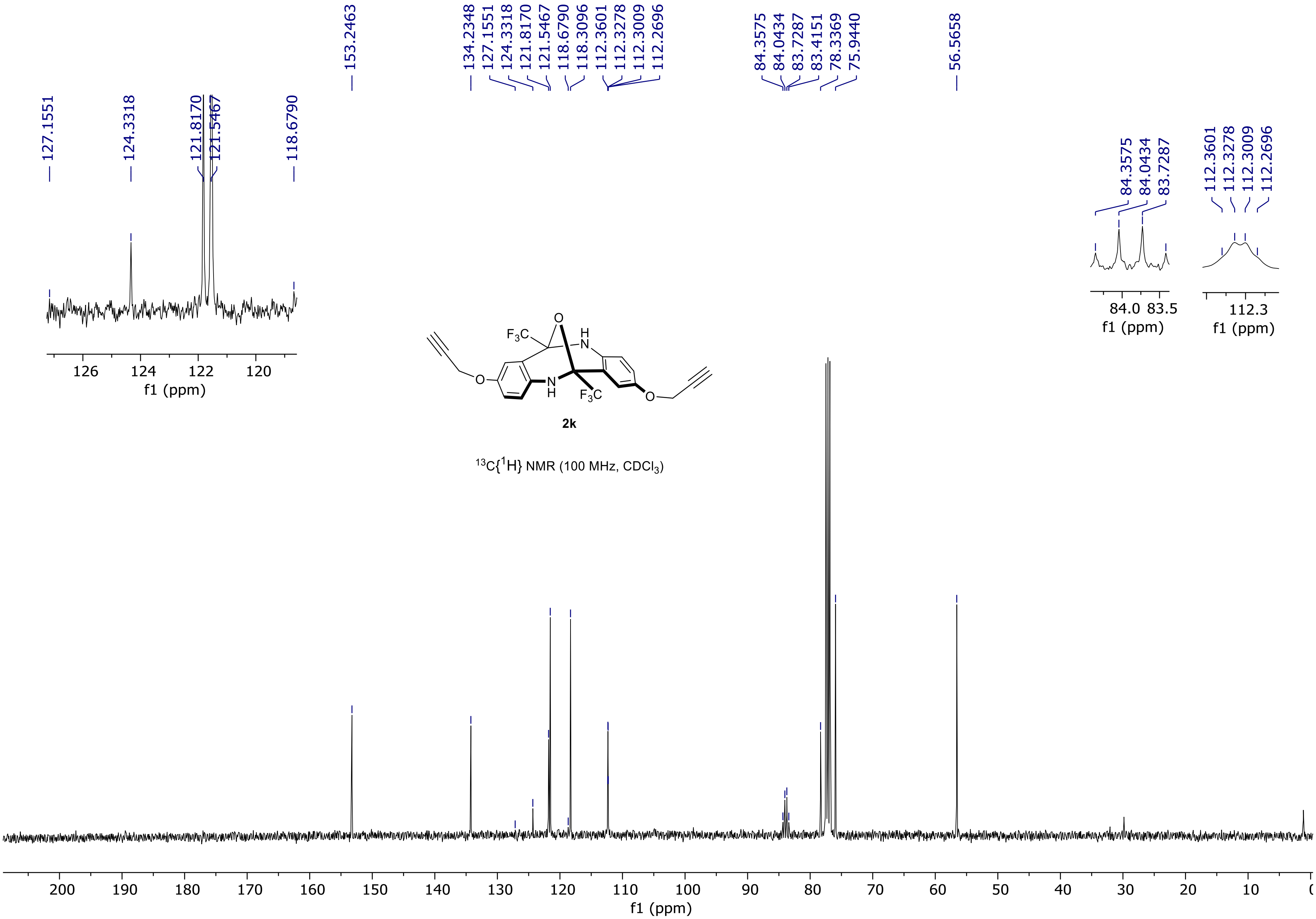


**2k**

$^{13}C\{^{1}H\}$ NMR (100 MHz, $CDCl_3$)

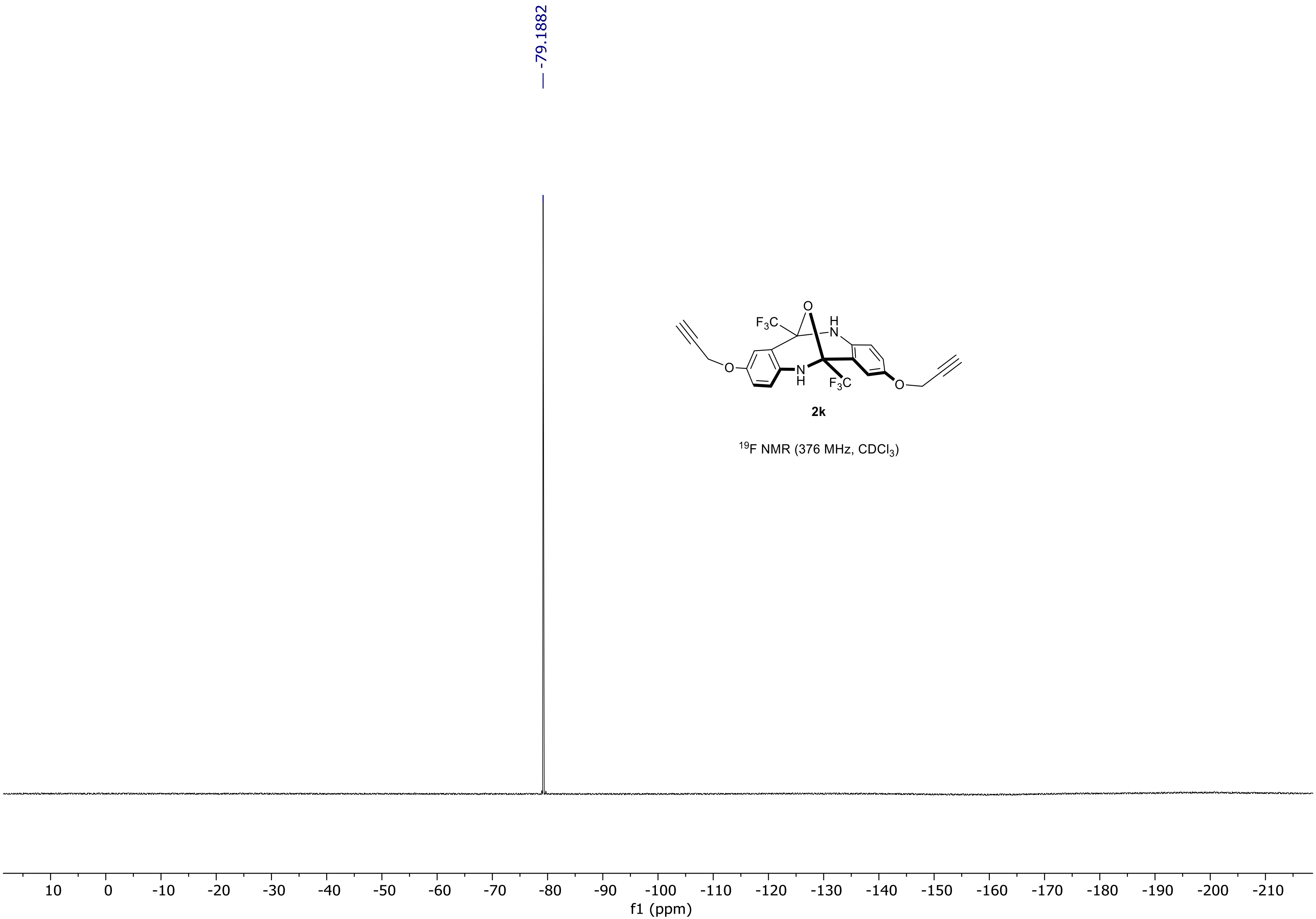

-79.1882
F3C
O
H
N
O
N
H
F3C
O
2k
19F NMR (376 MHz, CDCl3)
10
0
-10
-20
-30
-40
-50
-60
-70
-80
-90
-100
-110
-120
-130
-140
-150
-160
-170
-180
-190
-200
-210
f1 (ppm)

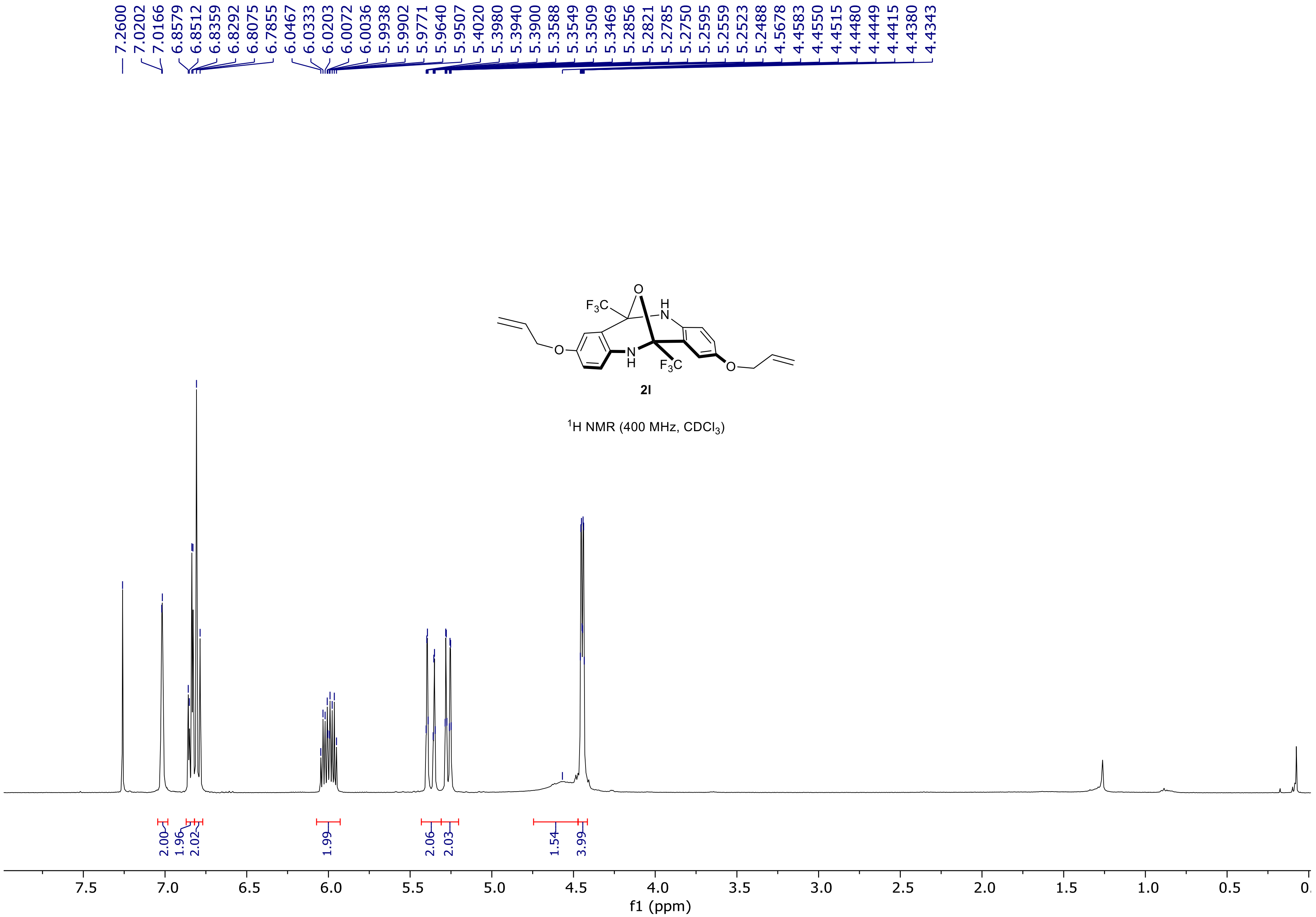
7.2600
7.0202
7.0166
6.8579
6.8512
6.8359
6.8292
6.8075
6.7855
6.0467
6.0333
6.0203
6.0072
6.0036
5.9938
5.9902
5.9771
5.9640
5.9507
5.4020
5.3980
5.3940
5.3900
5.3588
5.3549
5.3509
5.3469
5.2856
5.2821
5.2785
5.2750
5.2595
5.2559
5.2523
5.2488
4.5678
4.4583
4.4550
4.4515
4.4480
4.4449
4.4415
4.4380
4.4343
F3C
O
H
N
O
N
H
F3C
O
2l
1H NMR (400 MHz, CDCl3)
2.00
1.96
2.02
1.99
2.06
2.03
1.54
3.99
7.5
7.0
6.5
6.0
5.5
5.0
4.5
4.0
3.5
3.0
2.5
2.0
1.5
1.0
0.5
f1 (ppm)

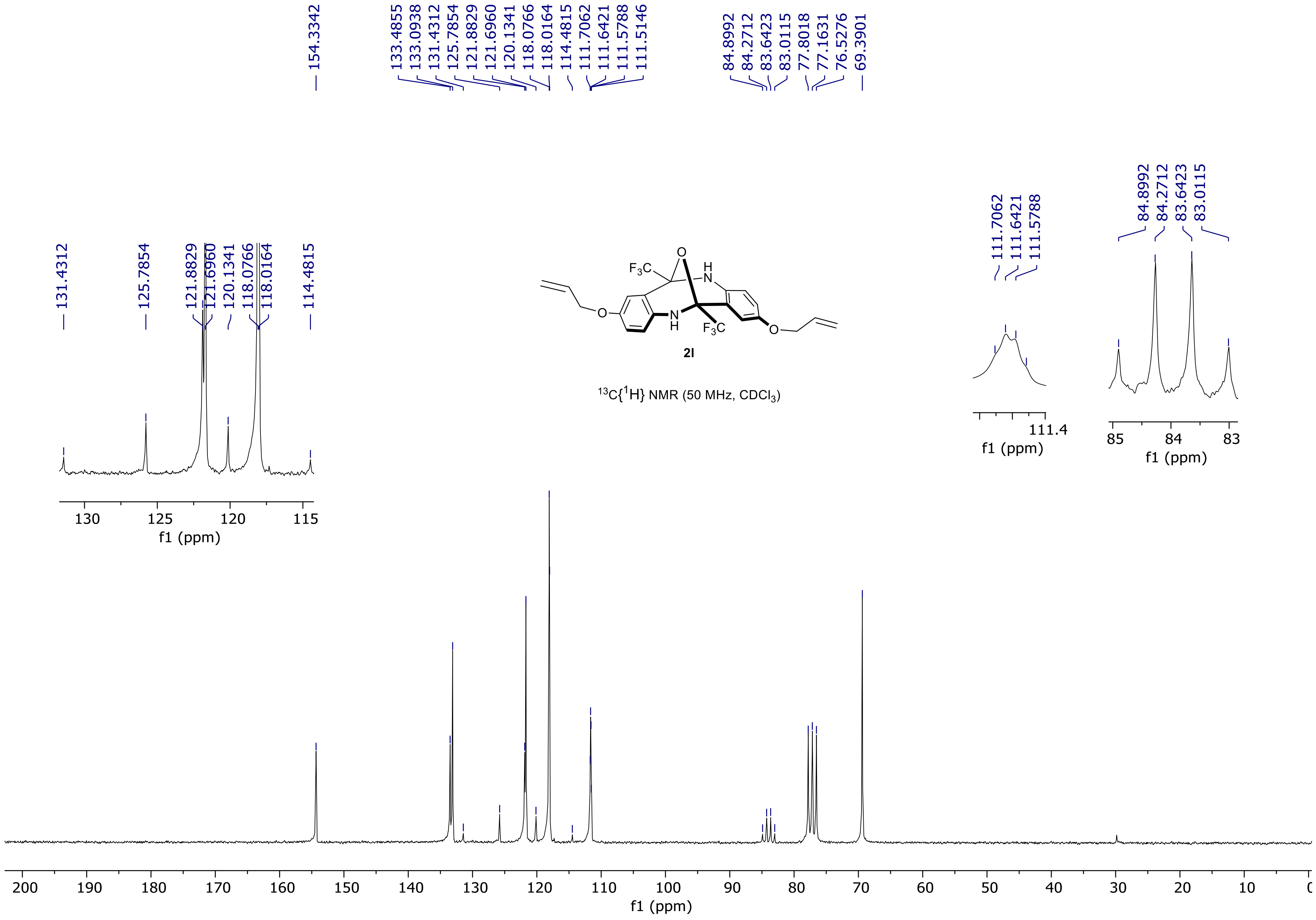
154.3342
133.4855
133.0938
131.4312
125.7854
121.8829
121.6960
120.1341
118.0766
118.0164
114.4815
111.7062
111.6421
111.5788
111.5146
84.8992
84.2712
83.6423
83.0115
77.8018
77.1631
76.5276
69.3901
F3C
O
H
N
N
H
CF3
O
O
2l
13C{1H} NMR (50 MHz, CDCl3)
f1 (ppm)

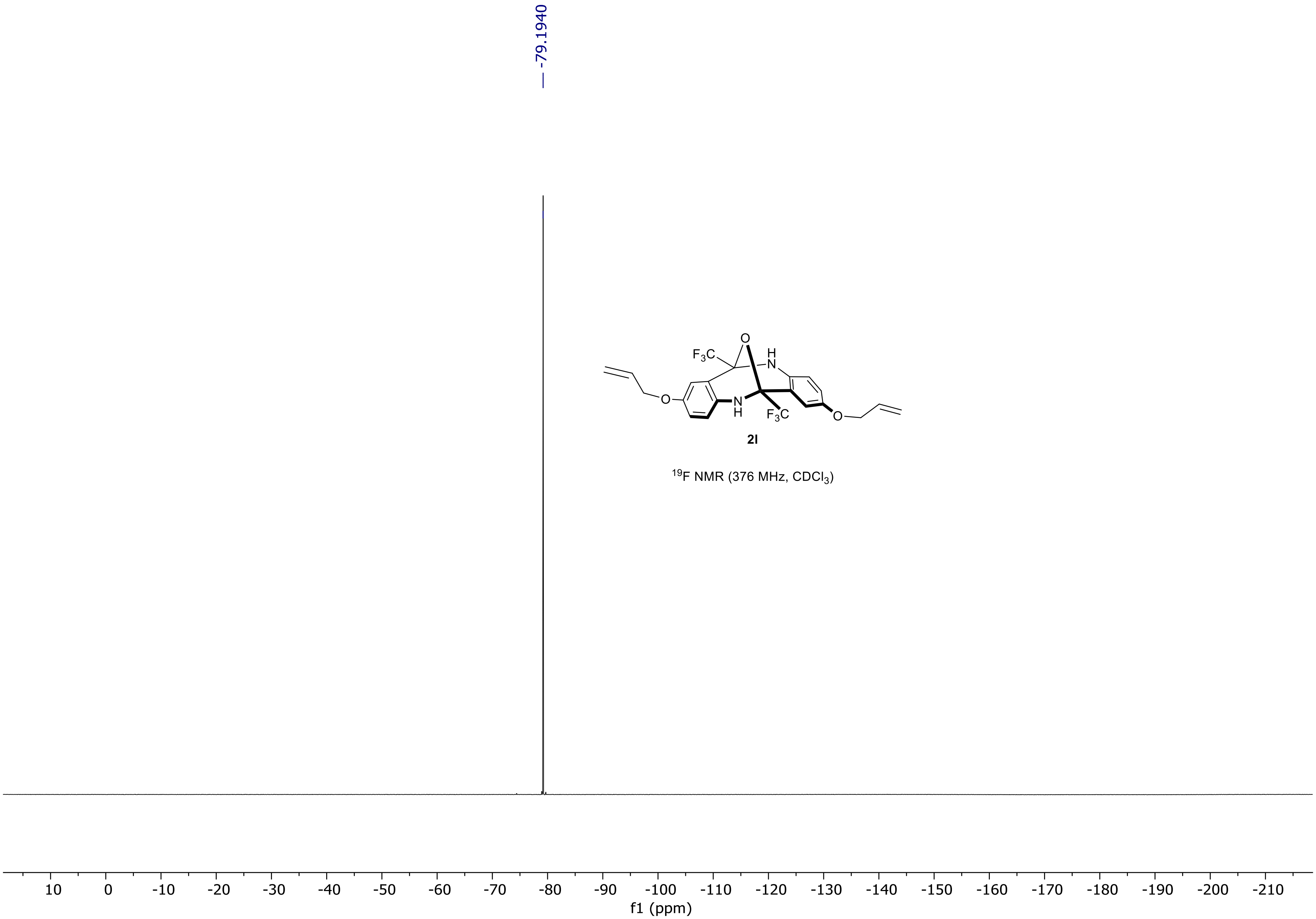

-79.1940
F3C
O
H
N
O
N
H
F3C
O
2l
19F NMR (376 MHz, CDCl3)
10 0 -10 -20 -30 -40 -50 -60 -70 -80 -90 -100 -110 -120 -130 -140 -150 -160 -170 -180 -190 -200 -210
f1 (ppm)

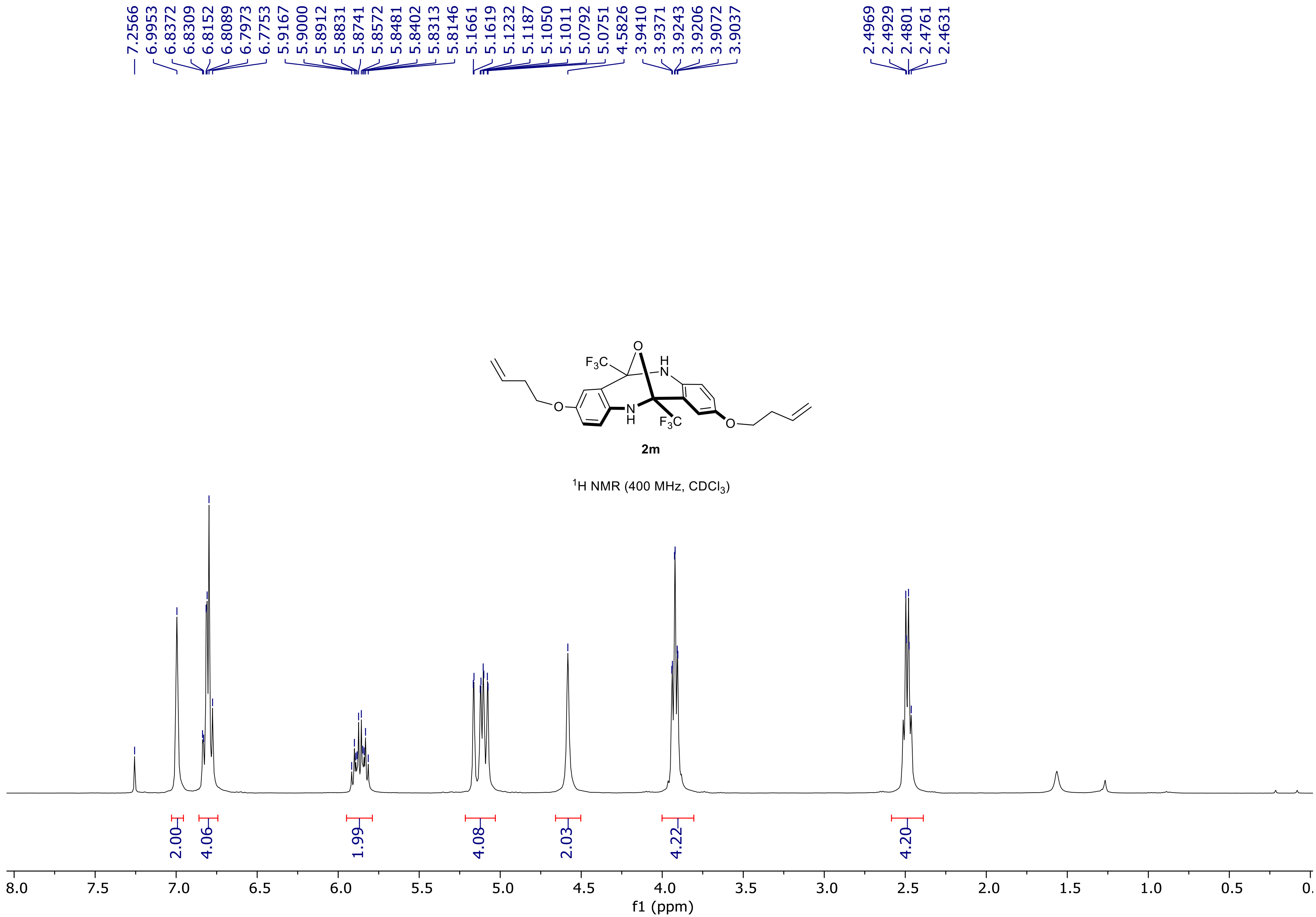
7.2566
6.9953
6.8372
6.8309
6.8152
6.8089
6.7973
6.7753
5.9167
5.9000
5.8912
5.8831
5.8741
5.8572
5.8481
5.8402
5.8313
5.8146
5.1661
5.1619
5.1232
5.1187
5.1050
5.1011
5.0792
5.0751
4.5826
3.9410
3.9371
3.9243
3.9206
3.9072
3.9037
2.4969
2.4929
2.4801
2.4761
2.4631
F3C
O
H
N
N
H
F3C
O
O
2m
1H NMR (400 MHz, CDCl3)
2.00
4.06
1.99
4.08
2.03
4.22
4.20
8.0
7.5
7.0
6.5
6.0
5.5
5.0
4.5
4.0
3.5
3.0
2.5
2.0
1.5
1.0
0.5
f1 (ppm)

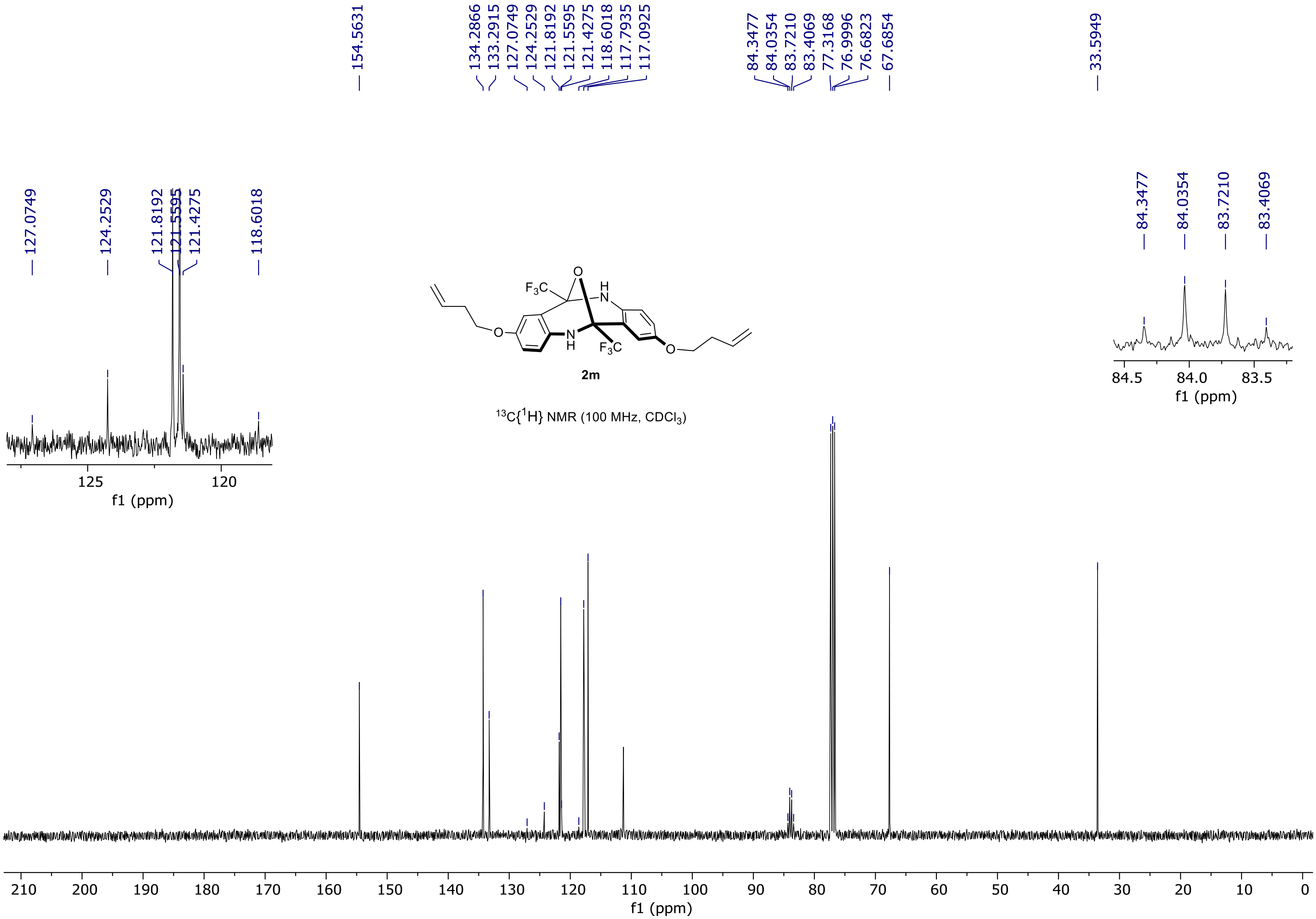
154.5631
134.2866
133.2915
127.0749
124.2529
121.8192
121.5595
121.4275
118.6018
117.7935
117.0925
84.3477
84.0354
83.7210
83.4069
77.3168
76.9996
76.6823
67.6854
33.5949
$F_3C$
O
H
N
N
H
$F_3C$
O
O
2m
$^{13}C\{^1H\}$ NMR (100 MHz, $CDCl_3$)
f1 (ppm)

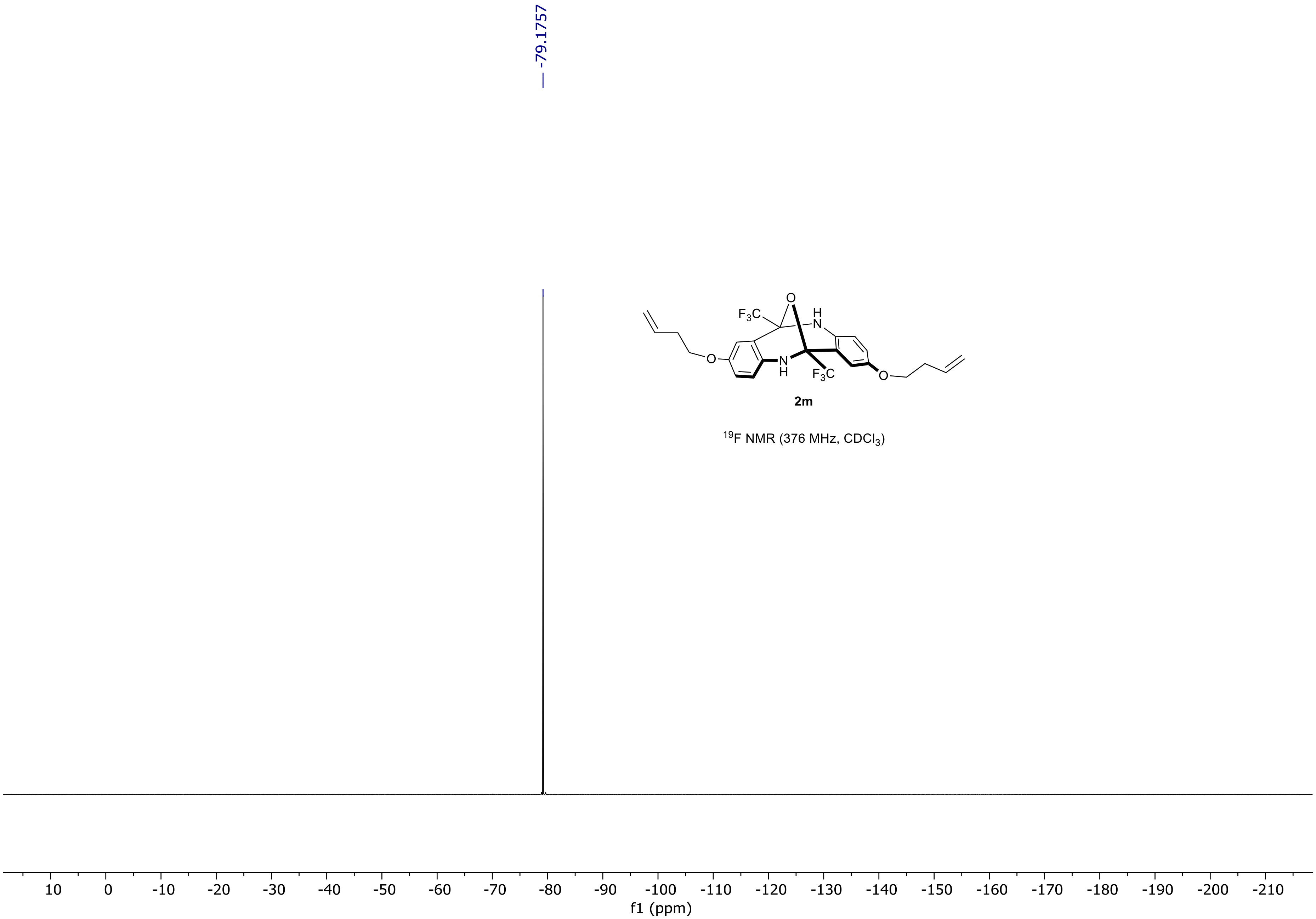
-79.1757
F3C
O
H
N
O
N
H
F3C
O
2m
19F NMR (376 MHz, CDCl3)
10 0 -10 -20 -30 -40 -50 -60 -70 -80 -90 -100 -110 -120 -130 -140 -150 -160 -170 -180 -190 -200 -210
f1 (ppm)

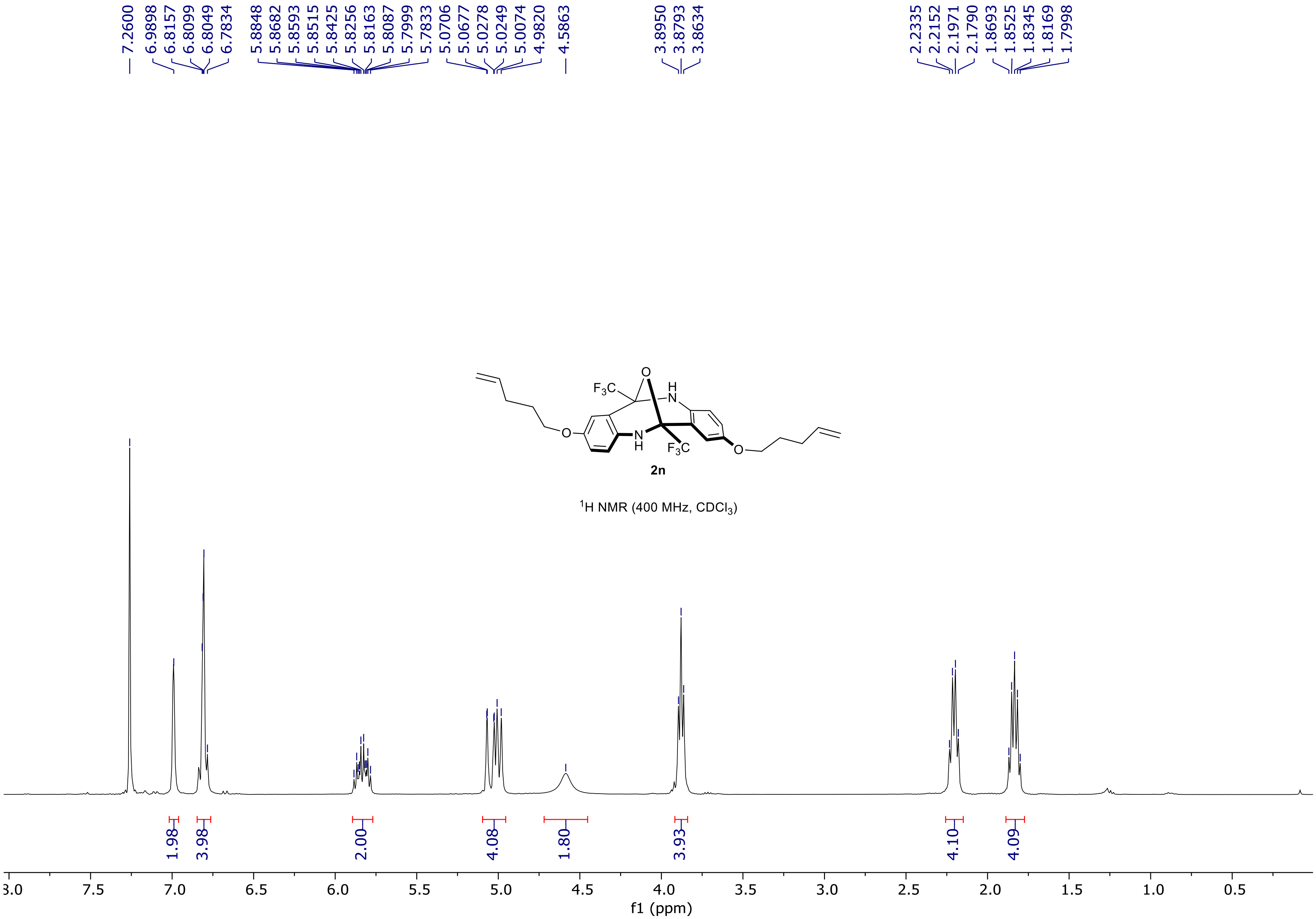
7.2600 6.9898 6.8157 6.8099 6.8049 6.7834 5.8848 5.8682 5.8593 5.8515 5.8425 5.8256 5.8163 5.8087 5.7999 5.7833 5.0706 5.0677 5.0278 5.0249 5.0074 4.9820 4.5863 3.8950 3.8793 3.8634 2.2335 2.2152 2.1971 2.1790 1.8693 1.8525 1.8345 1.8169 1.7998
F3C
O
H
N
O
N
H
F3C
O
2n
1H NMR (400 MHz, CDCl3)
1.98 3.98 2.00 4.08 1.80 3.93 4.10 4.09
f1 (ppm)

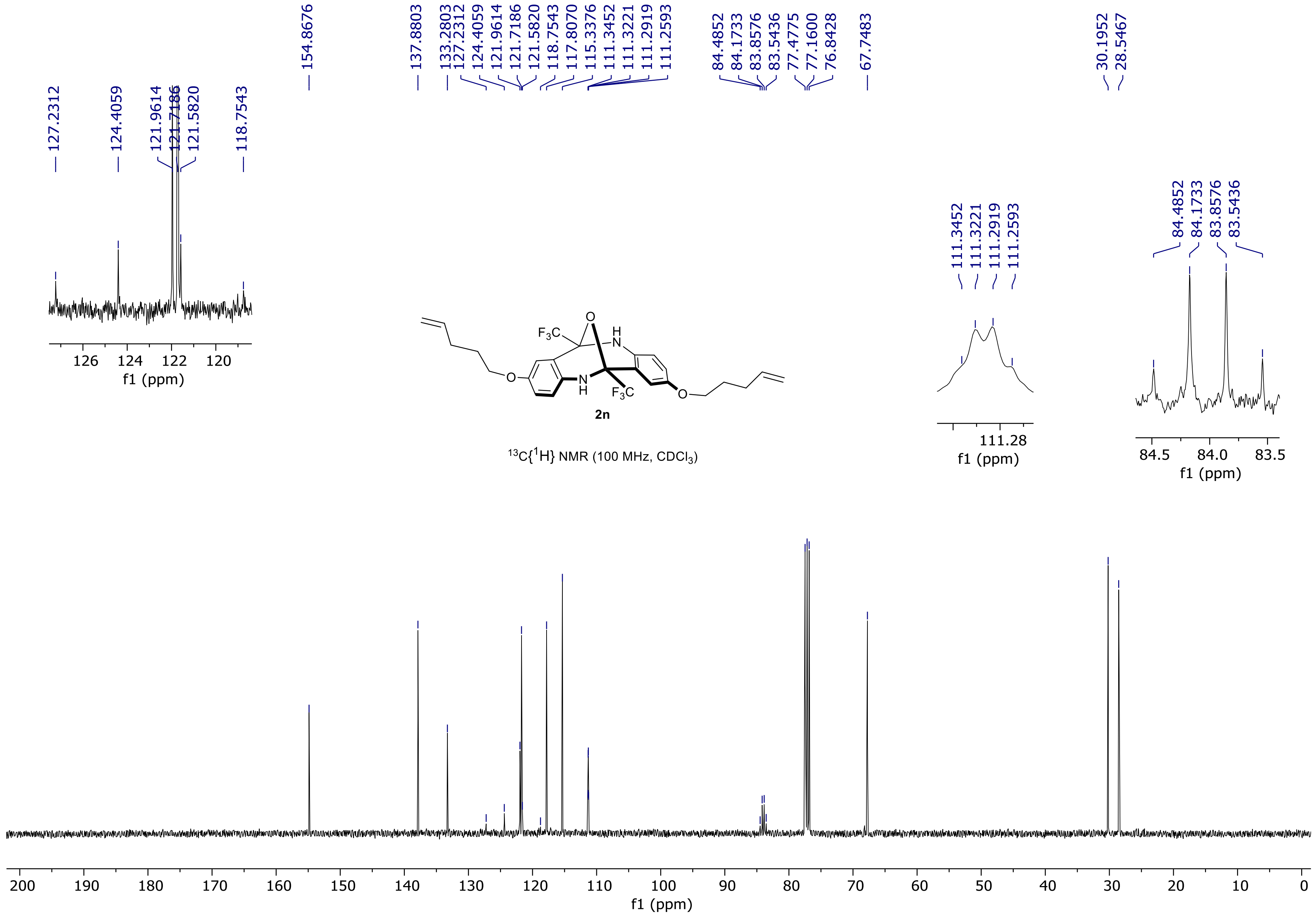

154.8676
137.8803
133.2803
127.2312
124.4059
121.9614
121.7186
121.5820
118.7543
117.8070
115.3376
111.3452
111.3221
111.2919
111.2593
84.4852
84.1733
83.8576
83.5436
77.4775
77.1600
76.8428
67.7483
30.1952
28.5467
2n
$^{13}C\{^{1}H\}$ NMR (100 MHz, $CDCl_3$)
f1 (ppm)

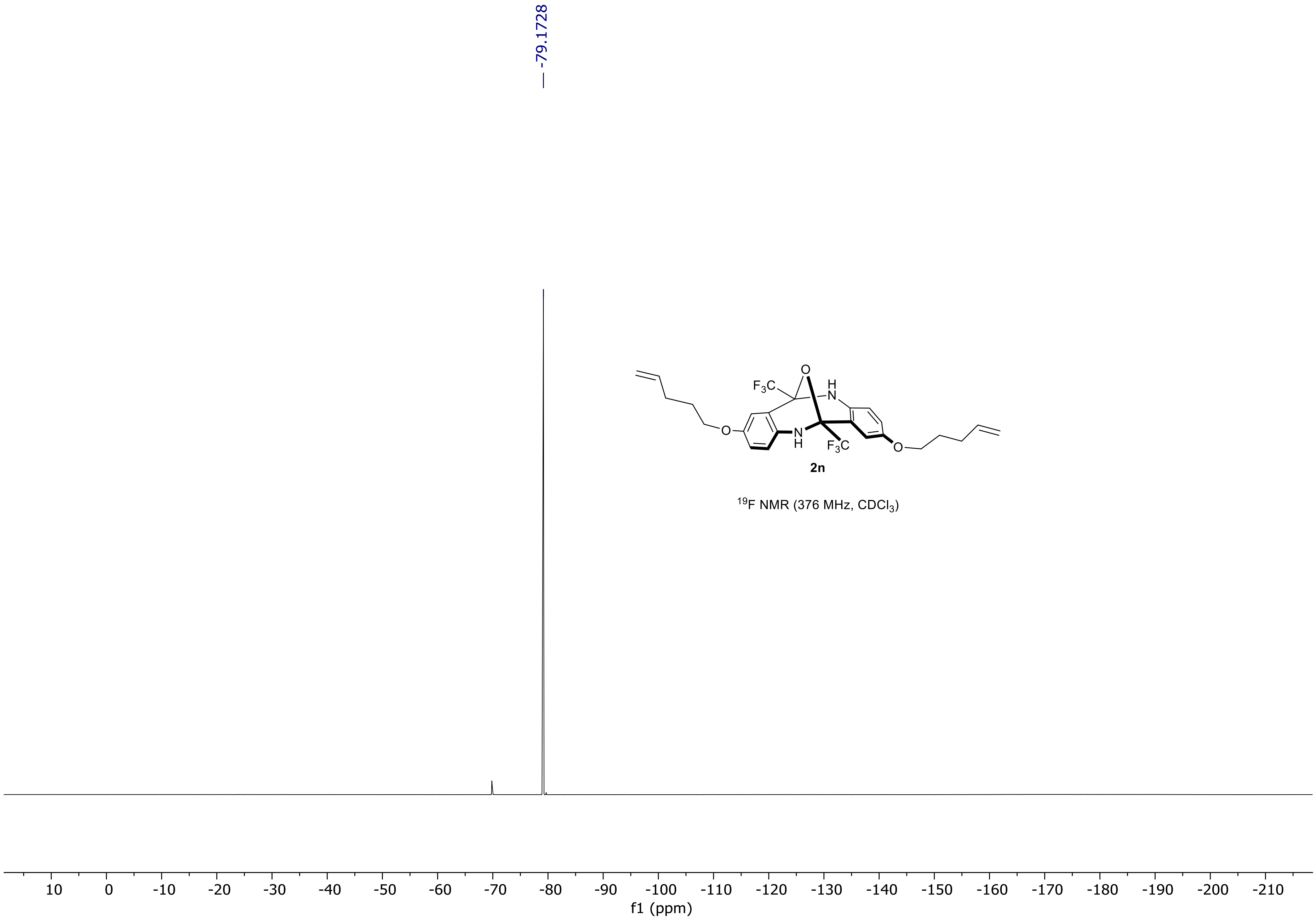
-79.1728
F$_3$C
O
H
N
N
H
F$_3$C
O
O
2n
$^{19}$F NMR (376 MHz, CDCl$_3$)
10 0 -10 -20 -30 -40 -50 -60 -70 -80 -90 -100 -110 -120 -130 -140 -150 -160 -170 -180 -190 -200 -210
f1 (ppm)

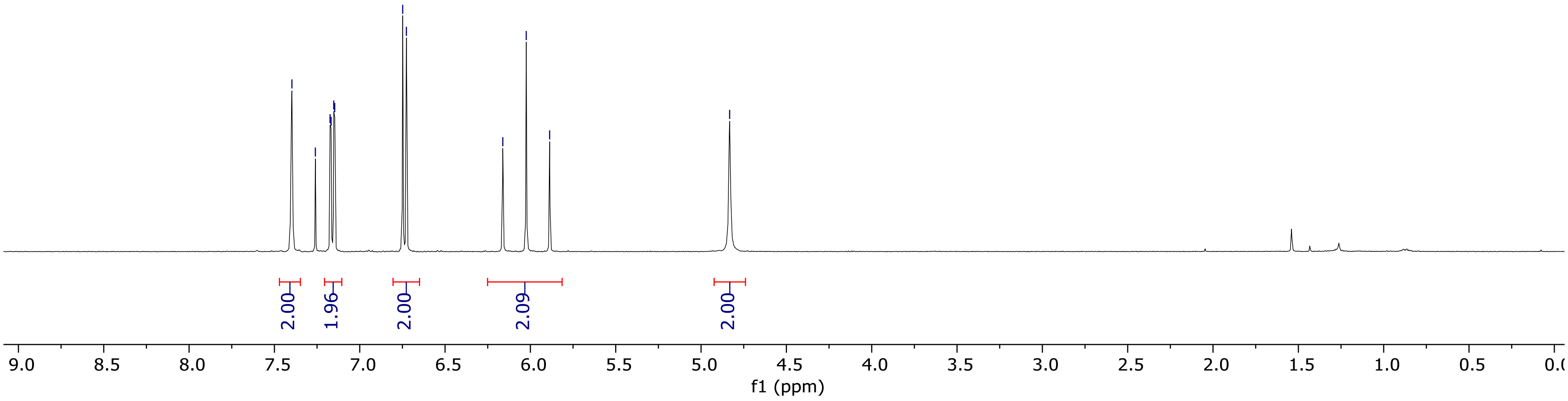


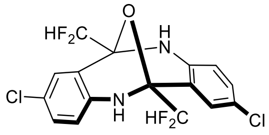


**2o**

$^{1}$H NMR (400 MHz, $CDCl_3$)

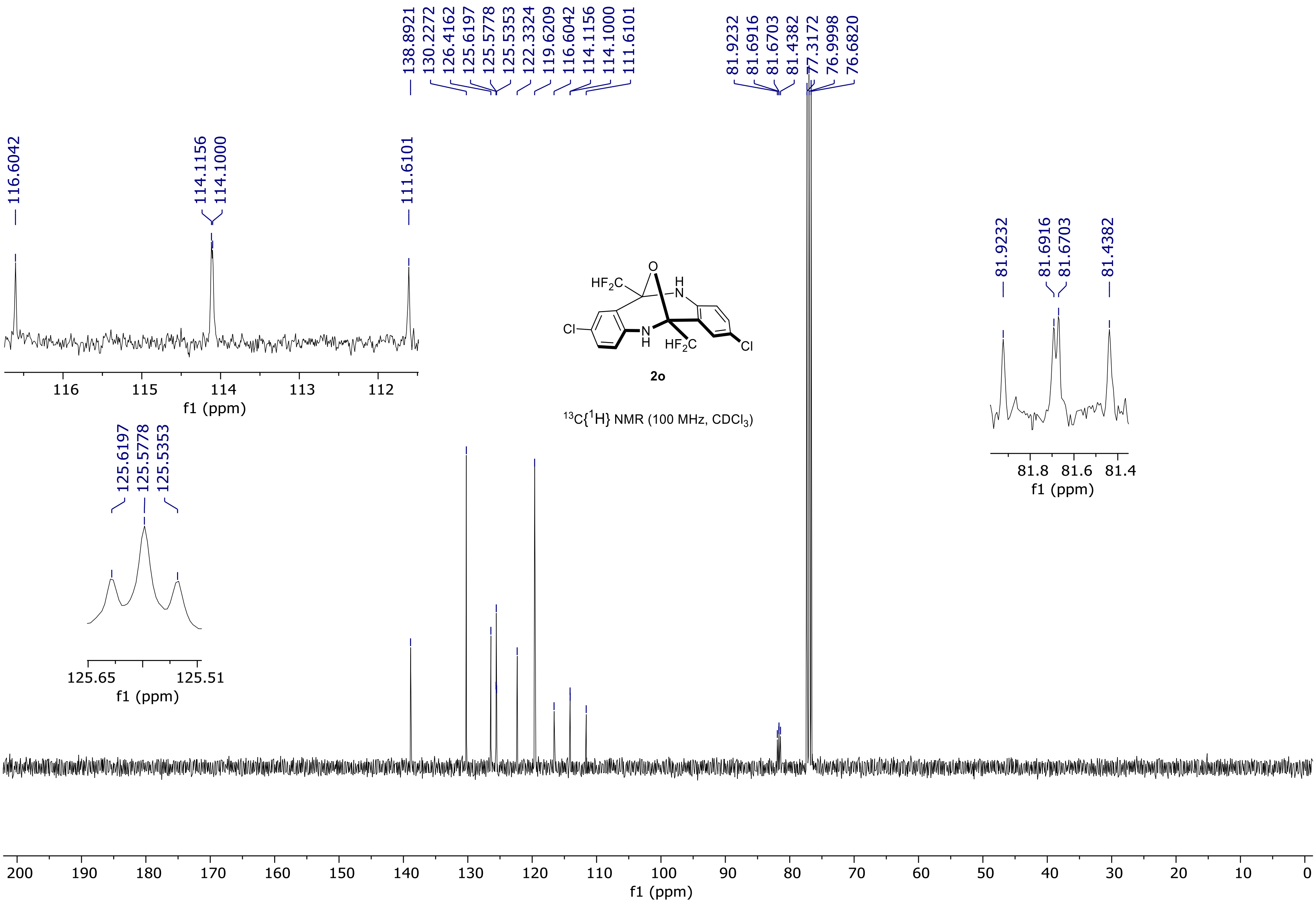
138.8921
130.2272
126.4162
125.6197
125.5778
125.5353
122.3324
119.6209
116.6042
114.1156
114.1000
111.6101
81.9232
81.6916
81.6703
81.4382
77.3172
76.9998
76.6820
HF2C
O
H
N
Cl
N
H
HF2C
Cl
2o
13C{1H} NMR (100 MHz, CDCl3)
f1 (ppm)

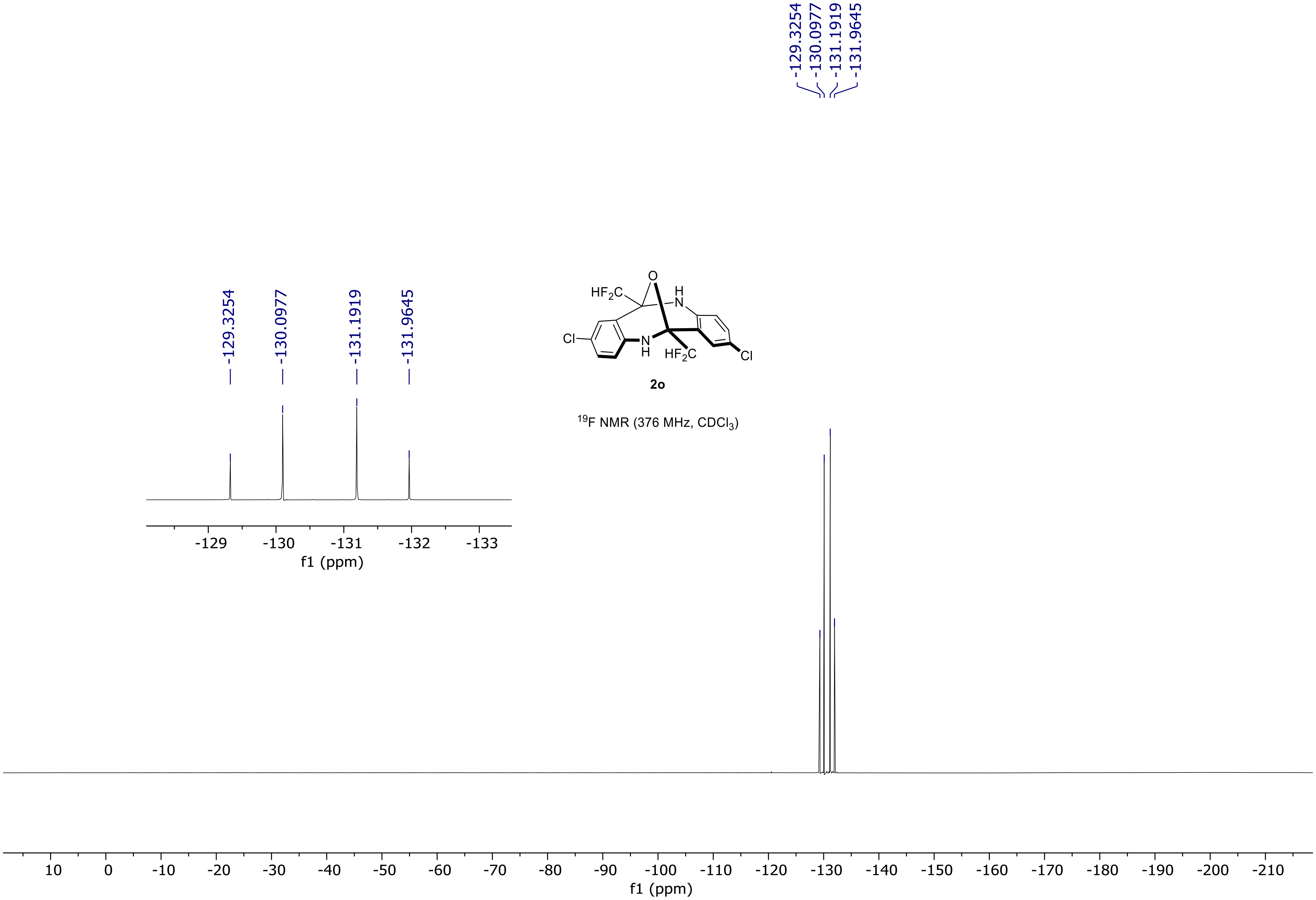
-129.3254
-130.0977
-131.1919
-131.9645
HF2C
O
H
N
Cl
N
H
HF2C
Cl
2o
19F NMR (376 MHz, CDCl3)
-129
-130
-131
-132
-133
f1 (ppm)
10 0 -10 -20 -30 -40 -50 -60 -70 -80 -90 -100 -110 -120 -130 -140 -150 -160 -170 -180 -190 -200 -210
f1 (ppm)

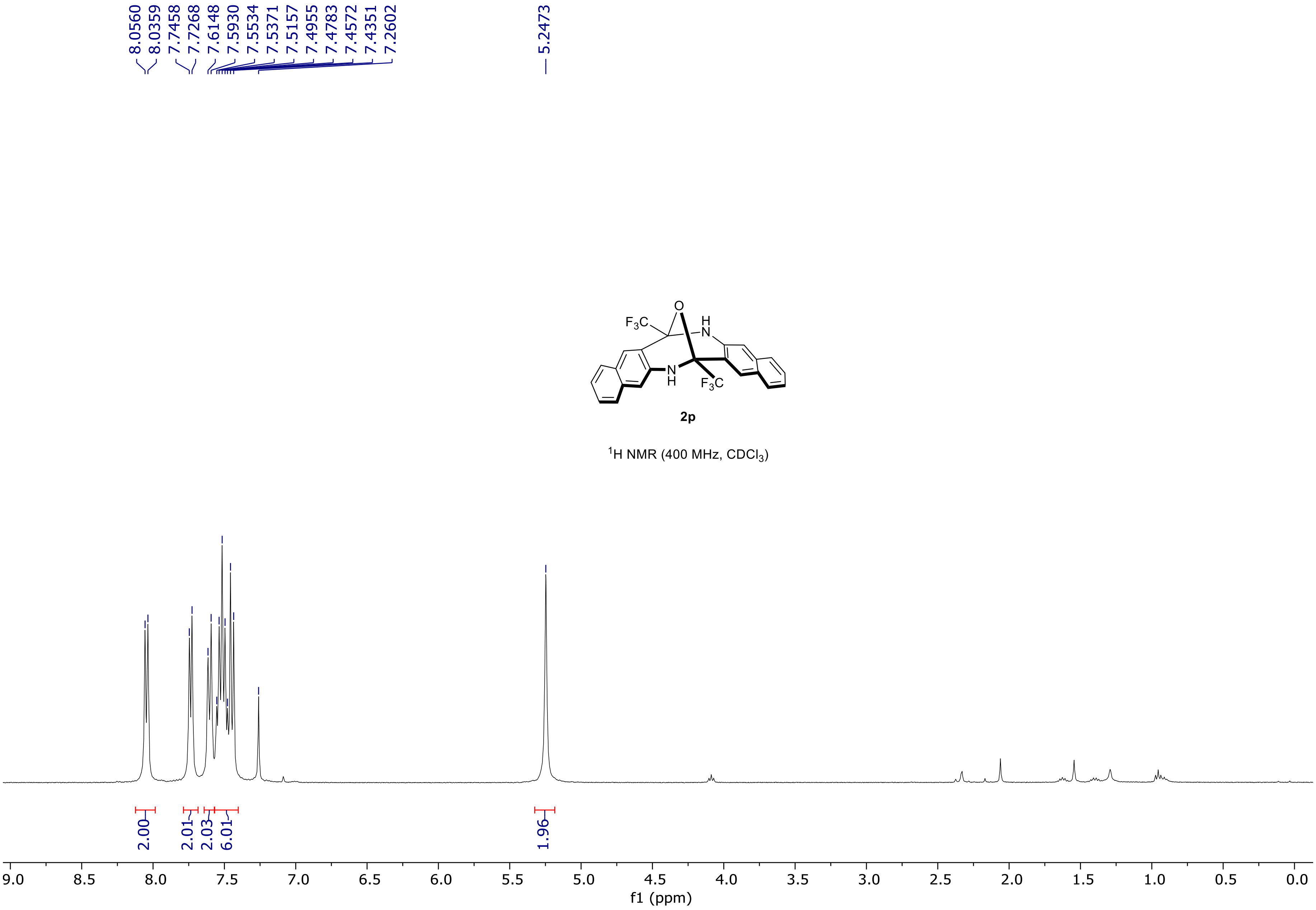
8.0560
8.0359
7.7458
7.7268
7.6148
7.5930
7.5534
7.5371
7.5157
7.4955
7.4783
7.4572
7.4351
7.2602
5.2473
$F_3C$
O
H
N
N
H
$F_3C$
2p
$^1$H NMR (400 MHz, $CDCl_3$)
2.00
2.01
2.03
6.01
1.96
9.0 8.5 8.0 7.5 7.0 6.5 6.0 5.5 5.0 4.5 4.0 3.5 3.0 2.5 2.0 1.5 1.0 0.5 0.0
f1 (ppm)

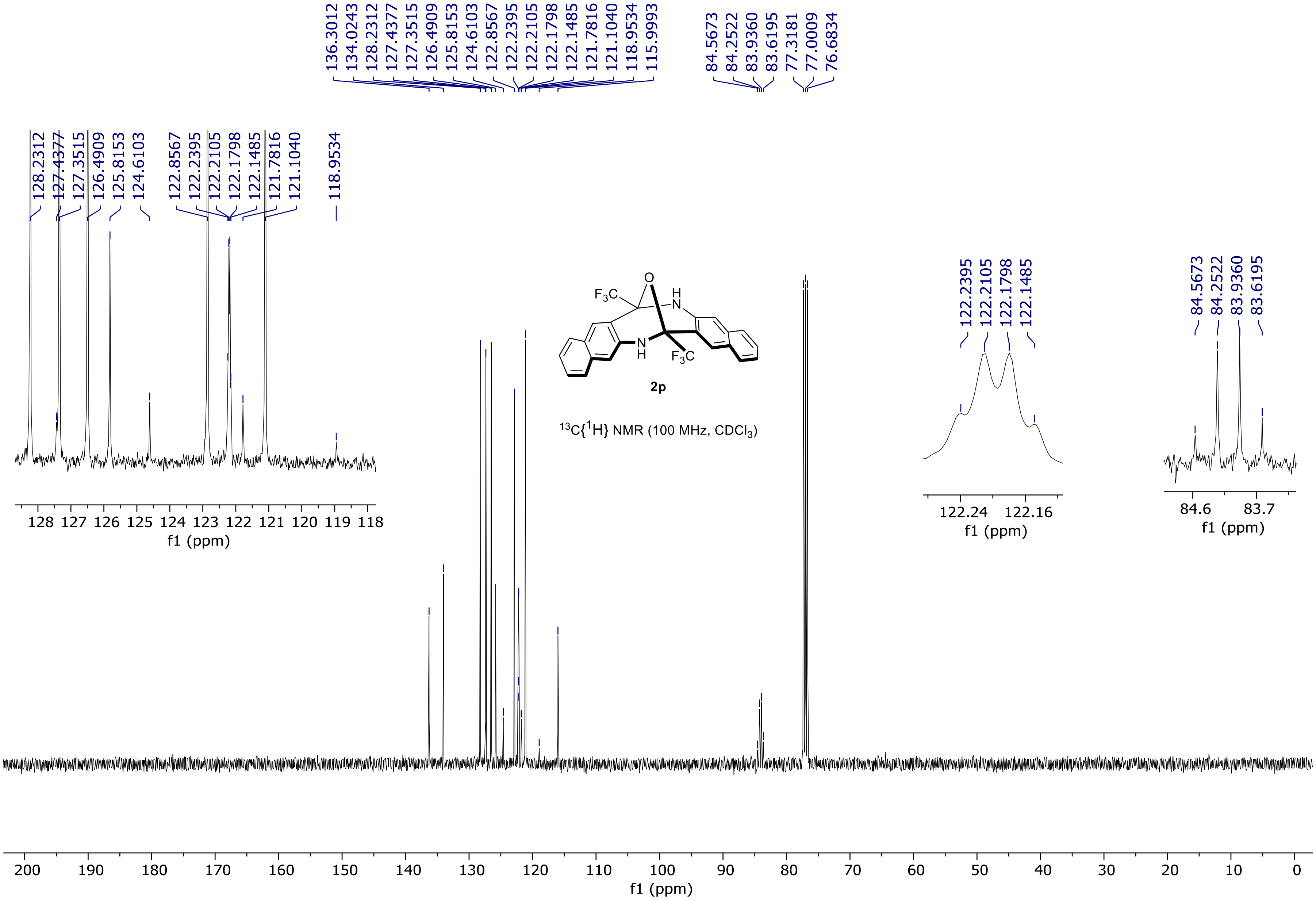
136.3012
134.0243
128.2312
127.4377
127.3515
126.4909
125.8153
124.6103
122.8567
122.2395
122.2105
122.1798
122.1485
121.7816
121.1040
118.9534
115.9993
84.5673
84.2522
83.9360
83.6195
77.3181
77.0009
76.6834
2p
$^{13}C\{^{1}H\}$ NMR (100 MHz, $CDCl_3$)
f1 (ppm)

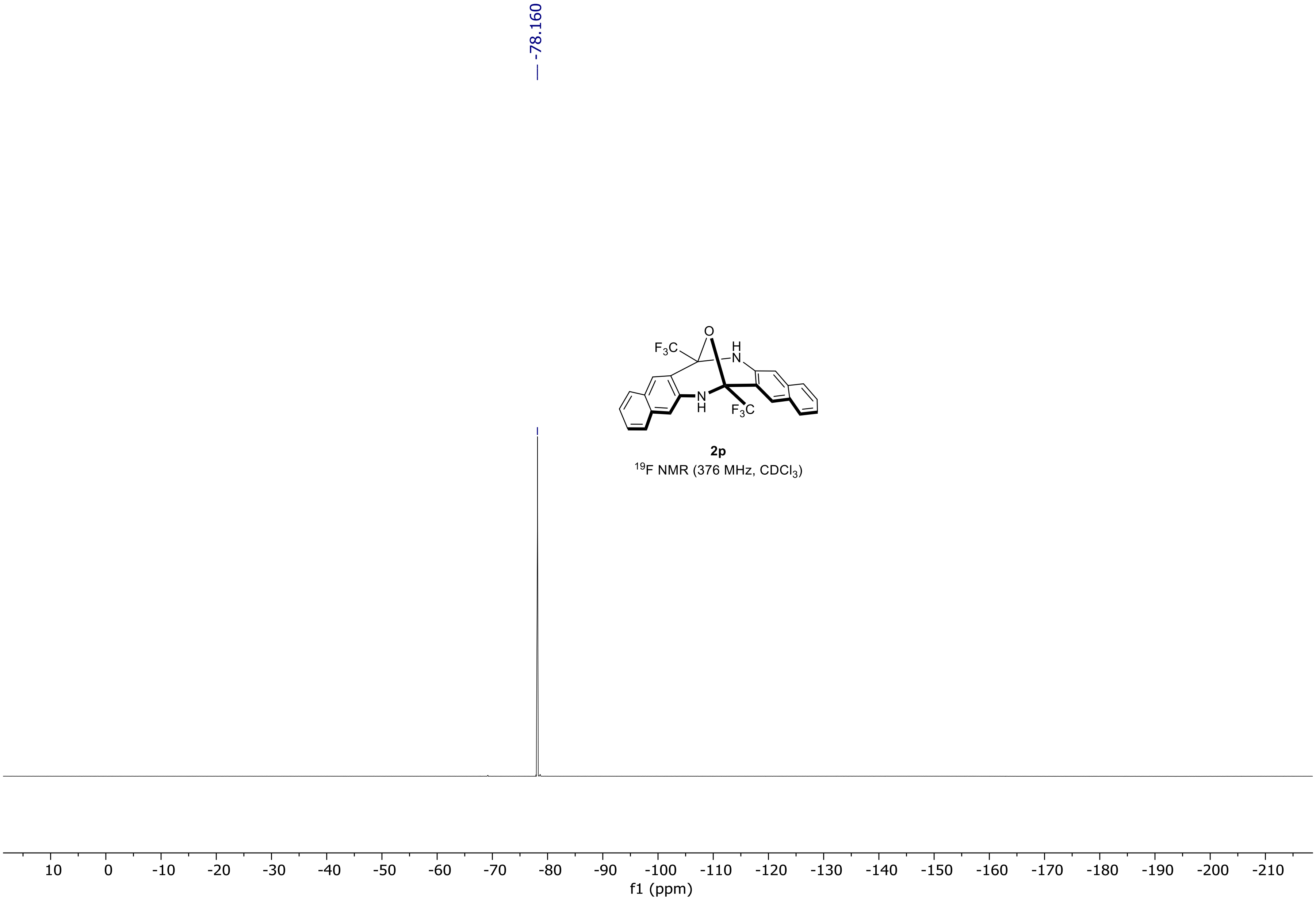
-78.160
F3C
O
H
N
N
H
F3C
2p
19F NMR (376 MHz, CDCl3)
10 0 -10 -20 -30 -40 -50 -60 -70 -80 -90 -100 -110 -120 -130 -140 -150 -160 -170 -180 -190 -200 -210
f1 (ppm)

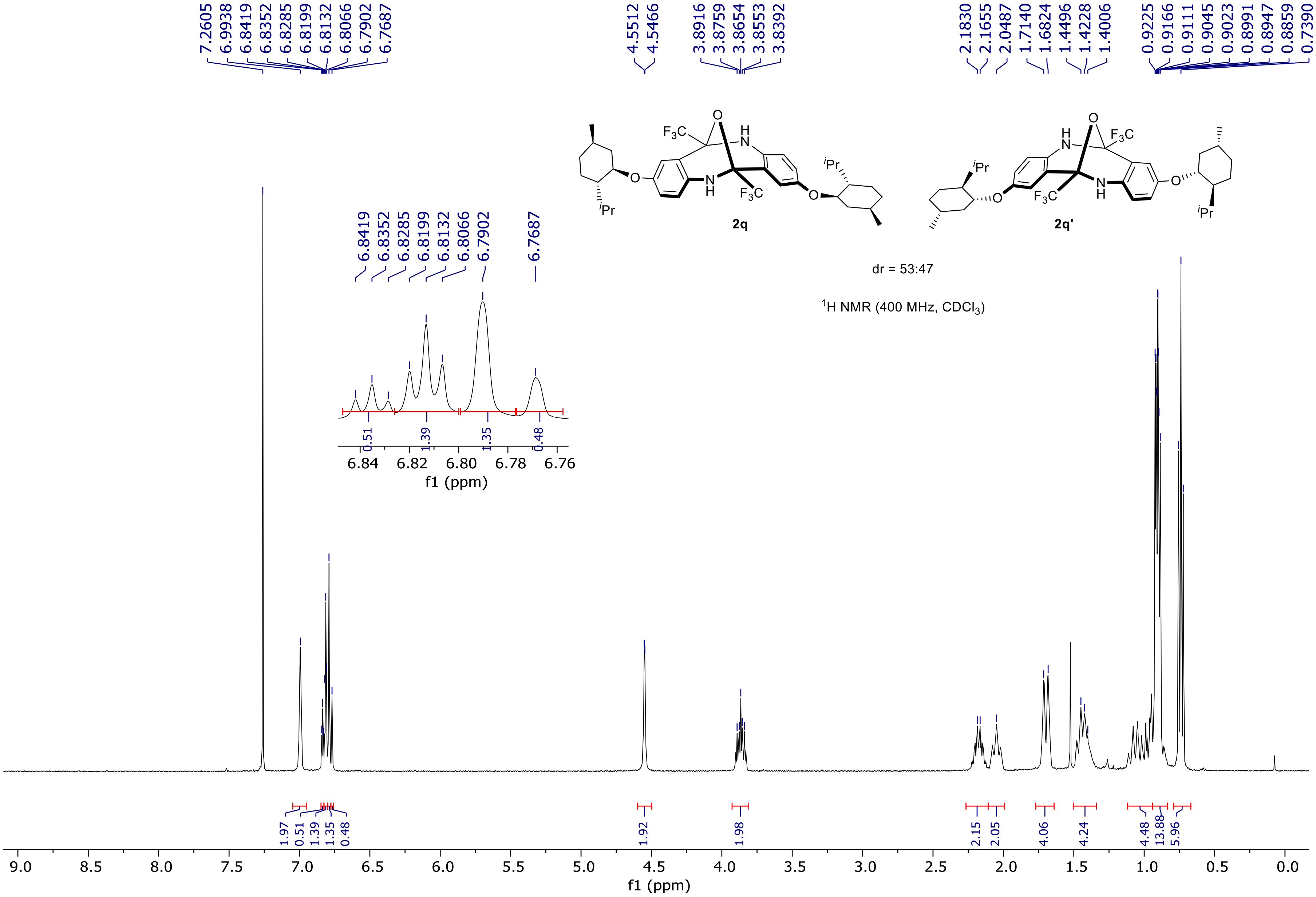
7.2605
6.9938
6.8419
6.8352
6.8285
6.8199
6.8132
6.8066
6.7902
6.7687
4.5512
4.5466
3.8916
3.8759
3.8654
3.8553
3.8392
2.1830
2.1655
2.0487
1.7140
1.6824
1.4496
1.4228
1.4006
0.9225
0.9166
0.9111
0.9045
0.9023
0.8991
0.8947
0.8859
0.7390
2q
2q'
dr = 53:47
1H NMR (400 MHz, CDCl3)
1.97
0.51
1.39
1.35
0.48
1.92
1.98
2.15
2.05
4.06
4.24
4.48
13.88
5.96
f1 (ppm)

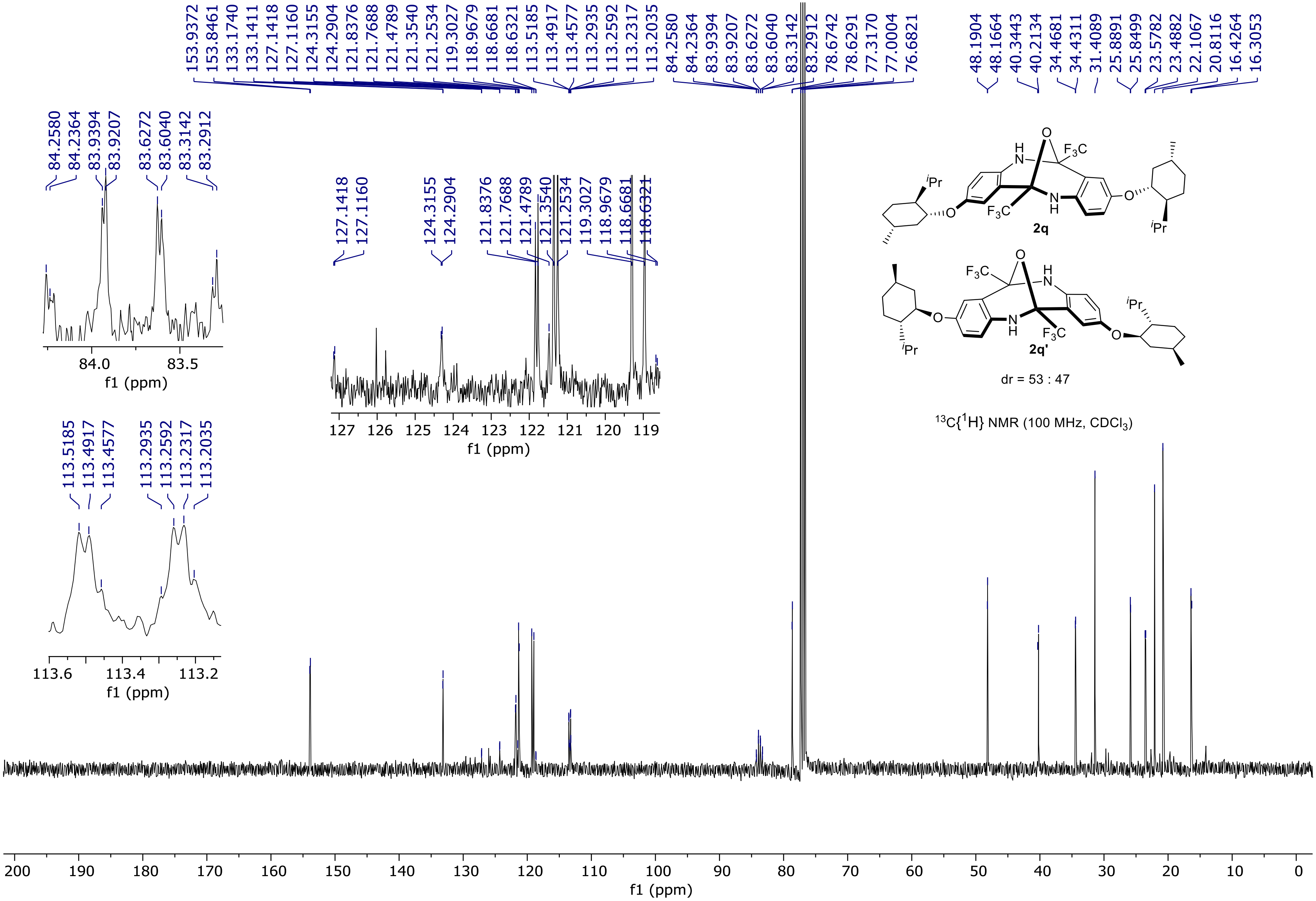
153.9372
153.8461
133.1740
133.1411
127.1418
127.1160
124.3155
124.2904
121.8376
121.7688
121.4789
121.3540
121.2534
119.3027
118.9679
118.6681
118.6321
113.5185
113.4917
113.4577
113.2935
113.2592
113.2317
113.2035
84.2580
84.2364
83.9394
83.9207
83.6272
83.6040
83.3142
83.2912
78.6742
78.6291
77.3170
77.0004
76.6821
48.1904
48.1664
40.3443
40.2134
34.4681
34.4311
31.4089
25.8891
25.8499
23.5782
23.4882
22.1067
20.8116
16.4264
16.3053
2q
2q'
dr = 53 : 47
$^{13}C\{^{1}H\}$ NMR (100 MHz, $CDCl_3$)
f1 (ppm)

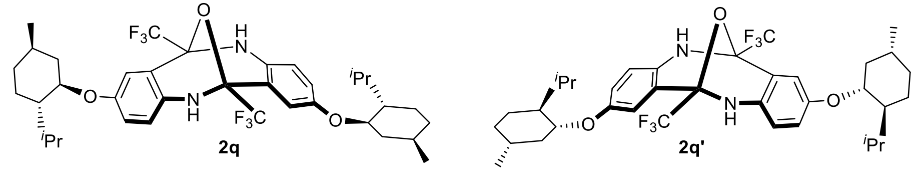


dr = 53 : 47

$^{19}$F NMR (376 MHz, $CDCl_3$)

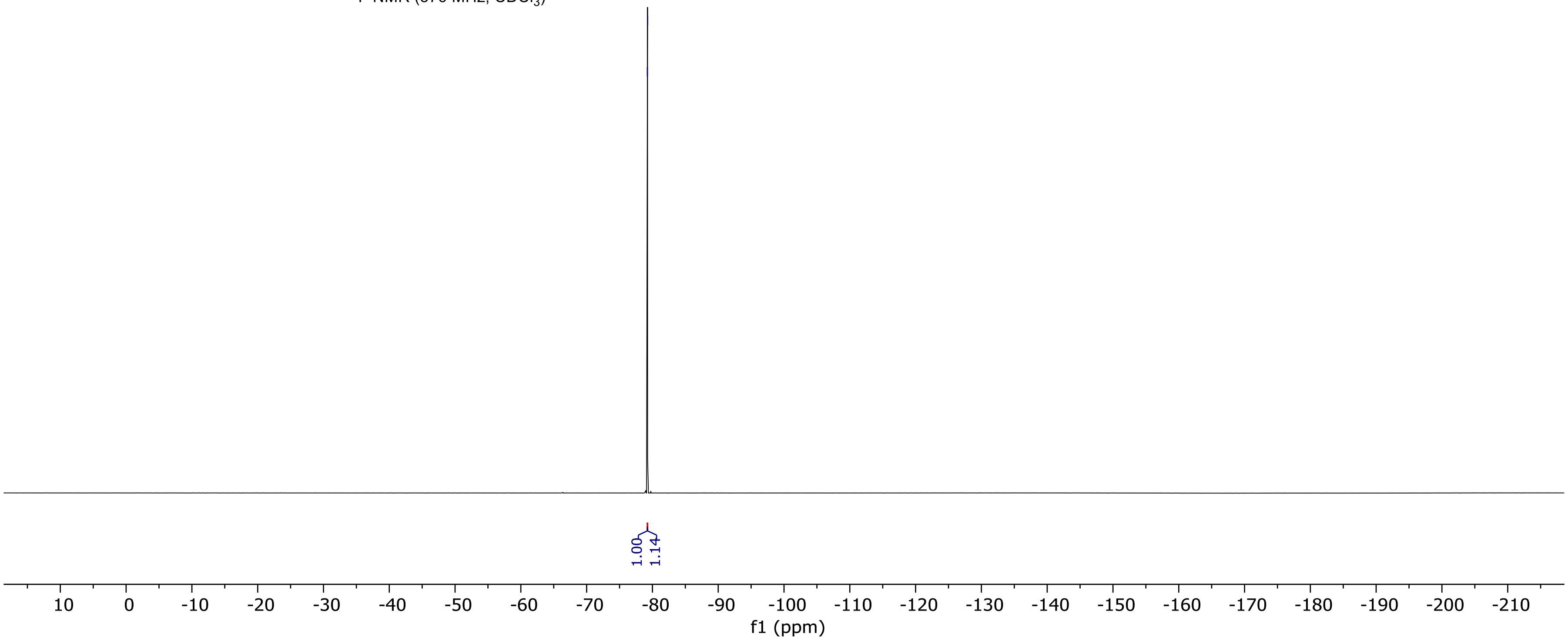


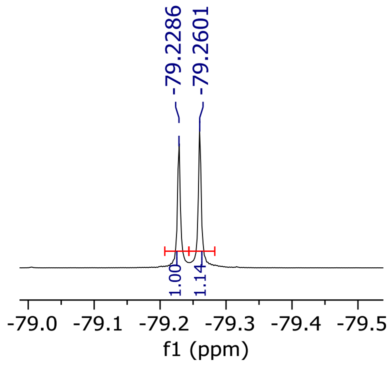

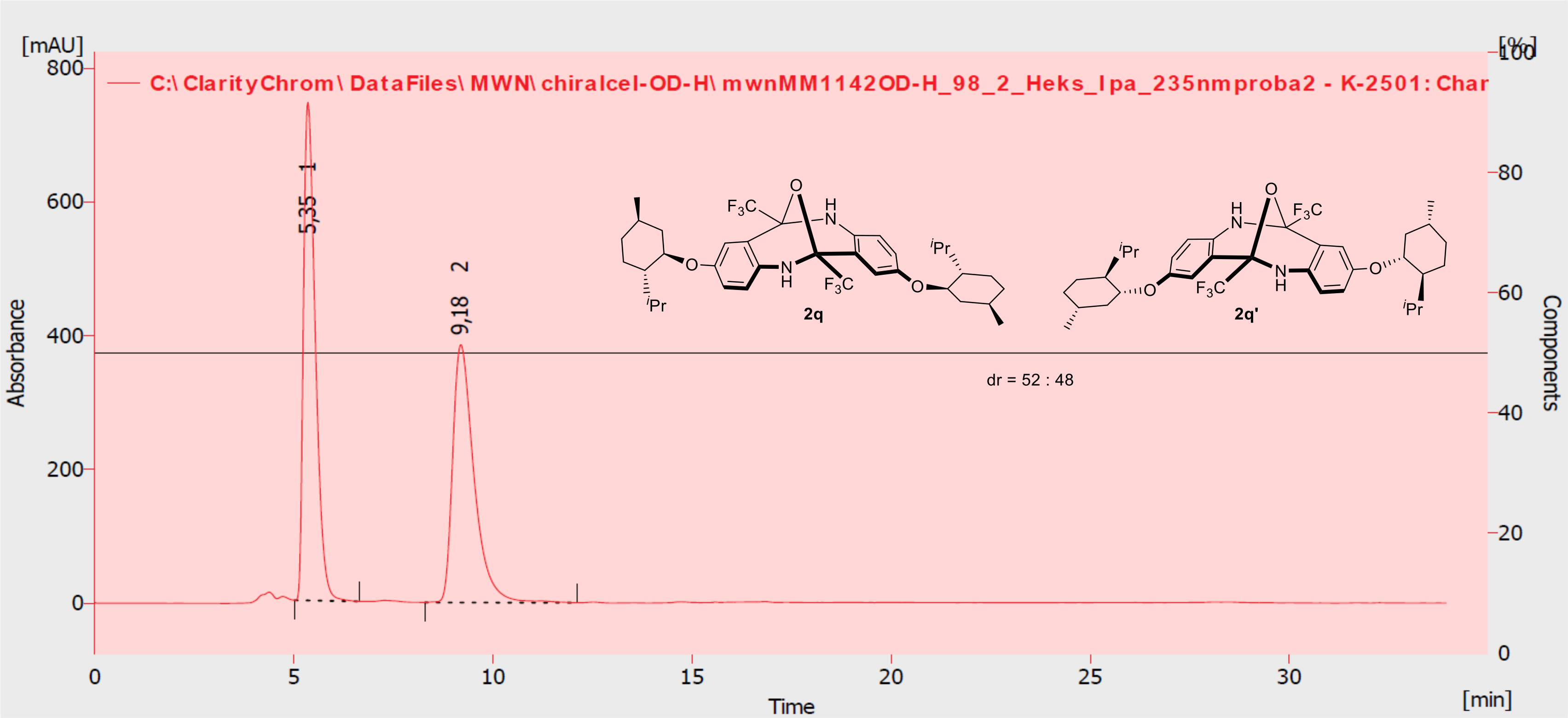


*Result Table (Uncal - C:\ClarityChrom\DataFiles\MWN\chiralcel-OD-H\mwnMM1142OD-H_98_2_Heks_Ipa_235nmproba2 - K-2501: Channel 1)*

| | Reten. Time [min] | Area [mAU.s] | Height [mAU] | Area [%] | Height [%] | W05 [min] | Compound Name |
|---|---|---|---|---|---|---|---|
| 1 | 5,350 | 15468,068 | 745,004 | 51,7 | 65,9 | 0,33 | |
| 2 | 9,183 | 14441,313 | 385,481 | 48,3 | 34,1 | 0,58 | |
| | Total | 29909,382 | 1130,486 | 100,0 | 100,0 | | |

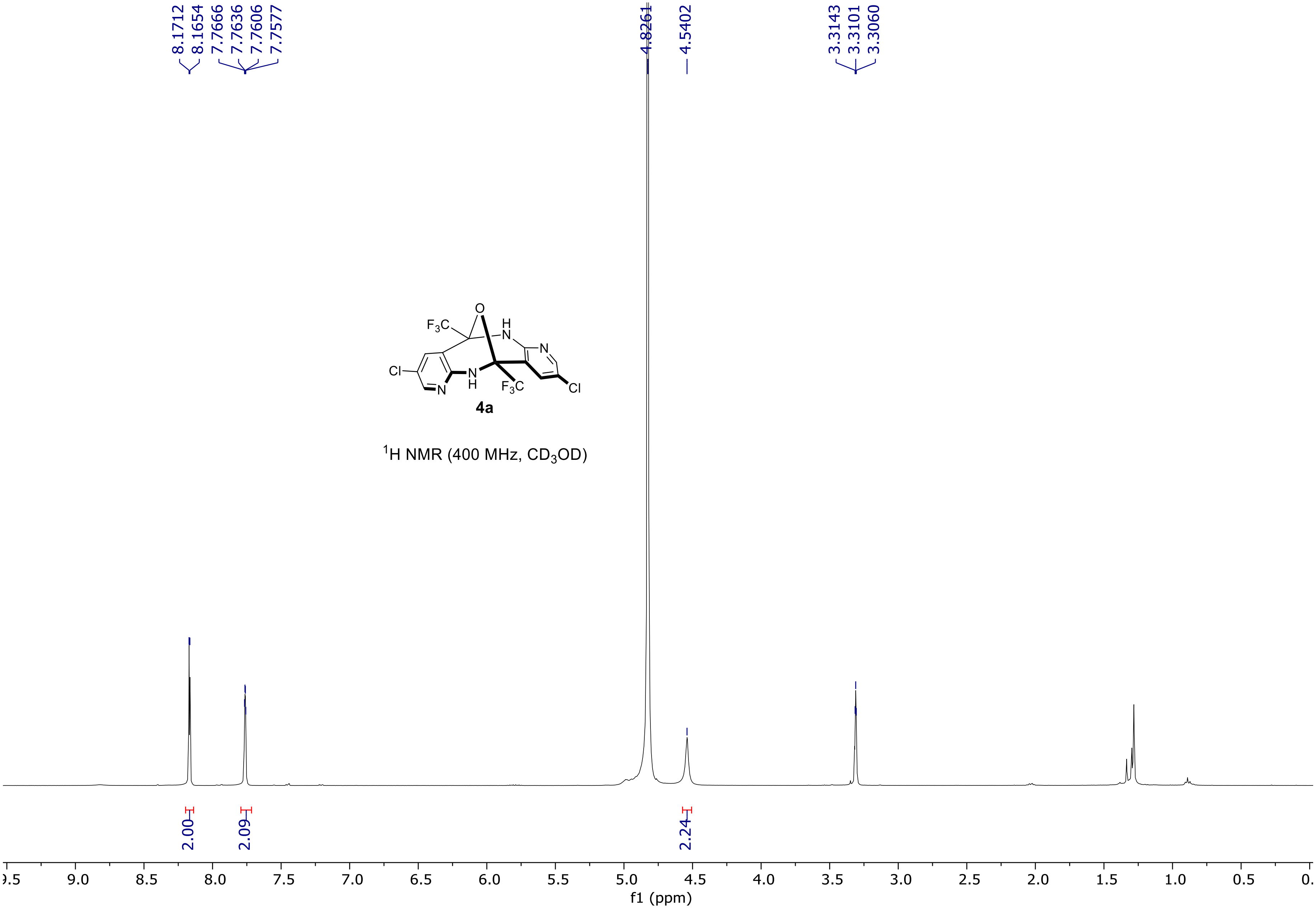
8.1712
8.1654
7.7666
7.7636
7.7606
7.7577
4.8261
4.5402
3.3143
3.3101
3.3060
4a
1H NMR (400 MHz, CD3OD)
2.00
2.09
2.24
f1 (ppm)

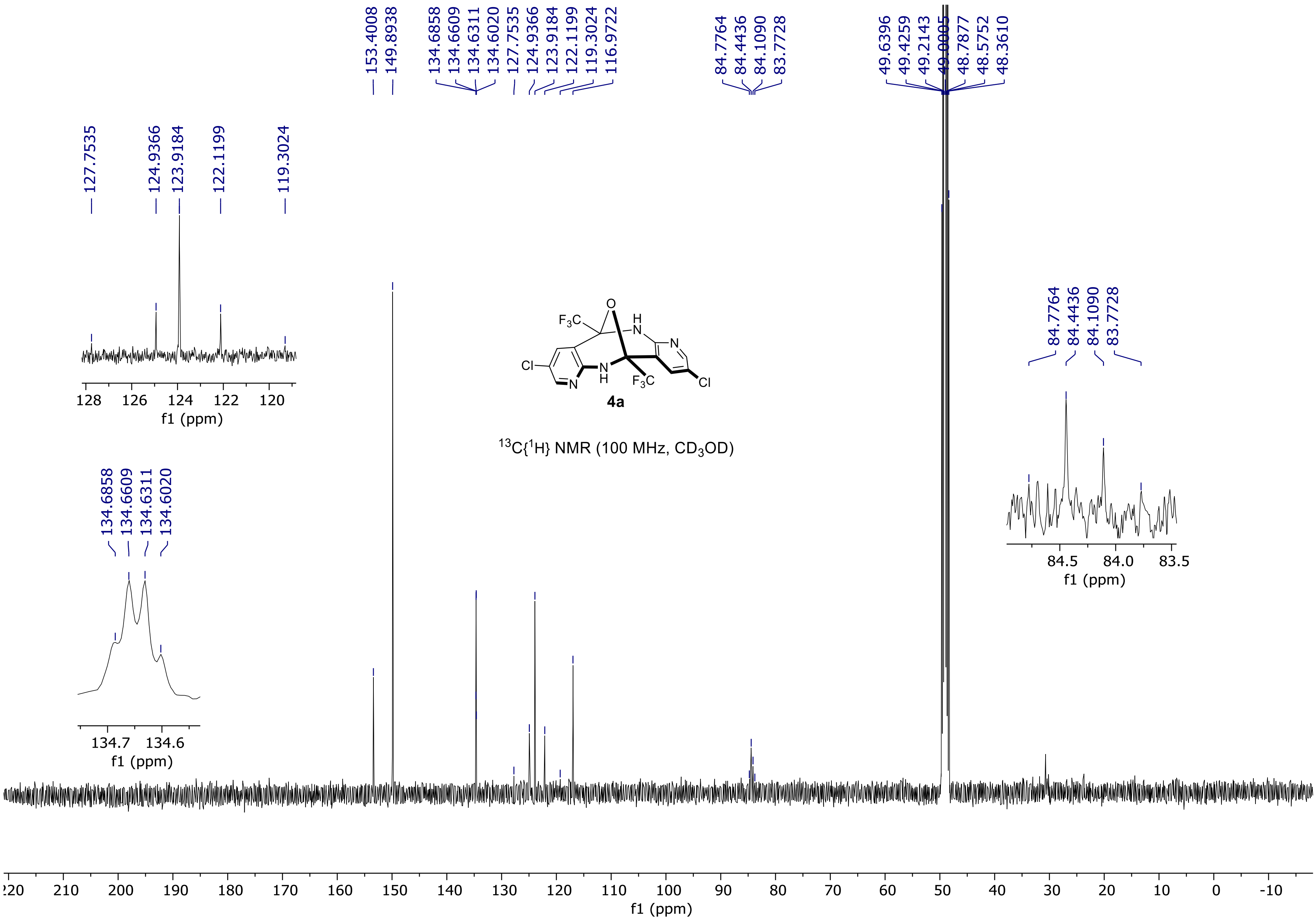
153.4008
149.8938
134.6858
134.6609
134.6311
134.6020
127.7535
124.9366
123.9184
122.1199
119.3024
116.9722
84.7764
84.4436
84.1090
83.7728
49.6396
49.4259
49.2143
49.0005
48.7877
48.5752
48.3610
4a
$^{13}C\{^1H\}$ NMR (100 MHz, $CD_3OD$)
f1 (ppm)

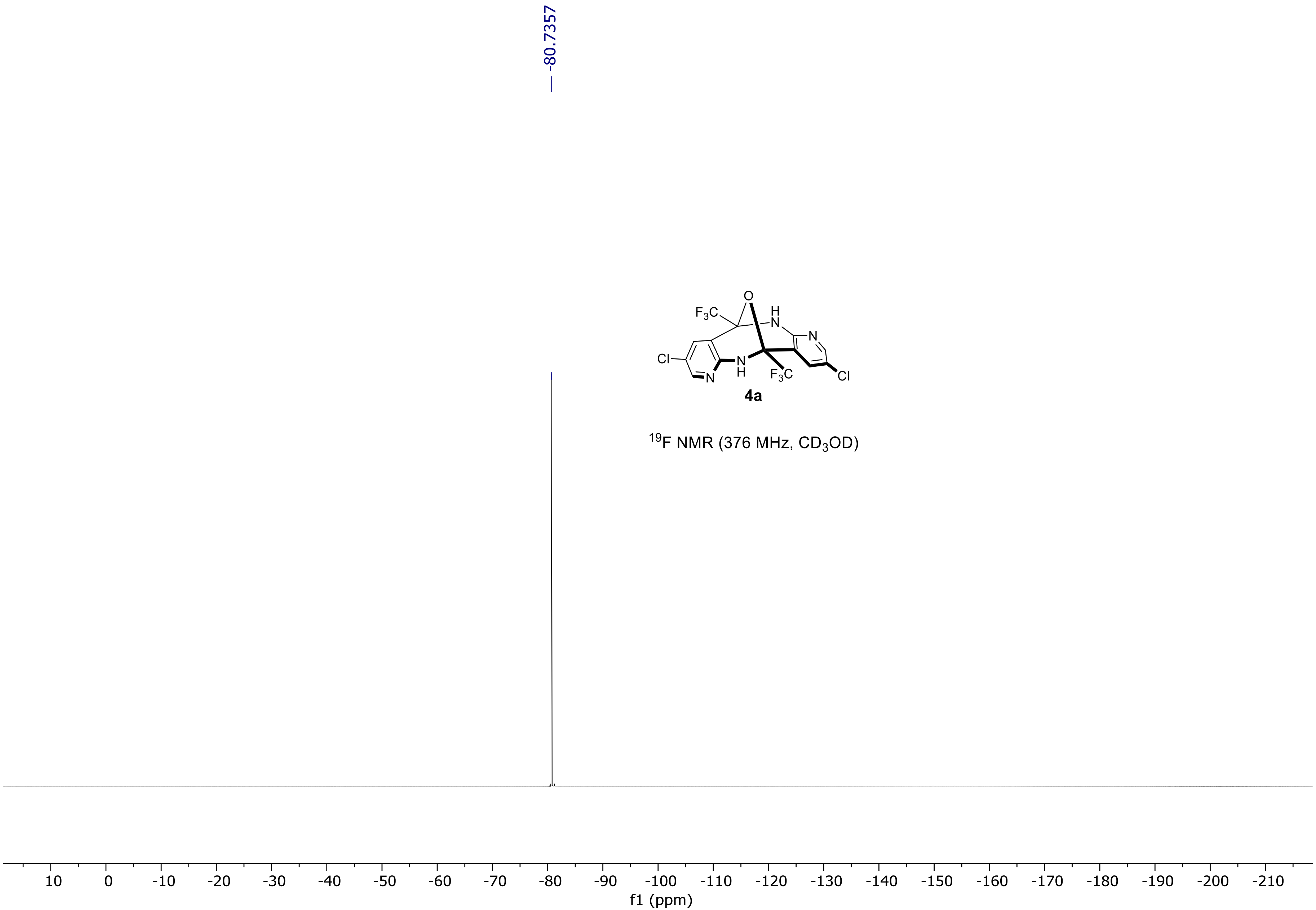
-80.7357
F3C
O
H
N
N
Cl
N
H
N
F3C
Cl
4a
19F NMR (376 MHz, CD3OD)
10 0 -10 -20 -30 -40 -50 -60 -70 -80 -90 -100 -110 -120 -130 -140 -150 -160 -170 -180 -190 -200 -210
f1 (ppm)

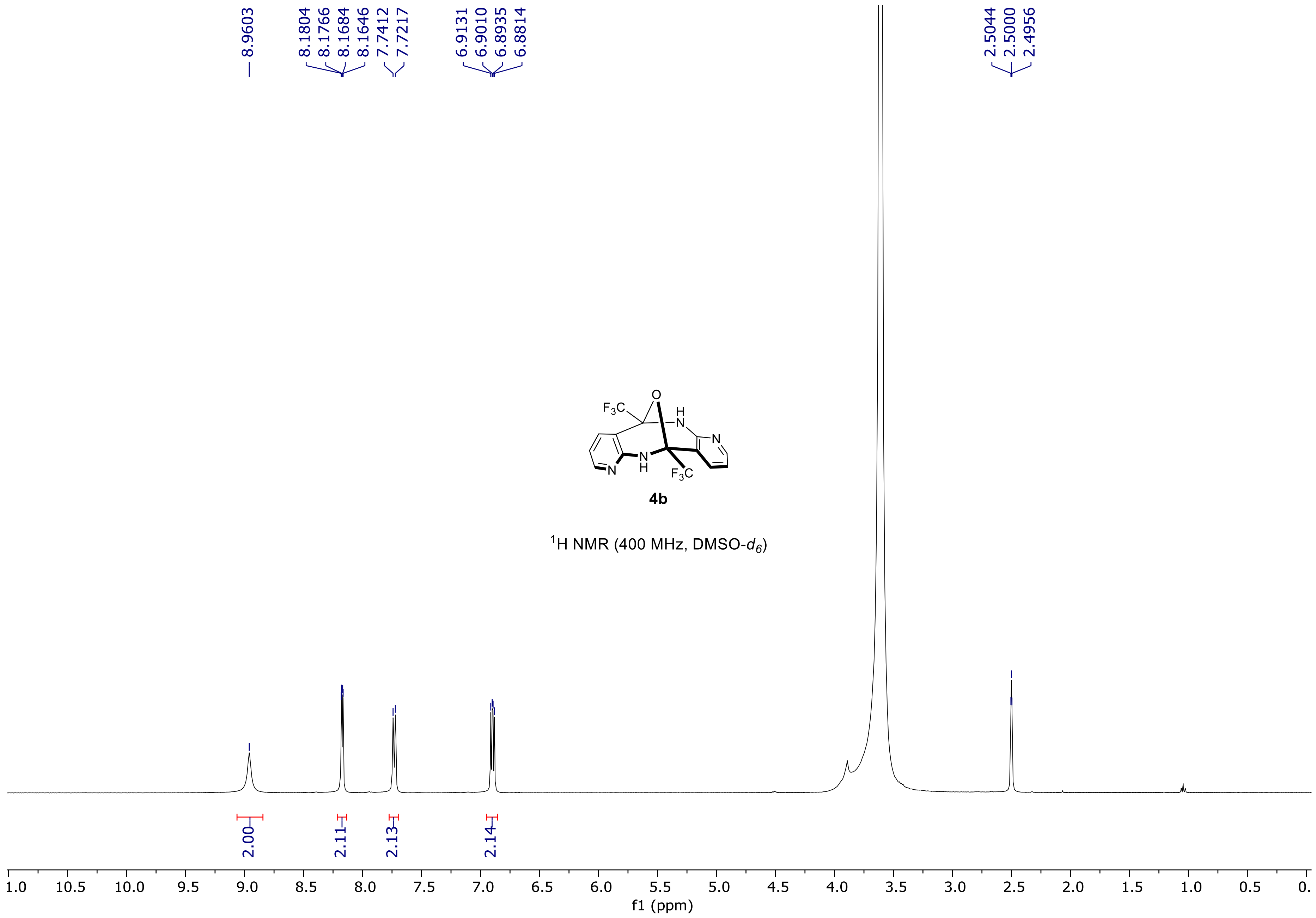
8.9603
8.1804
8.1766
8.1684
8.1646
7.7412
7.7217
6.9131
6.9010
6.8935
6.8814
2.5044
2.5000
2.4956
F3C
O
H
N
N
N
H
F3C
4b
1H NMR (400 MHz, DMSO-d6)
2.00
2.11
2.13
2.14
1.0 10.5 10.0 9.5 9.0 8.5 8.0 7.5 7.0 6.5 6.0 5.5 5.0 4.5 4.0 3.5 3.0 2.5 2.0 1.5 1.0 0.5 0.
f1 (ppm)

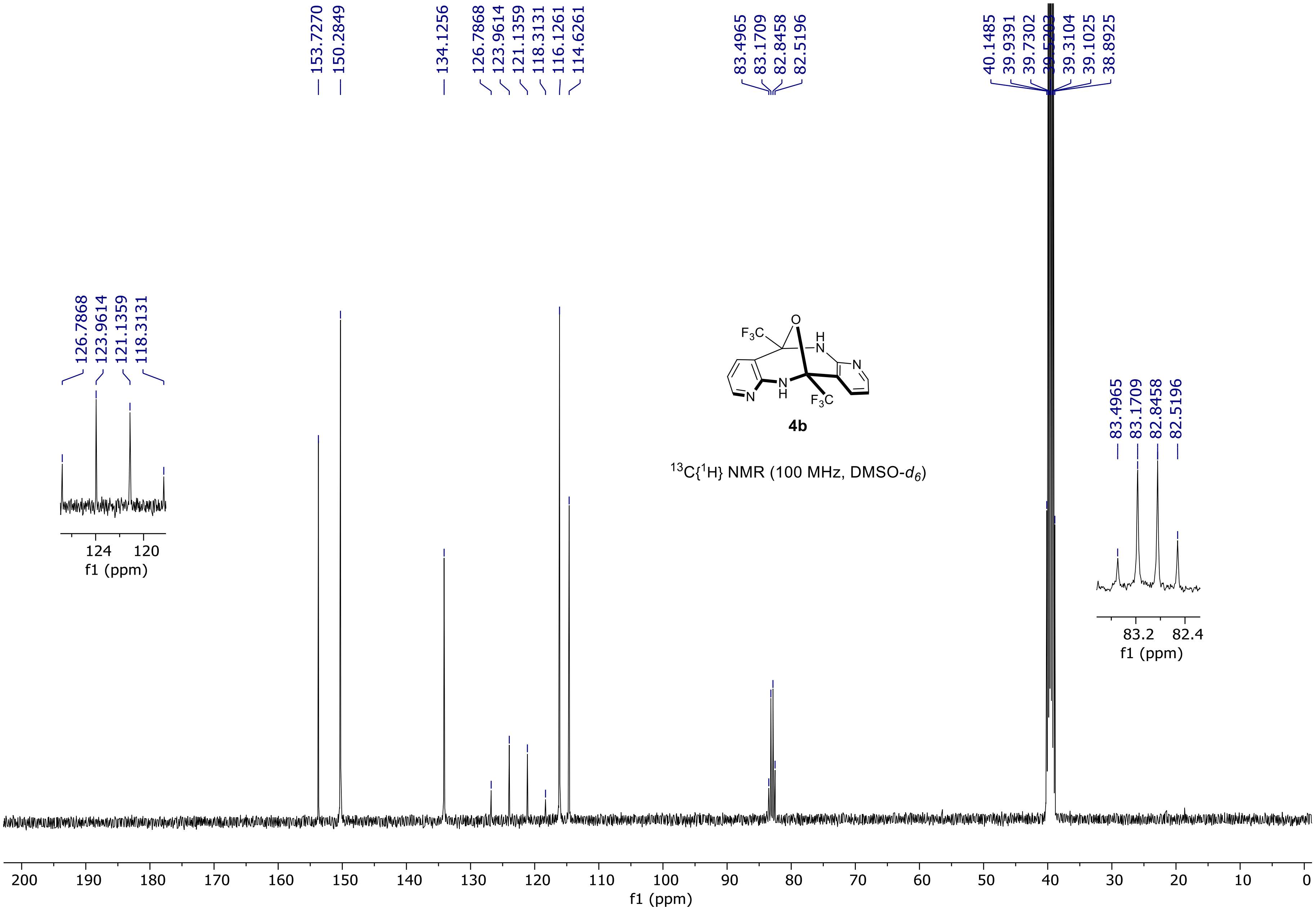
153.7270
150.2849
134.1256
126.7868
123.9614
121.1359
118.3131
116.1261
114.6261
83.4965
83.1709
82.8458
82.5196
40.1485
39.9391
39.7302
39.5203
39.3104
39.1025
38.8925
126.7868
123.9614
121.1359
118.3131
124
120
f1 (ppm)
F3C
O
H
N
N
N
H
F3C
4b
13C{1H} NMR (100 MHz, DMSO-d6)
83.4965
83.1709
82.8458
82.5196
83.2
82.4
f1 (ppm)
200
190
180
170
160
150
140
130
120
110
100
90
80
70
60
50
40
30
20
10
0
f1 (ppm)

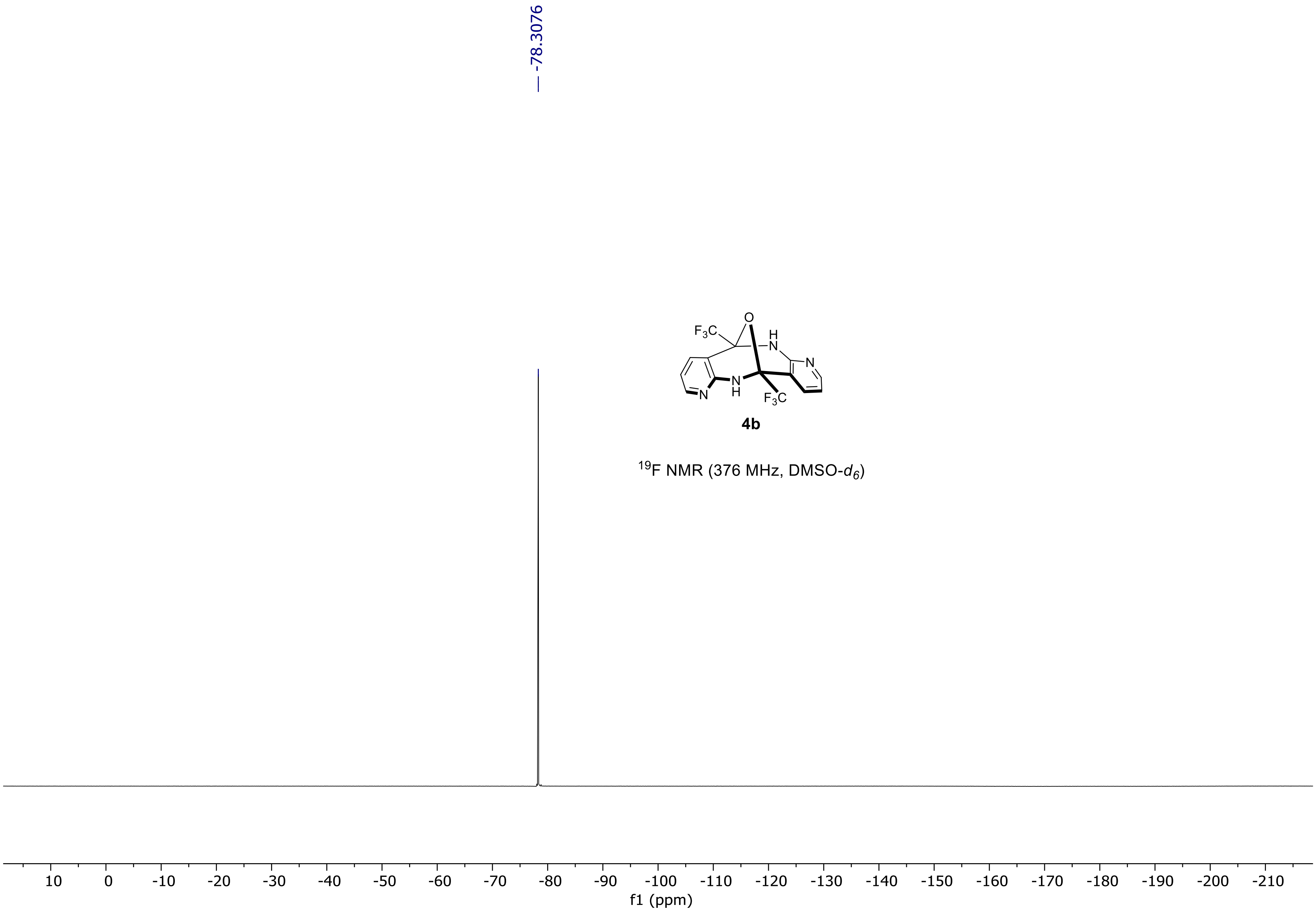
-78.3076
F3C
O
H
N
N
N
H
F3C
4b
19F NMR (376 MHz, DMSO-d6)
10 0 -10 -20 -30 -40 -50 -60 -70 -80 -90 -100 -110 -120 -130 -140 -150 -160 -170 -180 -190 -200 -210
f1 (ppm)

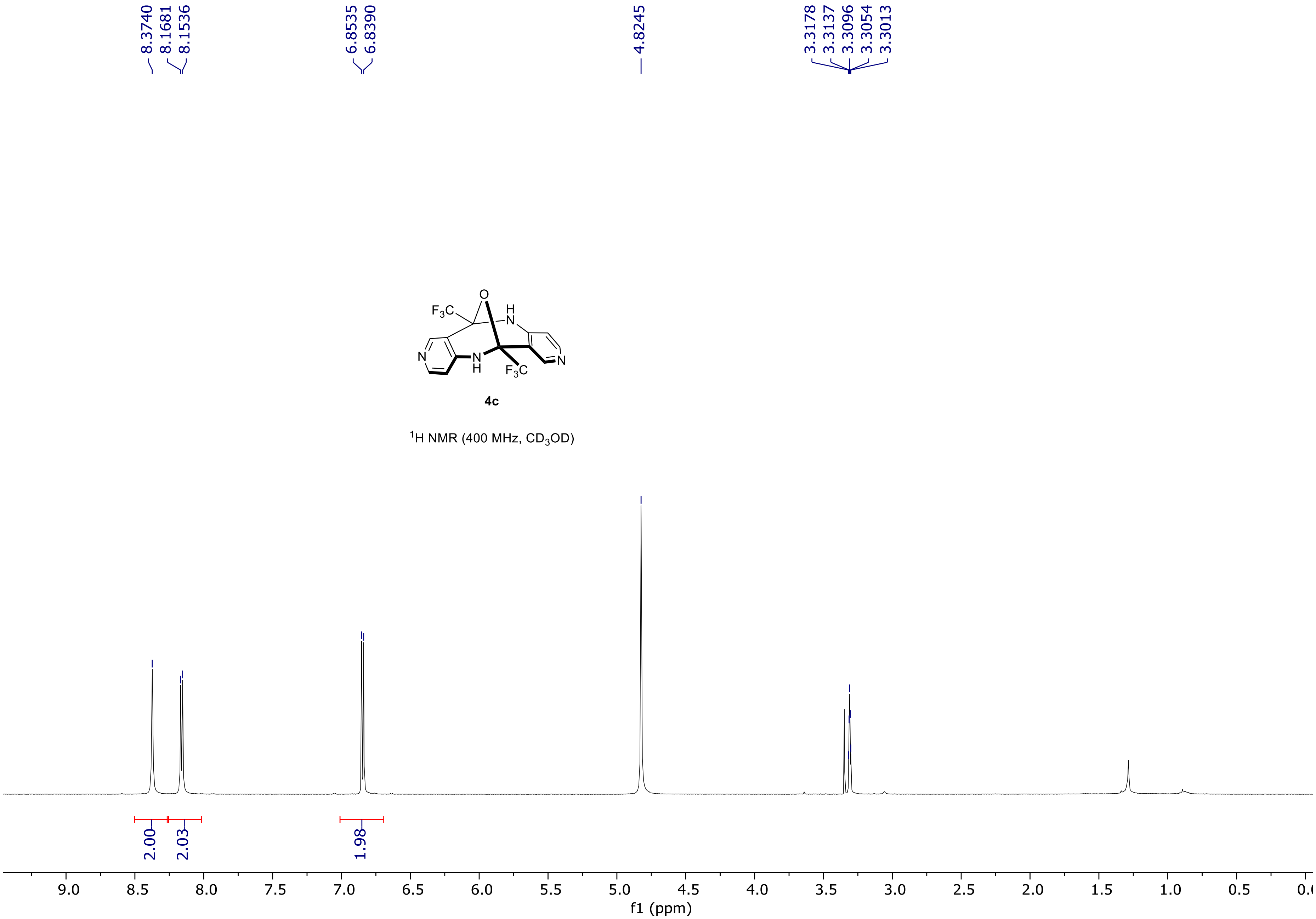
8.3740
8.1681
8.1536
6.8535
6.8390
4.8245
3.3178
3.3137
3.3096
3.3054
3.3013
F3C
O
H
N
N
N
H
F3C
N
4c
1H NMR (400 MHz, CD3OD)
2.00
2.03
1.98
9.0
8.5
8.0
7.5
7.0
6.5
6.0
5.5
5.0
4.5
4.0
3.5
3.0
2.5
2.0
1.5
1.0
0.5
0.0
f1 (ppm)

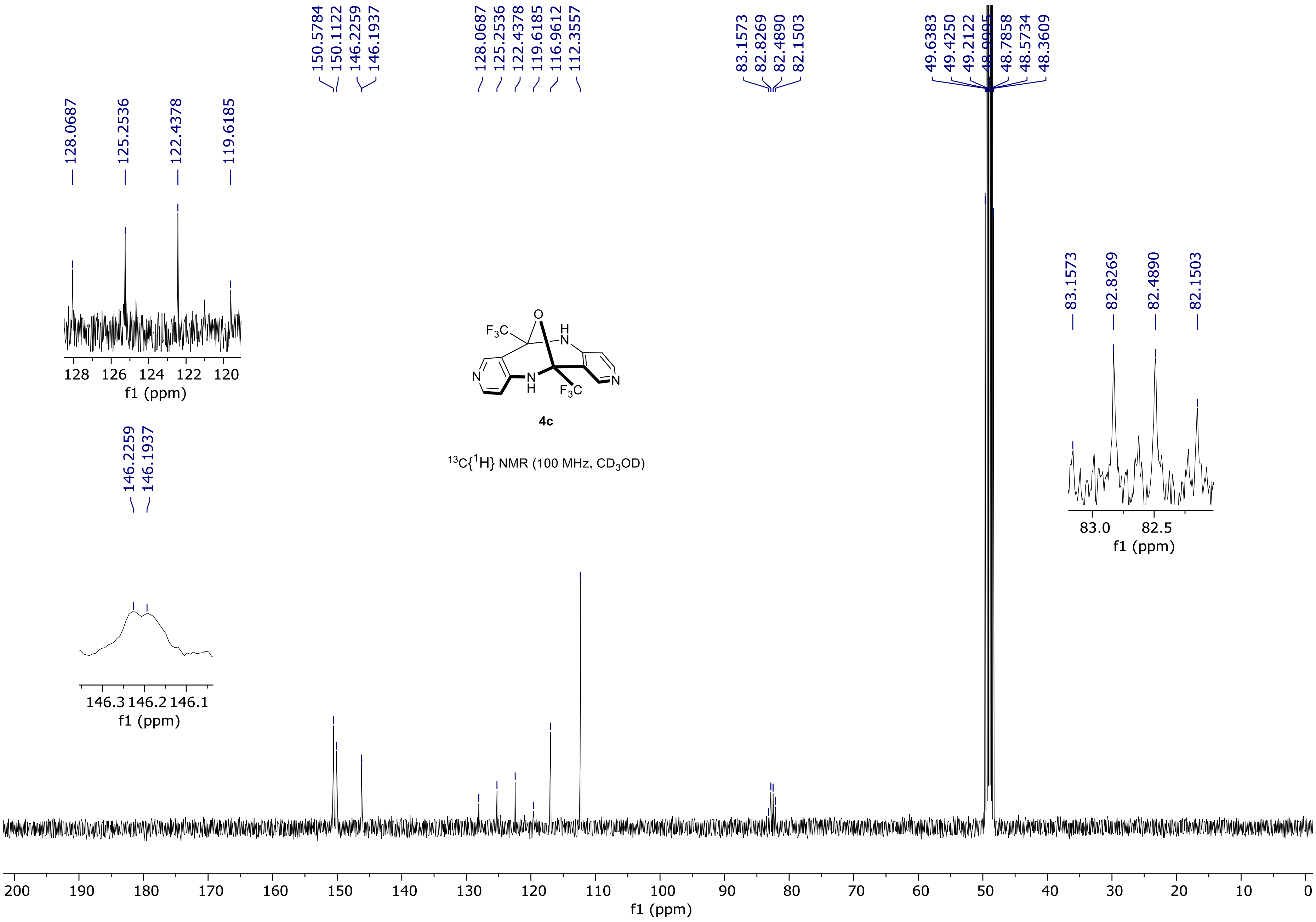
150.5784
150.1122
146.2259
146.1937
128.0687
125.2536
122.4378
119.6185
116.9612
112.3557
83.1573
82.8269
82.4890
82.1503
49.6383
49.4250
49.2122
48.9995
48.7858
48.5734
48.3609
F3C
O
H
N
N
H
F3C
4c
13C{1H} NMR (100 MHz, CD3OD)
f1 (ppm)

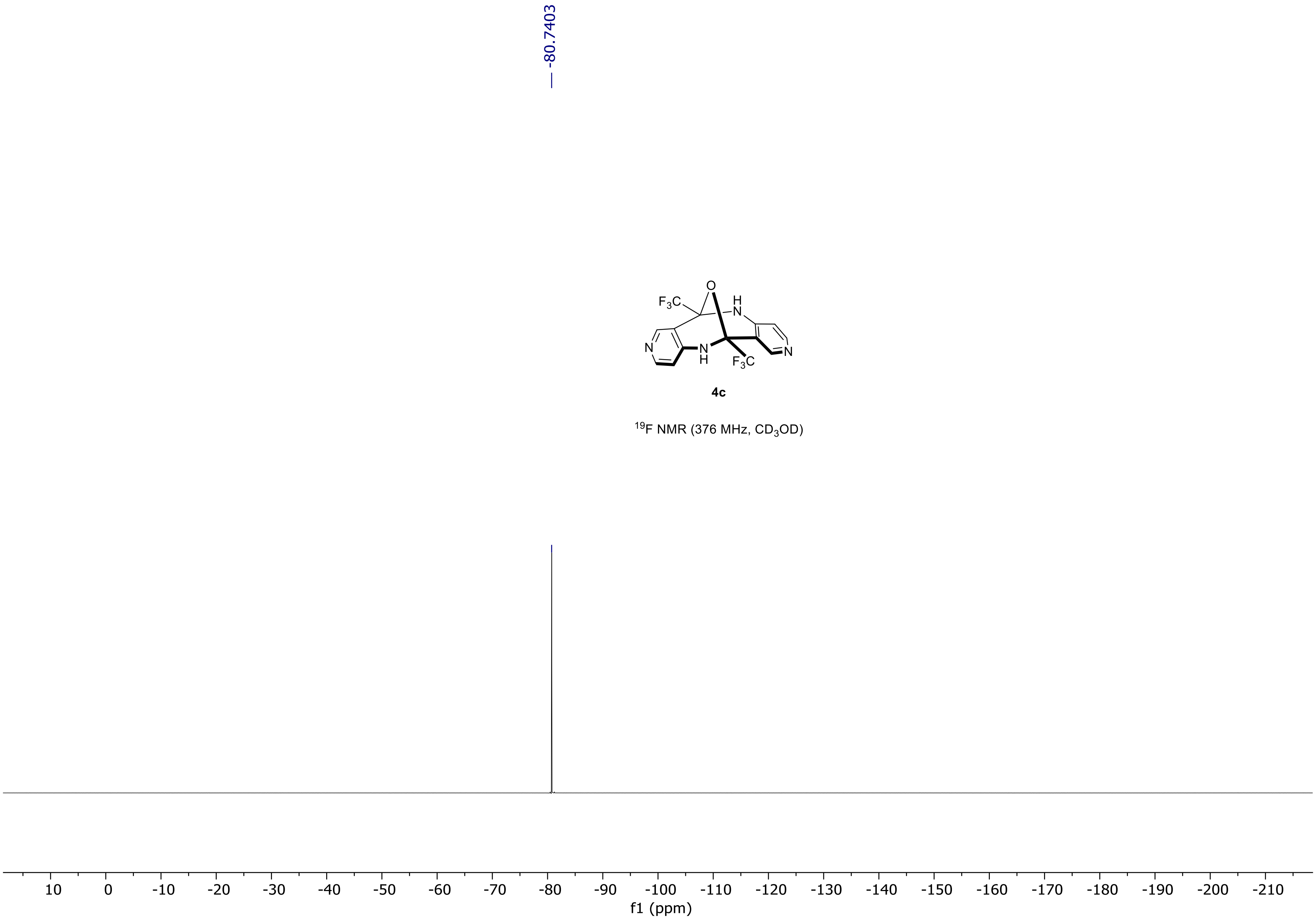


**4c**

$^{19}$F NMR (376 MHz, $CD_3OD$)

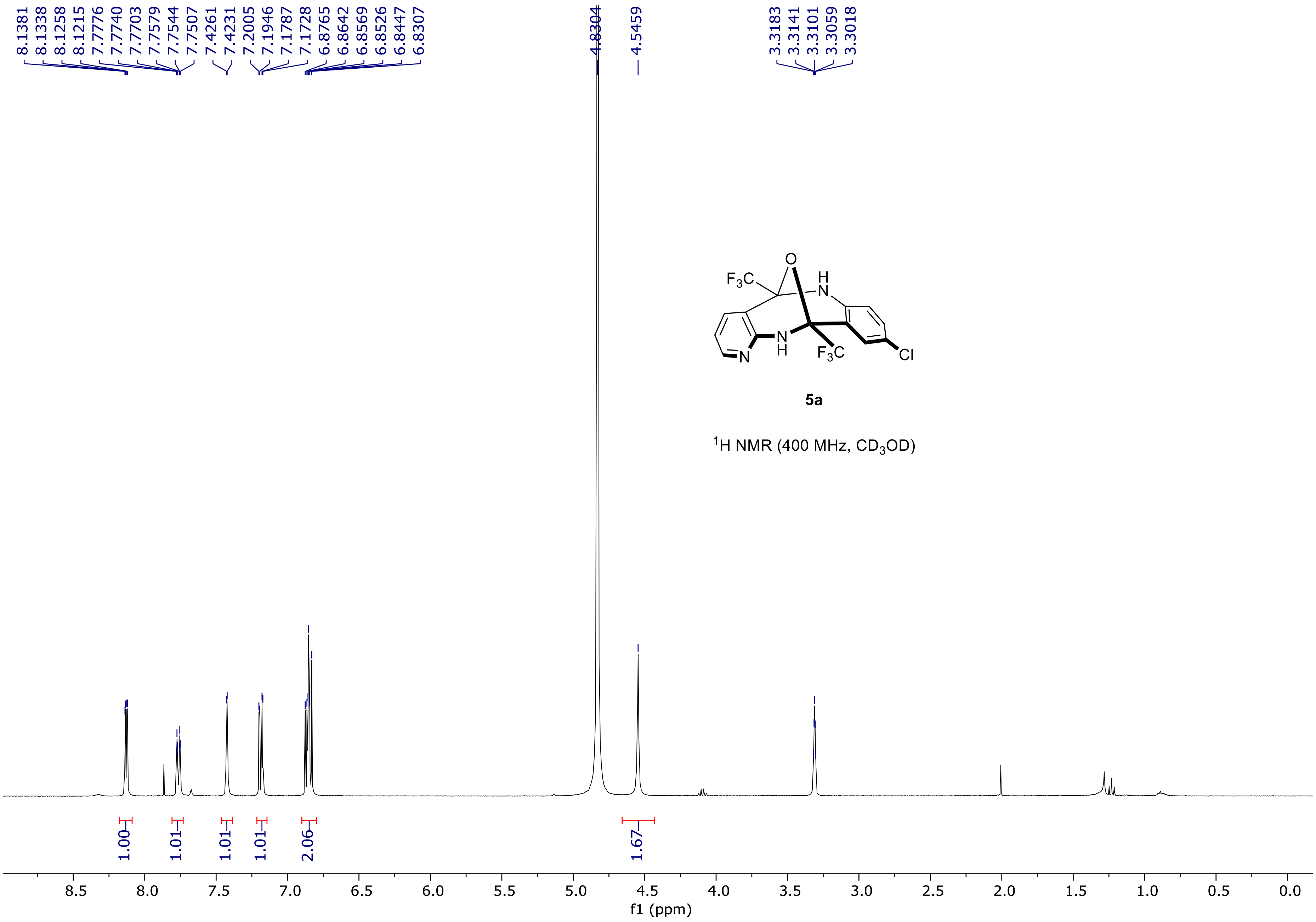
8.1381
8.1338
8.1258
8.1215
7.7776
7.7740
7.7703
7.7579
7.7544
7.7507
7.4261
7.4231
7.2005
7.1946
7.1787
7.1728
6.8765
6.8642
6.8569
6.8526
6.8447
6.8307
4.8304
4.5459
3.3183
3.3141
3.3101
3.3059
3.3018
F3C
O
H
N
N
H
N
F3C
Cl
5a
1H NMR (400 MHz, CD3OD)
1.00
1.01
1.01
1.01
2.06
1.67
8.5
8.0
7.5
7.0
6.5
6.0
5.5
5.0
4.5
4.0
3.5
3.0
2.5
2.0
1.5
1.0
0.5
0.0
f1 (ppm)

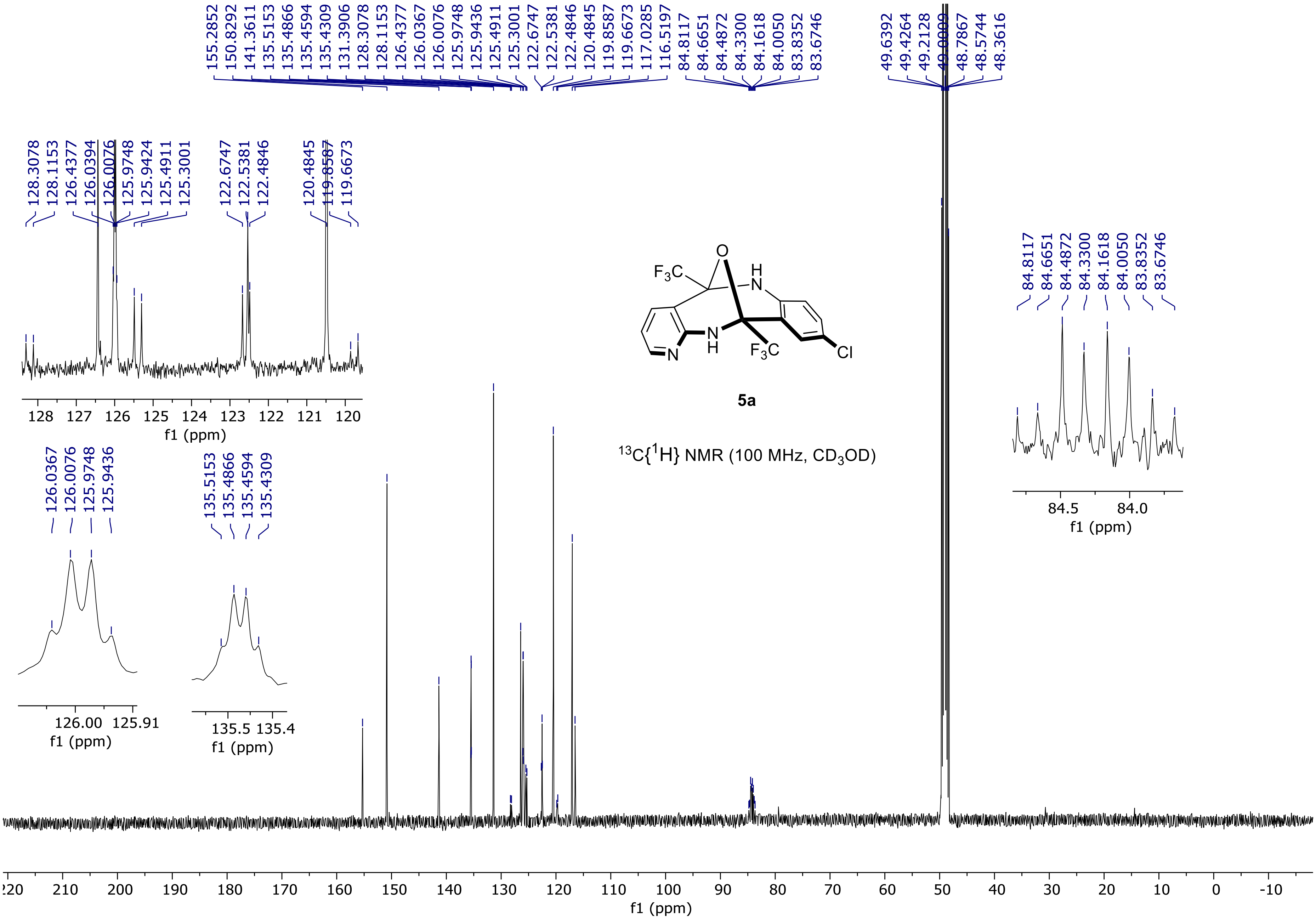
155.2852
150.8292
141.3611
135.5153
135.4866
135.4594
135.4309
131.3906
128.3078
128.1153
126.4377
126.0367
126.0076
125.9748
125.9436
125.4911
125.3001
122.6747
122.5381
122.4846
120.4845
119.8587
119.6673
117.0285
116.5197
84.8117
84.6651
84.4872
84.3300
84.1618
84.0050
83.8352
83.6746
49.6392
49.4264
49.2128
49.0000
48.7867
48.5744
48.3616
5a
$^{13}C\{^1H\}$ NMR (100 MHz, $CD_3OD$)
f1 (ppm)

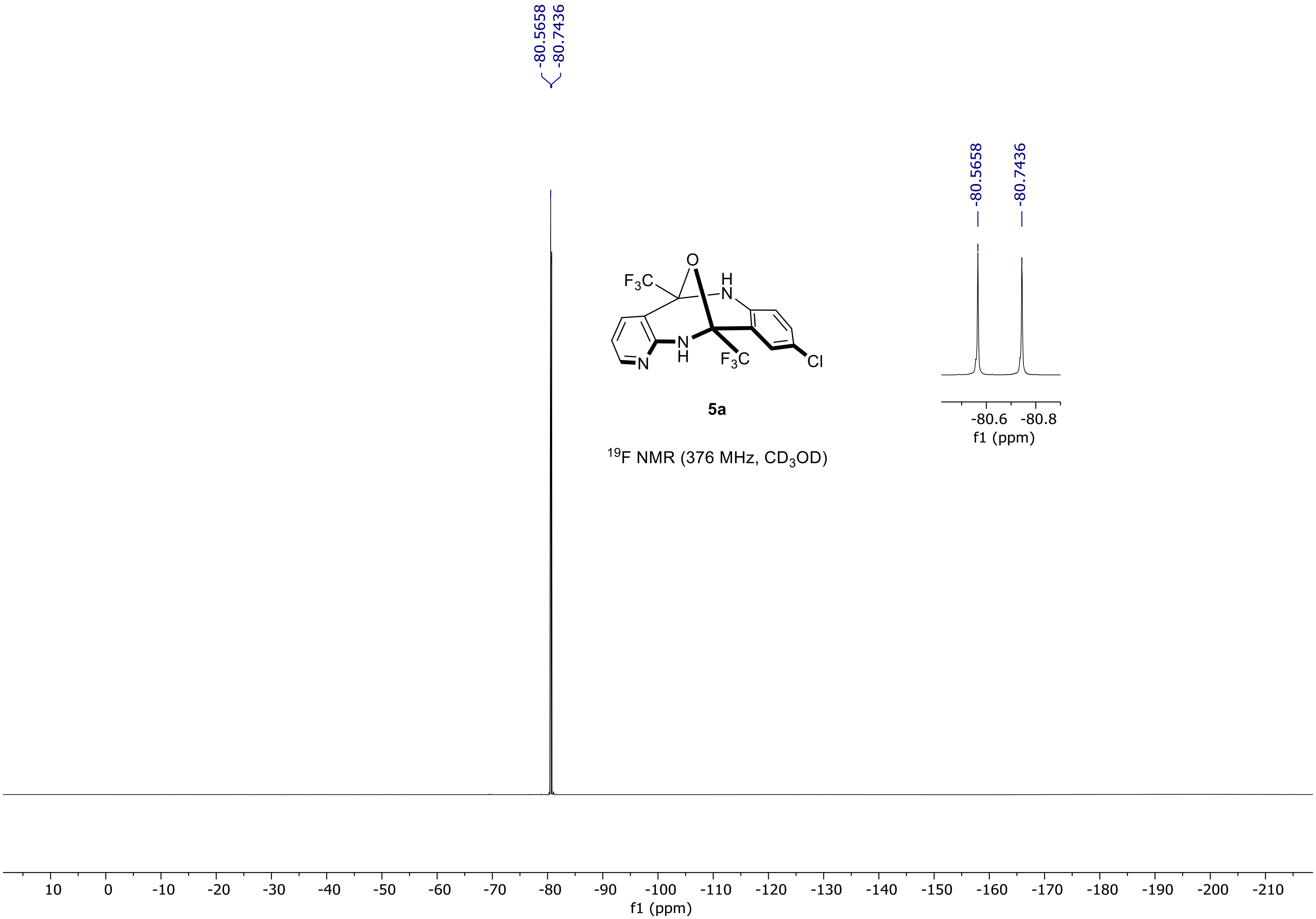
-80.5658
-80.7436
F3C
O
H
N
N
H
F3C
Cl
N
5a
19F NMR (376 MHz, CD3OD)
-80.6 -80.8
f1 (ppm)
10 0 -10 -20 -30 -40 -50 -60 -70 -80 -90 -100 -110 -120 -130 -140 -150 -160 -170 -180 -190 -200 -210
f1 (ppm)

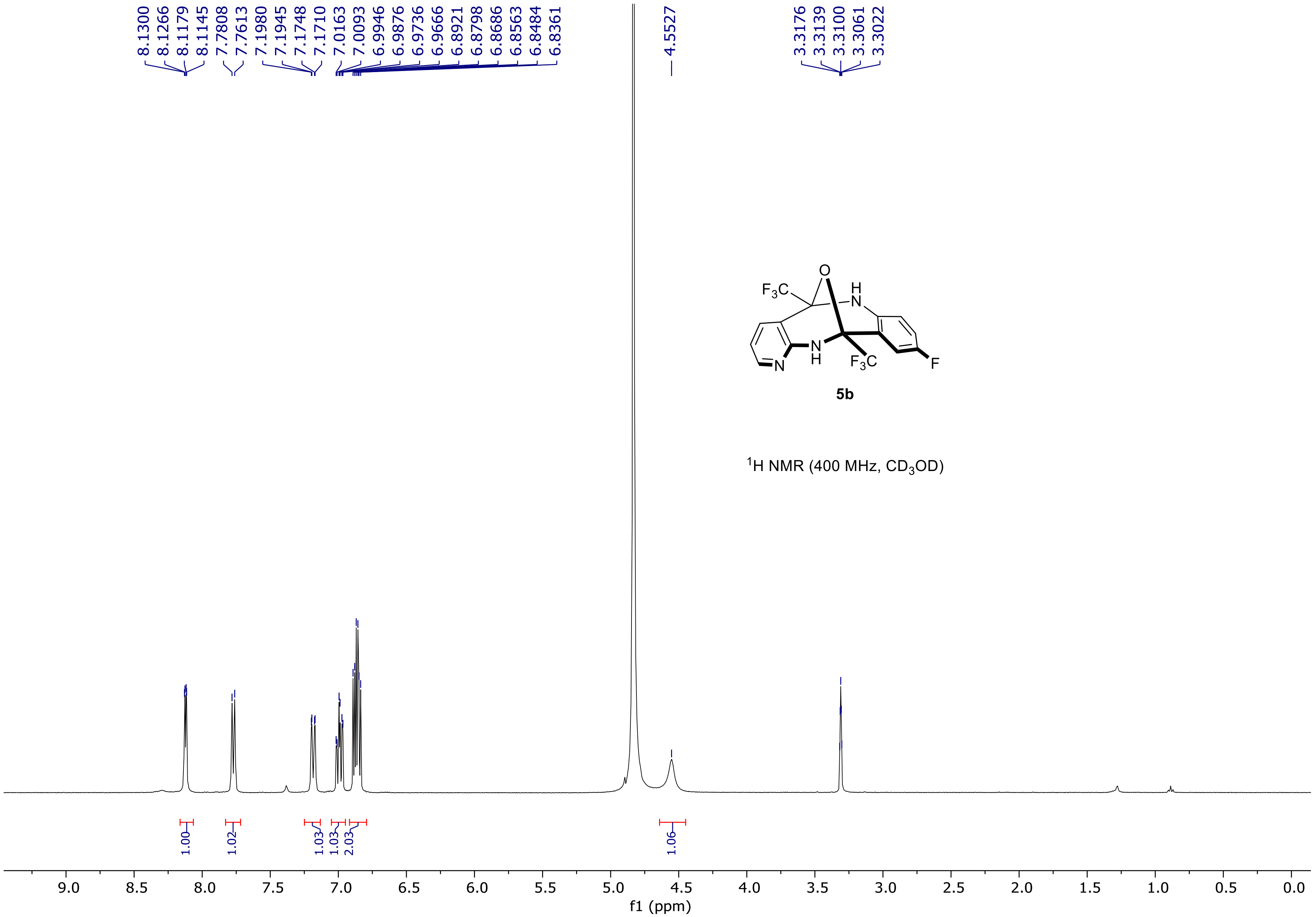
8.1300
8.1266
8.1179
8.1145
7.7808
7.7613
7.1980
7.1945
7.1748
7.1710
7.0163
7.0093
6.9946
6.9876
6.9736
6.9666
6.8921
6.8798
6.8686
6.8563
6.8484
6.8361
4.5527
3.3176
3.3139
3.3100
3.3061
3.3022
F3C
O
H
N
N
H
F3C
F
N
5b
1H NMR (400 MHz, CD3OD)
1.00
1.02
1.03
1.03
2.03
1.06
9.0 8.5 8.0 7.5 7.0 6.5 6.0 5.5 5.0 4.5 4.0 3.5 3.0 2.5 2.0 1.5 1.0 0.5 0.0
f1 (ppm)

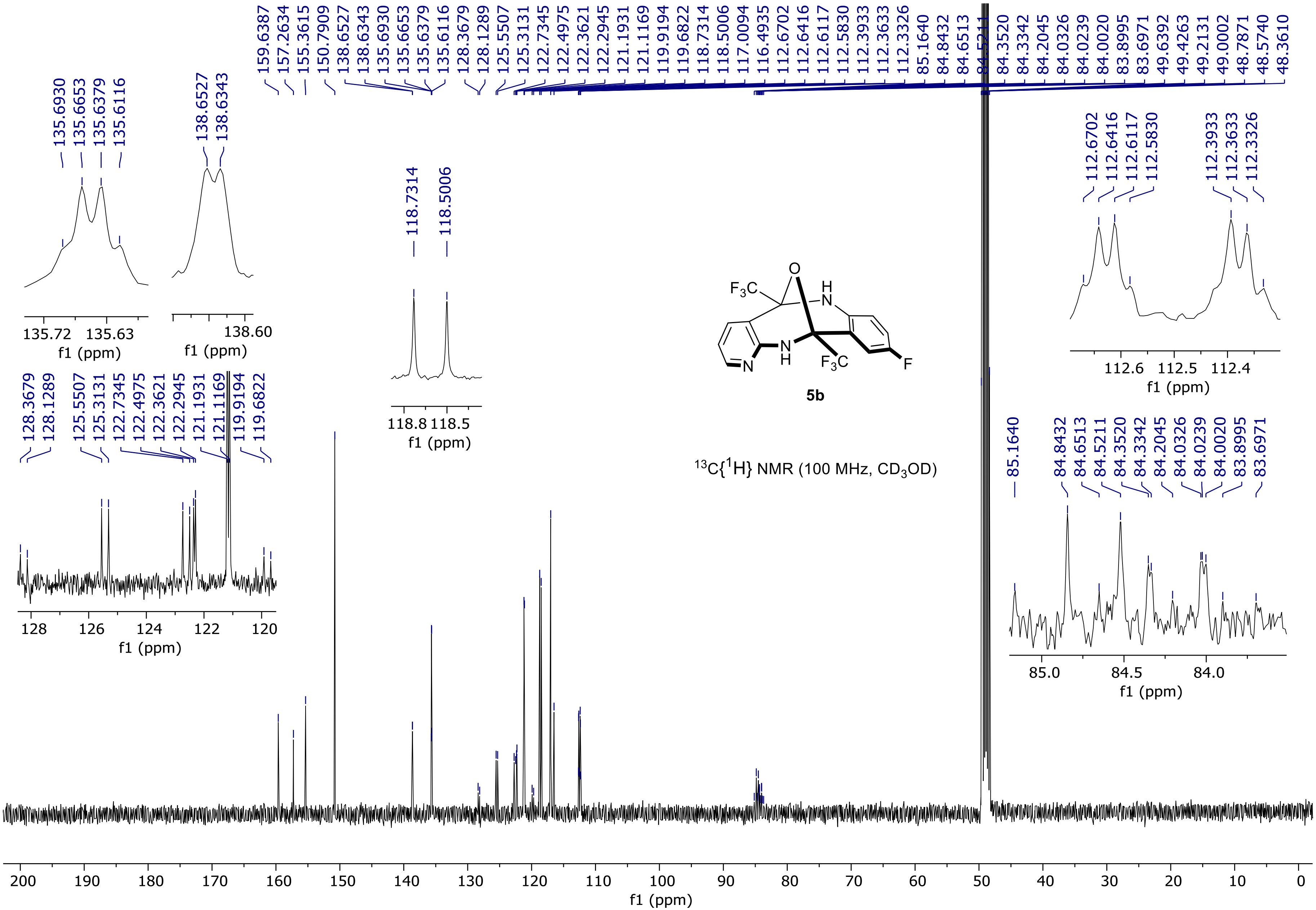
159.6387
157.2634
155.3615
150.7909
138.6527
138.6343
135.6930
135.6653
135.6379
135.6116
128.3679
128.1289
125.5507
125.3131
122.7345
122.4975
122.3621
122.2945
121.1931
121.1169
119.9194
119.6822
118.7314
118.5006
117.0094
116.4935
112.6702
112.6416
112.6117
112.5830
112.3933
112.3633
112.3326
85.1640
84.8432
84.6513
84.5211
84.3520
84.3342
84.2045
84.0326
84.0239
84.0020
83.8995
83.6971
49.6392
49.4263
49.2131
49.0002
48.7871
48.5740
48.3610
f1 (ppm)
5b
$^{13}C\{^1H\}$ NMR (100 MHz, $CD_3OD$)

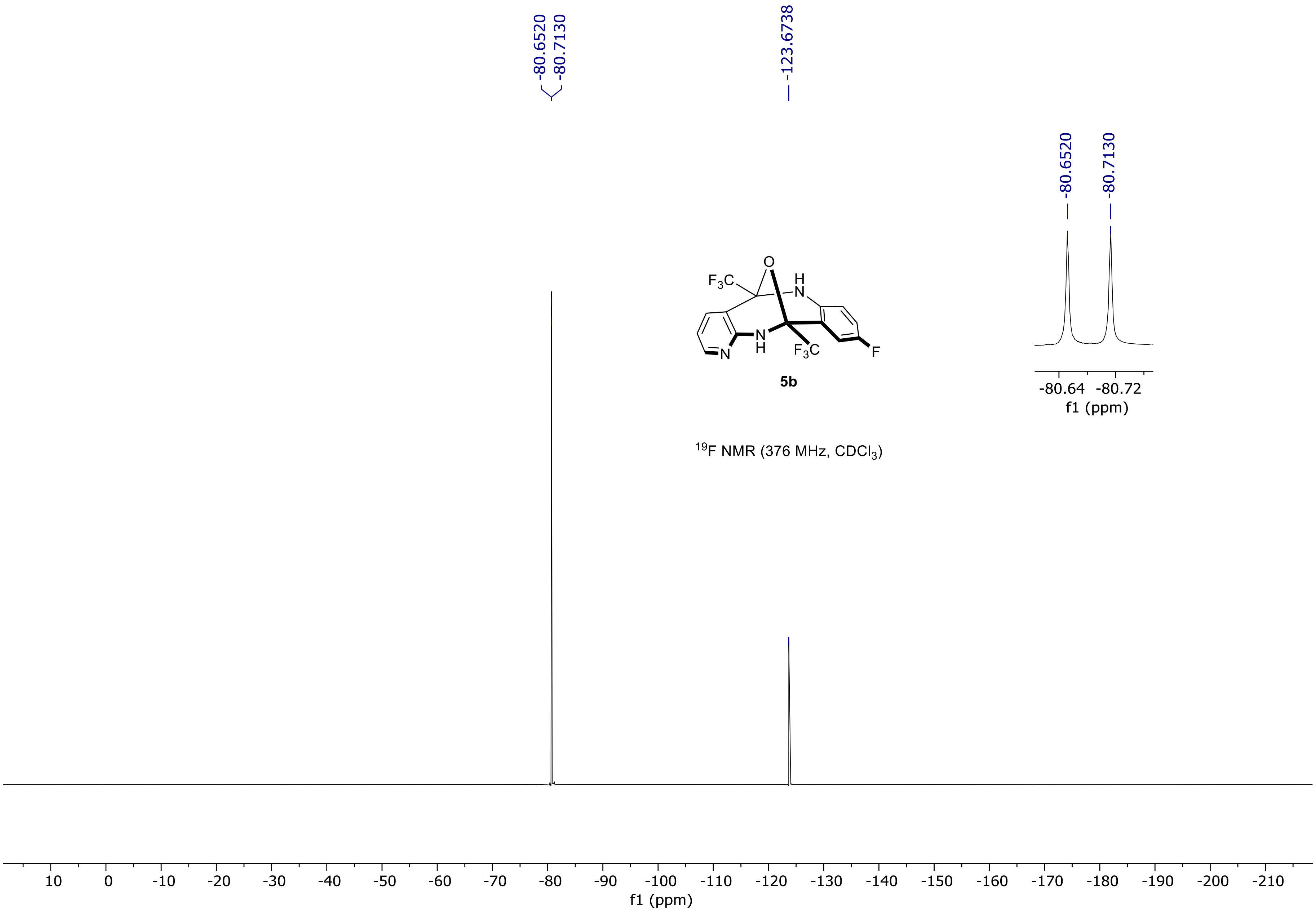
-80.6520
-80.7130
-123.6738
F3C
O
H
N
N
H
F3C
N
F
5b
19F NMR (376 MHz, CDCl3)
-80.64 -80.72
f1 (ppm)
10 0 -10 -20 -30 -40 -50 -60 -70 -80 -90 -100 -110 -120 -130 -140 -150 -160 -170 -180 -190 -200 -210
f1 (ppm)

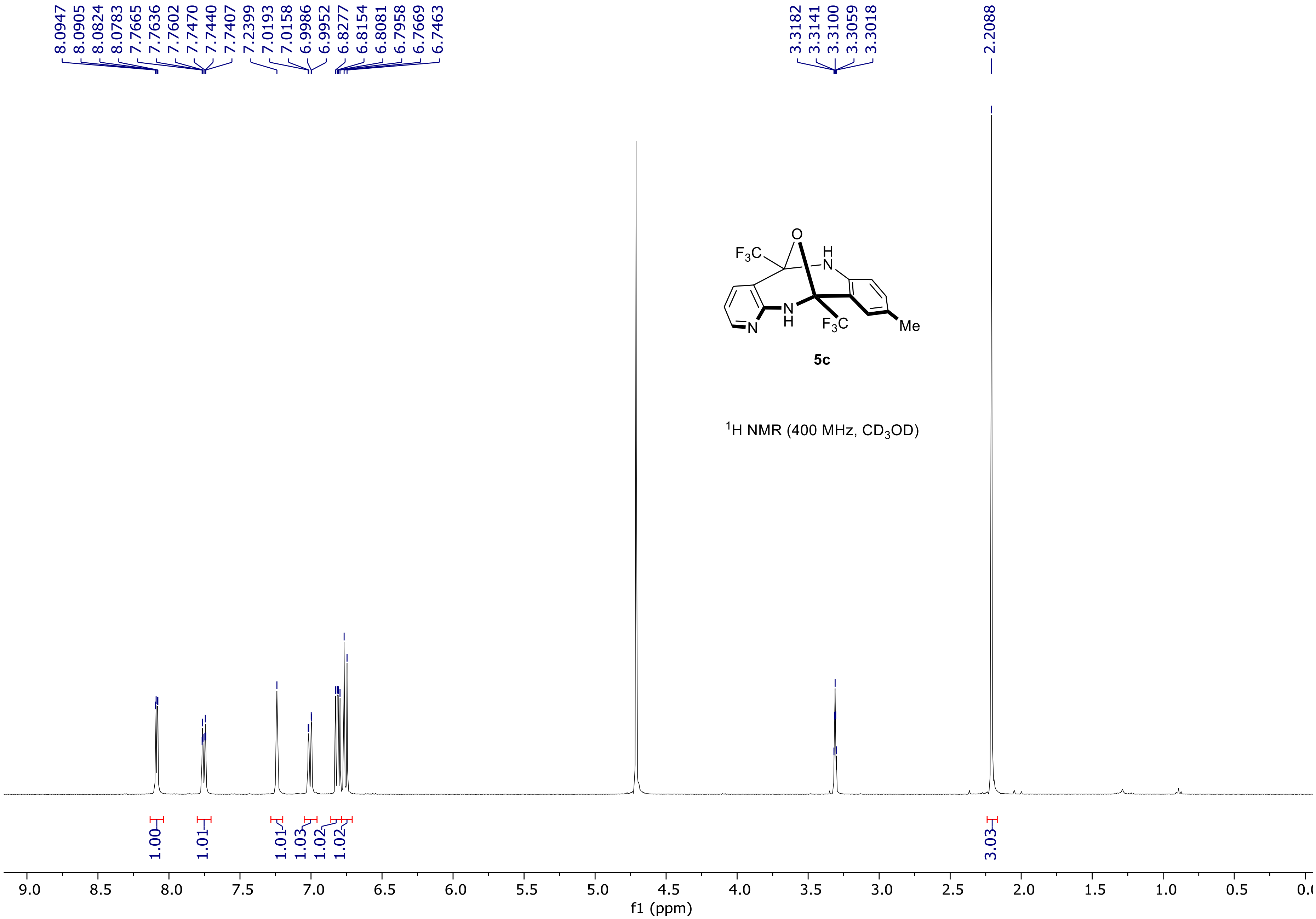
8.0947
8.0905
8.0824
8.0783
7.7665
7.7636
7.7602
7.7470
7.7440
7.7407
7.2399
7.0193
7.0158
6.9986
6.9952
6.8277
6.8154
6.8081
6.7958
6.7669
6.7463
3.3182
3.3141
3.3100
3.3059
3.3018
2.2088
F3C
O
H
N
N
N
H
F3C
Me
5c
1H NMR (400 MHz, CD3OD)
1.00
1.01
1.01
1.03
1.02
1.02
3.03
f1 (ppm)

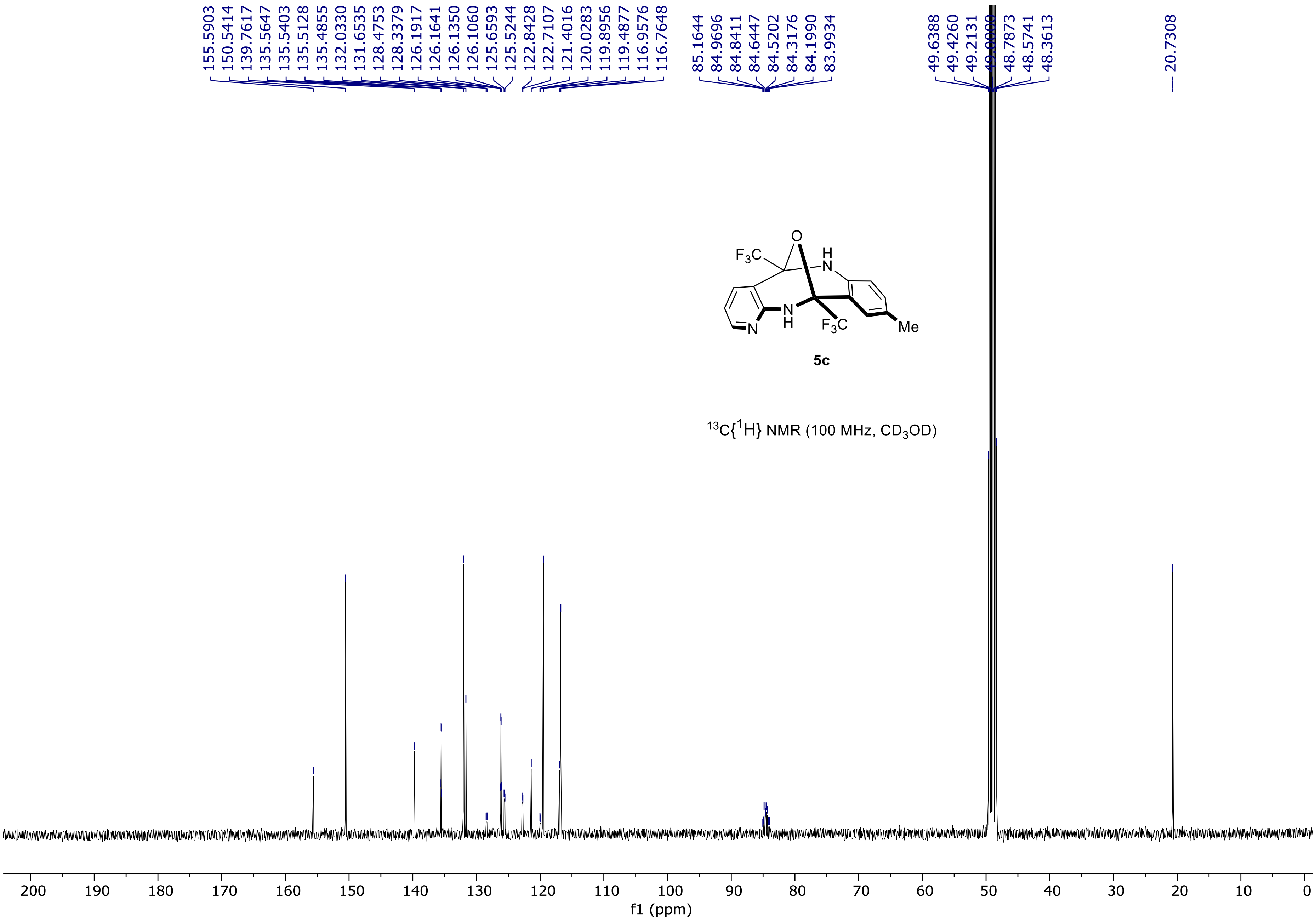
155.5903
150.5414
139.7617
135.5647
135.5403
135.5128
135.4855
132.0330
131.6535
128.4753
128.3379
126.1917
126.1641
126.1350
126.1060
125.6593
125.5244
122.8428
122.7107
121.4016
120.0283
119.8956
119.4877
116.9576
116.7648
85.1644
84.9696
84.8411
84.6447
84.5202
84.3176
84.1990
83.9934
49.6388
49.4260
49.2131
49.0000
48.7873
48.5741
48.3613
20.7308
O
$F_3C$
H
N
N
H
$F_3C$
N
Me
5c
$^{13}C\{^1H\}$ NMR (100 MHz, $CD_3OD$)
200 190 180 170 160 150 140 130 120 110 100 90 80 70 60 50 40 30 20 10 0
f1 (ppm)

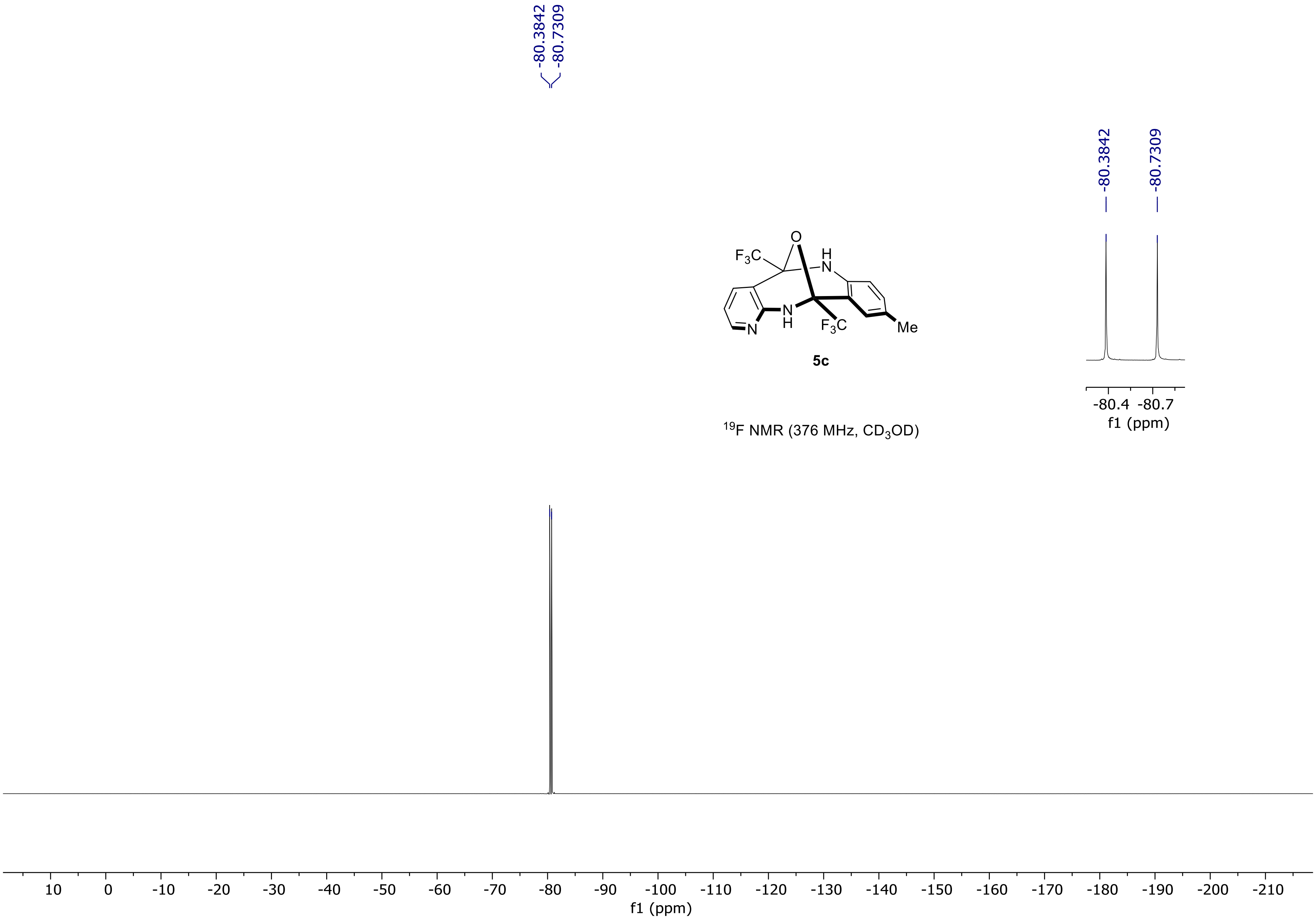
-80.3842
-80.7309
5c
19F NMR (376 MHz, CD3OD)
-80.4 -80.7
f1 (ppm)
10 0 -10 -20 -30 -40 -50 -60 -70 -80 -90 -100 -110 -120 -130 -140 -150 -160 -170 -180 -190 -200 -210
f1 (ppm)

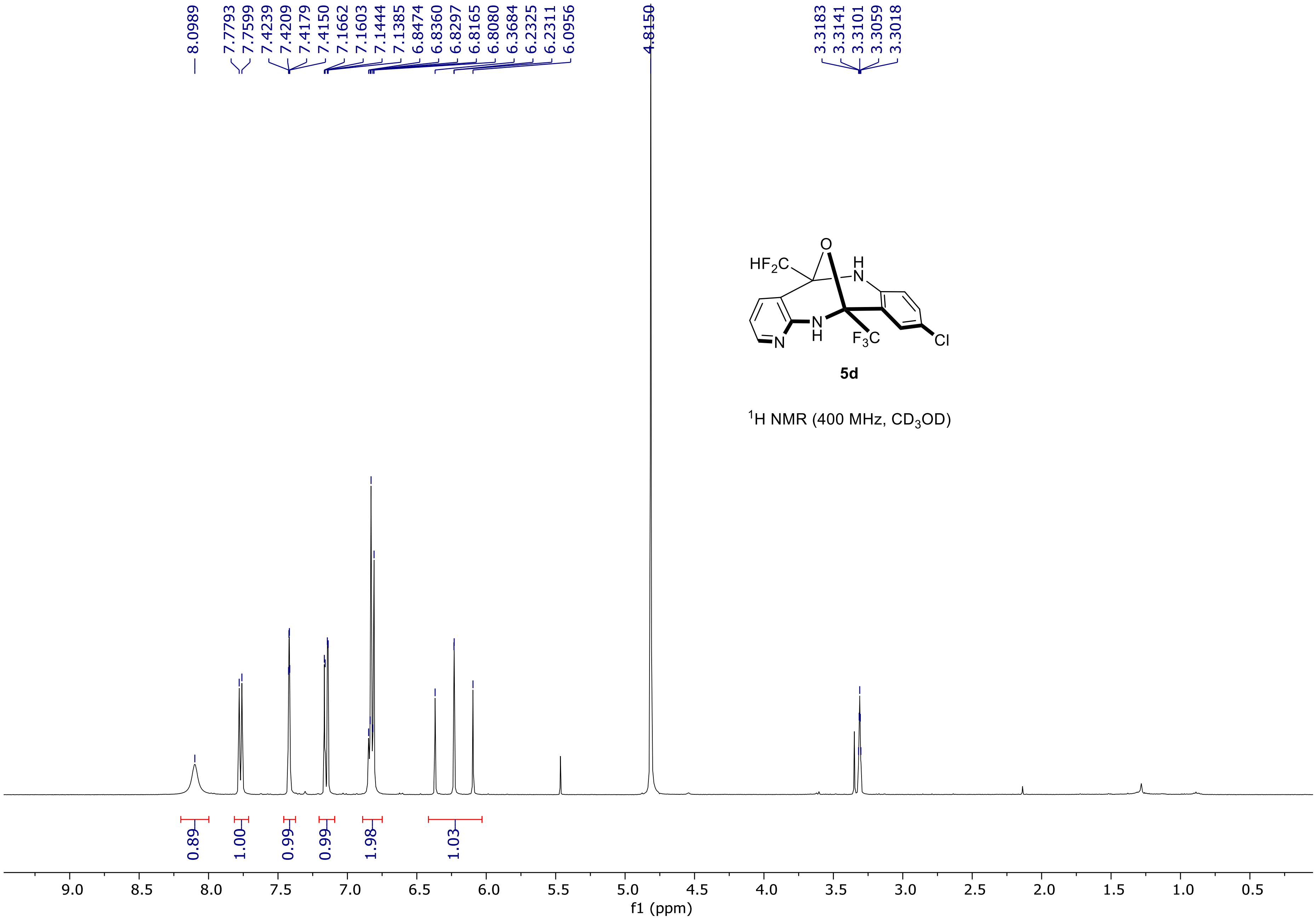


**5d**

$^{1}$H NMR (400 MHz, $CD_3OD$)

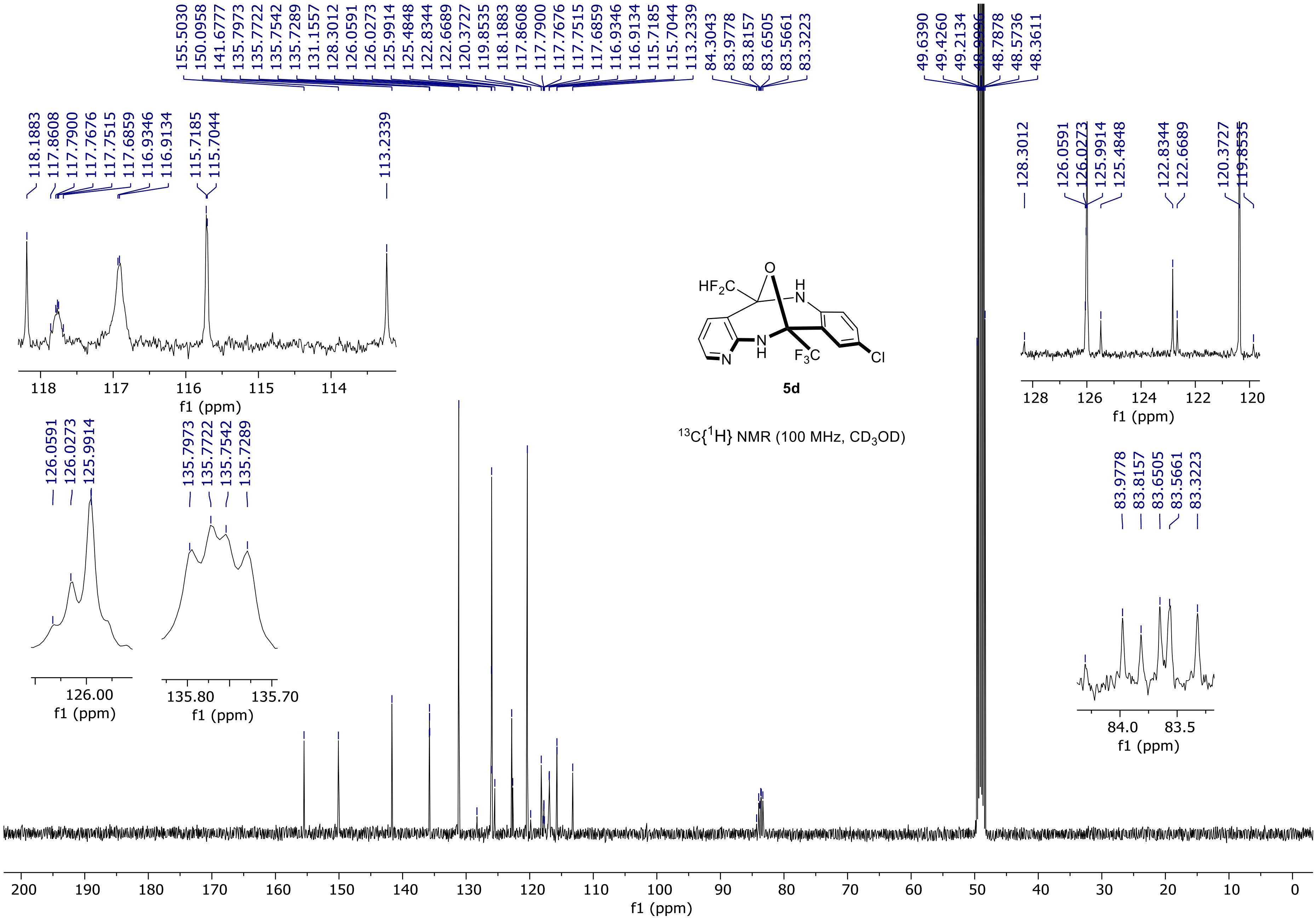
155.5030
150.0958
141.6777
135.7973
135.7722
135.7542
135.7289
131.1557
128.3012
126.0591
126.0273
125.9914
125.4848
122.8344
122.6689
120.3727
119.8535
118.1883
117.8608
117.7900
117.7676
117.7515
117.6859
116.9346
116.9134
115.7185
115.7044
113.2339
84.3043
83.9778
83.8157
83.6505
83.5661
83.3223
49.6390
49.4260
49.2134
48.7878
48.5736
48.3611
HF2C
O
H
N
N
H
N
F3C
Cl
5d
13C{1H} NMR (100 MHz, CD3OD)
f1 (ppm)

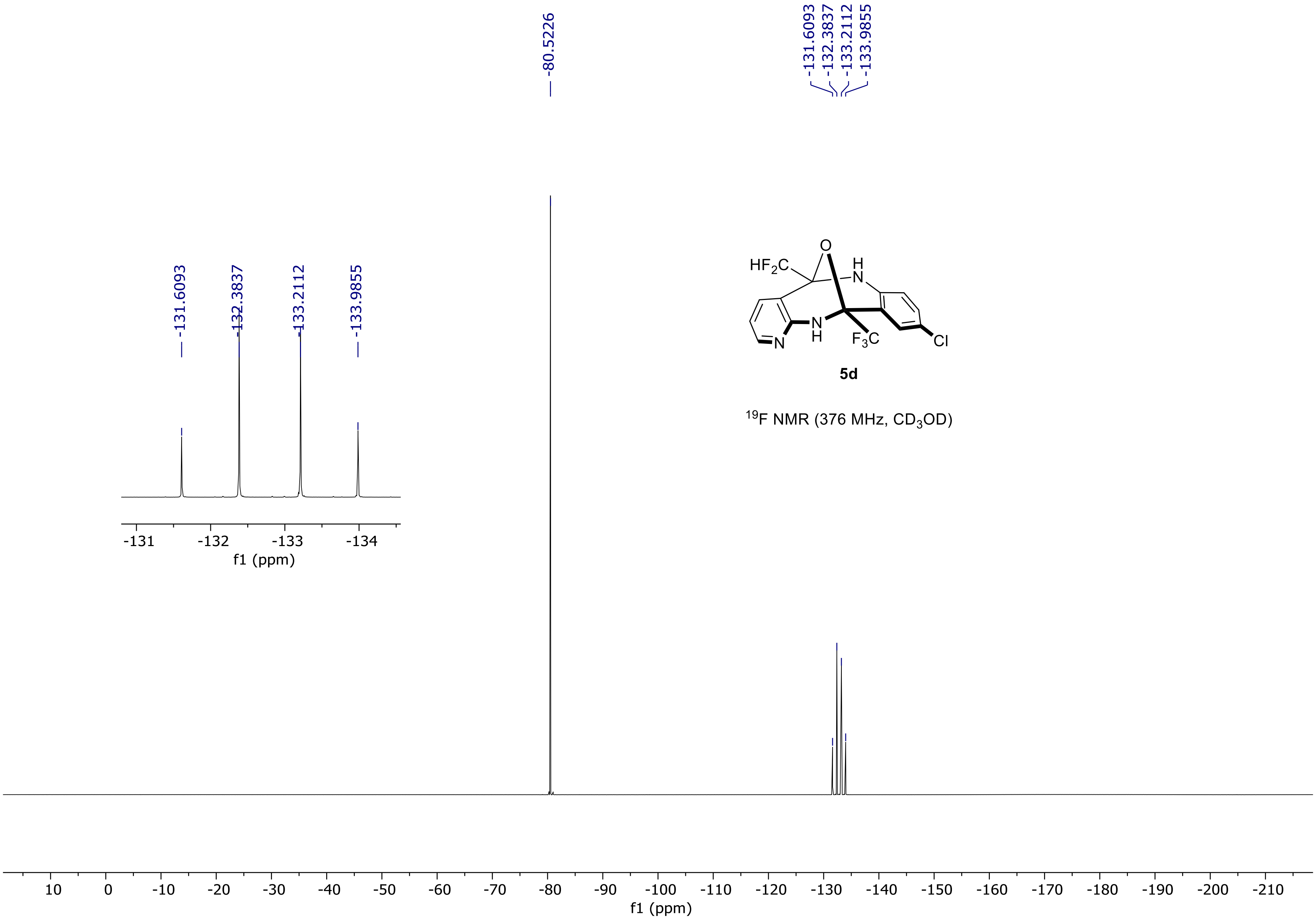
-80.5226
-131.6093
-132.3837
-133.2112
-133.9855
HF2C
O
H
N
N
H
N
F3C
Cl
5d
19F NMR (376 MHz, CD3OD)
-131
-132
-133
-134
f1 (ppm)
10 0 -10 -20 -30 -40 -50 -60 -70 -80 -90 -100 -110 -120 -130 -140 -150 -160 -170 -180 -190 -200 -210
f1 (ppm)

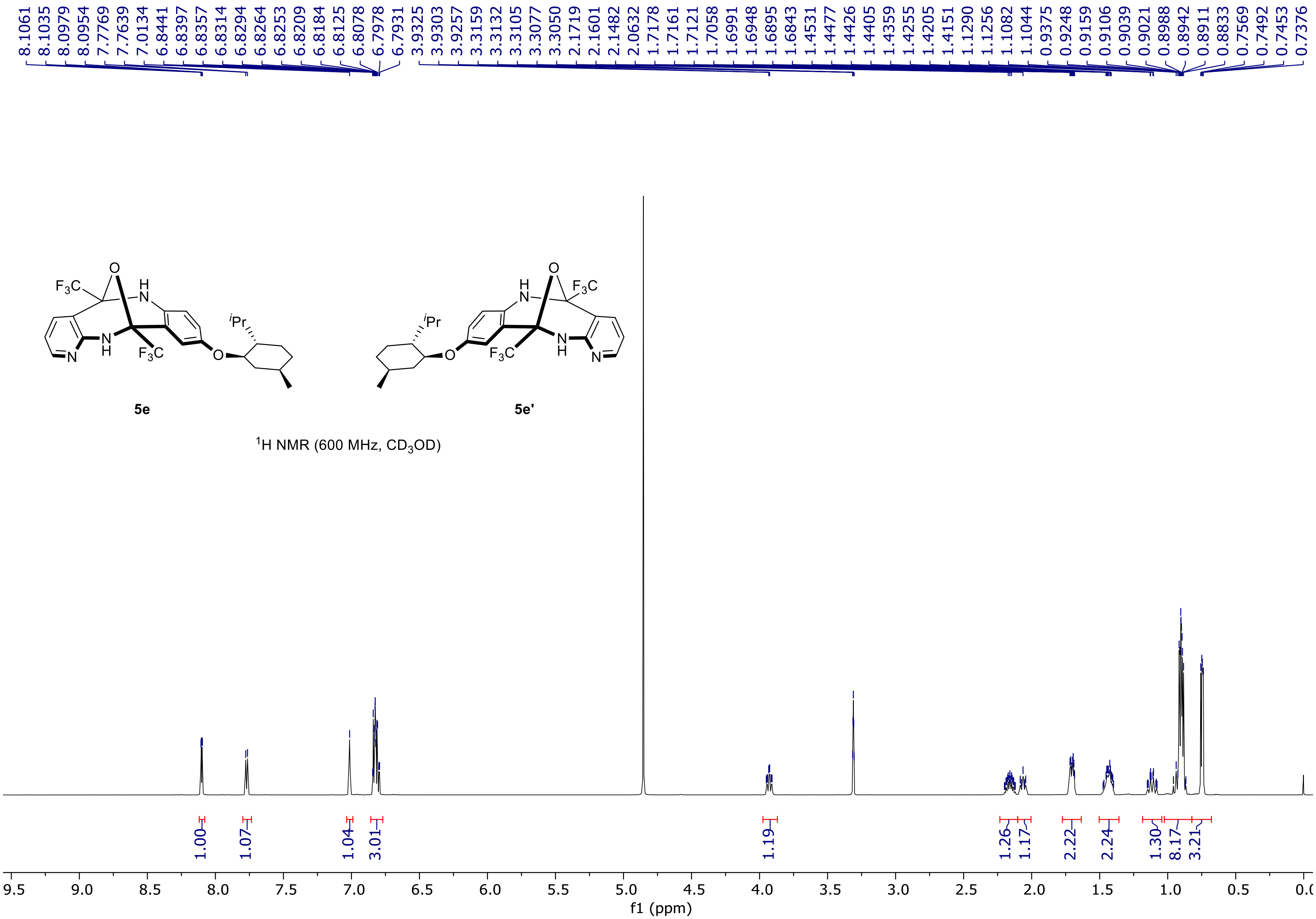
8.1061
8.1035
8.0979
8.0954
7.7769
7.7639
7.0134
6.8441
6.8397
6.8357
6.8314
6.8294
6.8264
6.8253
6.8209
6.8184
6.8125
6.8078
6.7978
6.7931
3.9325
3.9303
3.9257
3.3159
3.3132
3.3105
3.3077
3.3050
2.1719
2.1601
2.1482
2.0632
1.7178
1.7161
1.7121
1.7058
1.6991
1.6948
1.6895
1.6843
1.4531
1.4477
1.4426
1.4405
1.4359
1.4255
1.4205
1.4151
1.1290
1.1256
1.1082
1.1044
0.9375
0.9248
0.9159
0.9106
0.9039
0.9021
0.8988
0.8942
0.8911
0.8833
0.7569
0.7492
0.7453
0.7376
5e
5e'
$^1$H NMR (600 MHz, $CD_3OD$)
1.00
1.07
1.04
3.01
1.19
1.26
1.17
2.22
2.24
1.30
8.17
3.21
f1 (ppm)

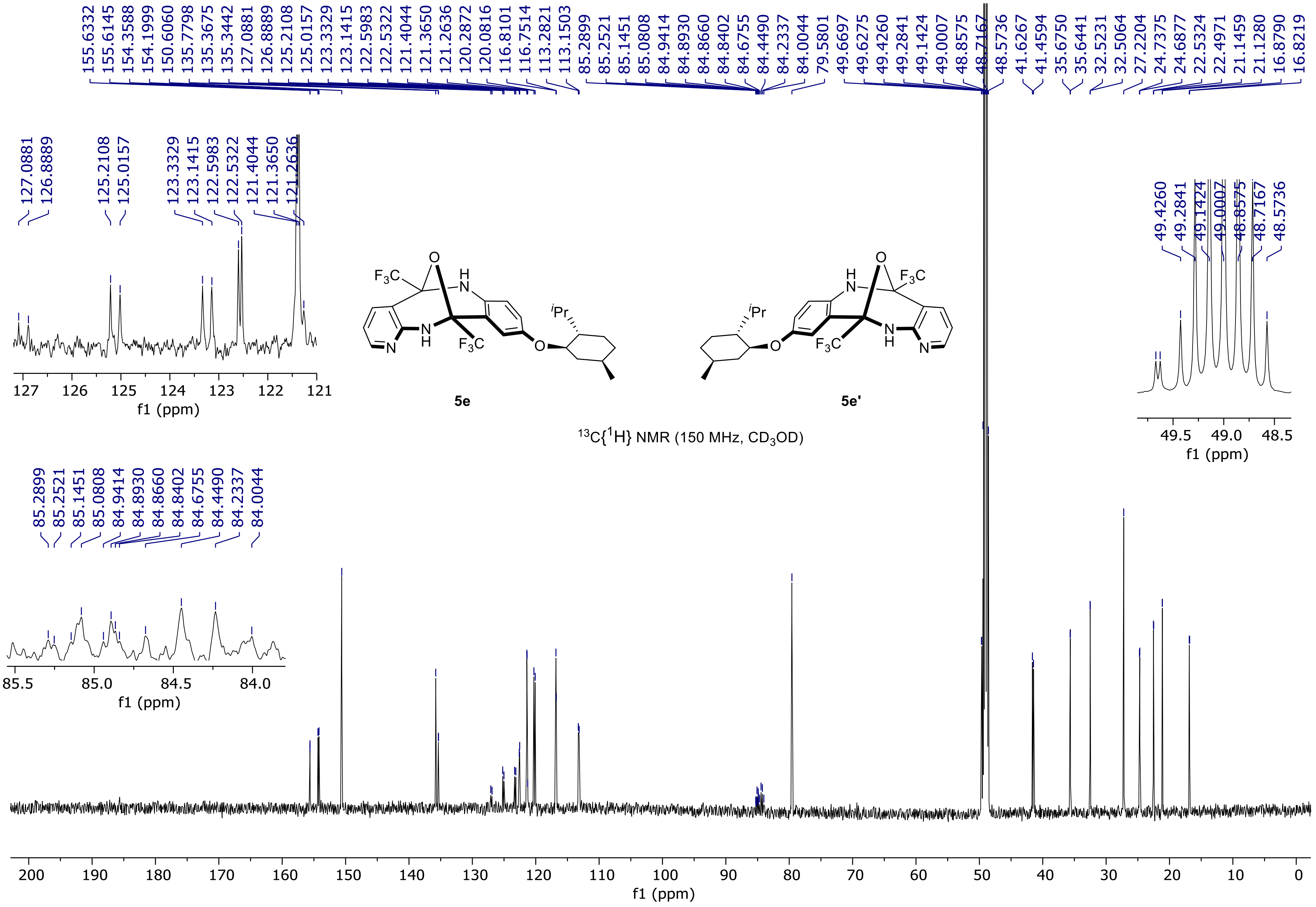
155.6332 155.6145 154.3588 154.1999 150.6060 135.7798 135.3675 135.3442 127.0881 126.8889 125.2108 125.0157 123.3329 123.1415 122.5983 122.5322 121.4044 121.3650 121.2636 120.2872 120.0816 116.8101 116.7514 113.2821 113.1503 85.2899 85.2521 85.1451 85.0808 84.9414 84.8930 84.8660 84.8402 84.6755 84.4490 84.2337 84.0044 79.5801 49.6697 49.6275 49.4260 49.2841 49.1424 49.0007 48.8575 48.7167 48.5736 41.6267 41.4594 35.6750 35.6441 32.5231 32.5064 27.2204 24.7375 24.6877 22.5324 22.4971 21.1459 21.1280 16.8790 16.8219
f1 (ppm)
5e
5e'
$^{13}C\{^{1}H\}$ NMR (150 MHz, $CD_3OD$)

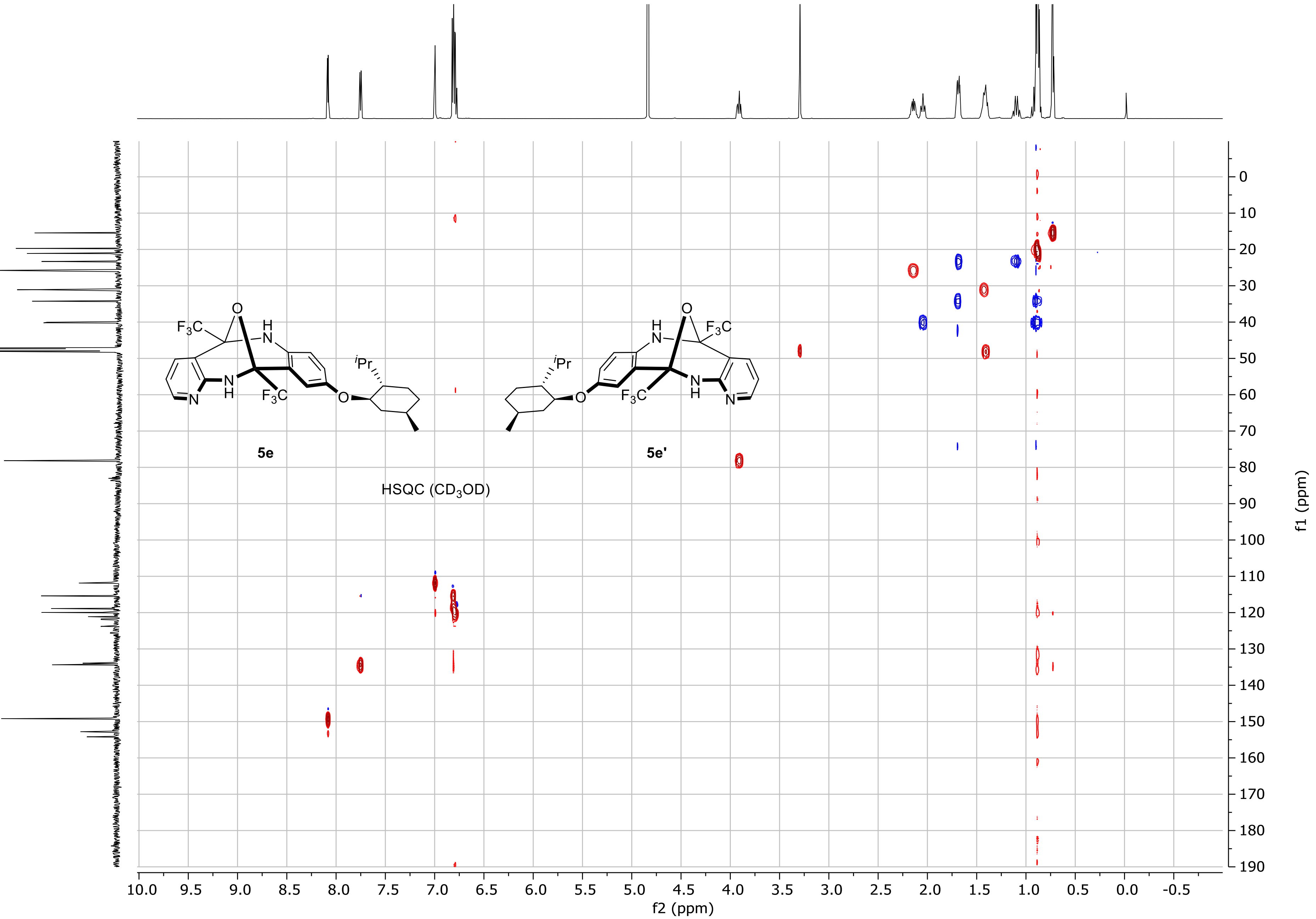
5e
5e'
HSQC ($CD_3OD$)
f2 (ppm)
f1 (ppm)

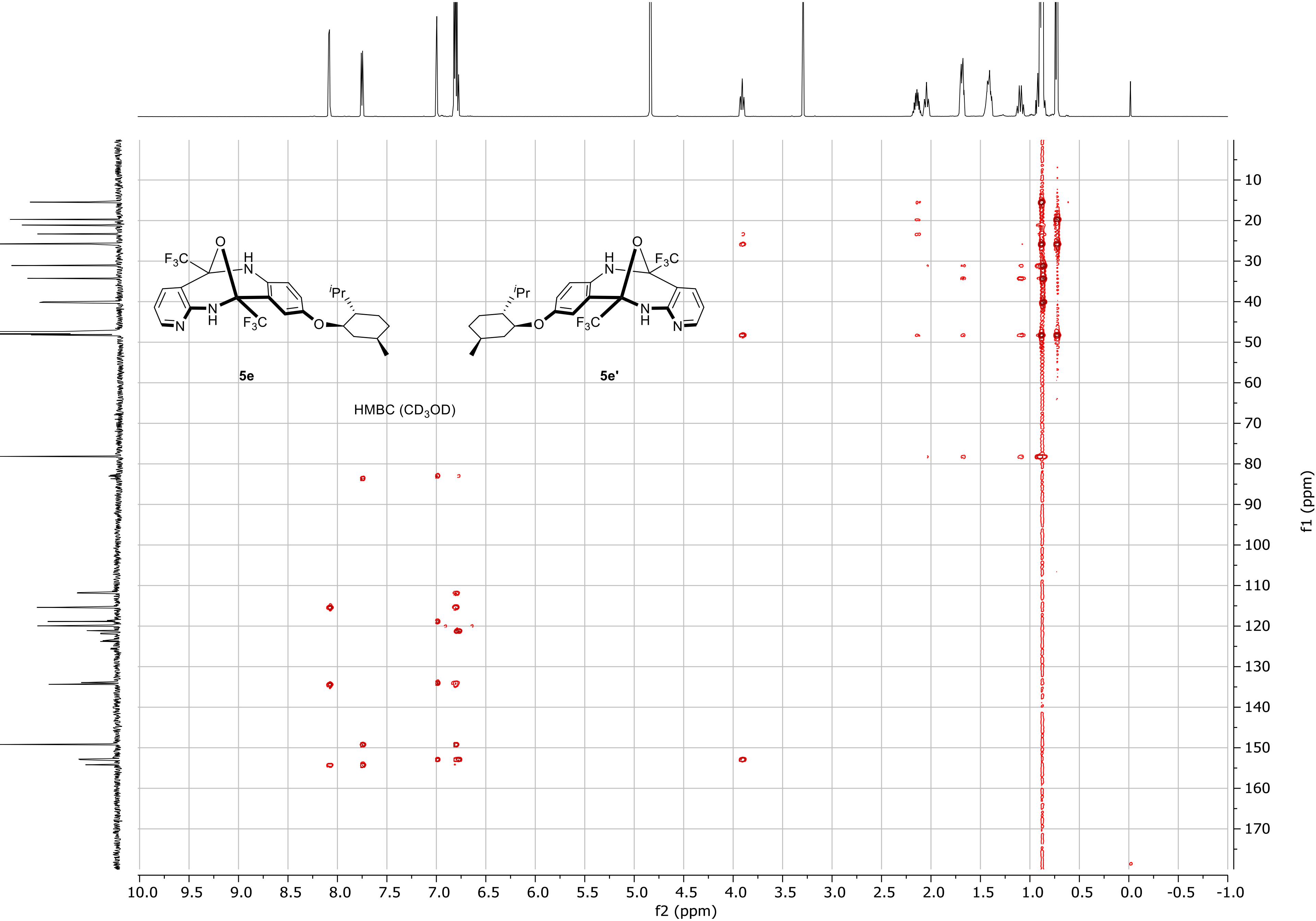
5e
5e'
HMBC (CD$_3$OD)
f2 (ppm)
f1 (ppm)

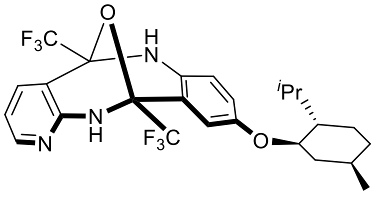


**5e**

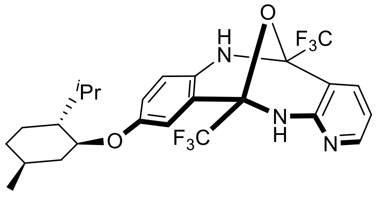


**5e'**

$^{19}$F NMR (376 MHz, $CD_3OD$)

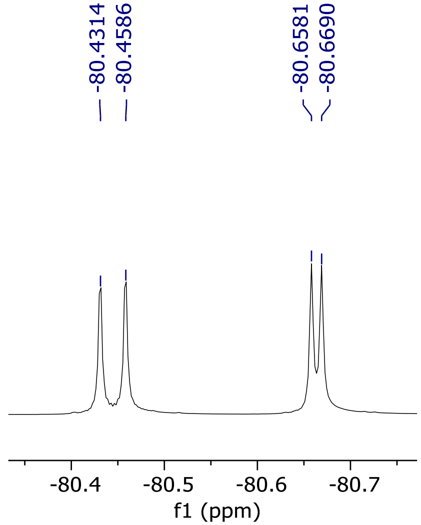
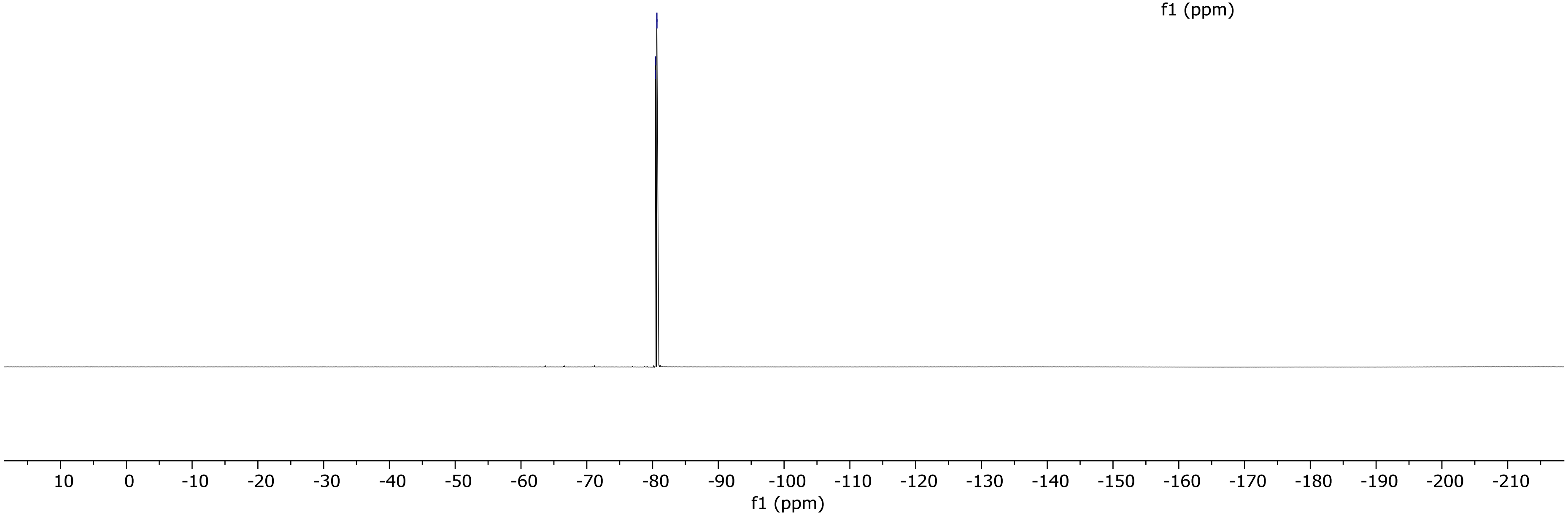

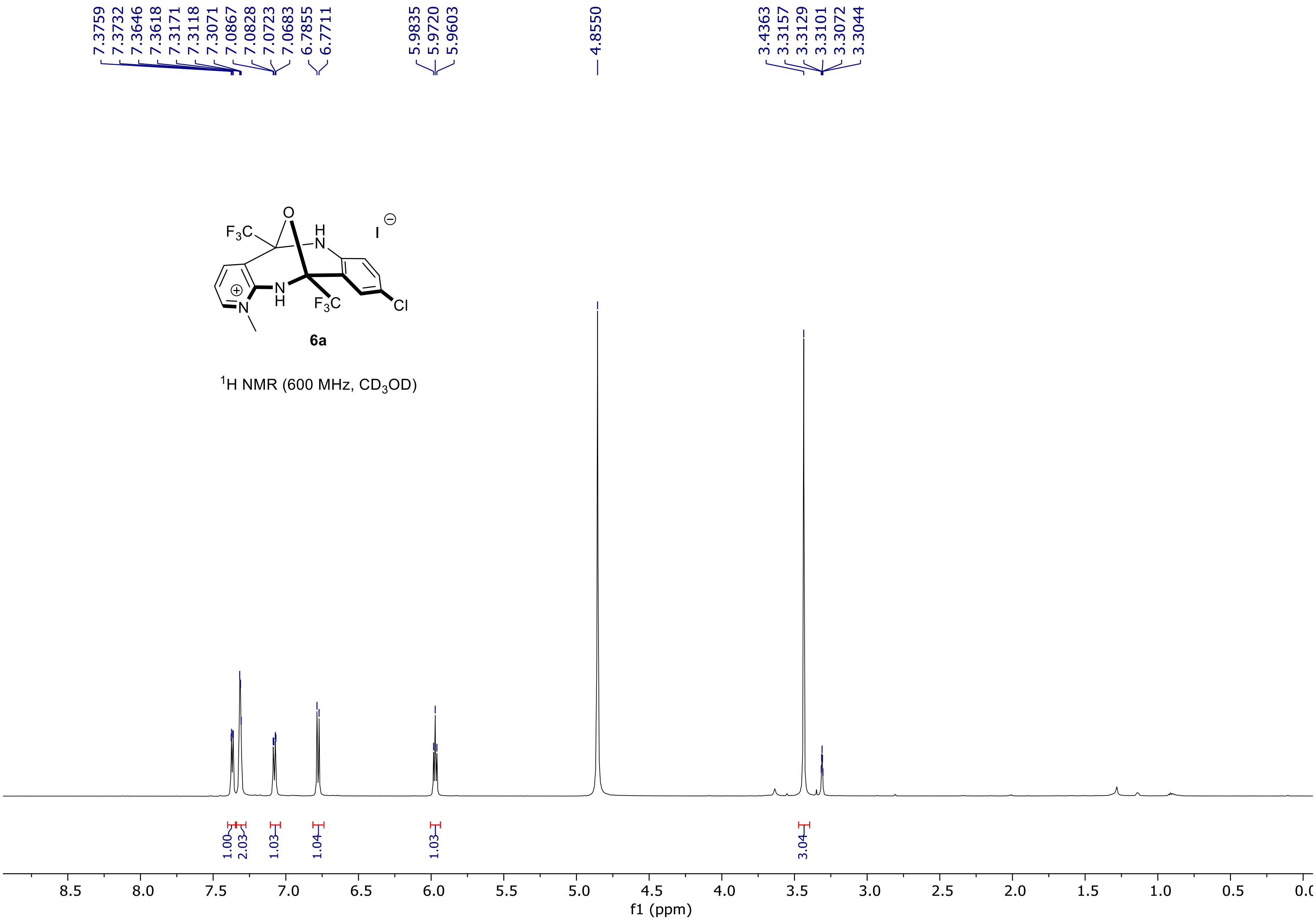
7.3759
7.3732
7.3646
7.3618
7.3171
7.3118
7.3071
7.0867
7.0828
7.0723
7.0683
6.7855
6.7711
5.9835
5.9720
5.9603
4.8550
3.4363
3.3157
3.3129
3.3101
3.3072
3.3044
F3C
O
H
N
I
N
H
N
F3C
Cl
6a
1H NMR (600 MHz, CD3OD)
1.00
2.03
1.03
1.04
1.03
3.04
8.5
8.0
7.5
7.0
6.5
6.0
5.5
5.0
4.5
4.0
3.5
3.0
2.5
2.0
1.5
1.0
0.5
0.0
f1 (ppm)

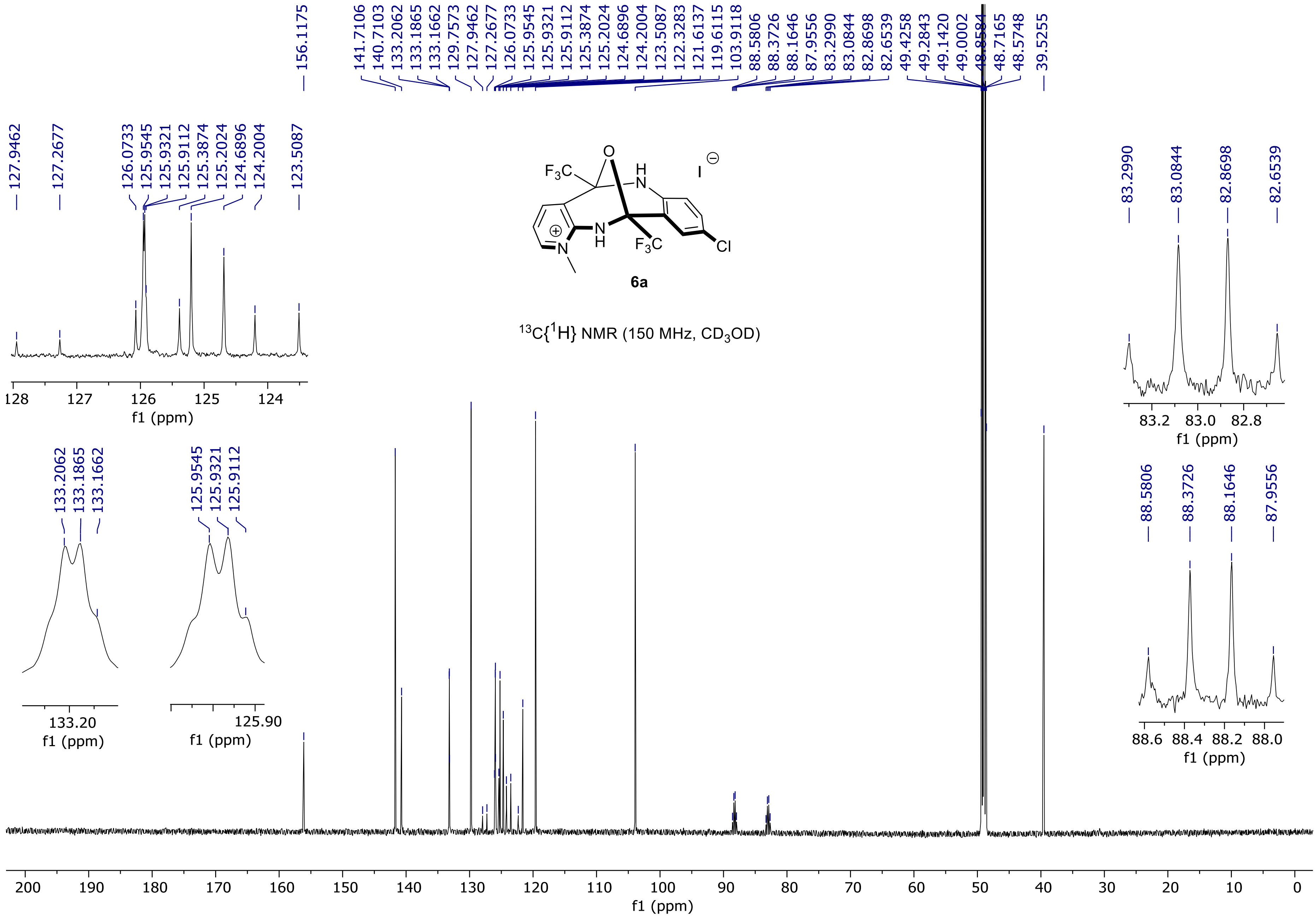
156.1175
141.7106
140.7103
133.2062
133.1865
133.1662
129.7573
127.9462
127.2677
126.0733
125.9545
125.9321
125.9112
125.3874
125.2024
124.6896
124.2004
123.5087
122.3283
121.6137
119.6115
103.9118
88.5806
88.3726
88.1646
87.9556
83.2990
83.0844
82.8698
82.6539
49.4258
49.2843
49.1420
49.0002
48.8584
48.7165
48.5748
39.5255
6a
$^{13}C\{^1H\}$ NMR (150 MHz, $CD_3OD$)
f1 (ppm)

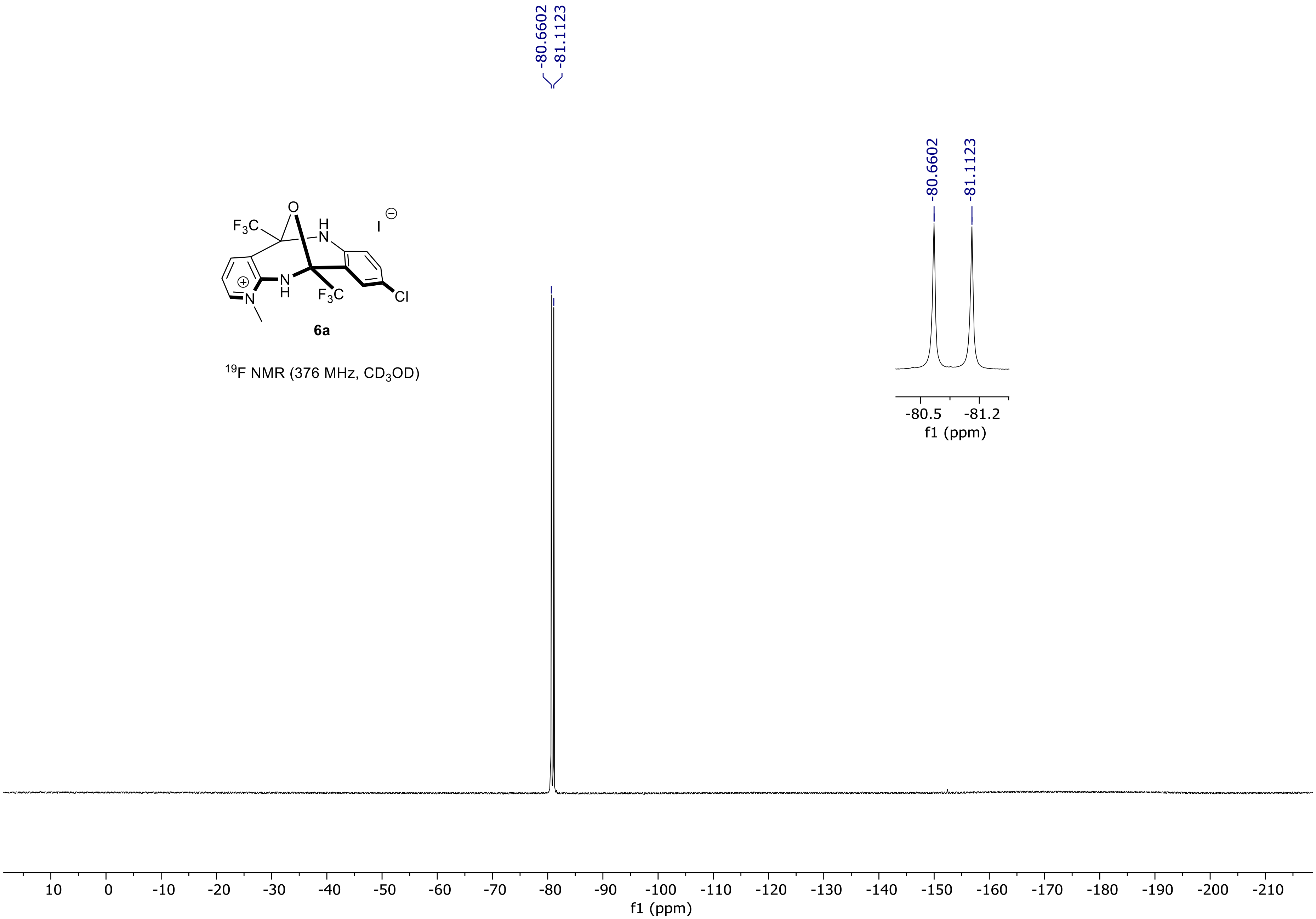


**6a**

$^{19}$F NMR (376 MHz, $CD_3OD$)

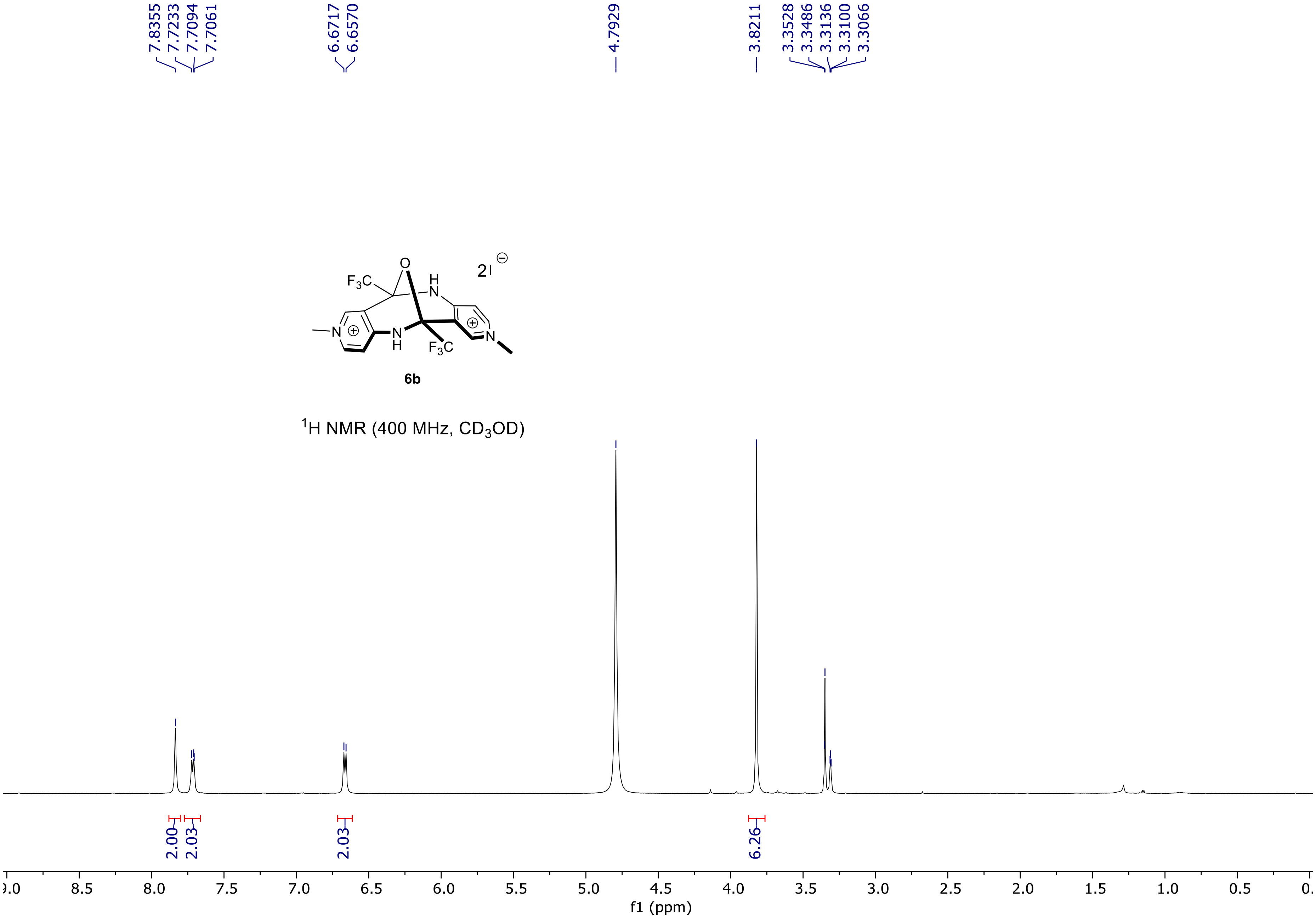
7.8355
7.7233
7.7094
7.7061
6.6717
6.6570
4.7929
3.8211
3.3528
3.3486
3.3136
3.3100
3.3066
F3C
O
H
N
2I
N
N
H
F3C
N
6b
1H NMR (400 MHz, CD3OD)
2.00
2.03
2.03
6.26
9.0
8.5
8.0
7.5
7.0
6.5
6.0
5.5
5.0
4.5
4.0
3.5
3.0
2.5
2.0
1.5
1.0
0.5
0.
f1 (ppm)

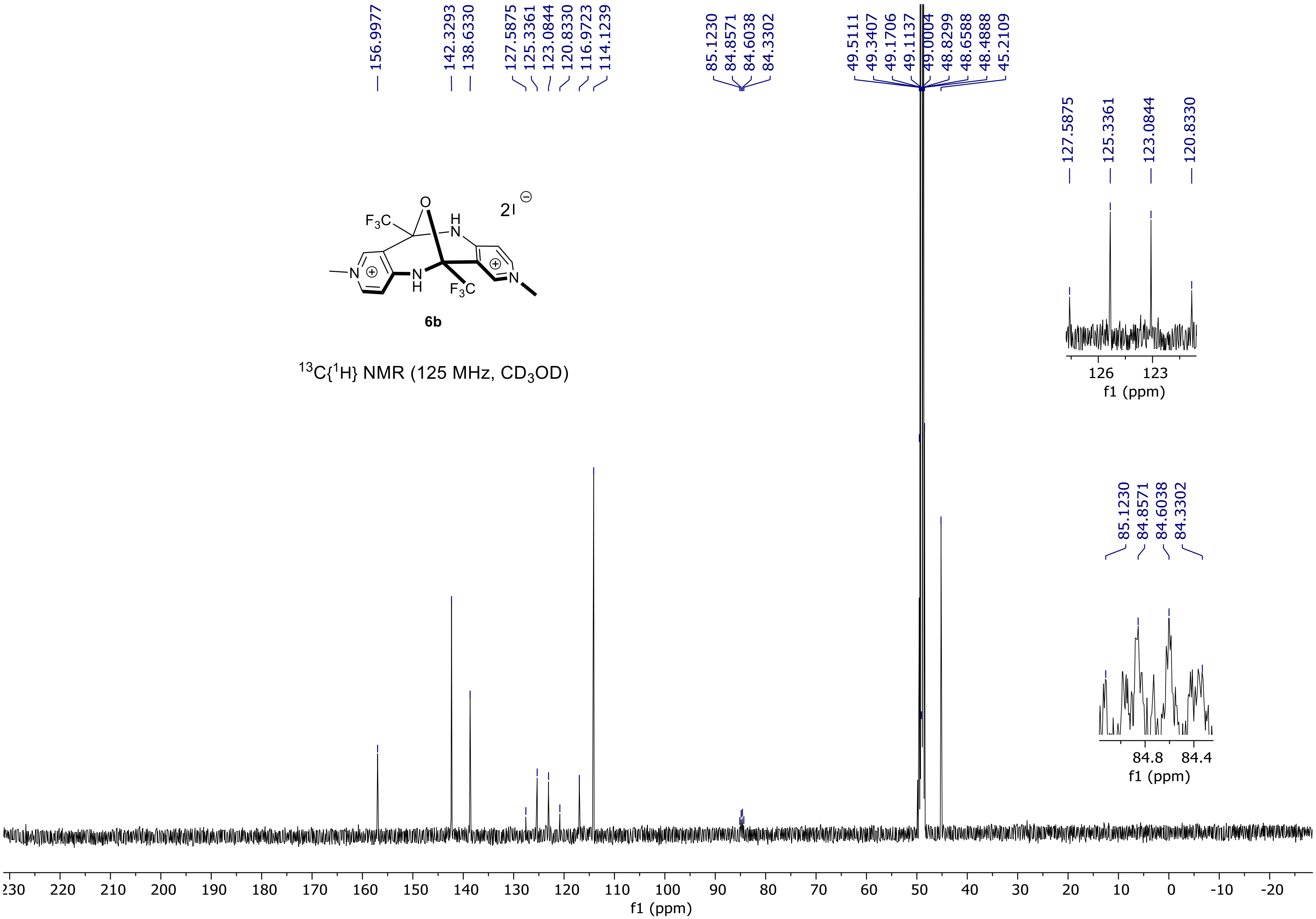
156.9977
142.3293
138.6330
127.5875
125.3361
123.0844
120.8330
116.9723
114.1239
85.1230
84.8571
84.6038
84.3302
49.5111
49.3407
49.1706
49.1137
49.0004
48.8299
48.6588
48.4888
45.2109
F3C
O
H
N
2I
N
N
H
F3C
N
6b
13C{1H} NMR (125 MHz, CD3OD)
126
123
f1 (ppm)
84.8
84.4
f1 (ppm)
230 220 210 200 190 180 170 160 150 140 130 120 110 100 90 80 70 60 50 40 30 20 10 0 -10 -20
f1 (ppm)

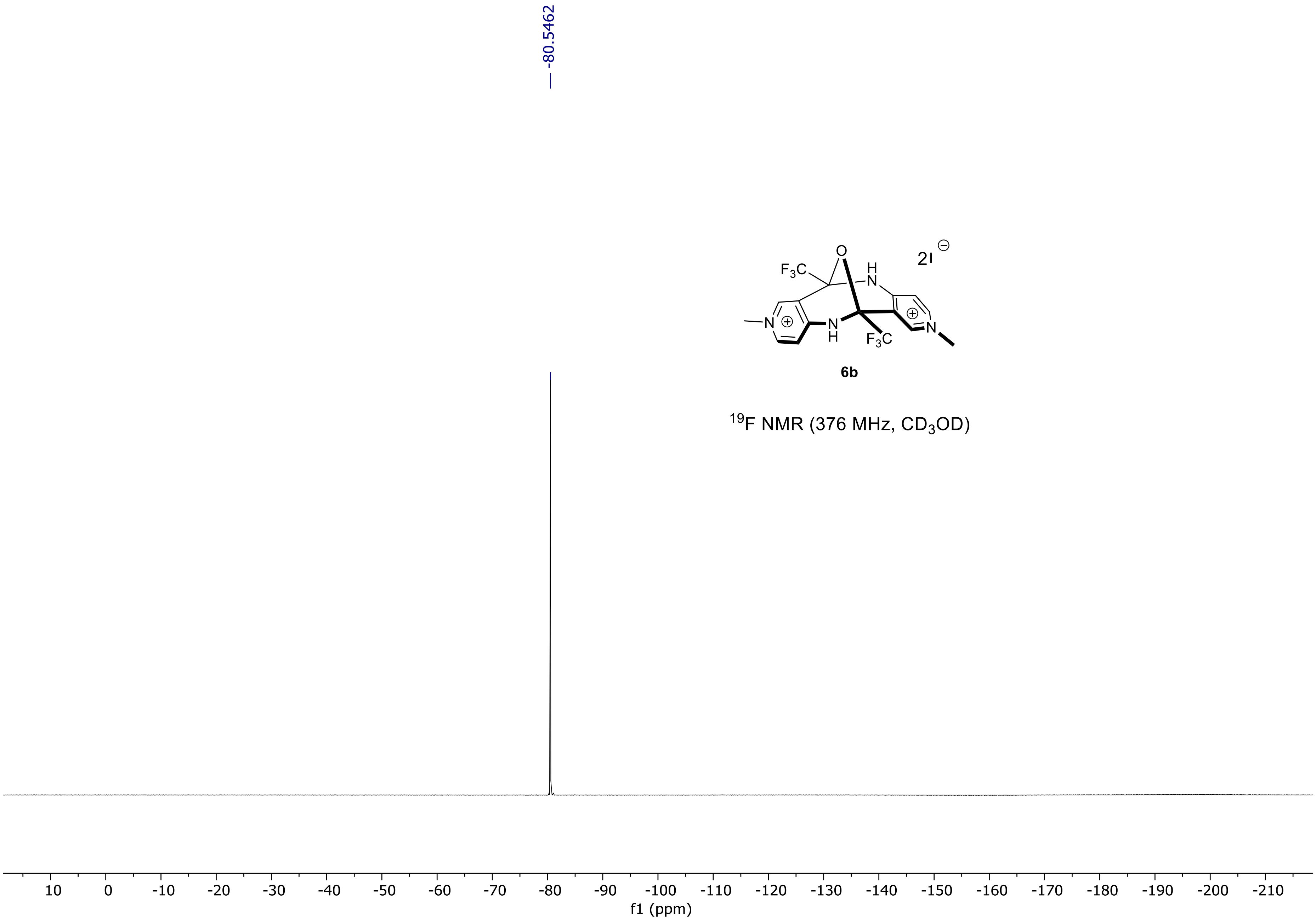
-80.5462
F3C
O
H
N
2I
N
N
H
F3C
N
6b
19F NMR (376 MHz, CD3OD)
10
0
-10
-20
-30
-40
-50
-60
-70
-80
-90
-100
-110
-120
-130
-140
-150
-160
-170
-180
-190
-200
-210
f1 (ppm)

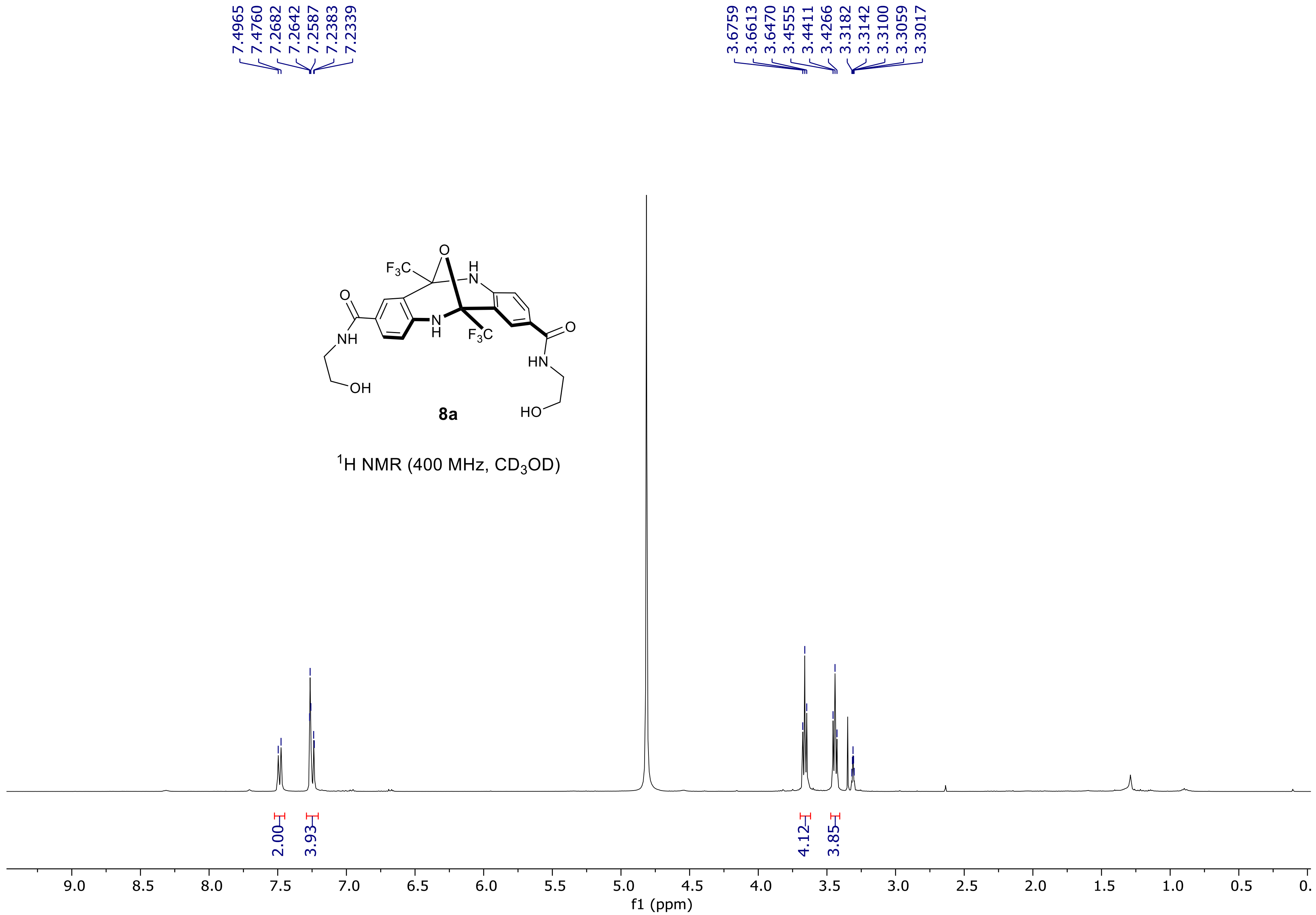
7.4965
7.4760
7.2682
7.2642
7.2587
7.2383
7.2339
3.6759
3.6613
3.6470
3.4555
3.4411
3.4266
3.3182
3.3142
3.3100
3.3059
3.3017
F3C
O
H
N
O
NH
N
H
F3C
O
HN
OH
HO
8a
1H NMR (400 MHz, CD3OD)
2.00
3.93
4.12
3.85
9.0
8.5
8.0
7.5
7.0
6.5
6.0
5.5
5.0
4.5
4.0
3.5
3.0
2.5
2.0
1.5
1.0
0.5
f1 (ppm)

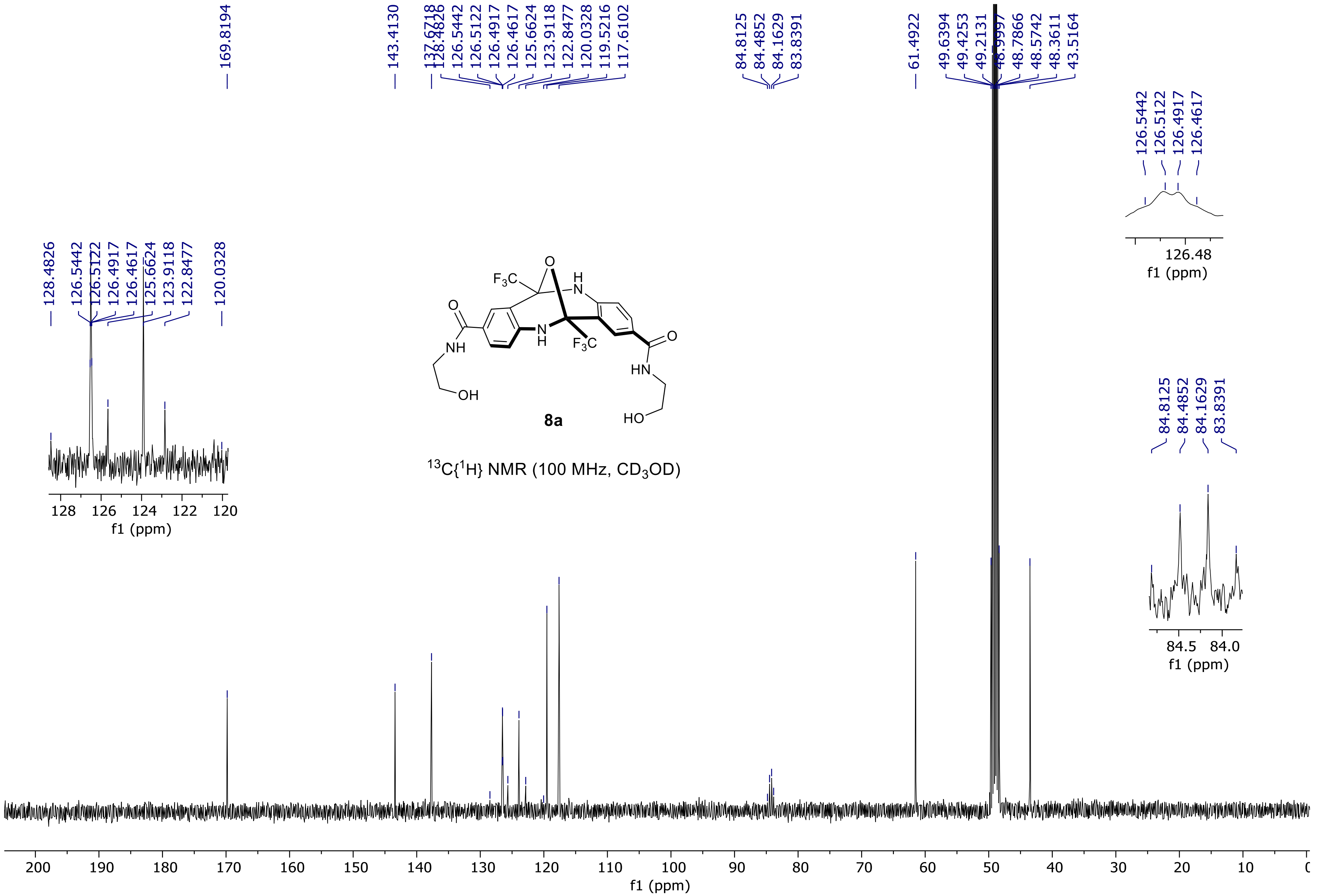


**8a**

$^{13}C\{^{1}H\}$ NMR (100 MHz, $CD_3OD$)

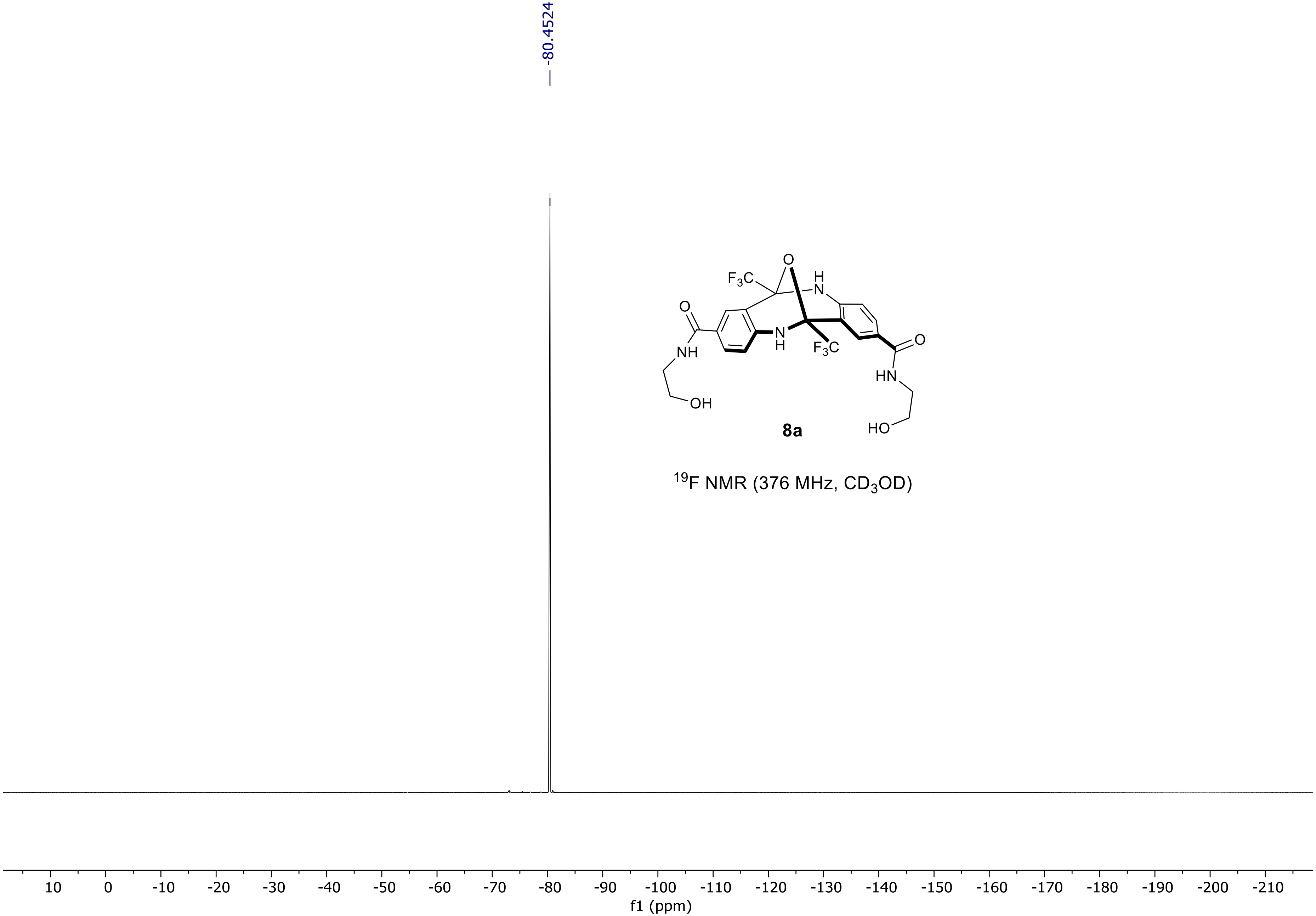
-80.4524
8a
19F NMR (376 MHz, CD3OD)
10 0 -10 -20 -30 -40 -50 -60 -70 -80 -90 -100 -110 -120 -130 -140 -150 -160 -170 -180 -190 -200 -210
f1 (ppm)

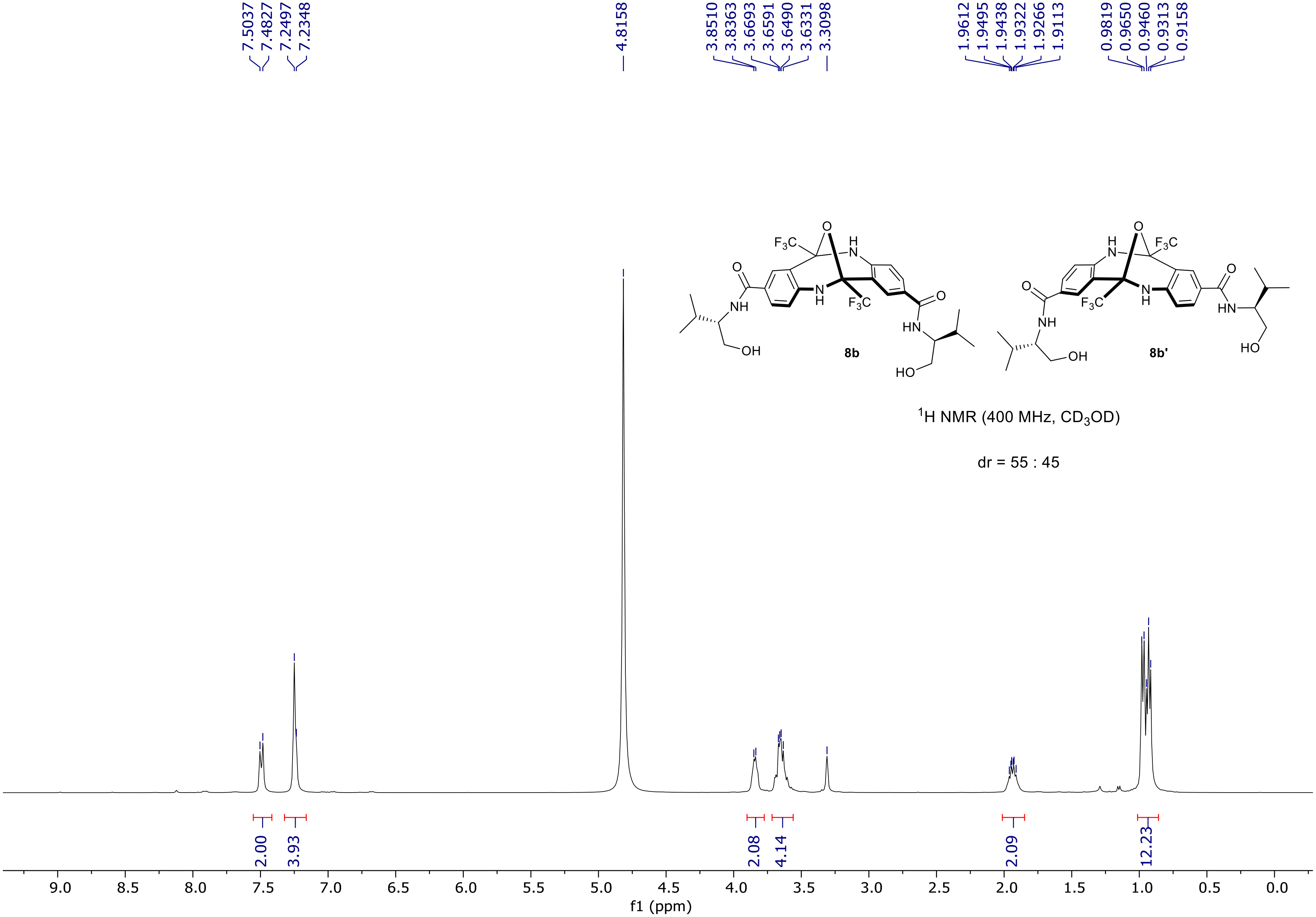
7.5037
7.4827
7.2497
7.2348
4.8158
3.8510
3.8363
3.6693
3.6591
3.6490
3.6331
3.3098
1.9612
1.9495
1.9438
1.9322
1.9266
1.9113
0.9819
0.9650
0.9460
0.9313
0.9158
8b
8b'
1H NMR (400 MHz, CD3OD)
dr = 55 : 45
2.00
3.93
2.08
4.14
2.09
12.23
9.0
8.5
8.0
7.5
7.0
6.5
6.0
5.5
5.0
4.5
4.0
3.5
3.0
2.5
2.0
1.5
1.0
0.5
0.0
f1 (ppm)

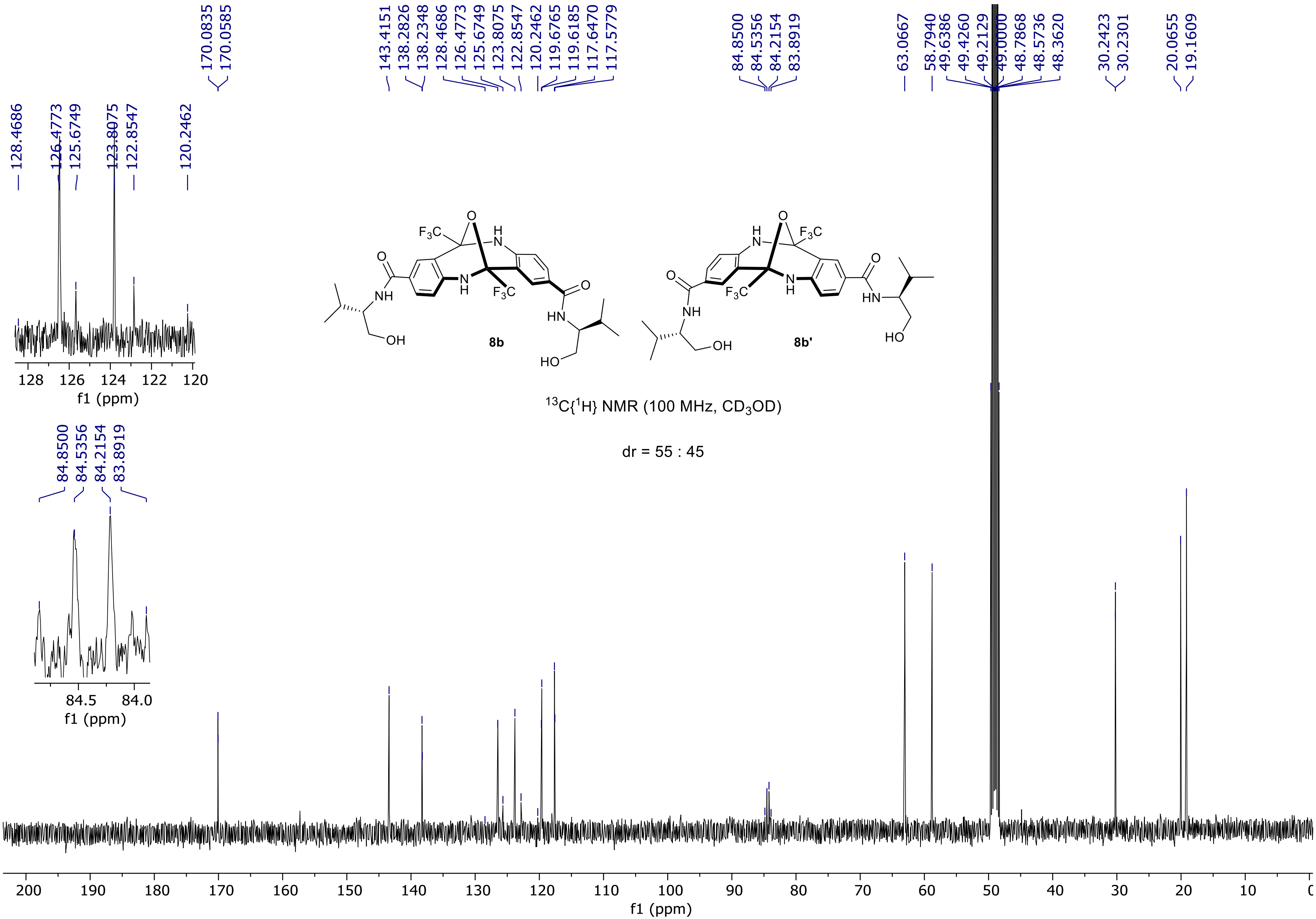
170.0835
170.0585
143.4151
138.2826
138.2348
128.4686
126.4773
125.6749
123.8075
122.8547
120.2462
119.6765
119.6185
117.6470
117.5779
84.8500
84.5356
84.2154
83.8919
63.0667
58.7940
49.6386
49.4260
49.2129
49.0000
48.7868
48.5736
48.3620
30.2423
30.2301
20.0655
19.1609
8b
8b'
$^{13}C\{^1H\}$ NMR (100 MHz, $CD_3OD$)
dr = 55 : 45
f1 (ppm)

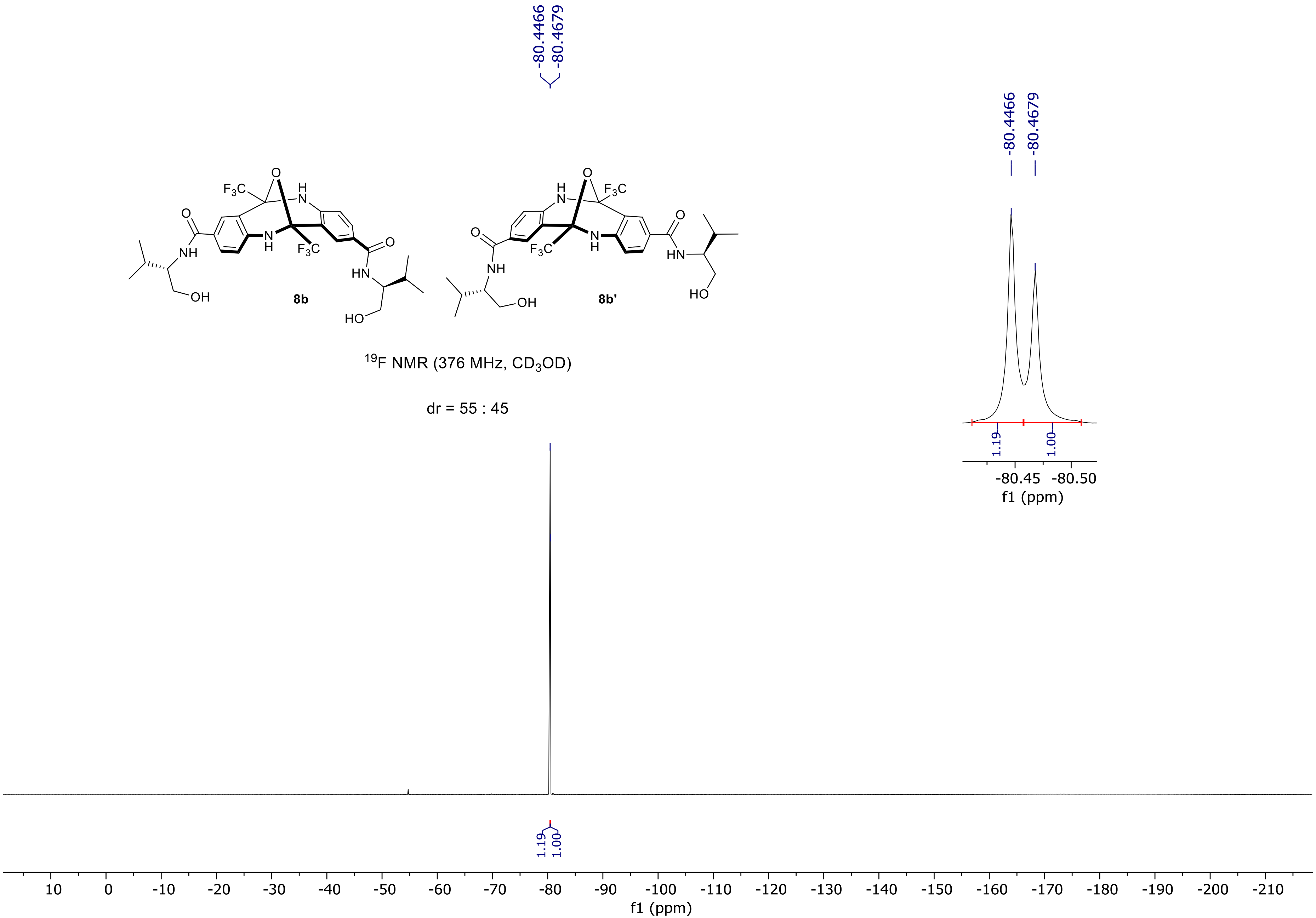
-80.4466
-80.4679
8b
8b'
$^{19}F$ NMR (376 MHz, $CD_3OD$)
dr = 55 : 45
1.19
1.00
-80.45
-80.50
f1 (ppm)
10 0 -10 -20 -30 -40 -50 -60 -70 -80 -90 -100 -110 -120 -130 -140 -150 -160 -170 -180 -190 -200 -210
f1 (ppm)

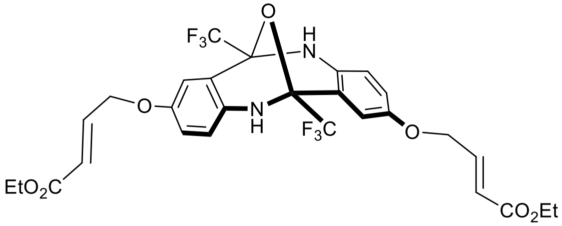


**9**

$^{1}$H NMR (400 MHz, $CDCl_3$)

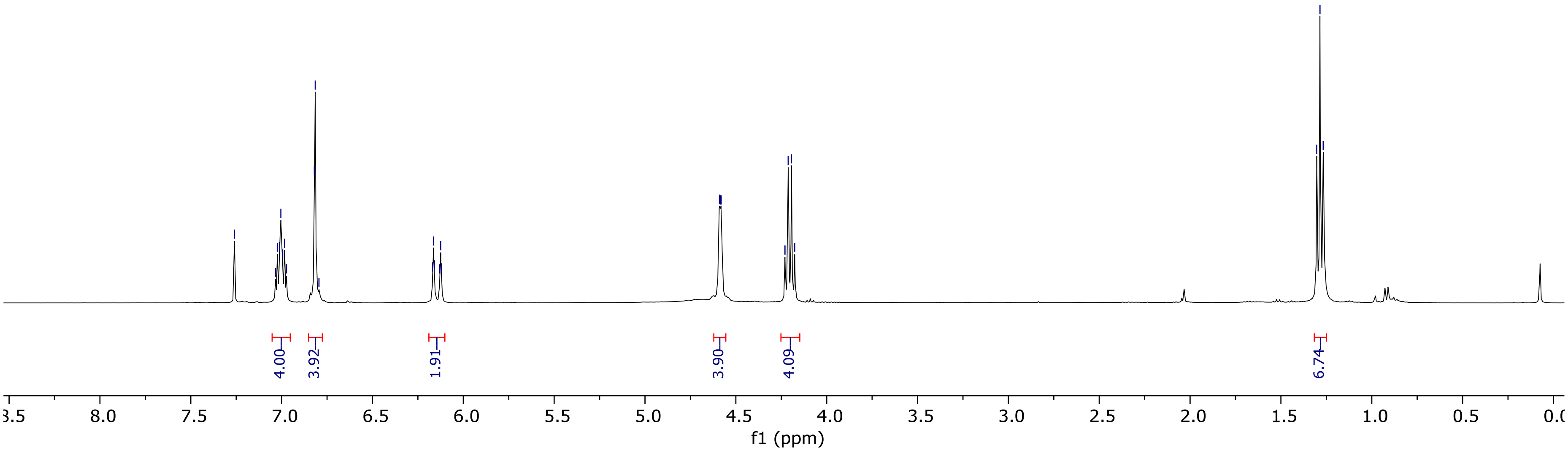

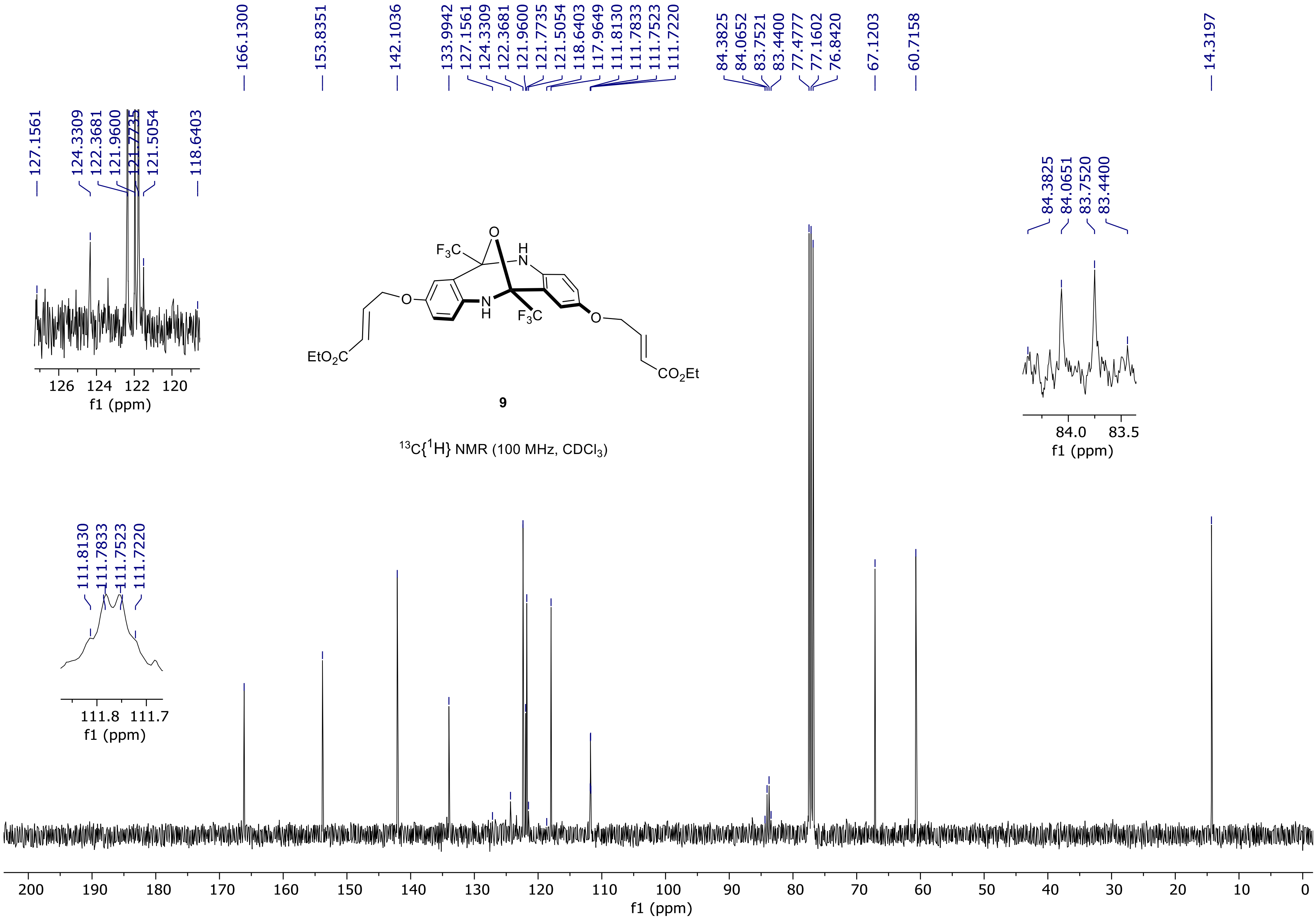
166.1300
153.8351
142.1036
133.9942
127.1561
124.3309
122.3681
121.9600
121.7735
121.5054
118.6403
117.9649
111.8130
111.7833
111.7523
111.7220
84.3825
84.0652
83.7521
83.4400
77.4777
77.1602
76.8420
67.1203
60.7158
14.3197
F3C
O
H
N
N
H
F3C
O
O
EtO2C
CO2Et
9
13C{1H} NMR (100 MHz, CDCl3)
f1 (ppm)

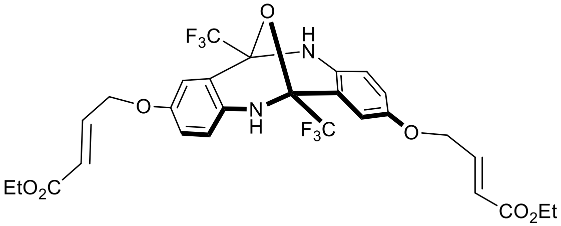


**9**

$^{19}$F NMR (376 MHz, $CDCl_3$)

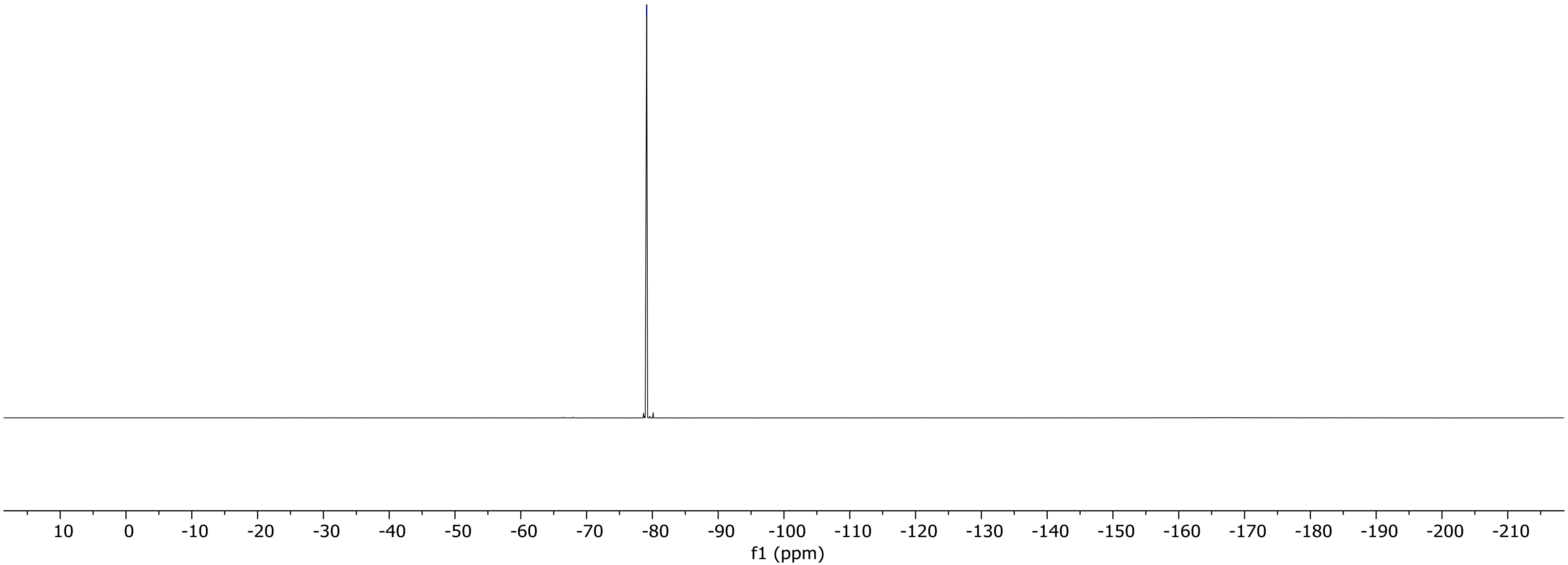

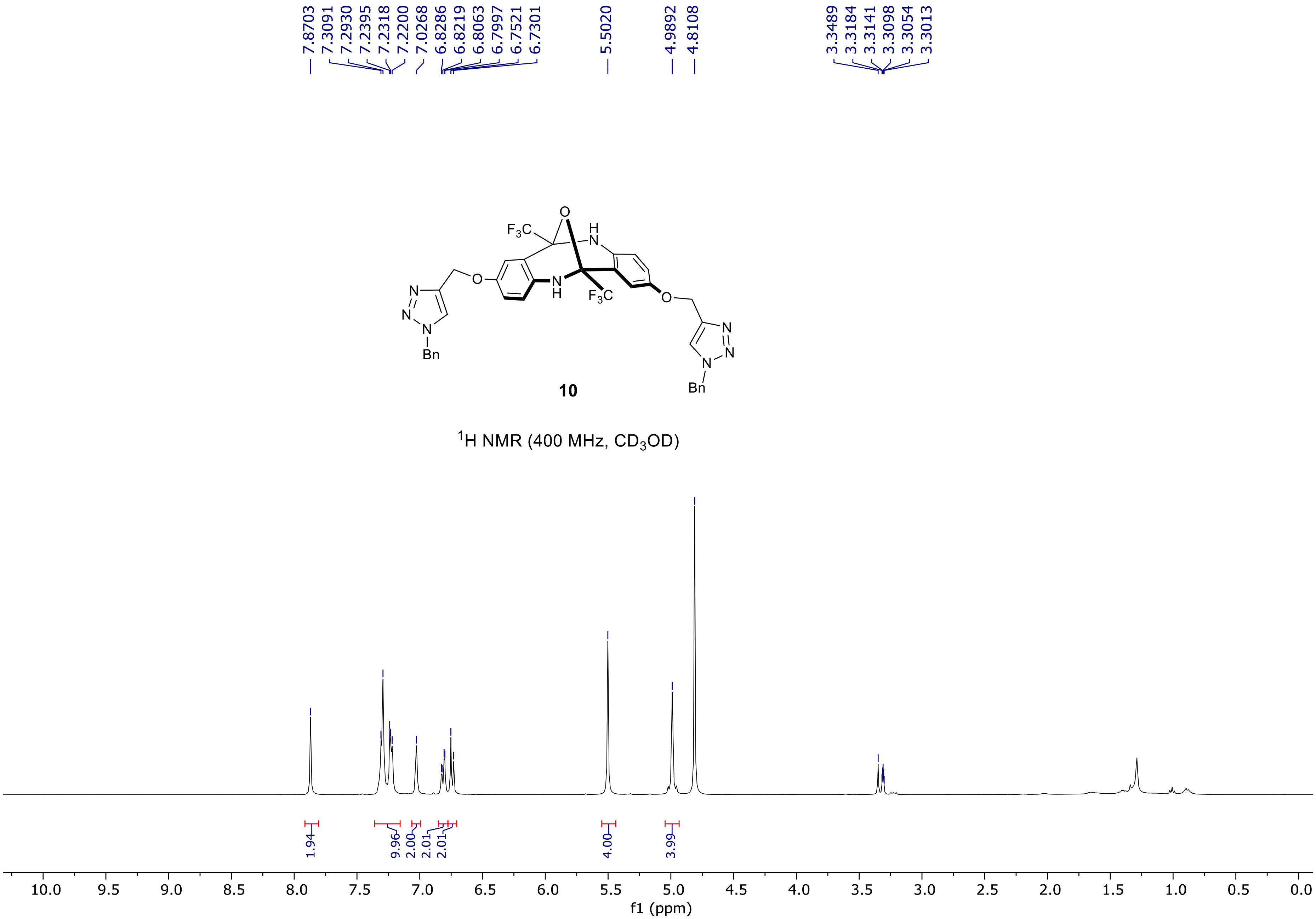
7.8703
7.3091
7.2930
7.2395
7.2318
7.2200
7.0268
6.8286
6.8219
6.8063
6.7997
6.7521
6.7301
5.5020
4.9892
4.8108
3.3489
3.3184
3.3141
3.3098
3.3054
3.3013
F3C
O
H
N
N
H
F3C
O
O
N
N
N
Bn
N
N
N
Bn
10
1H NMR (400 MHz, CD3OD)
1.94
9.96
2.00
2.01
2.01
4.00
3.99
10.0
9.5
9.0
8.5
8.0
7.5
7.0
6.5
6.0
5.5
5.0
4.5
4.0
3.5
3.0
2.5
2.0
1.5
1.0
0.5
0.0
f1 (ppm)

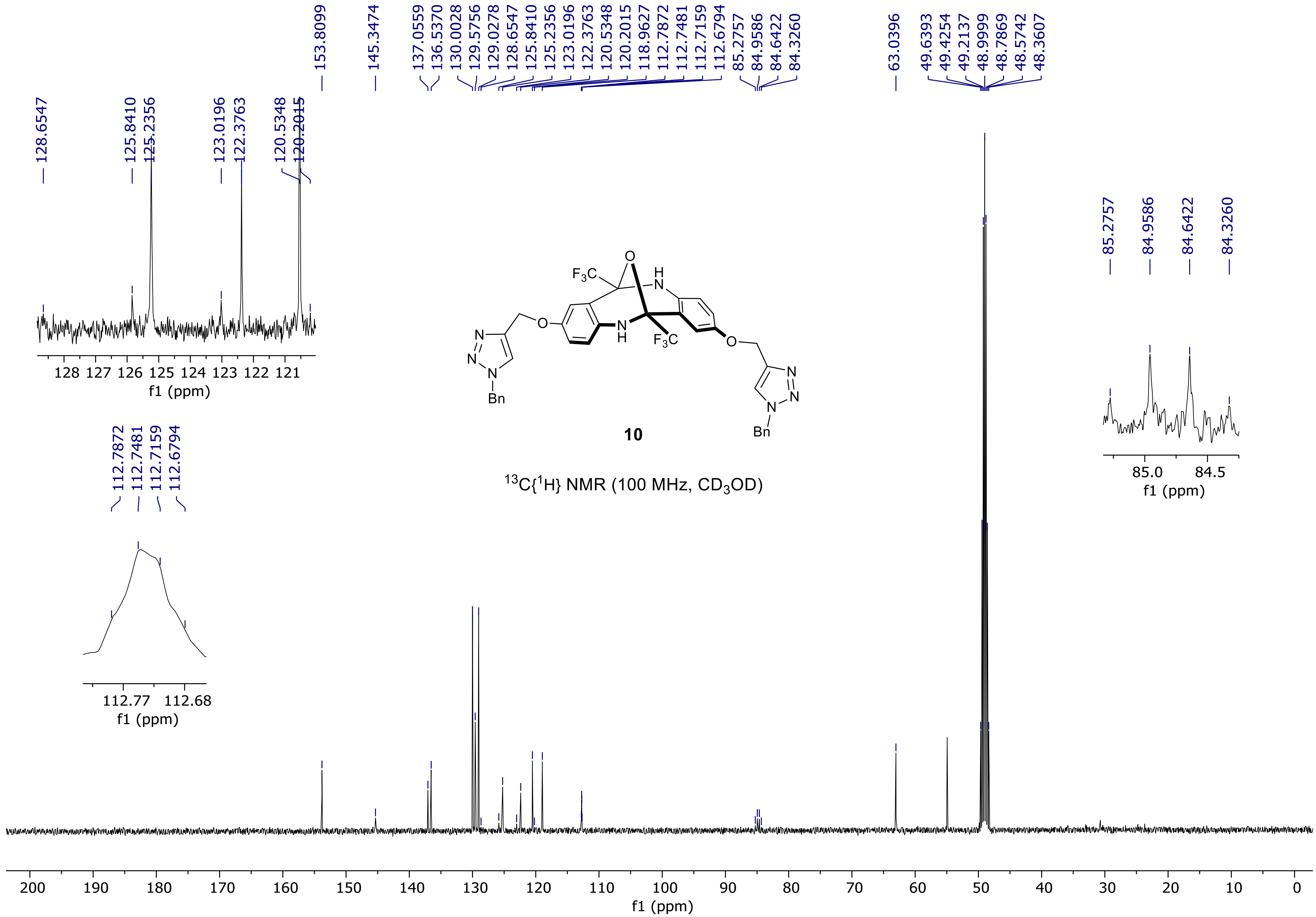
153.8099
145.3474
137.0559
136.5370
130.0028
129.5756
129.0278
128.6547
125.8410
125.2356
123.0196
122.3763
120.5348
120.2015
118.9627
112.7872
112.7481
112.7159
112.6794
85.2757
84.9586
84.6422
84.3260
63.0396
49.6393
49.4254
49.2137
48.9999
48.7869
48.5742
48.3607
$F_3C$
O
H
N
N
H
$F_3C$
O
O
N
N
N
Bn
N
N
N
Bn
10
$^{13}C\{^1H\}$ NMR (100 MHz, $CD_3OD$)
f1 (ppm)

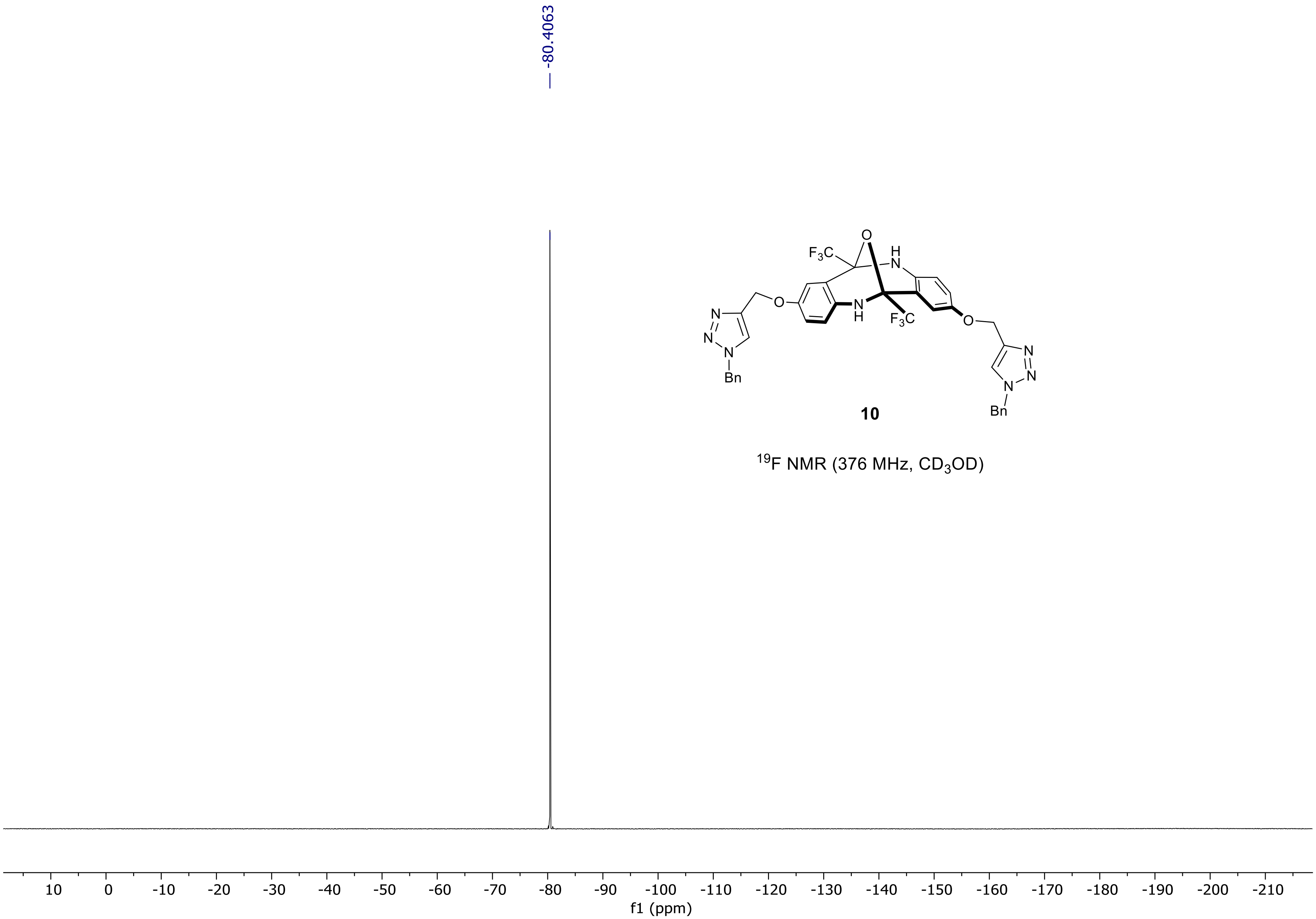


**10**

$^{19}$F NMR (376 MHz, $CD_3OD$)
